# Supplementary material for: Freezing stress affects the efficacy of clodinafop-propargyl and 2,4-D plus MCPA on wild oat (Avena ludoviciana Durieu) and turnipweed [Rapistrum rugosum (L.) All.] in wheat (Triticum aestivum L.)
Source: PLoS One. 2022 Oct 6;17(10):e0274945. doi: 10.1371/journal.pone.0274945 (PMC9536574; doi:10.1371/journal.pone.0274945)
Supplement: Attachment — Submitted filename: PONE-D-21-33766_R1.pdf [file pone.0274945.s004.pdf]

PLOS ONE

Freezing stress affects the efficacy of clodinafop-propargyl and 2,4-D plus MCPA on wild oat and turnipweed in wheat

--Manuscript Draft--

Manuscript Number: PONE-D-21-33766R1

Article Type: Research Article

Full Title: Freezing stress affects the efficacy of clodinafop-propargyl and 2,4-D plus MCPA on wild oat and turnipweed in wheat

Short Title: Freezing and herbicide efficacy

Corresponding Author: Mehdi Rastgoo
Ferdowsi University of Mashhad
Mashhad, IRAN, ISLAMIC REPUBLIC OF

Keywords: GR50; Herbicide efficacy; LD50; Selectivity index

Abstract: The occurrence of freezing stress around herbicides application is one of the most important factors influencing their performance. This experiment was performed to evaluate the efficacy of clodinafop-propargyl and 2,4-D plus MCPA, the most important herbicides used in wheat fields in Iran, under the influence of a freezing treatment (-4°C). Wheat and its two common weeds, winter wild oat and turnipweed, were exposed to the freezing treatment for three nights from 7:00 P.M. to 5:00 A.M. before and after herbicide application, and their response was compared with plants that did not grow under freezing stress. Under no freezing (NF) and freezing after spray (FAS) conditions, winter wild oat was completely controlled with the recommended dose of clodinafop-propargyl (64 g ai ha^{-1}). However, the survival percentage of winter wild oat in the freezing before spray (FBS) of clodinafop-propargyl 64 g ha^{-1} was 7%, and it was completely controlled with twice the recommended dose (128 g ha^{-1}). Under NF conditions and FAS treatment, turnipweed was completely controlled with twice the recommended dose of 2,4-D plus MCPA (2025 g ha^{-1}). However, in the FBS treatment, the survival of turnipweed was 7% (2025 g ha^{-1}). The LD_{50} (dose required to control 50% of individuals in the population) and GR_{50} (dose causing 50% growth reduction of plants) rankings were NF<FBS<FAS for clodinafop-propargyl and NF<FAS<FBS for 2,4-D plus MCPA. Selectivity index for clodinafop-propargyl in NF conditions, FBS, and FAS treatments was 2.4, 0.91, and 0.78, respectively, and for 2,4-D plus MCPA it was 2.6, 0.12, and 0.88, respectively. According to the results of this experiment, it can be stated that the freezing stress after the spraying of clodinafop-propargyl and the freezing stress before a spray of 2,4-D plus MCPA would further reduce the efficacy of these herbicides.

Order of Authors:
Alireza Hasanfard
Mehdi Rastgoo
Ebrahim Izadi Darbandi
Ahmad Nezami
Bhagirath Singh Chauhan

Opposed Reviewers:

Response to Reviewers: Manuscript Number: PONE-D-21-33766

Manuscript title: Freezing stress affects the efficacy of clodinafop-propargyl and 2,4-D plus MCPA on wild oat and turnipweed in wheat

Dear Editor

Thank you very much for arranging the review of our manuscript. We greatly appreciate the reviewers for their complimentary comments and suggestions. We hope that you find our responses satisfactory and that the manuscript is now acceptable.
Sincerely,

Powered by Editorial Manager® and ProduXion Manager® from Aries Systems Corporation

Corresponding author

Response to the comments of the reviewer #1
We would like to thank the reviewer for the careful and thorough reading of this manuscript and the thoughtful comments and constructive suggestions, which help improve the manuscript quality. As indicated below, we have checked all the general and specific comments provided by the referees and have made necessary changes according to their indications, which are highlighted in yellow.
Reviewer #1: Unpredicted variability in temperature is associated with frequent extreme low-temperature events. And it is threatening global food security. Due to the lack of information on the efficacy of herbicides under freezing stress, this research was conducted to evaluate the efficacy of clodinafop-propargyl and 2,4-D plus MCPA on wild oat and turnipweed under freezing stress before and after herbicide application. The research results have guiding significance for production, but the article still needs to be revised and improved.

（1）The introduction part should increase the impact of global climate change on the occurrence of low temperature adversity events. And its impact on wheat production.
-The requested information has been added to the text.
（2）Experimental site and plant material part：Explain the amount of fertilizer used and the ratio of N, P, K.
-We did not use fertilizer in this experiment.

（3）The source of the meteorological data in Figure 1 should be clearly explained.
-The requested information has been added to the text.

（4）Results part: The description of the results needs to be more in-depth. For example, how much does FBS significantly increase or decrease compared to NF?
-We respect the reviewer's opinion, but according to the results of the clodinafop-propargyl, we have written the requested information in another expression (gray highlight), and its re-expression is repetitive. But in the case of 2,4-D plus MCPA, the results were explained in more detail, and the requested information was added to the text (yellow highlight).

（5）The discussion part is not deep enough, some physiological discussions should be added.
-The requested information has been added to the text.

（6）The description in the conclusion part needs to be refined to increase readability.
-The requested information was added to the text. Clear text added.

（7）There are grammatical and editorial issues somewhere in this paper. Many of them could cause difficulties to the readers.
-We respect the reviewer's opinion, but a Native English-speaker colleague has edited the manuscript's text. We rechecked the text.

（8）The article does not have data on wheat yield. It would be very good if we could increase the data on the effects of herbicides before and after low temperature on wheat yield.
-It is an exciting suggestion. We are going to study the wheat yield in future studies. In this study, we focused on the effect of freezing stress on herbicide efficacy in the early stages of plant species. In general, it is predicted that wheat yield will decrease due to herbicide efficiency and its adverse effects on the crop (selectivity index).

Response to the comments of the reviewer #2
We greatly appreciate the reviewer's positive comments. As indicated below, we have checked all the general and specific comments provided by the referees and have made necessary changes according to their indications, which are highlighted in yellow.
Reviewer #2: Dear Authors,
Reviewer comments PONE-D-21-33766
The manuscript entitled „Freezing stress affects the efficacy of clodinafop-propargyl and 2,4-D plus MCPA on wild oat and turnipweed in wheat“ represents a useful study

focused on the impact of freezing stress on the efficiency of two herbicides treatments, clodinafop-propargyl and 2,4-D plus MCPA treatments, resulting in the statement that freezing stress attenuates the efficiency of herbicides in weed elimination as indicated by a decreased selectivity index. This result also indicates that freezing increases potential harmfulness of these herbicides for wheat as a crop.
I have only a few minor comments on the present manuscript:
In Materials and methods, Statistical analysis, as well as in Results, Table 1, a kind of a post-hoc test following ANOVA analysis used for determination of significant differences, has to be specified both in Materials and methods, Statistical analysis section and in results, Table 1 legend. Moreover, in Table 1 legend, it has to be clearly stated that the presented data represent mean±SE, and the number of replicates has to be specified.
-The requested information has been added to the text and Table 1.
Further formal comments on the manuscript text:
Abstract, lines 37-38: Add a comma both preceding and following the words „for 2,4-D plus MCPA,…“
-The requested revision was applied to the text.
Introduction, line 53: Use the term „winter cereals“ instead of „autumn cereals“ for autumn-sown cereals which have vernalization requirement thus they require winter temperatures prior to flowering.

-The requested revision was applied to the text.
Introduction, line 59: Use the term „optimum climatic conditions“ instead of „normal climatic conditions“.
-The requested revision was applied to the text.
Materials and methods, line 109: Replace the verb „obtain“ with „induce“ in the statement „To induce plant cold acclimation, the plants were kept outdoors,…“
-The requested revision was applied to the text.
Materials and methods, line 125: The authors wrote that „Freezing treatment was performed using a thermogradient freezer.“ However, basic information on the freezer has to be given including the manufacturer (company). Was the freezing treatment always just to -4 °C?
-The requested information was added to the text.
-Trend of temperature decrease in thermogradient freezer during the night from 7:00 P.M. to 5:00 A.M. (Please see Fig. 2 and gray highlight)
TimeTemperature (℃)
7 P.M.5
12 A.M. Reach a temperature of 0
1 A.M. kept at 0
2 A.M.Reach a temperature of -2
3 A.M.kept at -2
4 A.M.Reach a temperature of -4
5 A.M.kept at -4
5:30 A.M.Removed from the freezer

Line 175: Remove „the“ preceding the term „relative potency“.
-The requested revision was applied to the text.
Line 182: Modify the statement as follows: „Relative potency < 1 indicates the decreased effectiveness of the herbicides under freezing stress with respect to optimum conditions.“
-The requested revision was applied to the text.
Results, Figure 4 legend, line 209; Figure 5 legend, line 246: Write rather „64 g ha-1“, not „64 g ai ha-1“.
-The requested revision was applied to the text.
Results, line 252: Use a plural form of the verb „were“ instead of „was“ in the statement „The GR50 of NF, FBS, and FAS were 176, 299, and 208 g ha-1, respectively…“
-The requested revision was applied to the text.
Line 263: Remove „the“ preceding the term „selectivity index“ and modify the statement as follows: „As shown in Table 2, selectivity index values for clodinafop-propargyl in NF, FBS, and FAS treatments were 2.4, 0.91, and 0.78, respectively, and, for 2,4-D plus MCPA, they were 2.6, 0.12, and 0.88, respectively.“
-The requested revision was applied to the text.
Discussion, line 299: Remove „the“ Preceding the chemical name „clodinafop propargyl“.

Powered by Editorial Manager® and ProduXion Manager® from Aries Systems Corporation

	-The requested revision was applied to the text.<br>Discussion, line 332: Replace the word „less“ with „lower“ in the statement „The relative potency values of clodinafop-propargyl and 2,4-D plus MCPA were lower than one under both FBS and FAS treatments in the present experiment.“<br>-The requested revision was applied to the text.<br>Discussion, line 343: Modify the statement into a plural form as follows: „The LD10 values of 2,4-D plus MCPA in NF were 89% and 48% higher compared to FBS and FAS, respectively.“<br>-The requested revision was applied to the text.<br><br>We look forward to hearing from you regarding our submission. We would be glad to respond to any further questions and comments that you may have.
**Additional Information:**	
**Question**	**Response**
**Financial Disclosure**<br><br>Enter a financial disclosure statement that describes the sources of funding for the work included in this submission. Review the submission guidelines for detailed requirements. View published research articles from *PLOS ONE* for specific examples.<br><br>This statement is required for submission and **will appear in the published article** if the submission is accepted. Please make sure it is accurate.<br><br>**Unfunded studies**<br>Enter: *The author(s) received no specific funding for this work.*<br><br>**Funded studies**<br>Enter a statement with the following details:<br>• Initials of the authors who received each award<br>• Grant numbers awarded to each author<br>• The full name of each funder<br>• URL of each funder website<br>• Did the sponsors or funders play any role in the study design, data collection and analysis, decision to publish, or preparation of the manuscript?<br>• **NO** - Include this sentence at the end of your statement: *The funders had no role in study design, data collection and analysis, decision to publish, or preparation of the manuscript.*<br>• **YES** - Specify the role(s) played.<br><br>* typeset	No
**Competing Interests**	The authors have declared that no competing interests exist.

Use the instructions below to enter a competing interest statement for this submission. On behalf of all authors, disclose any competing interests that could be perceived to bias this work—acknowledging all financial support and any other relevant financial or non-financial competing interests.<br><br>This statement is required for submission and **will appear in the published article** if the submission is accepted. Please make sure it is accurate and that any funding sources listed in your Funding Information later in the submission form are also declared in your Financial Disclosure statement.<br><br>View published research articles from *PLOS ONE* for specific examples.<br><br>**NO authors have competing interests**<br><br>Enter: *The authors have declared that no competing interests exist.*<br><br>**Authors with competing interests**<br><br>Enter competing interest details beginning with this statement:<br><br>*I have read the journal's policy and the authors of this manuscript have the following competing interests: [insert competing interests here]*<br><br>* typeset	
**Ethics Statement**<br><br>Enter an ethics statement for this submission. This statement is required if the study involved:<br><br>• Human participants<br>• Human specimens or tissue<br>• Vertebrate animals or cephalopods<br>• Vertebrate embryos or tissues	N/A

Powered by Editorial Manager® and ProduXion Manager® from Aries Systems Corporation

• Field research<br><br>Write "N/A" if the submission does not require an ethics statement.<br><br>General guidance is provided below. Consult the submission guidelines for detailed instructions. **Make sure that all information entered here is included in the Methods section of the manuscript.**	

Powered by Editorial Manager® and ProduXion Manager® from Aries Systems Corporation

**Format for specific study types**<br><br>**Human Subject Research (involving human participants and/or tissue)**<br>• Give the name of the institutional review board or ethics committee that approved the study<br>• Include the approval number and/or a statement indicating approval of this research<br>• Indicate the form of consent obtained (written/oral) or the reason that consent was not obtained (e.g. the data were analyzed anonymously)<br><br>**Animal Research (involving vertebrate animals, embryos or tissues)**<br>• Provide the name of the Institutional Animal Care and Use Committee (IACUC) or other relevant ethics board that reviewed the study protocol, and indicate whether they approved this research or granted a formal waiver of ethical approval<br>• Include an approval number if one was obtained<br>• If the study involved *non-human primates*, add *additional details* about animal welfare and steps taken to ameliorate suffering<br>• If anesthesia, euthanasia, or any kind of animal sacrifice is part of the study, include briefly which substances and/or methods were applied<br><br>**Field Research**<br><br>Include the following details if this study involves the collection of plant, animal, or other materials from a natural setting:<br>• Field permit number<br>• Name of the institution or relevant body that granted permission	
**Data Availability**<br><br>Authors are required to make all data underlying the findings described fully available, without restriction, and from the time of publication. PLOS allows rare exceptions to address legal and ethical concerns. See the PLOS Data Policy and FAQ for detailed information.	Yes - all data are fully available without restriction

Powered by Editorial Manager® and ProduXion Manager® from Aries Systems Corporation

A Data Availability Statement describing where the data can be found is required at submission. Your answers to this question constitute the Data Availability Statement and **will be published in the article**, if accepted.<br><br>**Important:** Stating 'data available on request from the author' is not sufficient. If your data are only available upon request, select 'No' for the first question and explain your exceptional situation in the text box.<br><br>Do the authors confirm that all data underlying the findings described in their manuscript are fully available without restriction?	
**Describe where the data may be found in full sentences. If you are copying our sample text, replace any instances of XXX with the appropriate details.**<br><br>• If the data are **held or will be held in a public repository**, include URLs, accession numbers or DOIs. If this information will only be available after acceptance, indicate this by ticking the box below. For example: *All XXX files are available from the XXX database (accession number(s) XXX, XXX.).*<br>• If the data are all contained **within the manuscript and/or Supporting Information files**, enter the following: *All relevant data are within the manuscript and its Supporting Information files.*<br>• If neither of these applies but you are able to provide **details of access elsewhere**, with or without limitations, please do so. For example:<br><br>*Data cannot be shared publicly because of [XXX]. Data are available from the XXX Institutional Data Access / Ethics Committee (contact via XXX) for researchers who meet the criteria for access to confidential data.*<br><br>*The data underlying the results presented in the study are available from (include the name of the third party*	All relevant data are within the manuscript and its Supporting Information files.

and contact information or URL).<br>• This text is appropriate if the data are owned by a third party and authors do not have permission to share the data.<br><br>* typeset	
Additional data availability information:	Tick here if the URLs/accession numbers/DOIs will be available only after acceptance of the manuscript for publication so that we can ensure their inclusion before publication.

Powered by Editorial Manager® and ProduXion Manager® from Aries Systems Corporation

Cover letter

Dear Editors-in-Chief

We wish to submit an original research article entitled “Freezing stress affects the efficacy of clodinafop-propargyl and 2,4-D plus MCPA on wild oat and turnipweed in wheat” for consideration by Plos One.

We confirm that this work is original and has not been published elsewhere, nor is it currently under consideration for publication elsewhere. The authors have been participated in the preparation of the manuscript, and have given their permission to submit this manuscript with its present format.

The efficacy of herbicides is significantly affected by environmental conditions before, during, and after application. The occurrence of severe freezing that causes damage to plant leaves will have adverse effects on the activity and efficacy of foliar-applied herbicides. The present experiment aimed to investigate:

1. The effect of freezing stress on the performance of herbicides (clodinafop-propargyl and 2,4-D plus MCPA)
2. The effect of freezing stress on the relative potency
3. The selective performance of herbicides.

The results of this study could be very informative for the researchers, farmers, and herbicide producers.

We have no conflicts of interest to disclose.

Please address all correspondence concerning this manuscript to me at m.rastgoo@um.ac.ir

Thank you for your consideration of this manuscript.

Sincerely,

Dr Mehdi Rastgoo
Associate professor
Department of Agrotechnology, Faculty of Agriculture, Ferdowsi University of Mashhad, Iran
Corresponding author

Journal Requirements:

When submitting your revision, we need you to address these additional requirements.

1. Please ensure that your manuscript meets PLOS ONE's style requirements, including those for file naming. The PLOS ONE style templates can be found at

https://journals.plos.org/plosone/s/file?id=wjVg/PLOSOne_formatting_sample_main_body.pdf and

https://journals.plos.org/plosone/s/file?id=ba62/PLOSOne_formatting_sample_title_authors_affiliations.pdf

- The manuscript matches the style of PLOS ONE.

2. Thank you for stating the following financial disclosure:

"No"

At this time, please address the following queries:

a) Please clarify the sources of funding (financial or material support) for your study. List the grants or organizations that supported your study, including funding received from your institution.

- In the experimental implementation section, Ferdowsi University of Mashhad has supported us to a limited extent.

b) State what role the funders took in the study. If the funders had no role in your study, please state: “The funders had no role in study design, data collection and analysis, decision to publish, or preparation of the manuscript.”

- The funders had no role in study design, data collection and analysis, decision to publish, or preparation of the manuscript

c) If any authors received a salary from any of your funders, please state which authors and which funders.

- None

d) If you did not receive any funding for this study, please state: “The authors received no specific funding for this work.”

- Please include your amended statements within your cover letter; we will change the online submission form on your behalf.

3. Thank you for stating the following in the Acknowledgments Section of your manuscript:

"We are grateful to Ferdowsi University of Mashhad, Iran, for providing financial support for present research (Project No.3.49759). No additional external funding was received for this study."

We note that you have provided funding information that is not currently declared in your Funding Statement. However, funding information should not appear in the Acknowledgments section or other areas of your manuscript. We will only publish funding information present in the Funding Statement section of the online submission form.
Please remove any funding-related text from the manuscript and let us know how you would like to update your Funding Statement. Currently, your Funding Statement reads as follows:

" We are grateful to Ferdowsi University of Mashhad, Iran, for providing financial support for present research (Project No.3.49759). No additional external funding was received for this study."

Please include your amended statements within your cover letter; we will change the online submission form on your behalf.

We are grateful to Ferdowsi University of Mashhad, Iran, for providing financial support for present research (Project No.3.49759). No additional external funding was received for this study.

4. In your Data Availability statement, you have not specified where the minimal data set underlying the results described in your manuscript can be found. PLOS defines a study's minimal data set as the underlying data used to reach the conclusions drawn in the manuscript and any additional data required to replicate the reported study findings in their entirety. All PLOS journals require that the minimal data set be made fully available. For more information about our data policy, please see http://journals.plos.org/plosone/s/data-availability.

"Upon re-submitting your revised manuscript, please upload your study’s minimal underlying data set as either Supporting Information files or to a stable, public repository and include the relevant URLs, DOIs, or accession numbers within your revised cover letter. For a list of acceptable repositories, please see http://journals.plos.org/plosone/s/data-availability#loc-recommended-repositories. Any potentially identifying patient information must be fully anonymized.

Important: If there are ethical or legal restrictions to sharing your data publicly, please explain these restrictions in detail. Please see our guidelines for more information on what we consider unacceptable restrictions to publicly sharing data: http://journals.plos.org/plosone/s/data-availability#loc-unacceptable-data-access-restrictions. Note that it is not acceptable for the authors to be the sole named individuals responsible for ensuring data access.

We will update your Data Availability statement to reflect the information you provide in your cover letter.

- Experiment data are available and upload as Supporting Information files

5. PLOS requires an ORCID iD for the corresponding author in Editorial Manager on papers submitted after December 6th, 2016. Please ensure that you have an ORCID iD and that it is validated in Editorial Manager. To do this, go to ‘Update my Information’ (in the upper left-hand corner of the main menu), and click on the Fetch/Validate link next to the ORCID field. This will take you to the ORCID site and allow you to create a new iD or authenticate a pre-existing iD in Editorial Manager. Please see the following video for instructions on linking an ORCID iD to your Editorial Manager account: https://www.youtube.com/watch?v=_xcclfuvtxQ

ORCID iD validated in Editorial Manager.
Author for correspondence: Mehdi Rastgoo, ORCID iD: https://orcid.org/0000-0002-1974-8727

Manuscript Click here to access/download;Manuscript;Manuscript.docx

Freezing stress affects the efficacy of clodinafop-propargyl and 2,4-D plus MCPA on wild oat and turnipweed in wheat

Short running title: Freezing and herbicide efficacy

Alireza Hasanfard[1], Mehdi Rastgoo[1], Ebrahim Izadi Darbandi[1], Ahmad Nezami[1], Bhagirath Singh Chauhan[2]

[1] Department of Agrotechnology, Faculty of Agriculture, Ferdowsi University of Mashhad, Iran

[2] Queensland Alliance for Agriculture and Food Innovation (QAAFI) *and* School of Agriculture and Food Sciences (SAFS), The University of Queensland, Gatton, Queensland, Australia

***Corresponding author:** m.rastgoo@um.ac.ir

Abstract

The occurrence of freezing stress around herbicides application is one of the most important factors influencing their performance. This experiment was performed to evaluate the efficacy of clodinafop-propargyl and 2,4-D plus MCPA, the most important herbicides used in wheat fields in Iran, under the influence of a freezing treatment (-4℃). Wheat and its two common weeds, winter wild oat and turnipweed, were exposed to the freezing treatment for three nights from 7:00 P.M. to 5:00 A.M. before and after herbicide application, and their response was compared with plants that did not grow under freezing stress. Under no freezing (NF) and freezing after spray (FAS) conditions, winter wild oat was completely controlled with the recommended dose of clodinafop-propargyl (64 g ai ha^{-1}). However, the survival percentage of winter wild oat in the freezing before spray (FBS) of clodinafop-propargyl 64 g ha^{-1} was 7%, and it was completely controlled with twice the recommended dose (128 g ha^{-1}). Under NF conditions and FAS treatment, turnipweed was completely controlled with twice the recommended dose of 2,4-D plus MCPA (2025 g ha^{-1}). However, in the FBS treatment, the survival of turnipweed was 7% (2025 g ha^{-1}). The LD_{50} (dose required to control 50% of individuals in the population) and GR_{50} (dose causing 50% growth reduction of plants) rankings were NF<FBS<FAS for clodinafop-propargyl and NF<FAS<FBS for 2,4-D plus MCPA. Selectivity index for clodinafop-propargyl in NF conditions, FBS, and FAS treatments was 2.4, 0.91, and 0.78, respectively, and, for 2,4-D plus MCPA, it was 2.6, 0.12, and 0.88, respectively. According to the results of this experiment, it can be stated that the freezing stress after the spraying of clodinafop-propargyl and the freezing stress before a spray of 2,4-D plus MCPA would further reduce the efficacy of these herbicides.

Keywords: GR_{50}, Herbicide efficacy, LD_{50}, Selectivity index

Introduction

Winter wild oat (*Avena ludoviciana* Durieu) and turnipweed [*Rapistrum rugosum* (L.) All.] are the most important annual weeds of winter wheat (*Triticum aestivum* L.) in Iran, which cause significant reductions in wheat yield [1, 2]. The high reproduction potential and fitness to freezing stress, which results in a wide dispersal of weed seeds are the major challenges in the management of winter annual weeds like wild oat and turnipweed. Therefore, in cold regions of Iran, the dispersal and geographical distribution of these weeds are more significant than other weed species [3].

One of the most important consequences of climate change is the change in glaciers' occurrence patterns [4]. A sharp drop in temperature after high temperatures leads to crop damage. Increasing autumn temperatures in Iran, resulting from climate change, have led to reduced freezing tolerance of autumn crops [3]. In other words, autumn crops will not tolerate freezing stress due to the lack of hardening process in autumn. As a result, they will be damaged by decreasing temperatures during winter, and their yield will be reduced [3, 4]. It has been reported that increasing autumn temperatures resulting from climate change in western Canada led to reduced freezing tolerance of winter cereals [5]. In other words, due to the lack of optimal cold acclimation in autumn, crops will not be able to tolerate freezing stress. As a result, they will be damaged by decreasing temperatures during winter or by the occurrence of late spring frosts [6]. However, weeds generally have a higher ability to tolerate freezing stress [7]. Based on this, it can be inferred that climate change is adversely changing agricultural production and can significantly change the management of current cropping systems that have evolved under optimum climatic conditions.

Hand weeding and hoeing are not common in wheat fields, and mechanical weed management methods are not effective, so chemical methods are mainly used to control weeds in

the wheat fields in Iran. Clodinafop-propargyl and 2,4-D plus MCPA are the most common herbicides used for controlling weeds in wheat fields in Iran. Due to the particular importance of this crop in food security, their wide excessive applications can be justified [8, 9]. Clodinafop-propargyl is a systemic and selective herbicide of the aryloxyphenoxypropionate (AOPP) group and used post-emergent to control annual grass weeds in wheat. This herbicide inhibits the activity of acetyl-coenzyme A carboxylase (ACCase) and thus disrupts the biosynthesis of fatty acids [10]. 2,4-D plus MCPA, synthetic auxin herbicides, is a systemic herbicide mixture registered for selective post-emergence control of annual broadleaf weeds in wheat [11].

The efficacy of herbicides is significantly affected by environmental conditions before, during, and after application [12]. It has been reported that environmental factors such as light intensity, temperature, water stress, relative humidity, and nutrient availability alter the herbicide efficacy [13, 14]. On the other hand, plant species and their growth stages also affect herbicide efficacy [15]. Numerous studies indicate that ambient temperature is one of the most influential factors affecting the performance of herbicides [14, 16, 17]. Increasing the temperature is likely to increase the absorption and translocation of some herbicides and increase their performance. In contrast, the improvement in herbicide performance at low temperatures may be due to a reduction in the rate of herbicide metabolism [17, 18]. In cold regions, freezing temperatures occur around the time of herbicide application. Therefore, the occurrence of severe freezing that causes damage to plant leaves will have adverse effects on the activity and efficacy of foliar-applied herbicides [19]. On the other hand, application of herbicides in unfavorable environmental conditions (such as adverse temperature before, during, and after herbicide application) causes stress in the crop, and this changes the selectivity index of the herbicide, which will ultimately cause damage to the

crop [20]. The selectivity index refers to the potential of a herbicide to eliminate weeds in a crop without affecting the yield or quality of the final product [21].

At the appropriate growth stage of weeds for herbicide application, the temperature usually decreases, and this issue leads to a decrease in the performance of herbicides. Due to the lack of information on the efficacy of herbicides under freezing stress, this experiment was conducted to evaluate the efficacy of clodinafop-propargyl and 2,4-D plus MCPA on wild oat and turnipweed under freezing stress before and after herbicide application.

Materials and methods

Experimental site and plant material

Seeds of wheat (cv. Mihan) were harvested from Khorasan Razavi Agricultural and Natural Resources Research and Education Center, and seeds of winter wild oat and turnipweed were collected from infested farms around Mashhad-Iran in May 2019. Experiments were conducted at the Faculty of Agriculture, the Ferdowsi University of Mashhad, Iran (Lat 36° 15′ N, Long 59° 28 E; 985 m Altitude). To break the dormancy of winter wild oat and turnipweed, their seeds were exposed to moist-chilling treatment and potassium nitrate (KNO_3) 0.2% solution for 3 days at 5℃ in the dark, respectively [3]. The seeds were placed in 9-cm diameter Petri dishes (20-25 seeds per Petri dish) on moist filter papers and kept at 20℃. After 5 days, the germination percentage of winter wild oat and turnipweed seeds was >90%. The experiment was repeated twice with three replicates. To experience the same cold acclimation, the plants were planted at approximately the same time on October 21 (first run) and 24 (second run) in 2019. Plastic pots (12 cm in diameter and 20 cm in height) were used in the trial, and pots were filled with a mixture of sand, agricultural soil, and peat moss (2:2:1 sand, agricultural soil, and peat moss, respectively). For uniform

emergence, turnipweed seeds were planted in a seedling tray and transferred to pots in the cotyledon stage. Eight germinated seeds of wild oat were planted at 4-5 cm depth below the soil surface. To induce cold acclimation, the plants were kept outdoors, grown in natural conditions (Fig. 1), and irrigated according to daily requirements until they were tested.

Fig 1. Minimum and maximum daily air temperatures at the experimental site from October 2019 to February 2020. Relative humidity of 40-50% under the natural photoperiod [3].

Experimental factors

Experimental factors included freezing stress at three conditions, including no freezing (NF), freezing before spray (FBS), and freezing after spray (FAS). Plant species included wheat, winter wild oat, and turnipweed. Herbicides were applied at 0, 6.25, 12.5, 25, 50, 100, and 200% of the recommended dose for the herbicide clodinafop-propargyl (0.8 L ha^{-1}; 8% EC) and 2,4-D plus MCPA (1.5 L ha^{-1}; 67.5% SL).

Freezing treatment and herbicide application

Under NF, the plants grew completely in natural winter conditions without exposing to freezing stress. For FBS, the plants grew up to the 3-4 leaf stage under natural conditions. They were exposed to freezing from 7:00 P.M. to 5:00 A.M. for three nights before herbicide application [19]. Freezing treatment was performed using a thermogradient freezer (Weiss Technik). The initial temperature of the freezer was 5°C; however, the freezer was cooled down at a rate of 2°C per hour to reach a temperature of -4°C. The temperature drop slope was adjusted based on the long-term meteorological statistics of Mashhad in the time range of herbicide application. The pots were kept at -4°C for 1 h and then removed from the freezer (Fig. 2).

Fig. 2 Trend of temperature decrease in thermogradient freezer during the night from 7:00 P.M. to 5:00 A.M.

Each morning at 5:30 A.M. the plants were transferred out of the freezer, kept in natural conditions until 6:30 P.M., and then returned to the freezer during the night. After three nights of freezing exposure, the pots were taken out of the freezer and sprayed with herbicides after 2 h [19]. After spraying, the plants were kept outside under natural conditions for the duration of the experiment.

In the FAS treatment, freezing stress was applied for three nights by placing the samples treated with herbicides in the thermogradient freezer. For this purpose, the plants were taken out of the freezer at 5:30 A.M., transferred to natural conditions, and then returned to the freezer at 6:30 P.M. for the overnight freezing treatment (Similar to FBS treatment). Three nights after freezing treatment, the plants were transferred to natural conditions to continue the trial process (Fig. 3).

Fig. 3 Schematic diagram of the experimental study design. Abbreviation: FBS (freezing before spray); FAS (freezing after spray).

The conditions inside the thermogradient freezer were the same for both freezing treatments before and after spray. The experiment was performed in the freezer in completely dark conditions and at the same relative humidity (35±5%) for all treatments. Temperature changes in the freezer were regularly monitored when the samples were in the freezer. For all the freezing treatments, herbicides were applied at the same time. Plants were transferred to natural conditions after three nights of freezing treatment at 5:30 A.M. Also, plants related to freezing after spray were transferred to the freezer at 6:30 P.M. On the same day, plants that were not exposed to freezing were sprayed from 7:30 A.M. to 10.30 A.M. The herbicides were applied by a researcher-made

moving boom sprayer equipped with a TeeJet 8002 flat fan nozzle (spray output volume of 200 L ha^{-1} at a pressure of 260 kPa). Plants were returned and maintained outdoor under natural conditions of winter after the treatment.

Measurement of traits

Four weeks after herbicide treatments, survival percentage (Eq. 1) and shoot dry weight were measured by placing the samples in an oven at 75°C for 48 h. Plants that produced new leaves were considered alive and those that experienced severe chlorosis and stunted growth were recorded as dead [22].

$$Survival\ \% = \frac{N1}{N0} \times 100 \qquad \text{(Eq. 1)}$$

Where, N_1 and N_0 are the numbers of alive plants four weeks after herbicide treatment and the number of alive plants before herbicide treatment, respectively.

Dose–response experiments

The response of weed traits to the doses of herbicides tested was analyzed by nonlinear regression. For this purpose, the data were fitted to a three-parameter log-logistic equation (Eq. 2) [23].

$$Y = \frac{d}{1+e^{b(\log x - \log e)}} \qquad \text{(Eq. 2)}$$

Where, Y is the response rate (survival % or dry weight) at dose x, d is the upper limit for Y, b is the slope of the curve around e, and e is the effective dose (g ai ha^{-1}). Here, e is replaceable with LD_{50} (dose required to control 50% of individuals in the population) or GR_{50} (dose causing 50% dry weight reduction of plants).

Relative potency

Relative potency was used to determine herbicide efficacy under freezing stress and no freezing stress (Eq. 3) [24].

$$Relative\ potency = \frac{ED_{50A}}{ED_{50B}} \quad \text{(Eq. 3)}$$

Where, ED_{50A} is the dose required to reduce survival by 50% in NF, and ED_{50B} is the dose needed to reduce survival by 50% in FBS or FAS. Relative potency = 1 indicates that freezing treatment (FBS and FAS) did not affect herbicide efficacy. If relative potency > 1, it indicates that the herbicides are more efficient under freezing stress. Relative potency < 1 indicates the decreased effectiveness of the herbicides under freezing stress with respect to optimum conditions.

Selectivity index

The ratio between doses that caused 10% damage to crops and 90% damage to weeds was considered a selectivity index [24] and calculated using Eq. 4.

$$Selectivity\ index\ (10{,}90) = \frac{ED_{10\ (crop)}}{ED_{90\ (weed)}} \quad \text{(Eq. 4)}$$

Where, ED_{90} is 90% of the herbicide effect on weeds survival (herbicide dose to kill 90% of the population), and ED_{10} is 10% of the herbicide impact on crop survival (a 10% effect on the crop). The higher the SI, the more selective the herbicide in the crop [24].

Statistical analysis

The experiments were conducted using a completely randomized design in factorial arrangement with three replications. Afterward, differences between treatments were compared with the standard error of mean (SEM) values. For data analysis, analysis of variance (ANOVA) was performed using the SAS software (v. 9.4, SAS Institute Inc, Cary, NC, USA). Data for both runs

were pooled as no significant differences were observed between runs ($P > 0.05$). Homogeneity of variance was confirmed using Levene's test. Analysis of biometric data was performed by the R program (v. 4.0.4) [25]. Dose-response curves were also fitted with the asymmetrical sigmoid form. LD_{50} and GR_{50} were separated using 95% confidence intervals (CI).

Results

Clodinafop-propargyl

Survival percentage and LD_{50}

The dose-response experiments were similar for NF and FAS treatments at the recommended dose (Figure 4A). Thus, in both conditions, winter wild oat was completely controlled with the recommended dose (64 g ha^{-1}). However, the survival of seedlings in the FBS treatment was 7%, and to achieve complete control, 2-fold the recommended dose (128 g ha^{-1}) was needed. The LD_{50} in NF, FBS, and FAS was 17.9, 18.1, and 23.2 g ha^{-1}, respectively (Table 1).

Fig. 4 Survival (A) and dry weight (B) of winter wild oat treated with various doses of clodinafop-propargyl under different freezing treatments. Abbreviation: NF (no freezing); FBS (freezing before spray): FAS (freezing after spray). The recommended dose of clodinafop-propargyl in Iran is 64 g ha^{-1}.

Table 1 The dose of clodinafop-propargyl and 2,4-D plus MCPA required for 50% mortality (LD_{50}) and the dose that caused 50% inhibition of growth noted by biomass (GR_{50}) of winter wild oat and turnipweed under different freezing treatments.

Herbicide	Freezing Treatment^a	$b^b \pm SE^a$	$d^c \pm SE$	$LD_{50} \pm SE$	95% CI^e	R^2
			%	g ai ha^{-1}		
Clodinafop-propargyl	NF	2.0 ± 0.19^{**d}	100.3 ± 2.8**	17.9 ± 1.2**	15.6-20.2	0.99
	FBS	1.9 ± 0.17**	101.0 ± 2.9**	18.1 ± 1.2**	15.7-20.5	0.99
	FAS	1.9 ± 0.19**	98.7 ± 2.9**	23.2 ± 1.7**	19.8-26.6	0.96
2,4-D plus MCPA	NF	1.9 ± 0.20**	102.2 ± 3.2**	292.8 ± 21.9**	249.4-336.3	0.98
	FBS	1.5 ± 0.15**	102.4 ± 3.2**	472.8 ± 42.4**	388.8-556.8	0.98
	FAS	1.5 ± 0.14**	102.8 ± 3.5**	318.8 ± 28.9**	261.6-376.1	0.98
		$b^b \pm SE^a$	$d^c \pm SE$	$GR_{50} \pm SE$	95% CI^e	
			g pot^{-1}	g ai ha^{-1}		
Clodinafop-propargyl	NF	2.1 ± 0.15**	0.70 ± 0.02**	10.6 ± 0.52**	9.6-11.6	0.99
	FBS	2.0 ± 0.12**	0.80 ± 0.02**	11.6 ± 0.51**	10.6-12.6	0.99
	FAS	1.7 ± 0.12**	0.67 ± 0.02**	12.8 ± 0.74**	11.3-14.3	0.98
2,4-D plus MCPA	NF	2.2 ± 0.23**	0.46 ± 0.01**	176.2 ± 11.3**	153.9-198.6	0.99
	FBS	1.7 ± 0.16**	0.48 ± 0.01**	298.6 ± 21.5**	256.0-341.3	0.99
	FAS	1.6 ± 0.15**	0.47 ± 0.01**	207.8 ± 15.9**	176.2-239.4	0.99

Abbreviation: NF (no freezing); FBS (freezing before spray); FAS (freezing after spray); SE: Standard Error. b is the slope of the curve around e (LD_{50} and GR_{50}); d is the upper limit for Y (survival % or dry weight); **: significant at $p \leq 0.01$. LD_{50} and GR_{50} were separated using their 95% confidence intervals (CI). The data shows the parameter's mean ± SE. n = 6

Dry weight and GR_{50}

The dry weight of winter wild oat in NF and FAS with the recommended dose (64 g ha^{-1}) and FBS with twice the recommended dose (128 g ha^{-1}) reached 0 g (Fig. 4B). The GR_{50} of plants not exposed to freezing stress was 10.6 g ha^{-1}. The GR_{50} was 11.6 and 12.8 g ha^{-1} in FBS and FAS, respectively (Table 1).

2,4-D plus MCPA

Survival percentage and LD_{50}

The dose-response experiments of 2,4-D plus MCPA (similar to clodinafop-propargyl) were the same in NF and FAS treatments (Fig. 5A). In both conditions, turnipweed was completely

controlled with twice the recommended dose (2025 g ha^{-1}). However, in the FBS treatment, the survival of turnipweed was 7%. At the recommended dose (1012.5 g ha^{-1}), the survival of turnipweed in NF, FBS, and FAS was 3%, 33%, and 20%, respectively. The LD_{50} in NF, FBS, and FAS was 293, 473, and 319 g ha^{-1}, respectively (Table 1).

Fig. 5 Survival (A) and dry weight (B) of turnipweed treated with various doses of 2,4-D plus MCPA under different freezing treatments. Abbreviation: NF (no freezing); FBS (freezing before spray): FAS (freezing after spray). The recommended dose of 2,4-D+MCPA in Iran is 1012.5 g ha^{-1}.

Dry weight and GR_{50}

The dry weight of turnipweed in NF and FAS with twice the recommended dose (2025 g ha^{-1}) of 2,4-D plus MCPA was reduced to 0 (Fig. 5 B). However, in the FBS treatment, the dry weight of turnipweed with twice the recommended dose was 0.012 g per pot. At the recommended dose, the dry weight of turnipweed in FBS was 97% and 76% more than NF and FAS, respectively. The GR_{50} of NF, FBS, and FAS were 176, 299, and 208 g ha^{-1}, respectively (Table 1).

Relative potency

The relative potency of clodinafop-propargyl and 2,4-D plus MCPA was significantly reduced in FBS and FAS compared to NF (Fig. 6). Thus, the relative potency in FBS and FAS of clodinafop-propargyl was 0.99 and 0.77, respectively, and at 2,4-D plus MCPA was 0.62 and 0.92, respectively.

Fig. 6 Relative potency of clodinafop-propargyl and 2,4-D plus MCPA under different freezing treatments. Abbreviation: FBS (freezing before spray) and FAS (freezing after spray). The error bars indicate standard error (SE). The dashed line represents the relative potency = 1.0.

Selectivity index

As shown in Table 2, selectivity index values for clodinafop-propargyl in NF, FBS, and FAS treatments were 2.4, 0.91, and 0.78, respectively, and, for 2,4-D plus MCPA, they were 2.6, 0.12, and 0.88, respectively. For clodinafop-propargyl, the LD_{10} of wheat in NF was 127.9 g ha^{-1}. However, LD_{10} of wheat in the FBS and FAS was 53.5 and 57.1 g ha^{-1}, respectively (Table 2). For 2,4-D plus MCPA, the LD_{10} of wheat in NF, FBS, and FAS was 2380, 264, and 1236 g ha^{-1}, respectively.

Table 2 Sensitivity of plant species of wheat, winter wild oat, and turnipweed to clodinafop-propargyl and 2,4-D plus MCPA under different freezing treatments.

Freezing Treatment	Plant species	Clodinafop-propargyl: LD_{10} (wheat) and LD_{90} (winter wild oat) (g ai ha^{-1}) ± SE	Clodinafop-propargyl: SI	2,4-D plus MCPA: LD_{10} (wheat) and LD_{90} (turnipweed) (g ai ha^{-1}) ± SE	2,4-D plus MCPA: SI
NF	Wheat	127.9 ± 8.8	-	2380 ± 455	-
	Weed	53.5 ± 5.4	2.4	908 ± 108	2.6
FBS	Wheat	53.5 ± 11.0	-	264 ± 61.0	-
	Weed	59.0 ± 6.4	0.91	2144 ± 327	0.12
FAS	Wheat	57.1 ± 8.7	-	1236 ± 163	-
	Weed	73.6 ± 7.2	0.78	1400 ± 199	0.88

Abbreviation: NF; No freezing, FBS: Freezing before spray, FAS: Freezing after spray SI: Selectivity index. The selectivity index (SI) was calculated as follows: *Selectivity index (10, 90)*: $\frac{ED10\ (crop)}{ED90\ (weed)}$

Discussion

The required dose of clodinafop-propargyl for complete control of winter wild oat seedlings in FBS treatment was twice that of NF and FAS treatments. The trend of the reducing survival % in seedlings related to the FBS treatment and NF was similar. The LD_{50} for clodinafop-propargyl in freezing treatments shows that exposure of winter wild oat seedlings to freezing treatment for three nights before and after spray reduces the efficacy of this herbicide compared to NF. The LD_{50} was 22% and 23% higher in the FAS compared to the FBS and NF, respectively. Freezing treatment for three nights before and after spraying of clethodim herbicide (the mode of action similar to

clodinafop-propargyl) led to an increase in LD_{50} [19]. It decreased herbicide efficacy in rigid ryegrass populations (*Lolium rigidum* Gaud.) compared to NF.

The dose of clodinafop-propargyl required to reduce winter wild oat biomass to zero in the FBS treatment was twice that of the NF and FAS treatments. The GR_{50} is more pronounced in FBS and FAS compared to NF, indicating a decrease in the efficacy of clodinafop-propargyl. The GR_{50} was 17% and 8% higher in the FAS treatment compared to NF and FBS, respectively. In other words, the occurrence of three nights of freezing stress after herbicide application reduced the performance of clodinafop-propargyl more than other treatments. It has been reported that wild oat plants (*Avena fatua*) that grew at low temperatures were less sensitive to difenzoquat than plants that grew at high temperatures [26].

Our results clearly show that the occurrence of freezing stress around spray reduces the efficacy of clodinafop-propargyl, especially in the treatment of FAS. Herbicide translocation in the phloem in freezing conditions could decline due to damage to the phloem cells, which may lead to a decrease in the herbicide efficacy. Decreased efficacy of sethoxydim at low temperatures due to reduced herbicide translocation for Quackgrass [*Elymus repens* (L.) Gould] control was reported by Coupland [27]. In general, to maintain the herbicide efficacy under cold conditions, using clodinafop-propargyl after the freezing stress can be recommended in areas with a similar climate.

Although the three-night treatment of FBS and FAS resulted in a reduction in turnipweed control by 2,4-D plus MCPA compared to NF, this reduction was different in the two freezing treatments. At the recommended dose (1013 g ha^{-1}) in NF, almost all turnipweed plants were controlled, while the survival percentage of turnipweed in FBS and FAS treatments were 33% and 20%, respectively. Low temperatures around the time of herbicide application reduced the

effectiveness of systemic herbicides [28]. Based on LD_{50} values, the efficacy of 2,4-D plus MCPA under FBS and FAS was 1.6 and 1.1 times lower compared to NF.

FAS is possible to damage the phloem cells, which reduces the translocation of the herbicide. The efficacy of 2,4-D plus MCPA in the treatment of FBS was reduced. The decrease in the effect of 2,4-D plus MCPA in the treatment of FBS is probably due to the reduction of absorption and translocation of this herbicide in turnipweed seedlings. In this regard, an increase in efficacy of 2,4-D and glyphosate at high temperatures compared to low temperatures in common ragweed (*Ambrosia artemisiifolia* L.) and giant ragweed (*Ambrosia trifida* L.) due to the increased absorption and/or translocation at high temperatures around herbicide application has been reported by Ganie et al. [29]. It has been also reported that low temperatures reduced glufosinate activity and translocation in wild radish (*Raphanus raphanistrum* L.) [30].

Turnipweed's dry weight at the recommended dose of 2,4-D plus MCPA (1012.5 g ha^{-1}) in FBS and FAS treatments was 35.5 and 8.5 times higher than the NF, respectively. Also, GR_{50} in FBS and FAS was 41% and 15.2% higher compared to NF, respectively. Although a decrease in herbicide efficacy is evident in both FBS and FAS treatments, high GR_{50} in FBS shows an adverse effect of three nights of freezing before the application of 2,4-D plus MCPA. In other words, the efficacy of this herbicide reacts more to FBS than to FAS. In a similar study, the GR_{50} for 2,4-D increased at low temperature in common ragweed compared to high temperature [29].

The freezing stress around 2,4-D plus MCPA application reduced the efficacy of this herbicide in FBS and FAS treatments compared to NF. The decrease in the efficacy of this herbicide in the treatment of FBS is probably due to the reduction of absorption and translocation of the herbicide in the phloem during freezing. Absorption and translocation of 2,4-D plus MCPA

might be reduced at low temperatures due to the effect on herbicide penetration facilitated by physicochemical factors, including the decreased rate of diffusion, increased viscosity of the cuticle, and physiological factors comprising decreases in photosynthesis, phloem translocation, and protoplasmic streaming [29]. In Iranian wheat fields, using the 2,4-D plus MCPA before the freezing stress can be recommended to maintain the herbicide efficacy under cold conditions.

When relative potency > 1, the herbicide efficacy is higher than during normal conditions, and when relative potency < 1, the herbicide efficacy is lower than usual [24]. The relative potency values of clodinafop-propargyl and 2,4-D plus MCPA were lower than one under both FBS and FAS treatments in the present experiment. In other words, freezing stress reduced the efficacy of these herbicides. Based on the results of relative potency, the reduction of clodinafop-propargyl efficacy in the FAS treatment (33%) was more significant than in the FBS treatment (1%). Conversely, for 2,4-D plus MCPA, the reduction in efficacy was more significant in the FBS treatment (38%) compared to the FAS treatment (8%).

The selectivity index for clodinafop-propargyl and 2,4-D plus MCPA was > 1 in NF, indicating that both herbicides acted selectively in wheat. However, applying three nights of FBS and FAS of both herbicides reduced the selectivity index to less than one. In other words, freezing stress reduced the selectivity index of both herbicides, which led to phytotoxicity in wheat. For clodinafop-propargyl, LD_{10} of wheat in NF was 58% and 55% higher than FBS and FAS, respectively. The LD_{10} values of 2,4-D plus MCPA in NF were 89% and 48% higher compared to FBS and FAS, respectively. In other words, the dose required to kill 10% of wheat in both herbicides under NF was higher than FBS and FAS. Therefore, FBS and FAS increased the sensitivity of wheat to herbicide application. Freezing stress may reduce the rate of herbicide metabolism in the crop and ultimately increase its susceptibility [17].

Conclusions

The relative potency results for both herbicides were used to confirm the results of LD_{50} and GR_{50}. Thus, LD_{50} and GR_{50} were higher for clodinafop-propargyl in the FAS treatment than in NF and FBS treatments. The LD_{50} and GR_{50} were higher for the 2,4-D plus MCPA in the FBS treatment than in the NF and FAS treatments. In other words, clodinafop-propargyl was more effective than other conditions in the treatment of three nights of FAS and 2,4-D plus MCPA in the treatment of three nights of FBS. The selectivity index for clodinafop-propargyl was lower in the FAS treatment (0.78) and for 2,4-D plus MCPA was lower in the FBS treatment (0.12). The efficacy of both herbicides was affected by the FBS and FAS treatments. The efficacy of clodinafop-propargyl in the FAS treatment and the efficacy of 2,4-D plus MCPA in the FBS treatment were reduced. The values of the selectivity index of herbicides in the conditions of freezing stress showed that this index could be well used to determine the selective performance of these herbicides. Overall, according to the results of this experiment, it can be stated that FAS with ACCase inhibitor herbicides, such as clodinafop-propargyl, may reduce their effectiveness in controlling narrow leaf weeds such as winter wild oat, and FBS with auxin type herbicides, such as 2,4-D plus MCPA, reduce their efficacy in controlling broadleaf weeds such as turnipweed. FBS for clodinafop-propargyl and FAS for 2,4-D plus MCPA had a slight adverse effect on the herbicide efficacy and selectivity index. Therefore, the use of these herbicides in the mentioned conditions (FBS for clodinafop-propargyl and FAS for 2,4-D plus MCPA) is recommended. In other words, to maintain high herbicide efficacy under cold (freezing) conditions, the use of clodinafop-propargyl after the freezing stress and 2,4-D plus MCPA before the freezing stress can be recommended in areas with a similar climate to Mashhad. Finally, the use of adjuvants, alternate herbicides, and various

herbicide formulations to control grass and broadleaf weeds in Iranian wheat production is suggested to prevent the adverse effects of freezing stress on herbicides.

Conflicts of interest

The authors declare that they have no conflict of interest.

Acknowledgment

We are grateful to Ferdowsi University of Mashhad, Iran, for providing financial support for present research (Project No.3.49759). No additional external funding was received for this study.

References

1. Hatami ZM, Gherekhloo J, Rojano-Delgado AM, Osuna MD, Alcántara R, Fernández P, et al. Multiple mechanisms increase levels of resistance in *Rapistrum rugosum* to ALS herbicides. Front Plant Sci. 2016 Feb 22;7:169. https://doi.org/10.3389/fpls.2016.00169
2. Nosratti I, Sabeti P, Chaghamirzaee G, Heidari H. Weed problems, challenges, and opportunities in Iran. Crop Prot. 2020 Aug 1;134:104371. https://doi.org/10.1016/j.cropro.2017.10.007
3. Hasanfard A, Rastgoo M, Darbandi EI, Nezami A, Chauhan BS. Regeneration capacity after exposure to freezing in wild oat (*Avena ludoviciana* Durieu.) and turnipweed (*Rapistrum rugosum* (L.) All.) in comparison with winter wheat. Environ. Exp. Bot. 2021 Jan 1;181:104271. https://doi.org/10.1016/j.envexpbot.2020.104271
4. Sierra-Almeida A, Cavieres LA, Bravo LA. Warmer temperatures affect the in situ freezing resistance of the Antarctic vascular plants. Front. Plant Sci. 2018 Oct 8;9:1456. https://doi.org/10.3389/fpls.2018.01456
5. Willick IR, Tanino KK, Gusta LV. The impact of global climate change on the freezing tolerance of winter cereals in Western Canada. J. Agron. Crop Sci. 2021 Feb;207(1):88-99. https://doi.org/10.1111/jac.12447
6. Vyse K, Pagter M, Zuther E, Hincha DK. Deacclimation after cold acclimation—a crucial, but widely neglected part of plant winter survival. J. Exp. Bot. 2019 Sep 15;70(18):4595-604. https://doi.org/10.1093/jxb/erz229
7. Cici SZ, Van Acker RC. Relative freezing tolerance of facultative winter annual weeds. Can. J. Plant Sci. 2011;91(4):759-63. https://doi.org/10.4141/cjps2010-001
8. Baghestani MA, Zand E, Soufizadeh S, Beheshtian M, Haghighi A, Barjasteh A, Birgani DG, Deihimfard R. Study on the efficacy of weed control in wheat (*Triticum aestivum* L.) with tank mixtures of grass herbicides with broadleaved herbicides. Crop Prot. 2008 Jan 1;27(1):104-11. https://doi.org/10.1016/j.cropro.2007.04.013
9. Enghiad A, Ufer D, Countryman AM, Thilmany DD. An overview of global wheat market fundamentals in an era of climate concerns. Int J Agron. 2017 Jul 2;2017. https://doi.org/10.1155/2017/3931897
10. Bajwa AA, Akhter MJ, Iqbal N, Peerzada AM, Hanif Z, Manalil S, Hashim S, Ali HH, Kebaso L, Frimpong D, Namubiru H. Biology and management of Avena fatua and Avena

ludoviciana: two noxious weed species of agro-ecosystems. Environ Sci Pollut Res. 2017 Aug;24(24):19465-79. https://doi.org/10.1007/s11356-017-9810-y

11. Peterson MA, McMaster SA, Riechers DE, Skelton J, Stahlman PW. 2, 4-D past, present, and future: a review. Weed Technol. 2016 Jun;30(2):303-45. https://doi.org/10.1614/WT-D-15-00131.1
12. Collings LV, Blair AM, Gay AP, Dyer CJ, Mackay N. The effect of weather factors on the performance of herbicides to control *Alopecurus myosuroides* in winter wheat. Weed Res. 2003 Apr;43(2):146-53. https://doi.org/10.1046/j.1365-3180.2003.00327.x
13. Rocha-Pereira MR, Klar AE, Martins D, Ferreira de Souza GS, Villalba J. Efecto del estrés hídrico sobre la eficacia de los herbicidas aplicados en *Urochloa decumbens*. Cienc. Investig Agrar. 2012 Apr;39(1):211-20. http://dx.doi.org/10.4067/S0718-16202012000100018
14. Johnson BC, Young BG. Influence of temperature and relative humidity on the foliar activity of mesotrione. Weed Sci. 2002 Apr;50(2):157-61. https://doi.org/10.1614/0043-1745(2002)050[0157:IOTARH]2.0.CO;2.
15. Chahal PS, Aulakh JS, Rosenbaum K, Jhala AJ. Growth stage affects dose response of selected glyphosate-resistant weeds to premix of 2,4-D choline and glyphosate (Enlist Duo (TM) herbicide*). J. Agric. Sci. 2015 Nov 1;7(11):1. https://doi.org/10.5539/jas.v7n11p1
16. Waltz AL, Martin AR, Roeth FW, Lindquist JL. Glyphosate efficacy on velvetleaf varies with application time of day. Weed Technol. 2004 Dec;18(4):931-9. https://doi.org/10.1614/WT-03-123R3
17. Godar AS, Varanasi VK, Nakka S, Prasad PV, Thompson CR, Mithila J. Physiological and molecular mechanisms of differential sensitivity of Palmer amaranth (*Amaranthus palmeri*) to mesotrione at varying growth temperatures. Plos One. 2015 May 19;10(5):e0126731. https://doi.org/10.1371/journal.pone.0126731
18. Pline WA, Wu J, Hatzios KK. Effects of temperature and chemical additives on the response of transgenic herbicide-resistant soybeans to glufosinate and glyphosate applications. Pestic. Biochem. Phys. 1999 Oct 1;65(2):119-31. https://doi.org/10.1006/pest.1999.2437
19. Saini RK, Malone J, Preston C, Gill GS. Frost reduces clethodim efficacy in clethodim-resistant rigid ryegrass (*Lolium rigidum*) populations. Weed Sci. 2016 Jun;64(2):207-15. https://doi.org/10.1614/WS-D-15-00140.1
20. Moseley C, Hatzios KK, Hagood ES. Uptake, translocation, and metabolism of chlorimuron in soybean (*Glycine max*) and morningglory (*Ipomoea* spp.). Weed Tech. 1993 Jun;7(2):343-8. https://doi.org/10.1017/S0890037X00027706
21. Negrisoli E, Velini ED, Tofoli GR, Cavenaghi AL, Martins D, Morelli JL, Costa AG. Selectivity of pre-emergence herbicides to sugarcane treated with nematicides. Planta Daninha. 2004 Dec;22(4):567-75.
22. Powles SB, Lorraine-Colwill DF, Dellow JJ, Preston C. Evolved resistance to glyphosate in rigid ryegrass (*Lolium rigidum*) in Australia. Weed Sci. 1998 Oct;46(5):604-7.
23. Ritz C, Baty F, Streibig JC, Gerhard D. Dose-response analysis using R. Plos One. 2015 Dec 30;10(12):e0146021. https://doi.org/10.1371/journal.pone.0146021

24. Tind T, Mathiesen TJ, Jensen JE, Ritz C, Streibig JC. Using a selectivity index to evaluate logarithmic spraying in grass seed crops. Pest Manag Sci: formerly Pesticide Science. 2009 Nov;65(11):1257-62. https://doi.org/10.1002/ps.1819
25. Team RC. R Foundation for Statistical Computing, Vienna, available at: www. R-project. org. 2018.
26. Kowalczyk B, Caseley JC, McCready CC. The effect of pre-treatment temperature on some physiological responses of *Avena fatua* to difenzoquat and benzoylpropethyl. Asp Appl Biol. 1983;4:235-44.
27. Coupland D. Influence of environmental factors on the performance of sethoxydim against *Elymus repens* (L.). Weed Res. 1987 Oct;27(5):329-36. https://doi.org/10.1111/j.1365-3180.1987.tb00770.x
28. Derr J, Serensits T. Impact of application temperature on broadleaf herbicide efficacy. J Environ Hortic. 2016 Dec;34(4):123-30. https://doi.org/10.24266/0738-2898-34.4.123
29. Ganie ZA, Jugulam M, Jhala AJ. Temperature influences efficacy, absorption, and translocation of 2,4-D or glyphosate in glyphosate-resistant and glyphosate-susceptible common ragweed (*Ambrosia artemisiifolia*) and giant ragweed (*Ambrosia trifida*). Weed Sci. 2017 Sep;65(5):588-602. https://doi.org/10.1017/wsc.2017.32
30. Kumaratilake AR, Preston C. Low temperature reduces glufosinate activity and translocation in wild radish (*Raphanus raphanistrum*). Weed Sci. 2005 Feb;53(1):10-6. https://doi.org/10.1614/WS-03-140R

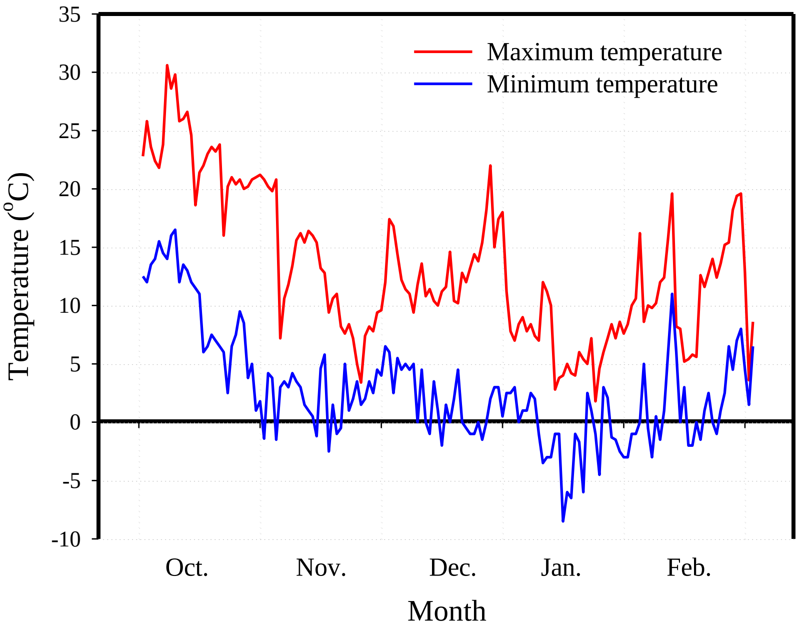
35
30
25
20
15
10
5
0
-5
-10
Temperature (^{o}C)
Maximum temperature
Minimum temperature
Oct.
Nov.
Dec.
Jan.
Feb.
Month

Fig 1.

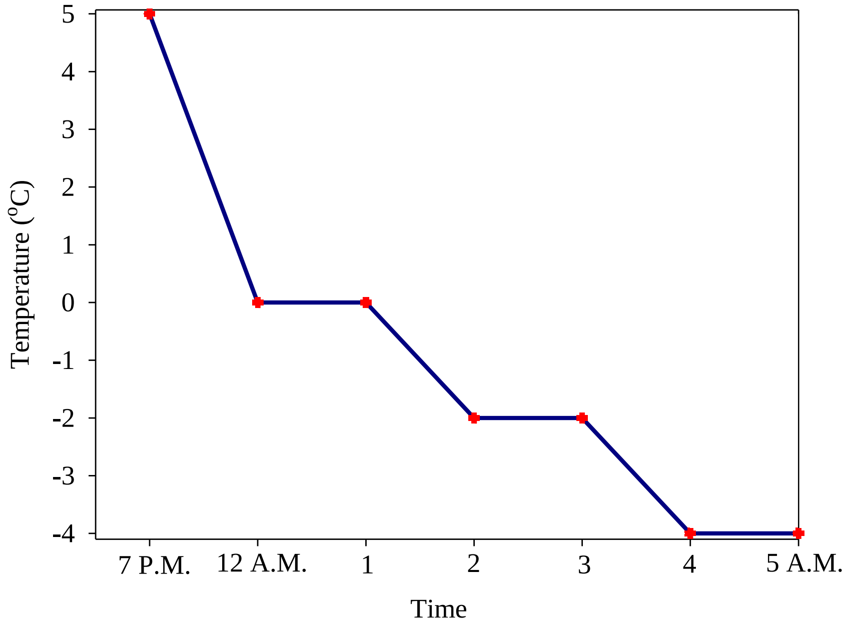
5
4
3
2
1
0
-1
-2
-3
-4
Temperature (^{o}C)
7 P.M.
12 A.M.
1
2
3
4
5 A.M.
Time

Fig. 2

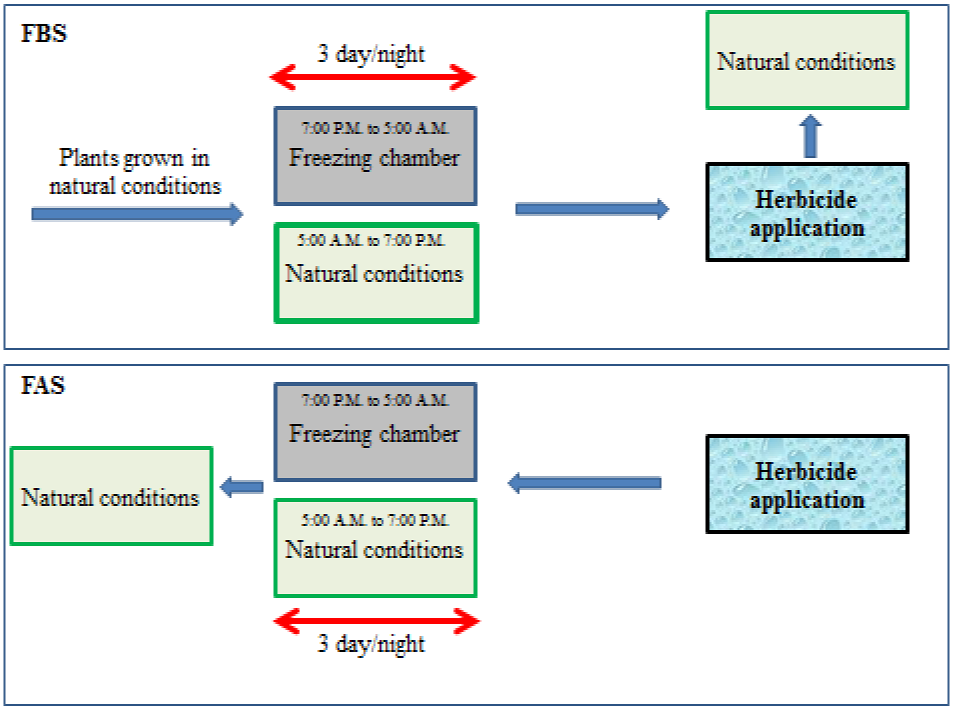
FBS
3 day/night
Natural conditions
7:00 P.M. to 5:00 A.M.
Freezing chamber
Plants grown in natural conditions
Herbicide application
5:00 A.M. to 7:00 P.M.
Natural conditions
FAS
7:00 P.M. to 5:00 A.M.
Freezing chamber
Natural conditions
Herbicide application
5:00 A.M. to 7:00 P.M.
Natural conditions
3 day/night

Fig. 3

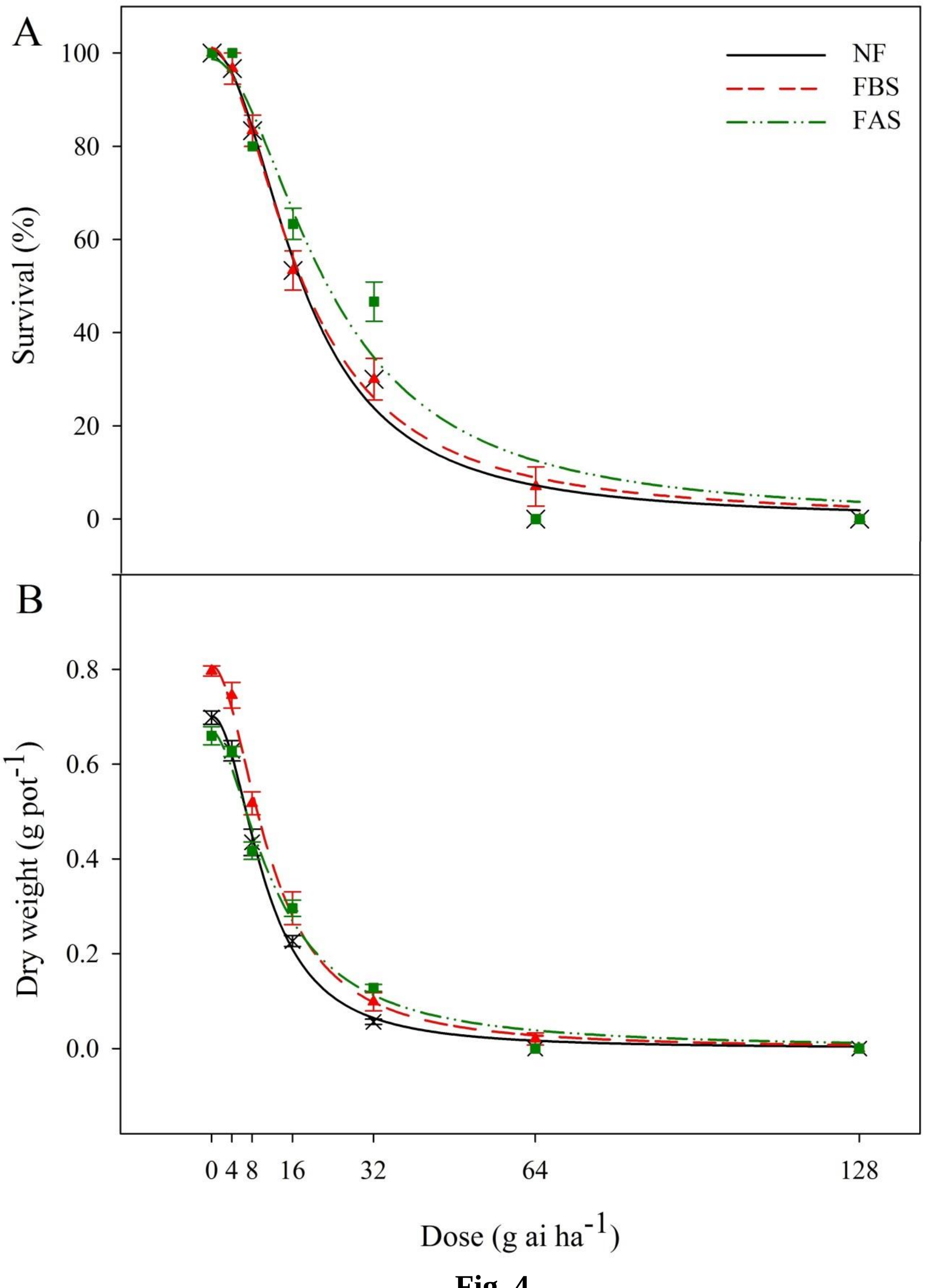
A
B
NF
FBS
FAS
Survival (%)
100
80
60
40
20
0
Dry weight (g pot^{-1})
0.8
0.6
0.4
0.2
0.0
0 4 8 16 32 64 128
Dose (g ai ha^{-1})

Fig. 4

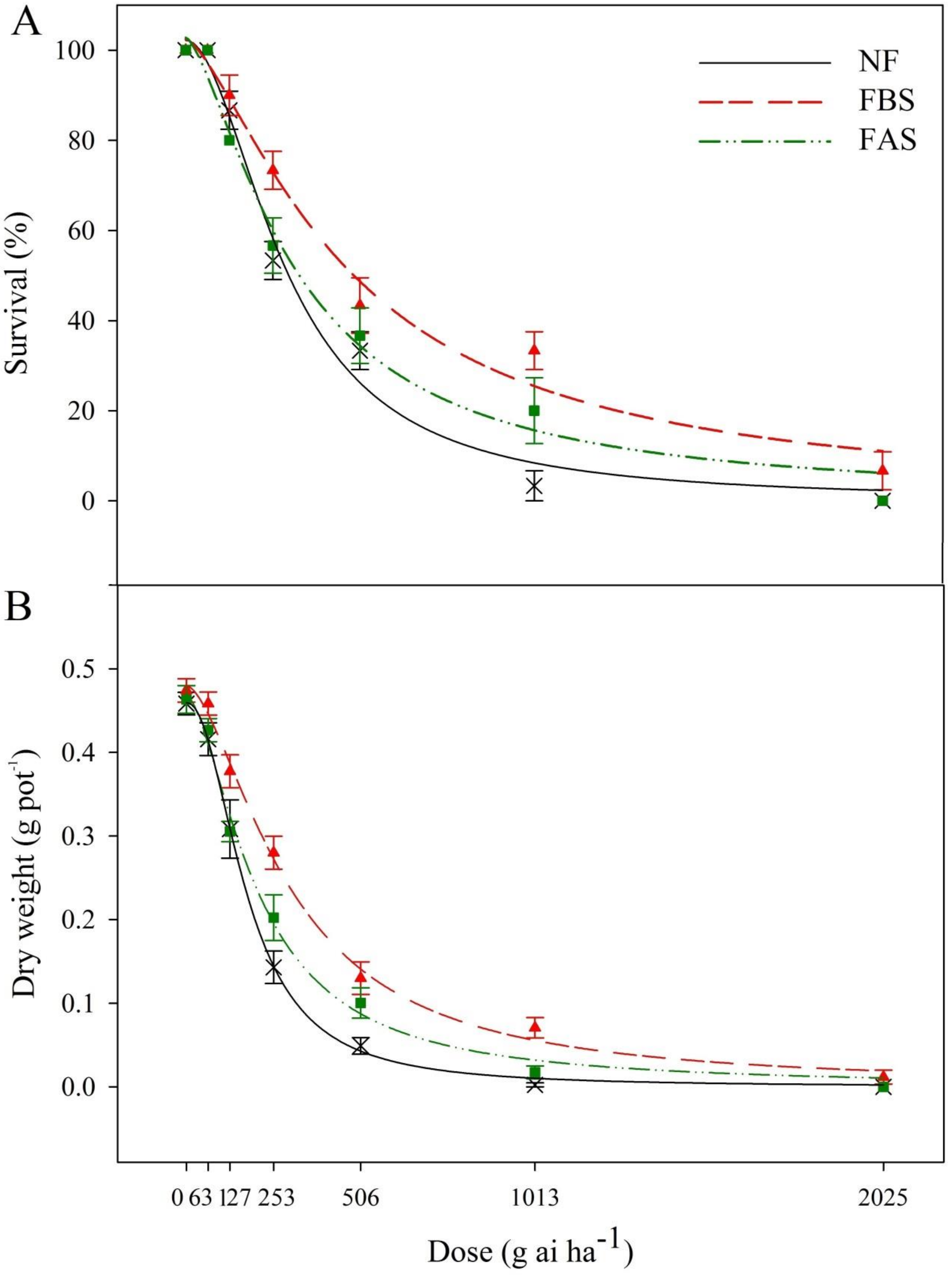

A
NF
FBS
FAS
Survival (%)
100
80
60
40
20
0
B
Dry weight (g pot^{-1})
0.5
0.4
0.3
0.2
0.1
0.0
0 63 127 253 506 1013 2025
Dose (g ai ha^{-1})

Fig. 5

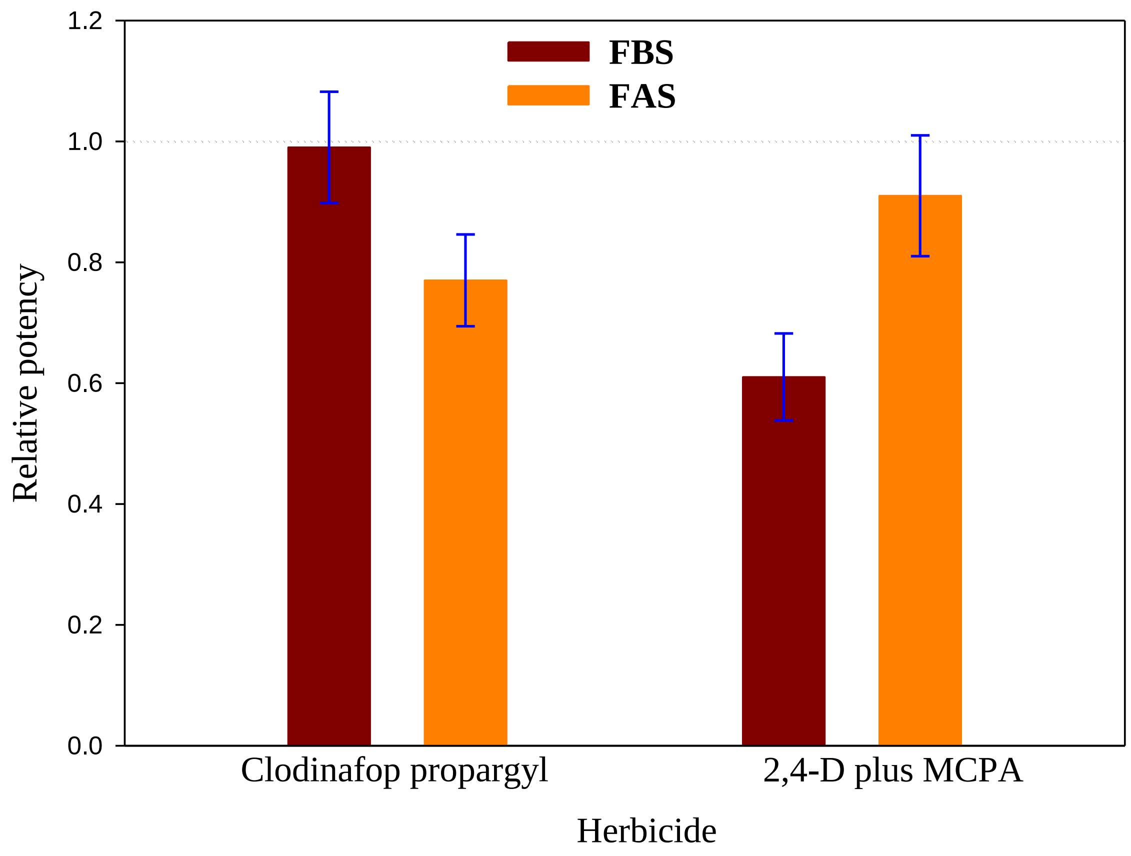
FBS
FAS
Relative potency
1.2
1.0
0.8
0.6
0.4
0.2
0.0
Clodinafop propargyl
2,4-D plus MCPA
Herbicide

Fig. 6

Click here to access/download
Supporting Information
DATA.xlsx

Freezing stress affects the efficacy of clodinafop-propargyl and 2,4-D plus MCPA on wild oat and turnipweed in wheat

Short running title: Freezing and herbicide efficacy

Alireza Hasanfard[1], Mehdi Rastgoo[1], Ebrahim Izadi Darbandi[1], Ahmad Nezami[1], Bhagirath Singh Chauhan[2]

[1] Department of Agrotechnology, Faculty of Agriculture, Ferdowsi University of Mashhad, Iran

[2] Queensland Alliance for Agriculture and Food Innovation (QAAFI) *and* School of Agriculture and Food Sciences (SAFS), The University of Queensland, Gatton, Queensland, Australia

***Corresponding author:** m.rastgoo@um.ac.ir

Abstract

The occurrence of freezing stress around herbicides application is one of the most important factors influencing their performance. This experiment was performed to evaluate the efficacy of clodinafop-propargyl and 2,4-D plus MCPA, the most important herbicides used in wheat fields in Iran, under the influence of a freezing treatment (-4°C). Wheat and its two common weeds, winter wild oat and turnipweed, were exposed to the freezing treatment for three nights from 7:00 P.M. to 5:00 A.M. before and after herbicide application, and their response was compared with plants that did not grow under freezing stress. Under no freezing (NF) and freezing after spray (FAS) conditions, winter wild oat was completely controlled with the recommended dose of clodinafop-propargyl (64 g ai ha^{-1}). However, the survival percentage of winter wild oat in the freezing before spray (FBS) of clodinafop-propargyl 64 g ha^{-1} was 7%, and it was completely controlled with twice the recommended dose (128 g ha^{-1}). Under NF conditions and FAS treatment, turnipweed was completely controlled with twice the recommended dose of 2,4-D plus MCPA (2025 g ha^{-1}). However, in the FBS treatment, the survival of turnipweed was 7% (2025 g ha^{-1}). The LD_{50} (dose required to control 50% of individuals in the population) and GR_{50} (dose causing 50% growth reduction of plants) rankings were NF<FBS<FAS for clodinafop-propargyl and NF<FAS<FBS for 2,4-D plus MCPA. Selectivity index for clodinafop-propargyl in NF conditions, FBS, and FAS treatments was 2.4, 0.91, and 0.78, respectively, and, for 2,4-D plus MCPA, it was 2.6, 0.12, and 0.88, respectively. According to the results of this experiment, it can be stated that the freezing stress after the spraying of clodinafop-propargyl and the freezing stress before a spray of 2,4-D plus MCPA would further reduce the efficacy of these herbicides.

Keywords: GR_{50}, Herbicide efficacy, LD_{50}, Selectivity index

Introduction

Winter wild oat (*Avena ludoviciana* Durieu) and turnipweed [*Rapistrum rugosum* (L.) All.] are the most important annual weeds of winter wheat (*Triticum aestivum* L.) in Iran, which cause significant reductions in wheat yield [1, 2]. The high reproduction potential and fitness to freezing stress, which results in a wide dispersal of weed seeds are the major challenges in the management of winter annual weeds like wild oat and turnipweed. Therefore, in cold regions of Iran, the dispersal and geographical distribution of these weeds are more significant than other weed species [3].

One of the most important consequences of climate change is the change in glaciers' occurrence patterns [4]. A sharp drop in temperature after high temperatures leads to crop damage. Increasing autumn temperatures in Iran, resulting from climate change, have led to reduced freezing tolerance of autumn crops [3]. In other words, autumn crops will not tolerate freezing stress due to the lack of hardening process in autumn. As a result, they will be damaged by decreasing temperatures during winter, and their yield will be reduced [3, 4]. It has been reported that increasing autumn temperatures resulting from climate change in western Canada led to reduced freezing tolerance of winter cereals [5]. In other words, due to the lack of optimal cold acclimation in autumn, crops will not be able to tolerate freezing stress. As a result, they will be damaged by decreasing temperatures during winter or by the occurrence of late spring frosts [6]. However, weeds generally have a higher ability to tolerate freezing stress [7]. Based on this, it can be inferred that climate change is adversely changing agricultural production and can significantly change the management of current cropping systems that have evolved under optimum climatic conditions.

Hand weeding and hoeing are not common in wheat fields, and mechanical weed management methods are not effective, so chemical methods are mainly used to control weeds in

the wheat fields in Iran. Clodinafop-propargyl and 2,4-D plus MCPA are the most common herbicides used for controlling weeds in wheat fields in Iran. Due to the particular importance of this crop in food security, their wide excessive applications can be justified [8, 9]. Clodinafop-propargyl is a systemic and selective herbicide of the aryloxyphenoxypropionate (AOPP) group and used post-emergent to control annual grass weeds in wheat. This herbicide inhibits the activity of acetyl-coenzyme A carboxylase (ACCase) and thus disrupts the biosynthesis of fatty acids [10]. 2,4-D plus MCPA, synthetic auxin herbicides, is a systemic herbicide mixture registered for selective post-emergence control of annual broadleaf weeds in wheat [11].

The efficacy of herbicides is significantly affected by environmental conditions before, during, and after application [12]. It has been reported that environmental factors such as light intensity, temperature, water stress, relative humidity, and nutrient availability alter the herbicide efficacy [13, 14]. On the other hand, plant species and their growth stages also affect herbicide efficacy [15]. Numerous studies indicate that ambient temperature is one of the most influential factors affecting the performance of herbicides [14, 16, 17]. Increasing the temperature is likely to increase the absorption and translocation of some herbicides and increase their performance. In contrast, the improvement in herbicide performance at low temperatures may be due to a reduction in the rate of herbicide metabolism [17, 18]. In cold regions, freezing temperatures occur around the time of herbicide application. Therefore, the occurrence of severe freezing that causes damage to plant leaves will have adverse effects on the activity and efficacy of foliar-applied herbicides [19]. On the other hand, application of herbicides in unfavorable environmental conditions (such as adverse temperature before, during, and after herbicide application) causes stress in the crop, and this changes the selectivity index of the herbicide, which will ultimately cause damage to the

crop [20]. The selectivity index refers to the potential of a herbicide to eliminate weeds in a crop without affecting the yield or quality of the final product [21].

At the appropriate growth stage of weeds for herbicide application, the temperature usually decreases, and this issue leads to a decrease in the performance of herbicides. Due to the lack of information on the efficacy of herbicides under freezing stress, this experiment was conducted to evaluate the efficacy of clodinafop-propargyl and 2,4-D plus MCPA on wild oat and turnipweed under freezing stress before and after herbicide application.

Materials and methods

Experimental site and plant material

Seeds of wheat (cv. Mihan) were harvested from Khorasan Razavi Agricultural and Natural Resources Research and Education Center, and seeds of winter wild oat and turnipweed were collected from infested farms around Mashhad-Iran in May 2019. Experiments were conducted at the Faculty of Agriculture, the Ferdowsi University of Mashhad, Iran (Lat 36° 15′ N, Long 59° 28 E; 985 m Altitude). To break the dormancy of winter wild oat and turnipweed, their seeds were exposed to moist-chilling treatment and potassium nitrate (KNO_3) 0.2% solution for 3 days at 5℃ in the dark, respectively [3]. The seeds were placed in 9-cm diameter Petri dishes (20-25 seeds per Petri dish) on moist filter papers and kept at 20℃. After 5 days, the germination percentage of winter wild oat and turnipweed seeds was >90%. The experiment was repeated twice with three replicates. To experience the same cold acclimation, the plants were planted at approximately the same time on October 21 (first run) and 24 (second run) in 2019. Plastic pots (12 cm in diameter and 20 cm in height) were used in the trial, and pots were filled with a mixture of sand, agricultural soil, and peat moss (2:2:1 sand, agricultural soil, and peat moss, respectively). For uniform

emergence, turnipweed seeds were planted in a seedling tray and transferred to pots in the cotyledon stage. Eight germinated seeds of wild oat were planted at 4-5 cm depth below the soil surface. To induce cold acclimation, the plants were kept outdoors, grown in natural conditions (Fig. 1), and irrigated according to daily requirements until they were tested.

Fig 1. Minimum and maximum daily air temperatures at the experimental site from October 2019 to February 2020. Relative humidity of 40-50% under the natural photoperiod [3].

Experimental factors

Experimental factors included freezing stress at three conditions, including no freezing (NF), freezing before spray (FBS), and freezing after spray (FAS). Plant species included wheat, winter wild oat, and turnipweed. Herbicides were applied at 0, 6.25, 12.5, 25, 50, 100, and 200% of the recommended dose for the herbicide clodinafop-propargyl (0.8 L ha^{-1}; 8% EC) and 2,4-D plus MCPA (1.5 L ha^{-1}; 67.5% SL).

Freezing treatment and herbicide application

Under NF, the plants grew completely in natural winter conditions without exposing to freezing stress. For FBS, the plants grew up to the 3-4 leaf stage under natural conditions. They were exposed to freezing from 7:00 P.M. to 5:00 A.M. for three nights before herbicide application [19]. Freezing treatment was performed using a thermogradient freezer (Weiss Technik). The initial temperature of the freezer was 5°C; however, the freezer was cooled down at a rate of 2°C per hour to reach a temperature of -4°C. The temperature drop slope was adjusted based on the long-term meteorological statistics of Mashhad in the time range of herbicide application. The pots were kept at -4°C for 1 h and then removed from the freezer (Fig. 2).

Fig. 2 Trend of temperature decrease in thermogradient freezer during the night from 7:00 P.M. to 5:00 A.M.

Each morning at 5:30 A.M. the plants were transferred out of the freezer, kept in natural conditions until 6:30 P.M., and then returned to the freezer during the night. After three nights of freezing exposure, the pots were taken out of the freezer and sprayed with herbicides after 2 h [19]. After spraying, the plants were kept outside under natural conditions for the duration of the experiment.

In the FAS treatment, freezing stress was applied for three nights by placing the samples treated with herbicides in the thermogradient freezer. For this purpose, the plants were taken out of the freezer at 5:30 A.M., transferred to natural conditions, and then returned to the freezer at 6:30 P.M. for the overnight freezing treatment (Similar to FBS treatment). Three nights after freezing treatment, the plants were transferred to natural conditions to continue the trial process (Fig. 3).

Fig. 3 Schematic diagram of the experimental study design. Abbreviation: FBS (freezing before spray); FAS (freezing after spray).

The conditions inside the thermogradient freezer were the same for both freezing treatments before and after spray. The experiment was performed in the freezer in completely dark conditions and at the same relative humidity (35±5%) for all treatments. Temperature changes in the freezer were regularly monitored when the samples were in the freezer. For all the freezing treatments, herbicides were applied at the same time. Plants were transferred to natural conditions after three nights of freezing treatment at 5:30 A.M. Also, plants related to freezing after spray were transferred to the freezer at 6:30 P.M. On the same day, plants that were not exposed to freezing were sprayed from 7:30 A.M. to 10.30 A.M. The herbicides were applied by a researcher-made

moving boom sprayer equipped with a TeeJet 8002 flat fan nozzle (spray output volume of 200 L ha^{-1} at a pressure of 260 kPa). Plants were returned and maintained outdoor under natural conditions of winter after the treatment.

Measurement of traits

Four weeks after herbicide treatments, survival percentage (Eq. 1) and shoot dry weight were measured by placing the samples in an oven at 75°C for 48 h. Plants that produced new leaves were considered alive and those that experienced severe chlorosis and stunted growth were recorded as dead [22].

$$Survival\ \% = \frac{N1}{N0} \times 100 \qquad \text{(Eq. 1)}$$

Where, N_1 and N_0 are the numbers of alive plants four weeks after herbicide treatment and the number of alive plants before herbicide treatment, respectively.

Dose–response experiments

The response of weed traits to the doses of herbicides tested was analyzed by nonlinear regression. For this purpose, the data were fitted to a three-parameter log-logistic equation (Eq. 2) [23].

$$Y = \frac{d}{1+e^{b(\log x - loge)}} \qquad \text{(Eq. 2)}$$

Where, Y is the response rate (survival % or dry weight) at dose x, d is the upper limit for Y, b is the slope of the curve around e, and e is the effective dose (g ai ha^{-1}). Here, e is replaceable with LD_{50} (dose required to control 50% of individuals in the population) or GR_{50} (dose causing 50% dry weight reduction of plants).

Relative potency

Relative potency was used to determine herbicide efficacy under freezing stress and no freezing stress (Eq. 3) [24].

$$Relative\ potency = \frac{ED_{50A}}{ED_{50B}} \qquad \text{(Eq. 3)}$$

Where, ED_{50A} is the dose required to reduce survival by 50% in NF, and ED_{50B} is the dose needed to reduce survival by 50% in FBS or FAS. Relative potency = 1 indicates that freezing treatment (FBS and FAS) did not affect herbicide efficacy. If relative potency > 1, it indicates that the herbicides are more efficient under freezing stress. Relative potency < 1 indicates the decreased effectiveness of the herbicides under freezing stress with respect to optimum conditions.

Selectivity index

The ratio between doses that caused 10% damage to crops and 90% damage to weeds was considered a selectivity index [24] and calculated using Eq. 4.

$$Selectivity\ index\ (10{,}90) = \frac{ED_{10\ (crop)}}{ED_{90\ (weed)}} \qquad \text{(Eq. 4)}$$

Where, ED_{90} is 90% of the herbicide effect on weeds survival (herbicide dose to kill 90% of the population), and ED_{10} is 10% of the herbicide impact on crop survival (a 10% effect on the crop). The higher the SI, the more selective the herbicide in the crop [24].

Statistical analysis

The experiments were conducted using a completely randomized design in factorial arrangement with three replications. Afterward, differences between treatments were compared with the standard error of mean (SEM) values. For data analysis, analysis of variance (ANOVA) was performed using the SAS software (v. 9.4, SAS Institute Inc, Cary, NC, USA). Data for both runs

were pooled as no significant differences were observed between runs ($P > 0.05$). Homogeneity of variance was confirmed using Levene's test. Analysis of biometric data was performed by the R program (v. 4.0.4) [25]. Dose-response curves were also fitted with the asymmetrical sigmoid form. LD_{50} and GR_{50} were separated using 95% confidence intervals (CI).

Results

Clodinafop-propargyl

Survival percentage and LD_{50}

The dose-response experiments were similar for NF and FAS treatments at the recommended dose (Figure 4A). Thus, in both conditions, winter wild oat was completely controlled with the recommended dose (64 g ha^{-1}). However, the survival of seedlings in the FBS treatment was 7%, and to achieve complete control, 2-fold the recommended dose (128 g ha^{-1}) was needed. The LD_{50} in NF, FBS, and FAS was 17.9, 18.1, and 23.2 g ha^{-1}, respectively (Table 1).

Fig. 4 Survival (A) and dry weight (B) of winter wild oat treated with various doses of clodinafop-propargyl under different freezing treatments. Abbreviation: NF (no freezing); FBS (freezing before spray): FAS (freezing after spray). The recommended dose of clodinafop-propargyl in Iran is 64 g ha^{-1}.

Table 1 The dose of clodinafop-propargyl and 2,4-D plus MCPA required for 50% mortality (LD_{50}) and the dose that caused 50% inhibition of growth noted by biomass (GR_{50}) of winter wild oat and turnipweed under different freezing treatments.

Herbicide	Freezing Treatment[a]	b[b]±SE[a]	d[c]±SE	LD_{50} ±SE	95% CI[e]	R^2
			%	g ai ha^{-1}		
Clodinafop-propargyl	NF	2.0 ± 0.19**[d]	100.3 ± 2.8**	17.9 ± 1.2**	15.6-20.2	0.99
	FBS	1.9 ± 0.17**	101.0 ± 2.9**	18.1 ± 1.2**	15.7-20.5	0.99
	FAS	1.9 ± 0.19**	98.7 ± 2.9**	23.2 ± 1.7**	19.8-26.6	0.96
2,4-D plus MCPA	NF	1.9 ± 0.20**	102.2 ± 3.2**	292.8 ± 21.9**	249.4-336.3	0.98
	FBS	1.5 ± 0.15**	102.4 ± 3.2**	472.8 ± 42.4**	388.8-556.8	0.98
	FAS	1.5 ± 0.14**	102.8 ± 3.5**	318.8 ± 28.9**	261.6-376.1	0.98
		b[b]±SE[a]	d[c]±SE	GR_{50} ±SE	95% CI[e]	
			g pot^{-1}	g ai ha^{-1}		
Clodinafop-propargyl	NF	2.1 ± 0.15**	0.70 ± 0.02**	10.6 ± 0.52**	9.6-11.6	0.99
	FBS	2.0 ± 0.12**	0.80 ± 0.02**	11.6 ± 0.51**	10.6-12.6	0.99
	FAS	1.7 ± 0.12**	0.67 ± 0.02**	12.8 ± 0.74**	11.3-14.3	0.98
2,4-D plus MCPA	NF	2.2 ± 0.23**	0.46 ± 0.01**	176.2 ± 11.3**	153.9-198.6	0.99
	FBS	1.7 ± 0.16**	0.48 ± 0.01**	298.6 ± 21.5**	256.0-341.3	0.99
	FAS	1.6 ± 0.15**	0.47 ± 0.01**	207.8 ± 15.9**	176.2-239.4	0.99

Abbreviation: NF (no freezing); FBS (freezing before spray); FAS (freezing after spray); SE: Standard Error. b is the slope of the curve around e (LD_{50} and GR_{50}); d is the upper limit for Y (survival % or dry weight); **: significant at $p \leq 0.01$. LD_{50} and GR_{50} were separated using their 95% confidence intervals (CI). The data shows the parameter's mean ± SE. n = 6

Dry weight and GR_{50}

The dry weight of winter wild oat in NF and FAS with the recommended dose (64 g ha^{-1}) and FBS with twice the recommended dose (128 g ha^{-1}) reached 0 g (Fig. 4B). The GR_{50} of plants not exposed to freezing stress was 10.6 g ha^{-1}. The GR_{50} was 11.6 and 12.8 g ha^{-1} in FBS and FAS, respectively (Table 1).

2,4-D plus MCPA

Survival percentage and LD_{50}

The dose-response experiments of 2,4-D plus MCPA (similar to clodinafop-propargyl) were the same in NF and FAS treatments (Fig. 5A). In both conditions, turnipweed was completely

controlled with twice the recommended dose (2025 g ha^{-1}). However, in the FBS treatment, the survival of turnipweed was 7%. At the recommended dose (1012.5 g ha^{-1}), the survival of turnipweed in NF, FBS, and FAS was 3%, 33%, and 20%, respectively. The LD_{50} in NF, FBS, and FAS was 293, 473, and 319 g ha^{-1}, respectively (Table 1).

Fig. 5 Survival (A) and dry weight (B) of turnipweed treated with various doses of 2,4-D plus MCPA under different freezing treatments. Abbreviation: NF (no freezing); FBS (freezing before spray): FAS (freezing after spray). The recommended dose of 2,4-D+MCPA in Iran is 1012.5 g ha^{-1}.

Dry weight and GR_{50}

The dry weight of turnipweed in NF and FAS with twice the recommended dose (2025 g ha^{-1}) of 2,4-D plus MCPA was reduced to 0 (Fig. 5 B). However, in the FBS treatment, the dry weight of turnipweed with twice the recommended dose was 0.012 g per pot. At the recommended dose, the dry weight of turnipweed in FBS was 97% and 76% more than NF and FAS, respectively. The GR_{50} of NF, FBS, and FAS were 176, 299, and 208 g ha^{-1}, respectively (Table 1).

Relative potency

The relative potency of clodinafop-propargyl and 2,4-D plus MCPA was significantly reduced in FBS and FAS compared to NF (Fig. 6). Thus, the relative potency in FBS and FAS of clodinafop-propargyl was 0.99 and 0.77, respectively, and at 2,4-D plus MCPA was 0.62 and 0.92, respectively.

Fig. 6 Relative potency of clodinafop-propargyl and 2,4-D plus MCPA under different freezing treatments. Abbreviation: FBS (freezing before spray) and FAS (freezing after spray). The error bars indicate standard error (SE). The dashed line represents the relative potency = 1.0.

Selectivity index

As shown in Table 2, selectivity index values for clodinafop-propargyl in NF, FBS, and FAS treatments were 2.4, 0.91, and 0.78, respectively, and, for 2,4-D plus MCPA, they were 2.6, 0.12, and 0.88, respectively. For clodinafop-propargyl, the LD_{10} of wheat in NF was 127.9 g ha^{-1}. However, LD_{10} of wheat in the FBS and FAS was 53.5 and 57.1 g ha^{-1}, respectively (Table 2). For 2,4-D plus MCPA, the LD_{10} of wheat in NF, FBS, and FAS was 2380, 264, and 1236 g ha^{-1}, respectively.

Table 2 Sensitivity of plant species of wheat, winter wild oat, and turnipweed to clodinafop-propargyl and 2,4-D plus MCPA under different freezing treatments.

Freezing Treatment	Plant species	Clodinafop-propargyl		2,4-D plus MCPA	
		LD_{10} (wheat) and LD_{90} (winter wild oat) (g ai ha^{-1}) ± SE	SI	LD_{10} (wheat) and LD_{90} (turnipweed) (g ai ha^{-1}) ± SE	SI
NF	Wheat	127.9 ± 8.8	-	2380 ± 455	-
	Weed	53.5 ± 5.4	2.4	908 ± 108	2.6
FBS	Wheat	53.5 ± 11.0	-	264 ± 61.0	-
	Weed	59.0 ± 6.4	0.91	2144 ± 327	0.12
FAS	Wheat	57.1 ± 8.7	-	1236 ± 163	-
	Weed	73.6 ± 7.2	0.78	1400 ± 199	0.88

Abbreviation: NF; No freezing, FBS: Freezing before spray, FAS: Freezing after spray SI: Selectivity index. The selectivity index (SI) was calculated as follows: *Selectivity index (10, 90):* $\frac{ED10\ (crop)}{ED90\ (weed)}$

Discussion

The required dose of clodinafop-propargyl for complete control of winter wild oat seedlings in FBS treatment was twice that of NF and FAS treatments. The trend of the reducing survival % in seedlings related to the FBS treatment and NF was similar. The LD_{50} for clodinafop-propargyl in freezing treatments shows that exposure of winter wild oat seedlings to freezing treatment for three nights before and after spray reduces the efficacy of this herbicide compared to NF. The LD_{50} was 22% and 23% higher in the FAS compared to the FBS and NF, respectively. Freezing treatment for three nights before and after spraying of clethodim herbicide (the mode of action similar to

clodinafop-propargyl) led to an increase in LD_{50} [19]. It decreased herbicide efficacy in rigid ryegrass populations (*Lolium rigidum* Gaud.) compared to NF.

The dose of clodinafop-propargyl required to reduce winter wild oat biomass to zero in the FBS treatment was twice that of the NF and FAS treatments. The GR_{50} is more pronounced in FBS and FAS compared to NF, indicating a decrease in the efficacy of clodinafop-propargyl. The GR_{50} was 17% and 8% higher in the FAS treatment compared to NF and FBS, respectively. In other words, the occurrence of three nights of freezing stress after herbicide application reduced the performance of clodinafop-propargyl more than other treatments. It has been reported that wild oat plants (*Avena fatua*) that grew at low temperatures were less sensitive to difenzoquat than plants that grew at high temperatures [26].

Our results clearly show that the occurrence of freezing stress around spray reduces the efficacy of clodinafop-propargyl, especially in the treatment of FAS. Herbicide translocation in the phloem in freezing conditions could decline due to damage to the phloem cells, which may lead to a decrease in the herbicide efficacy. Decreased efficacy of sethoxydim at low temperatures due to reduced herbicide translocation for Quackgrass [*Elymus repens* (L.) Gould] control was reported by Coupland [27]. In general, to maintain the herbicide efficacy under cold conditions, using clodinafop-propargyl after the freezing stress can be recommended in areas with a similar climate.

Although the three-night treatment of FBS and FAS resulted in a reduction in turnipweed control by 2,4-D plus MCPA compared to NF, this reduction was different in the two freezing treatments. At the recommended dose (1013 g ha^{-1}) in NF, almost all turnipweed plants were controlled, while the survival percentage of turnipweed in FBS and FAS treatments were 33% and 20%, respectively. Low temperatures around the time of herbicide application reduced the

effectiveness of systemic herbicides [28]. Based on LD_{50} values, the efficacy of 2,4-D plus MCPA under FBS and FAS was 1.6 and 1.1 times lower compared to NF.

FAS is possible to damage the phloem cells, which reduces the translocation of the herbicide. The efficacy of 2,4-D plus MCPA in the treatment of FBS was reduced. The decrease in the effect of 2,4-D plus MCPA in the treatment of FBS is probably due to the reduction of absorption and translocation of this herbicide in turnipweed seedlings. In this regard, an increase in efficacy of 2,4-D and glyphosate at high temperatures compared to low temperatures in common ragweed (*Ambrosia artemisiifolia* L.) and giant ragweed (*Ambrosia trifida* L.) due to the increased absorption and/or translocation at high temperatures around herbicide application has been reported by Ganie et al. [29]. It has been also reported that low temperatures reduced glufosinate activity and translocation in wild radish (*Raphanus raphanistrum* L.) [30].

Turnipweed's dry weight at the recommended dose of 2,4-D plus MCPA (1012.5 g ha^{-1}) in FBS and FAS treatments was 35.5 and 8.5 times higher than the NF, respectively. Also, GR_{50} in FBS and FAS was 41% and 15.2% higher compared to NF, respectively. Although a decrease in herbicide efficacy is evident in both FBS and FAS treatments, high GR_{50} in FBS shows an adverse effect of three nights of freezing before the application of 2,4-D plus MCPA. In other words, the efficacy of this herbicide reacts more to FBS than to FAS. In a similar study, the GR_{50} for 2,4-D increased at low temperature in common ragweed compared to high temperature [29].

The freezing stress around 2,4-D plus MCPA application reduced the efficacy of this herbicide in FBS and FAS treatments compared to NF. The decrease in the efficacy of this herbicide in the treatment of FBS is probably due to the reduction of absorption and translocation of the herbicide in the phloem during freezing. Absorption and translocation of 2,4-D plus MCPA

might be reduced at low temperatures due to the effect on herbicide penetration facilitated by physicochemical factors, including the decreased rate of diffusion, increased viscosity of the cuticle, and physiological factors comprising decreases in photosynthesis, phloem translocation, and protoplasmic streaming [29]. In Iranian wheat fields, using the 2,4-D plus MCPA before the freezing stress can be recommended to maintain the herbicide efficacy under cold conditions.

When relative potency > 1, the herbicide efficacy is higher than during normal conditions, and when relative potency < 1, the herbicide efficacy is lower than usual [24]. The relative potency values of clodinafop-propargyl and 2,4-D plus MCPA were lower than one under both FBS and FAS treatments in the present experiment. In other words, freezing stress reduced the efficacy of these herbicides. Based on the results of relative potency, the reduction of clodinafop-propargyl efficacy in the FAS treatment (33%) was more significant than in the FBS treatment (1%). Conversely, for 2,4-D plus MCPA, the reduction in efficacy was more significant in the FBS treatment (38%) compared to the FAS treatment (8%).

The selectivity index for clodinafop-propargyl and 2,4-D plus MCPA was > 1 in NF, indicating that both herbicides acted selectively in wheat. However, applying three nights of FBS and FAS of both herbicides reduced the selectivity index to less than one. In other words, freezing stress reduced the selectivity index of both herbicides, which led to phytotoxicity in wheat. For clodinafop-propargyl, LD_{10} of wheat in NF was 58% and 55% higher than FBS and FAS, respectively. The LD_{10} values of 2,4-D plus MCPA in NF were 89% and 48% higher compared to FBS and FAS, respectively. In other words, the dose required to kill 10% of wheat in both herbicides under NF was higher than FBS and FAS. Therefore, FBS and FAS increased the sensitivity of wheat to herbicide application. Freezing stress may reduce the rate of herbicide metabolism in the crop and ultimately increase its susceptibility [17].

Conclusions

The relative potency results for both herbicides were used to confirm the results of LD_{50} and GR_{50}. Thus, LD_{50} and GR_{50} were higher for clodinafop-propargyl in the FAS treatment than in NF and FBS treatments. The LD_{50} and GR_{50} were higher for the 2,4-D plus MCPA in the FBS treatment than in the NF and FAS treatments. In other words, clodinafop-propargyl was more effective than other conditions in the treatment of three nights of FAS and 2,4-D plus MCPA in the treatment of three nights of FBS. The selectivity index for clodinafop-propargyl was lower in the FAS treatment (0.78) and for 2,4-D plus MCPA was lower in the FBS treatment (0.12). The efficacy of both herbicides was affected by the FBS and FAS treatments. The efficacy of clodinafop-propargyl in the FAS treatment and the efficacy of 2,4-D plus MCPA in the FBS treatment were reduced. The values of the selectivity index of herbicides in the conditions of freezing stress showed that this index could be well used to determine the selective performance of these herbicides. Overall, according to the results of this experiment, it can be stated that FAS with ACCase inhibitor herbicides, such as clodinafop-propargyl, may reduce their effectiveness in controlling narrow leaf weeds such as winter wild oat, and FBS with auxin type herbicides, such as 2,4-D plus MCPA, reduce their efficacy in controlling broadleaf weeds such as turnipweed. FBS for clodinafop-propargyl and FAS for 2,4-D plus MCPA had a slight adverse effect on the herbicide efficacy and selectivity index. Therefore, the use of these herbicides in the mentioned conditions (FBS for clodinafop-propargyl and FAS for 2,4-D plus MCPA) is recommended. In other words, to maintain high herbicide efficacy under cold (freezing) conditions, the use of clodinafop-propargyl after the freezing stress and 2,4-D plus MCPA before the freezing stress can be recommended in areas with a similar climate to Mashhad. Finally, the use of adjuvants, alternate herbicides, and various

herbicide formulations to control grass and broadleaf weeds in Iranian wheat production is suggested to prevent the adverse effects of freezing stress on herbicides.

Conflicts of interest

The authors declare that they have no conflict of interest.

Acknowledgment

We are grateful to Ferdowsi University of Mashhad, Iran, for providing financial support for present research (Project No.3.49759). No additional external funding was received for this study.

References

1. Hatami ZM, Gherekhloo J, Rojano-Delgado AM, Osuna MD, Alcántara R, Fernández P, et al. Multiple mechanisms increase levels of resistance in *Rapistrum rugosum* to ALS herbicides. Front Plant Sci. 2016 Feb 22;7:169. https://doi.org/10.3389/fpls.2016.00169
2. Nosratti I, Sabeti P, Chaghamirzaee G, Heidari H. Weed problems, challenges, and opportunities in Iran. Crop Prot. 2020 Aug 1;134:104371. https://doi.org/10.1016/j.cropro.2017.10.007
3. Hasanfard A, Rastgoo M, Darbandi EI, Nezami A, Chauhan BS. Regeneration capacity after exposure to freezing in wild oat (*Avena ludoviciana* Durieu.) and turnipweed (*Rapistrum rugosum* (L.) All.) in comparison with winter wheat. Environ. Exp. Bot. 2021 Jan 1;181:104271. https://doi.org/10.1016/j.envexpbot.2020.104271
4. Sierra-Almeida A, Cavieres LA, Bravo LA. Warmer temperatures affect the in situ freezing resistance of the Antarctic vascular plants. Front. Plant Sci. 2018 Oct 8;9:1456. https://doi.org/10.3389/fpls.2018.01456
5. Willick IR, Tanino KK, Gusta LV. The impact of global climate change on the freezing tolerance of winter cereals in Western Canada. J. Agron. Crop Sci. 2021 Feb;207(1):88-99. https://doi.org/10.1111/jac.12447
6. Vyse K, Pagter M, Zuther E, Hincha DK. Deacclimation after cold acclimation—a crucial, but widely neglected part of plant winter survival. J. Exp. Bot. 2019 Sep 15;70(18):4595-604. https://doi.org/10.1093/jxb/erz229
7. Cici SZ, Van Acker RC. Relative freezing tolerance of facultative winter annual weeds. Can. J. Plant Sci. 2011;91(4):759-63. https://doi.org/10.4141/cjps2010-001
8. Baghestani MA, Zand E, Soufizadeh S, Beheshtian M, Haghighi A, Barjasteh A, Birgani DG, Deihimfard R. Study on the efficacy of weed control in wheat (*Triticum aestivum* L.) with tank mixtures of grass herbicides with broadleaved herbicides. Crop Prot. 2008 Jan 1;27(1):104-11. https://doi.org/10.1016/j.cropro.2007.04.013
9. Enghiad A, Ufer D, Countryman AM, Thilmany DD. An overview of global wheat market fundamentals in an era of climate concerns. Int J Agron. 2017 Jul 2;2017. https://doi.org/10.1155/2017/3931897
10. Bajwa AA, Akhter MJ, Iqbal N, Peerzada AM, Hanif Z, Manalil S, Hashim S, Ali HH, Kebaso L, Frimpong D, Namubiru H. Biology and management of Avena fatua and Avena

ludoviciana: two noxious weed species of agro-ecosystems. Environ Sci Pollut Res. 2017 Aug;24(24):19465-79. https://doi.org/10.1007/s11356-017-9810-y

11. Peterson MA, McMaster SA, Riechers DE, Skelton J, Stahlman PW. 2, 4-D past, present, and future: a review. Weed Technol. 2016 Jun;30(2):303-45. https://doi.org/10.1614/WT-D-15-00131.1
12. Collings LV, Blair AM, Gay AP, Dyer CJ, Mackay N. The effect of weather factors on the performance of herbicides to control *Alopecurus myosuroides* in winter wheat. Weed Res. 2003 Apr;43(2):146-53. https://doi.org/10.1046/j.1365-3180.2003.00327.x
13. Rocha-Pereira MR, Klar AE, Martins D, Ferreira de Souza GS, Villalba J. Efecto del estrés hídrico sobre la eficacia de los herbicidas aplicados en *Urochloa decumbens*. Cienc. Investig Agrar. 2012 Apr;39(1):211-20. http://dx.doi.org/10.4067/S0718-16202012000100018
14. Johnson BC, Young BG. Influence of temperature and relative humidity on the foliar activity of mesotrione. Weed Sci. 2002 Apr;50(2):157-61. https://doi.org/10.1614/0043-1745(2002)050[0157:IOTARH]2.0.CO;2.
15. Chahal PS, Aulakh JS, Rosenbaum K, Jhala AJ. Growth stage affects dose response of selected glyphosate-resistant weeds to premix of 2,4-D choline and glyphosate (Enlist Duo (TM) herbicide*). J. Agric. Sci. 2015 Nov 1;7(11):1. https://doi.org/10.5539/jas.v7n11p1
16. Waltz AL, Martin AR, Roeth FW, Lindquist JL. Glyphosate efficacy on velvetleaf varies with application time of day. Weed Technol. 2004 Dec;18(4):931-9. https://doi.org/10.1614/WT-03-123R3
17. Godar AS, Varanasi VK, Nakka S, Prasad PV, Thompson CR, Mithila J. Physiological and molecular mechanisms of differential sensitivity of Palmer amaranth (*Amaranthus palmeri*) to mesotrione at varying growth temperatures. Plos One. 2015 May 19;10(5):e0126731. https://doi.org/10.1371/journal.pone.0126731
18. Pline WA, Wu J, Hatzios KK. Effects of temperature and chemical additives on the response of transgenic herbicide-resistant soybeans to glufosinate and glyphosate applications. Pestic. Biochem. Phys. 1999 Oct 1;65(2):119-31. https://doi.org/10.1006/pest.1999.2437
19. Saini RK, Malone J, Preston C, Gill GS. Frost reduces clethodim efficacy in clethodim-resistant rigid ryegrass (*Lolium rigidum*) populations. Weed Sci. 2016 Jun;64(2):207-15. https://doi.org/10.1614/WS-D-15-00140.1
20. Moseley C, Hatzios KK, Hagood ES. Uptake, translocation, and metabolism of chlorimuron in soybean (*Glycine max*) and morningglory (*Ipomoea* spp.). Weed Tech. 1993 Jun;7(2):343-8. https://doi.org/10.1017/S0890037X00027706
21. Negrisoli E, Velini ED, Tofoli GR, Cavenaghi AL, Martins D, Morelli JL, Costa AG. Selectivity of pre-emergence herbicides to sugarcane treated with nematicides. Planta Daninha. 2004 Dec;22(4):567-75.
22. Powles SB, Lorraine-Colwill DF, Dellow JJ, Preston C. Evolved resistance to glyphosate in rigid ryegrass (*Lolium rigidum*) in Australia. Weed Sci. 1998 Oct;46(5):604-7.
23. Ritz C, Baty F, Streibig JC, Gerhard D. Dose-response analysis using R. Plos One. 2015 Dec 30;10(12):e0146021. https://doi.org/10.1371/journal.pone.0146021

24. Tind T, Mathiesen TJ, Jensen JE, Ritz C, Streibig JC. Using a selectivity index to evaluate logarithmic spraying in grass seed crops. Pest Manag Sci: formerly Pesticide Science. 2009 Nov;65(11):1257-62. https://doi.org/10.1002/ps.1819
25. Team RC. R Foundation for Statistical Computing, Vienna, available at: www. R-project. org. 2018.
26. Kowalczyk B, Caseley JC, McCready CC. The effect of pre-treatment temperature on some physiological responses of *Avena fatua* to difenzoquat and benzoylpropethyl. Asp Appl Biol. 1983;4:235-44.
27. Coupland D. Influence of environmental factors on the performance of sethoxydim against *Elymus repens* (L.). Weed Res. 1987 Oct;27(5):329-36. https://doi.org/10.1111/j.1365-3180.1987.tb00770.x
28. Derr J, Serensits T. Impact of application temperature on broadleaf herbicide efficacy. J Environ Hortic. 2016 Dec;34(4):123-30. https://doi.org/10.24266/0738-2898-34.4.123
29. Ganie ZA, Jugulam M, Jhala AJ. Temperature influences efficacy, absorption, and translocation of 2,4-D or glyphosate in glyphosate-resistant and glyphosate-susceptible common ragweed (*Ambrosia artemisiifolia*) and giant ragweed (*Ambrosia trifida*). Weed Sci. 2017 Sep;65(5):588-602. https://doi.org/10.1017/wsc.2017.32
30. Kumaratilake AR, Preston C. Low temperature reduces glufosinate activity and translocation in wild radish (*Raphanus raphanistrum*). Weed Sci. 2005 Feb;53(1):10-6. https://doi.org/10.1614/WS-03-140R

Manuscript Number: **PONE-D-21-33766**

Manuscript title: **Freezing stress affects the efficacy of clodinafop-propargyl and 2,4-D plus MCPA on wild oat and turnipweed in wheat**

Dear Editor

Thank you very much for arranging the review of our manuscript. We greatly appreciate the reviewers for their complimentary comments and suggestions. We hope that you find our responses satisfactory and that the manuscript is now acceptable.

Sincerely,

Mehdi Rastgoo
Associate professor,
Department of Agrotechnology, Faculty of Agriculture, Ferdowsi University of Mashhad, Iran
Corresponding author

Response to the comments of the reviewer #1

We would like to thank the reviewer for the careful and thorough reading of this manuscript and the thoughtful comments and constructive suggestions, which help improve the manuscript quality. As indicated below, we have checked all the general and specific comments provided by the referees and have made necessary changes according to their indications, which are highlighted in yellow.

Reviewer #1: Unpredicted variability in temperature is associated with frequent extreme low-temperature events. And it is threatening global food security. Due to the lack of information on the efficacy of herbicides under freezing stress, this research was conducted to evaluate the efficacy of clodinafop-propargyl and 2,4-D plus MCPA on wild oat and turnipweed under freezing stress before and after herbicide application. The research results have guiding significance for production, but the article still needs to be revised and improved.

（1）The introduction part should increase the impact of global climate change on the occurrence of low temperature adversity events. And its impact on wheat production.

- The requested information has been added to the text.

（2）Experimental site and plant material part：Explain the amount of fertilizer used and the ratio of N, P, K.

- We did not use fertilizer in this experiment.

（3）The source of the meteorological data in Figure 1 should be clearly explained.

- The requested information has been added to the text.

（4）Results part: The description of the results needs to be more in-depth. For example, how much does FBS significantly increase or decrease compared to NF?

- We respect the reviewer's opinion, but according to the results of the clodinafop-propargyl, we have written the requested information in another expression (gray highlight), and its re-expression is repetitive. But in the case of 2,4-D plus MCPA, the results were explained in more detail, and the requested information was added to the text (yellow highlight).

（5）The discussion part is not deep enough, some physiological discussions should be added.

- The requested information has been added to the text.

（6）The description in the conclusion part needs to be refined to increase readability.

- The requested information was added to the text. Clear text added.

（7）There are grammatical and editorial issues somewhere in this paper. Many of them could cause difficulties to the readers.

- We respect the reviewer's opinion, but a Native English-speaker colleague has edited the manuscript's text. We rechecked the text.

（8）The article does not have data on wheat yield. It would be very good if we could increase the data on the effects of herbicides before and after low temperature on wheat yield.

- It is an exciting suggestion. We are going to study the wheat yield in future studies. In this study, we focused on the effect of freezing stress on herbicide efficacy in the early stages of plant species. In general, it is predicted that wheat yield will decrease due to herbicide efficiency and its adverse effects on the crop (selectivity index).

Response to the comments of the reviewer #2

We greatly appreciate the reviewer's positive comments. As indicated below, we have checked all the general and specific comments provided by the referees and have made necessary changes according to their indications, which are highlighted in yellow.

Reviewer #2: Dear Authors,

Reviewer comments PONE-D-21-33766

The manuscript entitled „Freezing stress affects the efficacy of clodinafop-propargyl and 2,4-D plus MCPA on wild oat and turnipweed in wheat“ represents a useful study focused on the impact of freezing stress on the efficiency of two herbicides treatments, clodinafop-propargyl and 2,4-D plus MCPA treatments, resulting in the statement that freezing stress attenuates the efficiency of herbicides in weed elimination as indicated by a decreased selectivity index. This result also indicates that freezing increases potential harmfulness of these herbicides for wheat as a crop.

I have only a few minor comments on the present manuscript:

In Materials and methods, Statistical analysis, as well as in Results, Table 1, a kind of a post-hoc test following ANOVA analysis used for determination of significant differences, has to be specified both in Materials and methods, Statistical analysis section and in results, Table 1 legend. Moreover, in Table 1 legend, it has to be clearly stated that the presented data represent mean±SE, and the number of replicates has to be specified.

- The requested information has been added to the text and Table 1.

Further formal comments on the manuscript text:

Abstract, lines 37-38: Add a comma both preceding and following the words „for 2,4-D plus MCPA,…“

- The requested revision was applied to the text.

Introduction, line 53: Use the term „winter cereals“ instead of „autumn cereals“ for autumn-sown cereals which have vernalization requirement thus they require winter temperatures prior to flowering.

- The requested revision was applied to the text.

Introduction, line 59: Use the term „optimum climatic conditions“ instead of „normal climatic conditions“.

- The requested revision was applied to the text.

Materials and methods, line 109: Replace the verb „obtain“ with „induce“ in the statement „To induce plant cold acclimation, the plants were kept outdoors,…“

- The requested revision was applied to the text.

Materials and methods, line 125: The authors wrote that „Freezing treatment was performed using a thermogradient freezer.“ However, basic information on the freezer has to be given including the manufacturer (company). Was the freezing treatment always just to -4 °C?

- The requested information was added to the text.
- Trend of temperature decrease in thermogradient freezer during the night from 7:00 P.M. to 5:00 A.M. (Please see Fig. 2 and gray highlight)

**Time**	**Temperature (℃)**
7 P.M.	5
12 A.M.	Reach a temperature of 0
1 A.M.	kept at 0
2 A.M.	Reach a temperature of -2
3 A.M.	kept at -2
4 A.M.	Reach a temperature of -4
5 A.M.	kept at -4
5:30 A.M.	Removed from the freezer

Line 175: Remove „the“ preceding the term „relative potency“.

- The requested revision was applied to the text.

Line 182: Modify the statement as follows: „Relative potency < 1 indicates the decreased effectiveness of the herbicides under freezing stress with respect to optimum conditions.“

- The requested revision was applied to the text.

Results, Figure 4 legend, line 209; Figure 5 legend, line 246: Write rather „64 g ha-1“, not „64 g ai ha-1“.

- The requested revision was applied to the text.

Results, line 252: Use a plural form of the verb „were“ instead of „was“ in the statement „The GR50 of NF, FBS, and FAS were 176, 299, and 208 g ha-1, respectively…“

- The requested revision was applied to the text.

Line 263: Remove „the“ preceding the term „selectivity index“ and modify the statement as follows: „As shown in Table 2, selectivity index values for clodinafop-propargyl in NF, FBS, and FAS treatments were 2.4, 0.91, and 0.78, respectively, and, for 2,4-D plus MCPA, they were 2.6, 0.12, and 0.88, respectively.“

- The requested revision was applied to the text.

Discussion, line 299: Remove „the“ Preceding the chemical name „clodinafop propargyl“.

- The requested revision was applied to the text.

Discussion, line 332: Replace the word „less“ with „lower“ in the statement „The relative potency values of clodinafop-propargyl and 2,4-D plus MCPA were lower than one under both FBS and FAS treatments in the present experiment.“

- The requested revision was applied to the text.

Discussion, line 343: Modify the statement into a plural form as follows: „The LD10 values of 2,4-D plus MCPA in NF were 89% and 48% higher compared to FBS and FAS, respectively.“

- The requested revision was applied to the text.

We look forward to hearing from you regarding our submission. We would be glad to respond to any further questions and comments that you may have.
