# Supplementary material for: Freezing stress affects the efficacy of clodinafop-propargyl and 2,4-D plus MCPA on wild oat (Avena ludoviciana Durieu) and turnipweed [Rapistrum rugosum (L.) All.] in wheat (Triticum aestivum L.)
Source: PLoS One. 2022 Oct 6;17(10):e0274945. doi: 10.1371/journal.pone.0274945 (PMC9536574; doi:10.1371/journal.pone.0274945)
Supplement: Attachment — Submitted filename: PONE-D-21-33766_R4 Academic editor.pdf [file pone.0274945.s010.pdf]

PLOS ONE

Freezing stress affects the efficacy of clodinafop-propargyl and 2,4-D plus MCPA on wild oat (Avena ludoviciana Durieu) and turnipweed [Rapistrum rugosum (L.) All.] in wheat (Triticum aestivum L.)

--Manuscript Draft--

**Manuscript Number:**	PONE-D-21-33766R4
**Article Type:**	Research Article
**Full Title:**	Freezing stress affects the efficacy of clodinafop-propargyl and 2,4-D plus MCPA on wild oat (Avena ludoviciana Durieu) and turnipweed [Rapistrum rugosum (L.) All.] in wheat (Triticum aestivum L.)
**Short Title:**	Freezing and herbicide efficacy
**Corresponding Author:**	Mehdi Rastgoo<br>Ferdowsi University of Mashhad<br>Mashhad, IRAN, ISLAMIC REPUBLIC OF
**Keywords:**	GR50; Herbicide efficacy; LD50; Selectivity index
**Abstract:**	The occurrence of freezing stress around herbicides application is one of the most important factors influencing their performance. This experiment was performed to evaluate the efficacy of clodinafop-propargyl and 2,4-D plus MCPA (2,4-Dichlorophenoxyacetic acid plus 2-methyl-4-chlorophenoxyacetic acid), the most important herbicides used in wheat fields in Iran, under the influence of a freezing treatment (-4°C). Wheat and its two common weeds, winter wild oat (Avena ludoviciana Durieu) and turnipweed [Rapistrum rugosum (L.) All.], were exposed to the freezing treatment for three nights from 7:00 P.M. to 5:00 A.M. before and after herbicide application, and their response was compared with plants that did not grow under freezing stress. Under no freezing (NF) and freezing after spray (FAS) conditions, winter wild oat was completely controlled with the recommended dose of clodinafop-propargyl (64 g ai ha^{-1}; hereafter g ha^{-1}). However, the survival percentage of winter wild oat in the freezing before spray (FBS) of clodinafop-propargyl 64 g ha^{-1} was 7%, and it was completely controlled with twice the recommended dose (128 g ha^{-1}). Under NF conditions and FAS treatment, turnipweed was completely controlled with twice the recommended dose of 2,4-D plus MCPA (2025 g ae ha^{-1}; hereafter g ha^{-1}), while there was no complete control under recommended rate. However, in the FBS treatment, the survival of turnipweed was 7% under double dose. The LD_{50} (dose required to control 50% of individuals in the population) and GR_{50} (dose causing 50% growth reduction of plants) rankings were NF<FBS<FAS for clodinafop-propargyl and NF<FAS<FBS for 2,4-D plus MCPA. Selectivity index for clodinafop-propargyl in NF conditions, FBS, and FAS treatments was 2.4, 0.91, and 0.78, respectively, and, for 2,4-D plus MCPA, it was 2.6, 0.12, and 0.88, respectively. According to the results of LD_{50}, it can be stated that the freezing stress after the spraying of clodinafop-propargyl and before the spraying of 2,4-D plus MCPA would further reduce the efficacy of these herbicides.
**Order of Authors:**	Alireza Hasanfard
	Mehdi Rastgoo
	Ebrahim Izadi Darbandi
	Ahmad Nezami
	Bhagirath Singh Chauhan
**Opposed Reviewers:**	
**Response to Reviewers:**	Manuscript Number: PONE-D-21-33766<br><br>Manuscript title: Freezing stress affects the efficacy of clodinafop-propargyl and 2,4-D plus MCPA on wild oat (Avena ludoviciana Durieu) and turnipweed [Rapistrum rugosum (L.) All.] in wheat (Triticum aestivum L.)

Powered by Editorial Manager® and ProduXion Manager® from Aries Systems Corporation

Dear Dr Uludag,

I want you to know how much I value your comments. Thank you very much for organizing the review of our manuscript. We have addressed all the comments/issues raised by the reviewers. The suggestions have improved the manuscript significantly. Our specific responses are given below. We hope to hear from you soon.

Sincerely,
Corresponding author

I carefully reviewed your revisions/comments. I applied most of them and presented answers for some below.

Editor's comments

Is it not controversial with line 30-31? "FBS caused 7% survival" you have mentioned

-" The requested revision was added to the text. According to the results of LD50"
Is this not contraversial with abstract? As far as I understood NF and FBS caused 50% control similar for NF and FBS but FAS required more herbicide to kill 50% of the population.
According to Table 1, You should mention in the abstract clerary the difference between two herbicides/weeds because one needs hghre rate in FAS the other in FBS.
-The last sentence of the abstract was revised based on LD50.
You can mention in the abstract that FR50 and LD50 gave the similar outcomes. I think the final interpretation will not change for both outputs.
-We mentioned this result in another way in the Abstract.
"The LD50 (dose required to control 50% of individuals in the population) and GR50 (dose causing 50% growth reduction of plants) rankings were NF<FBS<FAS for clodinafop-propargyl and NF<FAS<FBS for 2,4-D plus MCPA."
Your data does not says twice. It is a value between x and 2x according to tables and figures you have given.

In addition is it FAS or FBS. I confused much.
Please see Figure 4 (A). For complete control of wild oat in FBS treatment, double the recommended dose (128 g ai ha-1) is needed, and the sentence is correct.Is it not controversial with former sentence?
-Please see Figure 4
. This sentence is also controversial with the first sentence. I think the first sentence is not correct.
-Please note that the first sentence is related to Figure 4. The discussion of LD50 is different and clarified in Table 1.
It is confusing for me FAS requires this not FBS. Am I wrong in interpretation of figure 4 and Table 1?
-Please see Figure 4 (B). The sentence is correct.
-
This is what I have seen in your results but I mentioned above that some of your statements are controversial with this statetment. Check your abstract as well.
Check the previous sentence. According to GR50, this sentence is mentioned. The sentence was improved.When you discusss effect of herbicides under freezing use all data together. You can add this paragraph above paragraphs. Because LD50, GR50, potency, all shows the same conclusion
I think using less word to explain your data makse it easier for the readers.
-Yes, I accept your opinion. We did this in the Conclusions. It confirms the relative potency of the previous sections.
This is eactly what your data shows. Some sentemces I have shown above are contoversial. Could you please check them carefully.
-I appreciate your attention. I carefully checked different parts of the MS. Ambiguities are resolved when we refer to figures and tables. I have made some changes to the paper.
I do not think so this is result of your experiment for clodinafop. FBS and NF is not statistically different for clodinaforp. please follow the what statstics tell.

Powered by Editorial Manager® and ProduXion Manager® from Aries Systems Corporation

	-The sentence was deleted.<br>I recommend you that please avoid generilazation. You can say an ACCase inhibitor clodinafop... It helps your aim without generilazation. As you know not all chemistries have similar effects.<br>-Yes, your opinion is correct. Our sentence is also such that it does not refer to all the herbicides of this family.<br>As I mentioned above use winter wild oat, a narrow leaf weed. It helps you avoiding unnecessary genetralization.<br><br>-The requested revision was applied to the text.<br>I do not think you need this psrt.<br>-The sentence was deleted.<br>I think this is not discussed in your manuscript. Instead, you can say that these things should be researched.<br>-The requested revision was applied to the text.
**Additional Information:**	
**Question**	**Response**
**Financial Disclosure**<br><br>Enter a financial disclosure statement that describes the sources of funding for the work included in this submission. Review the submission guidelines for detailed requirements. View published research articles from *PLOS ONE* for specific examples.<br><br>This statement is required for submission and **will appear in the published article** if the submission is accepted. Please make sure it is accurate.<br><br>**Unfunded studies**<br>Enter: *The author(s) received no specific funding for this work.*<br><br>**Funded studies**<br>Enter a statement with the following details:<br>• Initials of the authors who received each award<br>• Grant numbers awarded to each author<br>• The full name of each funder<br>• URL of each funder website<br>• Did the sponsors or funders play any role in the study design, data collection and analysis, decision to publish, or preparation of the manuscript?<br>• **NO** - Include this sentence at the end of your statement: *The funders had no role in study design, data collection and analysis, decision to publish, or preparation of the manuscript.*<br>• **YES** - Specify the role(s) played.	We are grateful to Ferdowsi University of Mashhad, Iran, for providing financial support for present research (Project No.3.49759). No additional external funding was received for this study. The funders (Ferdowsi University of Mashhad, Iran and University of Queensland) had no role in study design, data collection and analysis, decision to publish, or preparation of the manuscript.

Powered by Editorial Manager® and ProduXion Manager® from Aries Systems Corporation

* typeset	
**Competing Interests**<br><br>Use the instructions below to enter a competing interest statement for this submission. On behalf of all authors, disclose any competing interests that could be perceived to bias this work—acknowledging all financial support and any other relevant financial or non-financial competing interests.<br><br>This statement is required for submission and **will appear in the published article** if the submission is accepted. Please make sure it is accurate and that any funding sources listed in your Funding Information later in the submission form are also declared in your Financial Disclosure statement.<br><br>View published research articles from *PLOS ONE* for specific examples.<br><br>**NO authors have competing interests**<br><br>Enter: *The authors have declared that no competing interests exist.*<br><br>**Authors with competing interests**<br><br>Enter competing interest details beginning with this statement:<br><br>*I have read the journal's policy and the authors of this manuscript have the following competing interests: [insert competing interests here]*<br><br>* typeset	The authors have declared that no competing interests exist.
**Ethics Statement**<br><br>Enter an ethics statement for this submission. This statement is required if the study involved:<br><br>• Human participants<br>• Human specimens or tissue	N/A

Powered by Editorial Manager® and ProduXion Manager® from Aries Systems Corporation

• Vertebrate animals or cephalopods<br>• Vertebrate embryos or tissues<br>• Field research<br><br>Write "N/A" if the submission does not require an ethics statement.<br><br>General guidance is provided below. Consult the submission guidelines for detailed instructions. **Make sure that all information entered here is included in the Methods section of the manuscript.**	

Powered by Editorial Manager® and ProduXion Manager® from Aries Systems Corporation

**Format for specific study types**<br><br>**Human Subject Research (involving human participants and/or tissue)**<br>• Give the name of the institutional review board or ethics committee that approved the study<br>• Include the approval number and/or a statement indicating approval of this research<br>• Indicate the form of consent obtained (written/oral) or the reason that consent was not obtained (e.g. the data were analyzed anonymously)<br><br>**Animal Research (involving vertebrate animals, embryos or tissues)**<br>• Provide the name of the Institutional Animal Care and Use Committee (IACUC) or other relevant ethics board that reviewed the study protocol, and indicate whether they approved this research or granted a formal waiver of ethical approval<br>• Include an approval number if one was obtained<br>• If the study involved *non-human primates*, add *additional details* about animal welfare and steps taken to ameliorate suffering<br>• If anesthesia, euthanasia, or any kind of animal sacrifice is part of the study, include briefly which substances and/or methods were applied<br><br>**Field Research**<br><br>Include the following details if this study involves the collection of plant, animal, or other materials from a natural setting:<br>• Field permit number<br>• Name of the institution or relevant body that granted permission	
**Data Availability**<br><br>Authors are required to make all data underlying the findings described fully available, without restriction, and from the time of publication. PLOS allows rare exceptions to address legal and ethical concerns. See the PLOS Data Policy and FAQ for detailed information.	Yes - all data are fully available without restriction

Powered by Editorial Manager® and ProduXion Manager® from Aries Systems Corporation

A Data Availability Statement describing where the data can be found is required at submission. Your answers to this question constitute the Data Availability Statement and **will be published in the article**, if accepted.<br><br>**Important:** Stating 'data available on request from the author' is not sufficient. If your data are only available upon request, select 'No' for the first question and explain your exceptional situation in the text box.<br><br>Do the authors confirm that all data underlying the findings described in their manuscript are fully available without restriction?	
**Describe where the data may be found in full sentences. If you are copying our sample text, replace any instances of XXX with the appropriate details.**<br><br>• If the data are **held or will be held in a public repository**, include URLs, accession numbers or DOIs. If this information will only be available after acceptance, indicate this by ticking the box below. For example: *All XXX files are available from the XXX database (accession number(s) XXX, XXX.*).<br>• If the data are all contained **within the manuscript and/or Supporting Information files**, enter the following: *All relevant data are within the manuscript and its Supporting Information files.*<br>• If neither of these applies but you are able to provide **details of access elsewhere**, with or without limitations, please do so. For example:<br><br>*Data cannot be shared publicly because of [XXX]. Data are available from the XXX Institutional Data Access / Ethics Committee (contact via XXX) for researchers who meet the criteria for access to confidential data.*<br><br>*The data underlying the results presented in the study are available from (include the name of the third party*	All relevant data are within the paper and its Supporting Information files.

and contact information or URL).<br>• This text is appropriate if the data are owned by a third party and authors do not have permission to share the data.<br><br>* typeset	
Additional data availability information:	

Cover letter

Dear Editors-in-Chief

We wish to submit an original research article entitled "Freezing stress affects the efficacy of clodinafop-propargyl and 2,4-D plus MCPA on wild oat and turnipweed in wheat" for consideration by Plos One.

We confirm that this work is original and has not been published elsewhere, nor is it currently under consideration for publication elsewhere. The authors have been participated in the preparation of the manuscript, and have given their permission to submit this manuscript with its present format.

The efficacy of herbicides is significantly affected by environmental conditions before, during, and after application. The occurrence of severe freezing that causes damage to plant leaves will have adverse effects on the activity and efficacy of foliar-applied herbicides. The present experiment aimed to investigate:

1. The effect of freezing stress on the performance of herbicides (clodinafop-propargyl and 2,4-D plus MCPA)
2. The effect of freezing stress on the relative potency
3. The selective performance of herbicides.

The results of this study could be very informative for the researchers, farmers, and herbicide producers.

We have no conflicts of interest to disclose.

Please address all correspondence concerning this manuscript to me at m.rastgoo@um.ac.ir

Thank you for your consideration of this manuscript.

Sincerely,

Dr Mehdi Rastgoo
Associate professor
Department of Agrotechnology, Faculty of Agriculture, Ferdowsi University of Mashhad, Iran
Corresponding author

Journal Requirements:

When submitting your revision, we need you to address these additional requirements.

1. Please ensure that your manuscript meets PLOS ONE's style requirements, including those for file naming. The PLOS ONE style templates can be found at

https://journals.plos.org/plosone/s/file?id=wjVg/PLOSOne_formatting_sample_main_body.pdf and

https://journals.plos.org/plosone/s/file?id=ba62/PLOSOne_formatting_sample_title_authors_affiliations.pdf

- The manuscript matches the style of PLOS ONE.

2. Thank you for stating the following financial disclosure:

"No"

At this time, please address the following queries:

a) Please clarify the sources of funding (financial or material support) for your study. List the grants or organizations that supported your study, including funding received from your institution.

1. Ferdowsi University of Mashhad, Iran provide financial support for present research (Project No.3.49759).
2. University of Queensland, will provide probable publishing fee Up to $500 if necessary.

No additional external funding was received for this study.

b) State what role the funders took in the study. If the funders had no role in your study, please state: “The funders had no role in study design, data collection and analysis, decision to publish, or preparation of the manuscript.”

The funders (Ferdowsi University of Mashhad, Iran and University of Queensland) had no role in study design, data collection and analysis, decision to publish, or preparation of the manuscript

c) If any authors received a salary from any of your funders, please state which authors and which funders.

- None

d) If you did not receive any funding for this study, please state: “The authors received no specific funding for this work.”

- Please include your amended statements within your cover letter; we will change the online submission form on your behalf.

3. Thank you for stating the following in the Acknowledgments Section of your manuscript:

"We are grateful to Ferdowsi University of Mashhad, Iran, for providing financial support for present research (Project No.3.49759) and University of Queensland for providing publishing fee. No additional external funding was received for this study."

We note that you have provided funding information that is not currently declared in your Funding Statement. However, funding information should not appear in the Acknowledgments section or other areas of your manuscript. We will only publish funding information present in the Funding Statement section of the online submission form.
Please remove any funding-related text from the manuscript and let us know how you would like to update your Funding Statement. Currently, your Funding Statement reads as follows:

"We are grateful to Ferdowsi University of Mashhad, Iran, for providing financial support for present research (Project No.3.49759) and University of Queensland for providing publishing fee. No additional external funding was received for this study."

Please include your amended statements within your cover letter; we will change the online submission form on your behalf.

"We are grateful to Ferdowsi University of Mashhad, Iran, for providing financial support for present research (Project No.3.49759) and University of Queensland for providing publishing fee. No additional external funding was received for this study."

4. In your Data Availability statement, you have not specified where the minimal data set underlying the results described in your manuscript can be found. PLOS defines a study's minimal data set as the underlying data used to reach the conclusions drawn in the manuscript and any additional data required to replicate the reported study findings in their entirety. All PLOS journals require that the minimal data set be made fully available. For more information about our data policy, please see http://journals.plos.org/plosone/s/data-availability.

"Upon re-submitting your revised manuscript, please upload your study's minimal underlying data set as either Supporting Information files or to a stable, public repository and include the relevant URLs, DOIs, or accession numbers within your revised cover letter. For a list of acceptable repositories, please see http://journals.plos.org/plosone/s/data-availability#loc-recommended-repositories. Any potentially identifying patient information must be fully anonymized.

Important: If there are ethical or legal restrictions to sharing your data publicly, please explain these restrictions in detail. Please see our guidelines for more information on what we consider unacceptable restrictions to publicly sharing data: http://journals.plos.org/plosone/s/data-availability#loc-unacceptable-data-access-restrictions. Note that it is not acceptable for the authors to be the sole named individuals responsible for ensuring data access.

We will update your Data Availability statement to reflect the information you provide in your cover letter.

- Experiment data are available and upload as Supporting Information files

5. PLOS requires an ORCID iD for the corresponding author in Editorial Manager on papers submitted after December 6th, 2016. Please ensure that you have an ORCID iD and that it is validated in Editorial Manager. To do this, go to ‘Update my Information’ (in the upper left-hand corner of the main menu), and click on the Fetch/Validate link next to the ORCID field. This will take you to the ORCID site and allow you to create a new iD or authenticate a pre-existing iD in Editorial Manager. Please see the following video for instructions on linking an ORCID iD to your Editorial Manager account: https://www.youtube.com/watch?v=_xcclfuvtxQ

ORCID iD validated in Editorial Manager.
Author for correspondence: Mehdi Rastgoo, ORCID iD: https://orcid.org/0000-0002-1974-8727

Cover letter

Dear Editors-in-Chief

We wish to submit an original research article entitled "Freezing stress affects the efficacy of clodinafop-propargyl and 2,4-D plus MCPA on wild oat and turnipweed in wheat" for consideration by Plos One.

We confirm that this work is original and has not been published elsewhere, nor is it currently under consideration for publication elsewhere. The authors have been participated in the preparation of the manuscript, and have given their permission to submit this manuscript with its present format.

The efficacy of herbicides is significantly affected by environmental conditions before, during, and after application. The occurrence of severe freezing that causes damage to plant leaves will have adverse effects on the activity and efficacy of foliar-applied herbicides. The present experiment aimed to investigate:

1. The effect of freezing stress on the performance of herbicides (clodinafop-propargyl and 2,4-D plus MCPA)
2. The effect of freezing stress on the relative potency
3. The selective performance of herbicides.

The results of this study could be very informative for the researchers, farmers, and herbicide producers.

We have no conflicts of interest to disclose.

Please address all correspondence concerning this manuscript to me at m.rastgoo@um.ac.ir

Thank you for your consideration of this manuscript.

Sincerely,

Dr Mehdi Rastgoo
Associate professor
Department of Agrotechnology, Faculty of Agriculture, Ferdowsi University of Mashhad, Iran
Corresponding author

Journal Requirements:

Please review your reference list to ensure that it is complete and correct. If you have cited papers that have been retracted, please include the rationale for doing so in the manuscript text, or remove these references and replace them with relevant current references. Any changes to the reference list should be mentioned in the rebuttal letter that accompanies your revised manuscript. If you need to cite a retracted article, indicate the article's retracted status in the References list and also include a citation and full reference for the retraction notice.

- **The mentioned items were checked.**

When submitting your revision, we need you to address these additional requirements.

1. Please ensure that your manuscript meets PLOS ONE's style requirements, including those for file naming. The PLOS ONE style templates can be found at

https://journals.plos.org/plosone/s/file?id=wjVg/PLOSOne_formatting_sample_main_body.pdf and

https://journals.plos.org/plosone/s/file?id=ba62/PLOSOne_formatting_sample_title_authors_affiliations.pdf

- The manuscript matches the style of PLOS ONE.

2. Thank you for stating the following financial disclosure:

"No"

At this time, please address the following queries:

a) Please clarify the sources of funding (financial or material support) for your study. List the grants or organizations that supported your study, including funding received from your institution.

1. Ferdowsi University of Mashhad, Iran provide financial support for present research (Project No.3.49759).
2. University of Queensland, will provide probable publishing fee Up to $500 if necessary.

No additional external funding was received for this study.

b) State what role the funders took in the study. If the funders had no role in your study, please state: "The funders had no role in study design, data collection and analysis, decision to publish, or preparation of the manuscript."

The funders (Ferdowsi University of Mashhad, Iran and University of Queensland) had no role in study design, data collection and analysis, decision to publish, or preparation of the manuscript

c) If any authors received a salary from any of your funders, please state which authors and which funders.

- None

d) If you did not receive any funding for this study, please state: “The authors received no specific funding for this work.”

- Please include your amended statements within your cover letter; we will change the online submission form on your behalf.

3. Thank you for stating the following in the Acknowledgments Section of your manuscript:

"We are grateful to Ferdowsi University of Mashhad, Iran, for providing financial support for present research (Project No.3.49759) and University of Queensland for providing publishing fee. No additional external funding was received for this study."

We note that you have provided funding information that is not currently declared in your Funding Statement. However, funding information should not appear in the Acknowledgments section or other areas of your manuscript. We will only publish funding information present in the Funding Statement section of the online submission form.
Please remove any funding-related text from the manuscript and let us know how you would like to update your Funding Statement. Currently, your Funding Statement reads as follows:

"We are grateful to Ferdowsi University of Mashhad, Iran, for providing financial support for present research (Project No.3.49759) and University of Queensland for providing publishing fee. No additional external funding was received for this study."

Please include your amended statements within your cover letter; we will change the online submission form on your behalf.

"We are grateful to Ferdowsi University of Mashhad, Iran, for providing financial support for present research (Project No.3.49759) and University of Queensland for providing publishing fee. No additional external funding was received for this study."

4. In your Data Availability statement, you have not specified where the minimal data set underlying the results described in your manuscript can be found. PLOS defines a study's minimal data set as the underlying data used to reach the conclusions drawn in the manuscript and any additional data required to replicate the reported study findings in their entirety. All PLOS journals require that the minimal data set be made fully available. For more information about our data policy, please see http://journals.plos.org/plosone/s/data-availability.

"Upon re-submitting your revised manuscript, please upload your study's minimal underlying data set as either Supporting Information files or to a stable, public repository and include the relevant URLs, DOIs, or accession numbers within your revised cover letter. For a list of acceptable repositories, please see http://journals.plos.org/plosone/s/data-availability#loc-recommended-repositories. Any potentially identifying patient information must be fully anonymized.

Important: If there are ethical or legal restrictions to sharing your data publicly, please explain these restrictions in detail. Please see our guidelines for more information on what we consider unacceptable restrictions to publicly sharing data: http://journals.plos.org/plosone/s/data-availability#loc-unacceptable-data-access-restrictions. Note that it is not acceptable for the authors to be the sole named individuals responsible for ensuring data access.

We will update your Data Availability statement to reflect the information you provide in your cover letter.

- Experiment data are available and upload as Supporting Information files

5. PLOS requires an ORCID iD for the corresponding author in Editorial Manager on papers submitted after December 6th, 2016. Please ensure that you have an ORCID iD and that it is validated in Editorial Manager. To do this, go to 'Update my Information' (in the upper left-hand corner of the main menu), and click on the Fetch/Validate link next to the ORCID field. This will take you to the ORCID site and allow you to create a new iD or authenticate a pre-existing iD in Editorial Manager. Please see the following video for instructions on linking an ORCID iD to your Editorial Manager account: https://www.youtube.com/watch?v=_xcclfuvtxQ

ORCID iD validated in Editorial Manager.
Author for correspondence: Mehdi Rastgoo, ORCID iD: https://orcid.org/0000-0002-1974-8727

Manuscript Click here to access/download;Manuscript;Revised Manuscript (3).docx

Freezing stress affects the efficacy of clodinafop-propargyl and 2,4-D plus MCPA on wild oat (*Avena ludoviciana* Durieu) and turnipweed [*Rapistrum rugosum* (L.) All.] in wheat (*Triticum aestivum* L.)

Short running title: Freezing and herbicide efficacy

Alireza Hasanfard[1], Mehdi Rastgoo[1], Ebrahim Izadi Darbandi[1], Ahmad Nezami[1], Bhagirath Singh Chauhan[2]

[1] Department of Agrotechnology, Faculty of Agriculture, Ferdowsi University of Mashhad, Iran

[2] Queensland Alliance for Agriculture and Food Innovation (QAAFI) *and* School of Agriculture and Food Sciences (SAFS), The University of Queensland, Gatton, Queensland 4343, Australia

***Corresponding author:** m.rastgoo@um.ac.ir

Abstract

The occurrence of freezing stress around herbicides application is one of the most important factors influencing their performance. This experiment was performed to evaluate the efficacy of clodinafop-propargyl and 2,4-D plus MCPA (2,4-Dichlorophenoxyacetic acid plus 2-methyl-4-chlorophenoxyacetic acid), the most important herbicides used in wheat fields in Iran, under the influence of a freezing treatment (-4℃). Wheat and its two common weeds, winter wild oat (*Avena ludoviciana* Durieu) and turnipweed [*Rapistrum rugosum* (L.) All.], were exposed to the freezing treatment for three nights from 7:00 P.M. to 5:00 A.M. before and after herbicide application, and their response was compared with plants that did not grow under freezing stress. Under no freezing (NF) and freezing after spray (FAS) conditions, winter wild oat was completely controlled with the recommended dose of clodinafop-propargyl (64 g ai ha^{-1}; hereafter g ha^{-1}). However, the survival percentage of winter wild oat in the freezing before spray (FBS) of clodinafop-propargyl 64 g ha^{-1} was 7%, and it was completely controlled with twice the recommended dose (128 g ha^{-1}). Under NF conditions and FAS treatment, turnipweed was completely controlled with twice the recommended dose of 2,4-D plus MCPA (2025 g ae ha^{-1}; hereafter g ha^{-1}). However, in the FBS treatment, the survival of turnipweed was 7% (2025 g ha^{-1}). The LD$_{50}$ (dose required to control 50% of individuals in the population) and GR$_{50}$ (dose causing 50% growth reduction of plants) rankings were NF<FBS<FAS for clodinafop-propargyl and NF<FAS<FBS for 2,4-D plus MCPA. Selectivity index for clodinafop-propargyl in NF conditions, FBS, and FAS treatments was 2.4, 0.91, and 0.78, respectively, and, for 2,4-D plus MCPA, it was 2.6, 0.12, and 0.88, respectively. According to the results of this experiment, it can be stated that the freezing stress after the spraying of clodinafop-propargyl and the freezing stress before a spray of 2,4-D plus MCPA would further reduce the efficacy of these herbicides.

Keywords: GR_{50}, Herbicide efficacy, LD_{50}, Selectivity index

Introduction

Winter wild oat (*Avena ludoviciana* Durieu) and turnipweed [*Rapistrum rugosum* (L.) All.] are the most important annual weeds of winter wheat (*Triticum aestivum* L.) in Iran, which cause significant reductions in wheat yield [1, 2]. The high reproduction potential and fitness to freezing stress, which results in a wide dispersal of weed seeds are the major challenges in the management of winter annual weeds like wild oat and turnipweed. Therefore, in cold regions of Iran, the dispersal and geographical distribution of these weeds are more significant than other weed species [3].

One of the most important consequences of climate change is the change in glaciers' occurrence patterns [4]. A sharp drop in temperature after high temperatures leads to crop damage. Increasing autumn temperatures in Iran, resulting from climate change, have led to reduced freezing tolerance of autumn crops [3]. In other words, autumn crops will not tolerate freezing stress due to the lack of hardening process in autumn. As a result, they will be damaged by decreasing temperatures during winter, and their yield will be reduced [3, 4]. It has been reported that increasing autumn temperatures resulting from climate change in western Canada led to reduced freezing tolerance of winter cereals [5]. In other words, due to the lack of optimal cold acclimation in autumn, crops will not be able to tolerate freezing stress. As a result, they will be damaged by decreasing temperatures during winter or by the occurrence of late spring frosts [6]. However, weeds generally have a higher ability to tolerate freezing stress [7]. Based on this, it can be inferred that climate change is adversely changing agricultural production and can significantly change the management of current cropping systems that have evolved under optimum climatic conditions.

Hand weeding and hoeing are not common in wheat fields, and mechanical weed management methods are not effective, so chemical methods are mainly used to control weeds in the wheat fields in Iran. Clodinafop-propargyl and 2,4-D plus MCPA (2,4-Dichlorophenoxyacetic acid plus 2-methyl-4-chlorophenoxyacetic acid) are the most common herbicides used for controlling weeds in wheat fields in Iran. Due to the particular importance of this crop in food security, their wide excessive applications can be justified [8, 9]. Clodinafop-propargyl is a systemic and selective herbicide of the aryloxyphenoxypropionate (AOPP) group and used post-emergent to control annual grass weeds in wheat. This herbicide inhibits the activity of acetyl-coenzyme A carboxylase (ACCase) and thus disrupts the biosynthesis of fatty acids [10]. 2,4-D plus MCPA, synthetic auxin herbicides, is a systemic herbicide mixture registered for selective post-emergence control of annual broadleaf weeds in wheat [11].

The efficacy of herbicides is significantly affected by environmental conditions before, during, and after application [12]. It has been reported that environmental factors such as light intensity, temperature, water stress, relative humidity, and nutrient availability alter the herbicide efficacy [13, 14]. On the other hand, plant species and their growth stages also affect herbicide efficacy [15]. Numerous studies indicate that ambient temperature is one of the most influential factors affecting the performance of herbicides [14, 16, 17]. Increasing the temperature is likely to increase the absorption and translocation of some herbicides and increase their performance. In contrast, the improvement in herbicide performance at low temperatures may be due to a reduction in the rate of herbicide metabolism [17, 18]. In cold regions, freezing temperatures occur around the time of herbicide application. Therefore, the occurrence of severe freezing that causes damage to plant leaves will have adverse effects on the activity and efficacy of foliar-applied herbicides [19]. On the other hand, application of herbicides in unfavorable

environmental conditions (such as adverse temperature before, during, and after herbicide application) causes stress in the crop, and this changes the selectivity index of the herbicide, which will ultimately cause damage to the crop [20]. The selectivity index refers to the potential of a herbicide to eliminate weeds in a crop without affecting the yield or quality of the final product [21].

At the appropriate growth stage of weeds for herbicide application, the temperature usually decreases, and this issue leads to a decrease in the performance of herbicides. Due to the lack of information on the efficacy of herbicides under freezing stress, this experiment was conducted to evaluate the efficacy of clodinafop-propargyl and 2,4-D plus MCPA on wild oat and turnipweed under freezing stress before and after herbicide application.

Materials and methods

Experimental site and plant material

Seeds of wheat (cv. Mihan) were harvested from Khorasan Razavi Agricultural and Natural Resources Research and Education Center, and seeds of winter wild oat and turnipweed were collected (collected from about 500 plants) from infested farms around Mashhad-Iran in May 2019. Experiments were conducted at the Faculty of Agriculture, the Ferdowsi University of Mashhad, Iran (Lat 36° 15′ N, Long 59° 28 E; 985 m Altitude). To break the dormancy of winter wild oat and turnipweed, their seeds were exposed to moist-chilling treatment and potassium nitrate (KNO_3) 0.2% solution for 3 days at 5°C in the dark, respectively [3]. Weed seeds were placed in 9-cm diameter Petri dishes (20-25 seeds per Petri dish) on moist filter papers and kept at 20°C. After 5 days, the germination percentage of winter wild oat and turnipweed seeds was >90%. The experiment was repeated twice with three replicates. To experience the same cold

acclimation, the plants were planted at approximately the same time on October 21 (first run) and 24 (second run) in 2019. Plastic pots (12 cm in diameter and 20 cm in height) were used in the trial, and pots were filled with a mixture of sand, agricultural soil, and peat moss (2:2:1 sand, agricultural soil, and peat moss, respectively). For uniform emergence, turnipweed germinated seeds were planted in a seedling tray. Eight turnipweed seedlings transferred to pots in the cotyledon stage. Eight germinated seeds of wild oat were planted at 4-5 cm depth below the soil surface. Wheat seeds were planted directly in each pot at the same depth. To induce cold acclimation, the plants were kept outdoors, grown in natural conditions (Fig. 1), and irrigated according to daily requirements until they were tested.

Fig 1. Minimum and maximum daily air temperatures at the experimental site from October 2019 to February 2020. Relative humidity of 40-50% under the natural photoperiod [3].

Experimental factors

Experimental factors included freezing stress at three conditions, including no freezing (NF), freezing before spray (FBS), and freezing after spray (FAS). Plant species included wheat, winter wild oat, and turnipweed. Herbicides were applied at 0, 6.25, 12.5, 25, 50, 100, and 200% of the recommended dose for the herbicide clodinafop-propargyl (0.8 L ha^{-1}; Topik, 8% EC) and 2,4-D plus MCPA (1.5 L ha^{-1}; U 46 combi fluid, 67.5% SL).

Freezing treatment and herbicide application

Under NF, the plants grew completely in natural winter conditions without exposing to freezing stress. For FBS, the plants grew up to the 3-4 leaf stage under natural conditions. They were exposed to freezing from 7:00 P.M. to 5:00 A.M. for three nights before herbicide application [19]. Freezing treatment was performed using a thermogradient freezer (Weiss Technik). The

initial temperature of the freezer was 5℃; however, the freezer was cooled down at a rate of 2℃ per hour to reach a temperature of -4℃. The temperature drop slope was adjusted based on the long-term meteorological statistics of Mashhad in the time range of herbicide application. The pots were kept at -4℃ for 1 h and then removed from the freezer (Fig. 2).

Fig. 2 Trend of temperature decrease in thermogradient freezer during the night from 7:00 P.M. to 5:00 A.M.

Each morning at 5:30 A.M., the plants were transferred out of the freezer, kept in natural conditions until 6:30 P.M., and then returned to the freezer during the night. After three nights of freezing exposure, the pots were taken out of the freezer and sprayed with herbicides after 2 h [19]. After spraying, the plants were kept outside under natural conditions for the duration of the experiment.

In the FAS treatment, freezing stress was applied for three nights by placing the samples treated with herbicides in the thermogradient freezer. For this purpose, the plants were taken out of the freezer at 5:30 A.M., transferred to natural conditions, and then returned to the freezer at 6:30 P.M. for the overnight freezing treatment (Similar to FBS treatment). Three nights after freezing treatment, the plants were transferred to natural conditions to continue the trial process (Fig. 3).

Fig. 3 Schematic diagram of the experimental study design. Abbreviation: FBS (freezing before spray); FAS (freezing after spray).

The conditions inside the thermogradient freezer were the same for both freezing treatments before and after spray. The experiment was performed in the freezer in completely dark conditions and at the same relative humidity (35±5%) for all treatments. Temperature changes in

the freezer were regularly monitored when the samples were in the freezer. For all the freezing treatments, herbicides were applied at the same time. Plants were transferred to natural conditions after three nights of freezing treatment at 5:30 A.M. Also, plants related to freezing after spray were transferred to the freezer at 6:30 P.M. On the same day, plants that were not exposed to freezing were sprayed from 7:30 A.M. to 10.30 A.M. The herbicides were applied by a researcher-made moving boom sprayer equipped with a TeeJet 8002 flat fan nozzle (spray output volume of 200 L ha^{-1} at a pressure of 260 kPa). Plants were returned and maintained outdoor under natural conditions of winter after the treatment.

Measurement of traits

Four weeks after herbicide treatments, survival percentage (Eq. 1) and shoot dry weight were measured by placing the samples in an oven at 75℃ for 48 h. Plants that produced new leaves were considered alive and those that experienced severe chlorosis and stunted growth were recorded as dead [22].

$$Survival\ \% = \frac{N1}{N0} \times 100 \qquad \text{(Eq. 1)}$$

Where, N_1 and N_0 are the numbers of alive plants four weeks after herbicide treatment and the number of alive plants before herbicide treatment, respectively.

Dose–response experiments

The response of weed traits to the doses of herbicides tested was analyzed by nonlinear regression. For this purpose, the data were fitted to a three-parameter log-logistic equation (Eq. 2) [23].

$$Y = \frac{d}{1+e^{b(\log x - \log e)}} \quad \text{(Eq. 2)}$$

Where, Y is the response rate (survival % or dry weight) at dose x, d is the upper limit for Y, b is the slope of the curve around e, and e is the effective dose (g ai or ae ha^{-1}). Here, e is replaceable with LD_{50} (dose required to control 50% of individuals in the population) or GR_{50} (dose causing 50% dry weight reduction of plants).

Relative potency

Relative potency was used to determine herbicide efficacy under freezing stress and no freezing stress (Eq. 3) [24].

$$Relative\ potency = \frac{ED_{50A}}{ED_{50B}} \quad \text{(Eq. 3)}$$

Where, ED_{50A} is the dose required to reduce survival by 50% in NF, and ED_{50B} is the dose needed to reduce survival by 50% in FBS or FAS. Relative potency = 1 indicates that freezing treatment (FBS and FAS) did not affect herbicide efficacy. If relative potency > 1, it indicates that the herbicides are more efficient under freezing stress. Relative potency < 1 indicates the decreased effectiveness of the herbicides under freezing stress with respect to optimum conditions.

Selectivity index

The ratio between doses that caused 10% damage to crops and 90% damage to weeds was considered a selectivity index [24] and calculated using Eq. 4.

$$Selectivity\ index\ (10{,}90) = \frac{ED_{10\ (crop)}}{ED_{90\ (weed)}} \quad \text{(Eq. 4)}$$

Where, ED_{90} is 90% of the herbicide effect on weed survival (herbicide dose to kill 90% of the population), and ED_{10} is 10% of the herbicide impact on crop survival (a 10% effect on the crop). The higher the SI, the more selective the herbicide in the crop [24].

Statistical analysis

The experiments were conducted using a completely randomized design in factorial arrangement with three replications. Afterward, differences between treatments were compared with the standard error of mean (SEM) values. For data analysis, analysis of variance (ANOVA) was performed using the SAS software (v. 9.4, SAS Institute Inc, Cary, NC, USA). Data for both runs were pooled as no significant differences were observed between runs ($P > 0.05$). Homogeneity of variance was confirmed using Levene's test. Analysis of biometric data was performed by the R program (v. 4.0.4) [25]. Dose-response curves were also fitted with the asymmetrical sigmoid form. LD_{50} and GR_{50} were separated using 95% confidence intervals (CI).

Results

Clodinafop-propargyl

Survival percentage and LD_{50}

The dose-response experiments were similar for NF and FAS treatments at the recommended dose (Figure 4A). Thus, in both conditions, winter wild oat was completely controlled with the recommended dose (64 g ai ha^{-1}). However, the survival of seedlings in the FBS treatment was 7%, and to achieve complete control, 2-fold the recommended dose (128 g ha^{-1}) was needed. The LD_{50} in NF, FBS, and FAS was 17.9, 18.1, and 23.2 g ha^{-1}, respectively (Table 1).

Fig. 4 Survival (A) and dry weight (B) of winter wild oat treated with various doses of clodinafop-propargyl under different freezing treatments. Abbreviation: NF (no freezing); FBS (freezing before spray): FAS (freezing after spray). The recommended dose of clodinafop-propargyl in Iran is 64 g ha^{-1}.

Table 1 The dose of clodinafop-propargyl and 2,4-D plus MCPA required for 50% mortality (LD_{50}) and the dose that caused 50% inhibition of growth noted by biomass (GR_{50}) of winter wild oat and turnipweed under different freezing treatments.

Herbicide	Freezing Treatment	b±SE	d±SE	LD_{50} ±SE	95% CI	R^2
			%	g ai or ae ha^{-1}		
Clodinafop-propargyl	NF	2.0 ± 0.19**	100.3 ± 2.8**	17.9 ± 1.2**b	15.6-20.2	0.99
	FBS	1.9 ± 0.17**	101.0 ± 2.9**	18.1 ± 1.2**b	15.7-20.5	0.99
	FAS	1.9 ± 0.19**	98.7 ± 2.9**	23.2 ± 1.7**a	19.8-26.6	0.96
2,4-D plus MCPA	NF	1.9 ± 0.20**	102.2 ± 3.2**	292.8 ± 21.9**c	249.4-336.3	0.98
	FBS	1.5 ± 0.15**	102.4 ± 3.2**	472.8 ± 42.4**a	388.8-556.8	0.98
	FAS	1.5 ± 0.14**	102.8 ± 3.5**	318.8 ± 28.9**b	261.6-376.1	0.98
		b±SE	d±SE	GR_{50} ±SE	95% CI	
			g pot^{-1}	g ai or ae ha^{-1}		
Clodinafop-propargyl	NF	2.1 ± 0.15**	0.70 ± 0.02**	10.6 ± 0.52**c	9.6-11.6	0.99
	FBS	2.0 ± 0.12**	0.80 ± 0.02**	11.6 ± 0.51**b	10.6-12.6	0.99
	FAS	1.7 ± 0.12**	0.67 ± 0.02**	12.8 ± 0.74**a	11.3-14.3	0.98
2,4-D plus MCPA	NF	2.2 ± 0.23**	0.46 ± 0.01**	176.2 ± 11.3**c	153.9-198.6	0.99
	FBS	1.7 ± 0.16**	0.48 ± 0.01**	298.6 ± 21.5**a	256.0-341.3	0.99
	FAS	1.6 ± 0.15**	0.47 ± 0.01**	207.8 ± 15.9**b	176.2-239.4	0.99

Abbreviation: NF (no freezing); FBS (freezing before spray); FAS (freezing after spray); SE: Standard Error. b is the slope of the curve around e (LD_{50} and GR_{50}); d is the upper limit for Y (survival % or dry weight); **: significant at p≤0.01. LD_{50} and GR_{50} were separated using their 95% confidence intervals (CI). The data shows the parameter's mean ± SE. The data (LD_{50} and GR_{50}) with the different letters indicate significant differences according to LSD at P ≤0.05. n = 6

Dry weight and GR_{50}

The dry weight of winter wild oat in NF and FAS with the recommended dose (64 g ha^{-1}) and FBS with twice the recommended dose (128 g ha^{-1}) reached 0 g (Fig. 4B). The GR_{50} of plants not exposed to freezing stress was 10.6 g ha^{-1}. The GR_{50} was 11.6 and 12.8 g ha^{-1} in FBS and FAS, respectively (Table 1).

2,4-D plus MCPA

Survival percentage and LD_{50}

The dose-response experiments of 2,4-D plus MCPA (similar to clodinafop-propargyl) were the same in NF and FAS treatments (Fig. 5A). In both conditions, turnipweed was completely controlled with twice the recommended dose (2025 g ae ha^{-1}). However, in the FBS treatment, the survival of turnipweed was 7%. At the recommended dose (1012.5 g ha^{-1}), the survival of turnipweed in NF, FBS, and FAS was 3%, 33%, and 20%, respectively. The LD_{50} in NF, FBS, and FAS was 293, 473, and 319 g ha^{-1}, respectively (Table 1).

Fig. 5 Survival (A) and dry weight (B) of turnipweed treated with various doses of 2,4-D plus MCPA under different freezing treatments. Abbreviation: NF (no freezing); FBS (freezing before spray): FAS (freezing after spray). The recommended dose of 2,4-D+MCPA in Iran is 1012.5 g ha^{-1}.

Dry weight and GR_{50}

The dry weight of turnipweed in NF and FAS with twice the recommended dose (2025 g ha^{-1}) of 2,4-D plus MCPA was reduced to 0 (Fig. 5 B). However, in the FBS treatment, the dry weight of turnipweed with twice the recommended dose was 0.012 g per pot. At the recommended dose, the dry weight of turnipweed in FBS was 97% and 76% more than NF and FAS, respectively. The GR_{50} of NF, FBS, and FAS were 176, 299, and 208 g ha^{-1}, respectively (Table 1).

Relative potency

The relative potency of clodinafop-propargyl and 2,4-D plus MCPA was significantly reduced in FBS and FAS compared to NF (Fig. 6). Thus, the relative potency in FBS and FAS of clodinafop-propargyl was 0.99 and 0.77, respectively, and at 2,4-D plus MCPA was 0.62 and 0.92, respectively.

Fig. 6 Relative potency of clodinafop-propargyl and 2,4-D plus MCPA under different freezing treatments. Abbreviation: FBS (freezing before spray) and FAS (freezing after spray). The error bars indicate standard error (SE). The dashed line represents the relative potency = 1.0.

Selectivity index

As shown in Table 2, selectivity index values for clodinafop-propargyl in NF, FBS, and FAS treatments were 2.4, 0.91, and 0.78, respectively, and, for 2,4-D plus MCPA, they were 2.6, 0.12, and 0.88, respectively. For clodinafop-propargyl, the LD_{10} of wheat in NF was 127.9 g ha^{-1}. However, LD_{10} of wheat in the FBS and FAS was 53.5 and 57.1 g ha^{-1}, respectively (Table 2). For 2,4-D plus MCPA, the LD_{10} of wheat in NF, FBS, and FAS was 2380, 264, and 1236 g ha^{-1}, respectively.

Table 2 Sensitivity of plant species of wheat, winter wild oat, and turnipweed to clodinafop-propargyl and 2,4-D plus MCPA under different freezing treatments.

Freezing Treatment	Plant species	Clodinafop-propargyl		2,4-D plus MCPA	
		LD_{10} (wheat) and LD_{90} (winter wild oat) (g ai ha^{-1}) ± SE	SI	LD_{10} (wheat) and LD_{90} (turnipweed) (g ae ha^{-1}) ± SE	SI
NF	Wheat	127.9 ± 8.8	-	2380 ± 455	-
	Weed	53.5 ± 5.4	2.4	908 ± 108	2.6
FBS	Wheat	53.5 ± 11.0	-	264 ± 61.0	-
	Weed	59.0 ± 6.4	0.91	2144 ± 327	0.12
FAS	Wheat	57.1 ± 8.7	-	1236 ± 163	-
	Weed	73.6 ± 7.2	0.78	1400 ± 199	0.88

Abbreviation: NF; No freezing, FBS: Freezing before spray, FAS: Freezing after spray SI: Selectivity index. The selectivity index (SI) was calculated as follows: *Selectivity index (10, 90):* $\frac{ED10\ (crop)}{ED90\ (weed)}$

Discussion

The required dose of clodinafop-propargyl for complete control of winter wild oat seedlings in FBS treatment was twice that of NF and FAS treatments. The trend of the reducing survival % in seedlings related to the FBS treatment and NF was similar. The LD_{50} for clodinafop-propargyl in freezing treatments shows that exposure of winter wild oat seedlings to freezing treatment for three nights before and after spray reduces the efficacy of this herbicide compared to NF. The LD_{50} was 22% and 23% higher in the FAS compared to the FBS and NF, respectively. The slight difference between NF and FBS can be due to individual variability. Freezing treatment for three nights before and after spraying of clethodim herbicide (the mode of action similar to clodinafop-propargyl) led to an increase in LD_{50} [19]. It decreased herbicide efficacy in rigid ryegrass (*Lolium rigidum* Gaud.) populations compared to NF.

The dose of clodinafop-propargyl required to reduce winter wild oat biomass to zero in the FBS treatment was twice that of the NF and FAS treatments. The GR_{50} is more pronounced in FBS and FAS compared to NF, indicating a decrease in the efficacy of clodinafop-propargyl. The GR_{50} was 17% and 8% higher in the FAS treatment compared to NF and FBS, respectively. In other words, the occurrence of three nights of freezing stress after herbicide application reduced the performance of clodinafop-propargyl more than other treatments. It has been reported that wild oat plants (*Avena fatua*) that grew at low temperatures were less sensitive to difenzoquat than plants that grew at high temperatures [26].

Our results clearly show that the occurrence of freezing stress around spray reduces the efficacy of clodinafop-propargyl, especially in the treatment of FAS. Herbicide translocation in the phloem in freezing conditions could decline due to damage to the phloem cells, which may lead to a decrease in the herbicide efficacy. Decreased efficacy of sethoxydim at low

temperatures due to reduced herbicide translocation for Quackgrass [*Elymus repens* (L.) Gould] control was reported by Coupland [27]. In general, to maintain the herbicide efficacy under cold conditions, using clodinafop-propargyl after the freezing stress can be recommended in areas with a similar climate.

Although the three-night treatment of FBS and FAS resulted in a reduction in turnipweed control by 2,4-D plus MCPA compared to NF, this reduction was different in the two freezing treatments. At the recommended dose (1013 g ha^{-1}) in NF, almost all turnipweed plants were controlled, while the survival percentage of turnipweed in FBS and FAS treatments were 33% and 20%, respectively. Low temperatures around the time of herbicide application reduced the effectiveness of systemic herbicides [28]. Based on LD_{50} values, the efficacy of 2,4-D plus MCPA under FBS and FAS was 1.6 and 1.1 times lower compared to NF.

FAS is possible to damage the phloem cells, which reduces the translocation of the herbicide. The efficacy of 2,4-D plus MCPA in the treatment of FBS was reduced. The decrease in the effect of 2,4-D plus MCPA in the treatment of FBS is probably due to the reduction of absorption and translocation of this herbicide in turnipweed seedlings. In this regard, an increase in efficacy of 2,4-D and glyphosate at high temperatures compared to low temperatures in common ragweed (*Ambrosia artemisiifolia* L.) and giant ragweed (*Ambrosia trifida* L.) due to the increased absorption and/or translocation at high temperatures around herbicide application has been reported by Ganie et al. [29]. It has been also reported that low temperatures reduced glufosinate activity and translocation in wild radish (*Raphanus raphanistrum* L.) [30].

Turnipweed's dry weight at the recommended dose of 2,4-D plus MCPA (1012.5 g ha^{-1}) in FBS and FAS treatments was 35.5 and 8.5 times higher than the NF, respectively. Also, GR_{50} in FBS and FAS was 41% and 15.2% higher compared to NF, respectively. Although a decrease

in herbicide efficacy is evident in both FBS and FAS treatments, high GR_{50} in FBS shows an adverse effect of three nights of freezing before the application of 2,4-D plus MCPA. In other words, the efficacy of this herbicide reacts more to FBS than to FAS. In a similar study, the GR_{50} for 2,4-D increased at low temperature in common ragweed compared to high temperature [29].

The freezing stress around 2,4-D plus MCPA application reduced the efficacy of this herbicide in FBS and FAS treatments compared to NF. The decrease in the efficacy of this herbicide in the treatment of FBS is probably due to the reduction of absorption and translocation of the herbicide in the phloem during freezing. Absorption and translocation of 2,4-D plus MCPA might be reduced at low temperatures due to the effect on herbicide penetration facilitated by physicochemical factors, including the decreased rate of diffusion, increased viscosity of the cuticle, and physiological factors comprising decreases in photosynthesis, phloem translocation, and protoplasmic streaming [29]. In Iranian wheat fields, using the 2,4-D plus MCPA before the freezing stress can be recommended to maintain the herbicide efficacy under cold conditions.

When relative potency > 1, the herbicide efficacy is higher than optimal non-freezing conditions, and when relative potency < 1, the herbicide efficacy is lower than usual [24]. The relative potency values of clodinafop-propargyl and 2,4-D plus MCPA were lower than one under both FBS and FAS treatments in the present experiment. In other words, freezing stress reduced the efficacy of these herbicides. Based on the results of relative potency, the reduction of clodinafop-propargyl efficacy in the FAS treatment (33%) was more significant than in the FBS treatment (1%). Conversely, for 2,4-D plus MCPA, the reduction in efficacy was more significant in the FBS treatment (38%) compared to the FAS treatment (8%).

The selectivity index for clodinafop-propargyl and 2,4-D plus MCPA was > 1 in NF, indicating that both herbicides acted selectively in wheat. However, applying three nights of FBS and FAS of both herbicides reduced the selectivity index to less than one. In other words, freezing stress reduced the selectivity index of both herbicides, which led to phytotoxicity in wheat. For clodinafop-propargyl, LD_{10} of wheat in NF was 58% and 55% higher than FBS and FAS, respectively. The LD_{10} values of 2,4-D plus MCPA in NF were 89% and 48% higher compared to FBS and FAS, respectively. In other words, the dose required to kill 10% of wheat in both herbicides under NF was higher than FBS and FAS. Therefore, FBS and FAS increased the sensitivity of wheat to herbicide application. Freezing stress may reduce the rate of herbicide metabolism in the crop and ultimately increase its susceptibility [17].

Conclusions

The relative potency results for both herbicides were used to confirm the results of LD_{50} and GR_{50}. Thus, LD_{50} and GR_{50} were higher for clodinafop-propargyl in the FAS treatment than in NF and FBS treatments. The LD_{50} and GR_{50} were higher for the 2,4-D plus MCPA in the FBS treatment than in the NF and FAS treatments. In other words, clodinafop-propargyl was more effective than other conditions in the treatment of three nights of FAS and 2,4-D plus MCPA in the treatment of three nights of FBS. The selectivity index for clodinafop-propargyl was lower in the FAS treatment (0.78) and for 2,4-D plus MCPA was lower in the FBS treatment (0.12). The efficacy of both herbicides was affected by the FBS and FAS treatments. The efficacy of clodinafop-propargyl in the FAS treatment and the efficacy of 2,4-D plus MCPA in the FBS treatment were reduced. The values of the selectivity index of herbicides in the conditions of freezing stress showed that this index could be well used to determine the selective performance of these herbicides. Overall, according to the results of this experiment, it can be stated that FAS

with ACCase inhibitor herbicides, such as clodinafop-propargyl, may reduce their effectiveness in controlling narrow leaf weeds such as winter wild oat, and FBS with auxin type herbicides, such as 2,4-D plus MCPA, reduce their efficacy in controlling broadleaf weeds such as turnipweed. FBS for clodinafop-propargyl and FAS for 2,4-D plus MCPA had a slight adverse effect on the herbicide efficacy and selectivity index. Therefore, the use of these herbicides in the mentioned conditions (FBS for clodinafop-propargyl and FAS for 2,4-D plus MCPA) is recommended. In other words, to maintain high herbicide efficacy under cold (freezing) conditions, the use of clodinafop-propargyl after the freezing stress and 2,4-D plus MCPA before the freezing stress can be recommended in areas with a similar climate to Mashhad. Finally, the use of adjuvants, alternate herbicides, and various herbicide formulations to control grass and broadleaf weeds in Iranian wheat production is suggested to prevent the adverse effects of freezing stress on herbicides.

Conflicts of interest

The authors declare that they have no conflict of interest.

Acknowledgment

We are grateful to Ferdowsi University of Mashhad, Iran, for providing financial support for present research (Project No.3.49759). No additional external funding was received for this study.

References

1. Hatami ZM, Gherekhloo J, Rojano-Delgado AM, Osuna MD, Alcántara R, Fernández P, et al. Multiple mechanisms increase levels of resistance in *Rapistrum rugosum* to ALS herbicides. Front Plant Sci. 2016 Feb 22;7:169. https://doi.org/10.3389/fpls.2016.00169
2. Nosratti I, Sabeti P, Chaghamirzaee G, Heidari H. Weed problems, challenges, and opportunities in Iran. Crop Prot. 2020 Aug 1;134:104371. https://doi.org/10.1016/j.cropro.2017.10.007
3. Hasanfard A, Rastgoo M, Darbandi EI, Nezami A, Chauhan BS. Regeneration capacity after exposure to freezing in wild oat (*Avena ludoviciana* Durieu.) and turnipweed

(*Rapistrum rugosum* (L.) All.) in comparison with winter wheat. Environ. Exp. Bot. 2021 Jan 1;181:104271. https://doi.org/10.1016/j.envexpbot.2020.104271

4. Sierra-Almeida A, Cavieres LA, Bravo LA. Warmer temperatures affect the in situ freezing resistance of the Antarctic vascular plants. Front. Plant Sci. 2018 Oct 8;9:1456. https://doi.org/10.3389/fpls.2018.01456
5. Willick IR, Tanino KK, Gusta LV. The impact of global climate change on the freezing tolerance of winter cereals in Western Canada. J. Agron. Crop Sci. 2021 Feb;207(1):88-99. https://doi.org/10.1111/jac.12447
6. Vyse K, Pagter M, Zuther E, Hincha DK. Deacclimation after cold acclimation—a crucial, but widely neglected part of plant winter survival. J. Exp. Bot. 2019 Sep 15;70(18):4595-604. https://doi.org/10.1093/jxb/erz229
7. Cici SZ, Van Acker RC. Relative freezing tolerance of facultative winter annual weeds. Can. J. Plant Sci. 2011;91(4):759-63. https://doi.org/10.4141/cjps2010-001
8. Baghestani MA, Zand E, Soufizadeh S, Beheshtian M, Haghighi A, Barjasteh A, Birgani DG, Deihimfard R. Study on the efficacy of weed control in wheat (*Triticum aestivum* L.) with tank mixtures of grass herbicides with broadleaved herbicides. Crop Prot. 2008 Jan 1;27(1):104-11. https://doi.org/10.1016/j.cropro.2007.04.013
9. Enghiad A, Ufer D, Countryman AM, Thilmany DD. An overview of global wheat market fundamentals in an era of climate concerns. Int J Agron. 2017 Jul 2;2017. https://doi.org/10.1155/2017/3931897
10. Bajwa AA, Akhter MJ, Iqbal N, Peerzada AM, Hanif Z, Manalil S, Hashim S, Ali HH, Kebaso L, Frimpong D, Namubiru H. Biology and management of Avena fatua and Avena ludoviciana: two noxious weed species of agro-ecosystems. Environ Sci Pollut Res. 2017 Aug;24(24):19465-79. https://doi.org/10.1007/s11356-017-9810-y
11. Peterson MA, McMaster SA, Riechers DE, Skelton J, Stahlman PW. 2, 4-D past, present, and future: a review. Weed Technol. 2016 Jun;30(2):303-45. https://doi.org/10.1614/WT-D-15-00131.1
12. Collings LV, Blair AM, Gay AP, Dyer CJ, Mackay N. The effect of weather factors on the performance of herbicides to control *Alopecurus myosuroides* in winter wheat. Weed Res. 2003 Apr;43(2):146-53. https://doi.org/10.1046/j.1365-3180.2003.00327.x
13. Rocha-Pereira MR, Klar AE, Martins D, Ferreira de Souza GS, Villalba J. Efecto del estrés hídrico sobre la eficacia de los herbicidas aplicados en *Urochloa decumbens*. Cienc. Investig Agrar. 2012 Apr;39(1):211-20. http://dx.doi.org/10.4067/S0718-16202012000100018
14. Johnson BC, Young BG. Influence of temperature and relative humidity on the foliar activity of mesotrione. Weed Sci. 2002 Apr;50(2):157-61. https://doi.org/10.1614/0043-1745(2002)050[0157:IOTARH]2.0.CO;2.
15. Chahal PS, Aulakh JS, Rosenbaum K, Jhala AJ. Growth stage affects dose response of selected glyphosate-resistant weeds to premix of 2,4-D choline and glyphosate (Enlist Duo (TM) herbicide*). J. Agric. Sci. 2015 Nov 1;7(11):1. https://doi.org/10.5539/jas.v7n11p1
16. Waltz AL, Martin AR, Roeth FW, Lindquist JL. Glyphosate efficacy on velvetleaf varies with application time of day. Weed Technol. 2004 Dec;18(4):931-9. https://doi.org/10.1614/WT-03-123R3

17. Godar AS, Varanasi VK, Nakka S, Prasad PV, Thompson CR, Mithila J. Physiological and molecular mechanisms of differential sensitivity of Palmer amaranth (*Amaranthus palmeri*) to mesotrione at varying growth temperatures. Plos One. 2015 May 19;10(5):e0126731. https://doi.org/10.1371/journal.pone.0126731
18. Pline WA, Wu J, Hatzios KK. Effects of temperature and chemical additives on the response of transgenic herbicide-resistant soybeans to glufosinate and glyphosate applications. Pestic. Biochem. Phys. 1999 Oct 1;65(2):119-31. https://doi.org/10.1006/pest.1999.2437
19. Saini RK, Malone J, Preston C, Gill GS. Frost reduces clethodim efficacy in clethodim-resistant rigid ryegrass (*Lolium rigidum*) populations. Weed Sci. 2016 Jun;64(2):207-15. https://doi.org/10.1614/WS-D-15-00140.1
20. Moseley C, Hatzios KK, Hagood ES. Uptake, translocation, and metabolism of chlorimuron in soybean (*Glycine max*) and morningglory (*Ipomoea* spp.). Weed Tech. 1993 Jun;7(2):343-8. https://doi.org/10.1017/S0890037X00027706
21. Negrisoli E, Velini ED, Tofoli GR, Cavenaghi AL, Martins D, Morelli JL, Costa AG. Selectivity of pre-emergence herbicides to sugarcane treated with nematicides. Planta Daninha. 2004 Dec;22(4):567-75.
22. Powles SB, Lorraine-Colwill DF, Dellow JJ, Preston C. Evolved resistance to glyphosate in rigid ryegrass (*Lolium rigidum*) in Australia. Weed Sci. 1998 Oct;46(5):604-7.
23. Ritz C, Baty F, Streibig JC, Gerhard D. Dose-response analysis using R. Plos One. 2015 Dec 30;10(12):e0146021. https://doi.org/10.1371/journal.pone.0146021
24. Tind T, Mathiesen TJ, Jensen JE, Ritz C, Streibig JC. Using a selectivity index to evaluate logarithmic spraying in grass seed crops. Pest Manag Sci: formerly Pesticide Science. 2009 Nov;65(11):1257-62. https://doi.org/10.1002/ps.1819
25. Team RC. R Foundation for Statistical Computing, Vienna, available at: www. R-project. org. 2018.
26. Kowalczyk B, Caseley JC, McCready CC. The effect of pre-treatment temperature on some physiological responses of *Avena fatua* to difenzoquat and benzoylpropethyl. Asp Appl Biol. 1983;4:235-44.
27. Coupland D. Influence of environmental factors on the performance of sethoxydim against *Elymus repens* (L.). Weed Res. 1987 Oct;27(5):329-36. https://doi.org/10.1111/j.1365-3180.1987.tb00770.x
28. Derr J, Serensits T. Impact of application temperature on broadleaf herbicide efficacy. J Environ Hortic. 2016 Dec;34(4):123-30. https://doi.org/10.24266/0738-2898-34.4.123
29. Ganie ZA, Jugulam M, Jhala AJ. Temperature influences efficacy, absorption, and translocation of 2,4-D or glyphosate in glyphosate-resistant and glyphosate-susceptible common ragweed (*Ambrosia artemisiifolia*) and giant ragweed (*Ambrosia trifida*). Weed Sci. 2017 Sep;65(5):588-602. https://doi.org/10.1017/wsc.2017.32
30. Kumaratilake AR, Preston C. Low temperature reduces glufosinate activity and translocation in wild radish (*Raphanus raphanistrum*). Weed Sci. 2005 Feb;53(1):10-6. https://doi.org/10.1614/WS-03-140R

Manuscript
Click here to access/download;Manuscript;Revised Manuscript (4).docx

Freezing stress affects the efficacy of clodinafop-propargyl and 2,4-D plus MCPA on wild oat (*Avena ludoviciana* Durieu) and turnipweed [*Rapistrum rugosum* (L.) All.] in wheat (*Triticum aestivum* L.)

Short running title: Freezing and herbicide efficacy

Alireza Hasanfard[1], Mehdi Rastgoo[1], Ebrahim Izadi Darbandi[1], Ahmad Nezami[1], Bhagirath Singh Chauhan[2]

[1] Department of Agrotechnology, Faculty of Agriculture, Ferdowsi University of Mashhad, Iran

[2] Queensland Alliance for Agriculture and Food Innovation (QAAFI) *and* School of Agriculture and Food Sciences (SAFS), The University of Queensland, Gatton, Queensland 4343, Australia

***Corresponding author:** m.rastgoo@um.ac.ir

Abstract

The occurrence of freezing stress around herbicides application is one of the most important factors influencing their performance. This experiment was performed to evaluate the efficacy of clodinafop-propargyl and 2,4-D plus MCPA (2,4-Dichlorophenoxyacetic acid plus 2-methyl-4-chlorophenoxyacetic acid), the most important herbicides used in wheat fields in Iran, under the influence of a freezing treatment (-4℃). Wheat and its two common weeds, winter wild oat (*Avena ludoviciana* Durieu) and turnipweed [*Rapistrum rugosum* (L.) All.], were exposed to the freezing treatment for three nights from 7:00 P.M. to 5:00 A.M. before and after herbicide application, and their response was compared with plants that did not grow under freezing stress. Under no freezing (NF) and freezing after spray (FAS) conditions, winter wild oat was completely controlled with the recommended dose of clodinafop-propargyl (64 g ai ha^{-1}; hereafter g ha^{-1}). However, the survival percentage of winter wild oat in the freezing before spray (FBS) of clodinafop-propargyl 64 g ha^{-1} was 7%, and it was completely controlled with twice the recommended dose (128 g ha^{-1}). Under NF conditions and FAS treatment, turnipweed was completely controlled with twice the recommended dose of 2,4-D plus MCPA (2025 g ae ha^{-1}; hereafter g ha^{-1}), while there was no complete control under recommended rate. However, in the FBS treatment, the survival of turnipweed was 7% under double dose. The LD_{50} (dose required to control 50% of individuals in the population) and GR_{50} (dose causing 50% growth reduction of plants) rankings were NF<FBS<FAS for clodinafop-propargyl and NF<FAS<FBS for 2,4-D plus MCPA. Selectivity index for clodinafop-propargyl in NF conditions, FBS, and FAS treatments was 2.4, 0.91, and 0.78, respectively, and, for 2,4-D plus MCPA, it was 2.6, 0.12, and 0.88, respectively. According to the results of LD_{50}, it can be stated that the freezing stress after

the spraying of clodinafop-propargyl and before the spraying of 2,4-D plus MCPA would further reduce the efficacy of these herbicides.

Keywords: GR_{50}, Herbicide efficacy, LD_{50}, Selectivity index

Introduction

Winter wild oat (*Avena ludoviciana* Durieu) and turnipweed [*Rapistrum rugosum* (L.) All.] are the most important annual weeds of winter wheat (*Triticum aestivum* L.) in Iran, which cause significant reductions in wheat yield [1, 2]. The high reproduction potential and fitness to freezing stress, which results in a wide dispersal of weed seeds are the major challenges in the management of winter annual weeds like wild oat and turnipweed. Therefore, in cold regions of Iran, the dispersal and geographical distribution of these weeds are more significant than other weed species [3].

One of the most important consequences of climate change is the change in glaciers' occurrence patterns [4]. A sharp drop in temperature after high temperatures leads to crop damage. Increasing autumn temperatures in Iran, resulting from climate change, have led to reduced freezing tolerance of autumn crops [3]. In other words, autumn crops will not tolerate freezing stress due to the lack of hardening process in autumn. As a result, they will be damaged by decreasing temperatures during winter, and their yield will be reduced [3, 4]. It has been reported that increasing autumn temperatures resulting from climate change in western Canada led to reduced freezing tolerance of winter cereals [5]. In other words, due to the lack of optimal cold acclimation in autumn, crops will not be able to tolerate freezing stress. As a result, they will be damaged by decreasing temperatures during winter or by the occurrence of late spring frosts [6]. However, weeds generally have a higher ability to tolerate freezing stress [7]. Based

on this fact, it can be inferred that climate change is adversely changing agricultural production and can significantly change the management of current cropping systems that have evolved under optimum climatic conditions.

Hand weeding and hoeing are not common in wheat fields, and mechanical weed management methods are not effective, so chemical methods are mainly used to control weeds in the wheat fields in Iran. Clodinafop-propargyl and 2,4-D plus MCPA (2,4-Dichlorophenoxyacetic acid plus 2-methyl-4-chlorophenoxyacetic acid) are the most common herbicides used for controlling weeds in wheat fields in Iran. Due to the particular importance of this crop in food security, their wide excessive applications can be justified [8, 9]. Clodinafop-propargyl is a systemic and selective herbicide of the aryloxyphenoxypropionate (APP) group and used post-emergent to control annual grass weeds in wheat. This herbicide inhibits the activity of acetyl-coenzyme A carboxylase (ACCase) and thus disrupts the biosynthesis of fatty acids [10]. 2,4-D plus MCPA, synthetic auxin herbicides, is a systemic herbicide mixture registered for selective post-emergence control of annual broadleaf weeds in wheat [11].

The efficacy of herbicides is significantly affected by environmental conditions before, during, and after application [12]. It has been reported that environmental factors such as light intensity, temperature, water stress, relative humidity, and nutrient availability alter the herbicide efficacy [13, 14]. On the other hand, plant species and their growth stages also affect herbicide efficacy [15]. Numerous studies indicate that ambient temperature is one of the most influential factors affecting the performance of herbicides [14, 16, 17]. Increasing the temperature is likely to increase the absorption and translocation of some herbicides and increase their performance. In contrast, the improvement in herbicide performance at low temperatures may be due to a reduction in the rate of herbicide metabolism [17, 18]. In cold regions, freezing temperatures

occur around the time of herbicide application. Therefore, the occurrence of severe freezing that causes damage to plant leaves will have adverse effects on the activity and efficacy of foliar-applied herbicides [19]. On the other hand, application of herbicides in unfavorable environmental conditions (such as adverse temperature before, during, and after herbicide application) causes stress in the crop, and this changes the selectivity index of the herbicide, which will ultimately cause damage to the crop [20]. The selectivity index refers to the potential of a herbicide to eliminate weeds in a crop without affecting the yield or quality of the final product [21].

At the appropriate growth stage of weeds for herbicide application, the temperature usually decreases, and this issue leads to a decrease in the performance of herbicides. Due to the lack of information on the efficacy of herbicides under freezing stress, this experiment was conducted to evaluate the efficacy of clodinafop-propargyl and 2,4-D plus MCPA on wild oat and turnipweed under freezing stress before and after herbicide application.

Materials and methods

Experimental site and plant material

Seeds of wheat (cv. Mihan) were harvested from Khorasan Razavi Agricultural and Natural Resources Research and Education Center, and seeds of winter wild oat and turnipweed were collected (collected from about 500 plants) from infested farms around Mashhad-Iran in May 2019. Experiments were conducted at the Faculty of Agriculture, the Ferdowsi University of Mashhad, Iran (Lat 36° 15′ N, Long 59° 28 E; 985 m Altitude). To break the dormancy of winter wild oat and turnipweed, their seeds were exposed to moist-chilling treatment and potassium nitrate (KNO_3) 0.2% solution for 3 days at 5℃ in the dark, respectively [3]. Weed seeds were

placed in 9-cm diameter Petri dishes (20-25 seeds per Petri dish) on moist filter papers and kept at 20℃. After 5 days, the germination percentage of winter wild oat and turnipweed seeds was >90%. The experiment was repeated twice with three replicates. To experience the same cold acclimation, the plants were planted at approximately the same time on October 21 (first run) and 24 (second run) in 2019. Plastic pots (12 cm in diameter and 20 cm in height) were used in the trial, and pots were filled with a mixture of sand, agricultural soil, and peat moss (2:2:1 sand, agricultural soil, and peat moss, respectively). For uniform emergence, turnipweed germinated seeds were planted in a seedling tray. Eight turnipweed seedlings were transferred to pots in the cotyledon stage. Eight germinated seeds of wild oat were planted at 4-5 cm depth below the soil surface. Wheat seeds were planted directly in each pot at the same depth. To induce cold acclimation, the plants were kept outdoors, grown in natural conditions (Fig. 1), and irrigated according to daily requirements until they were tested.

Fig 1. Minimum and maximum daily air temperatures at the experimental site from October 2019 to February 2020. Relative humidity of 40-50% under the natural photoperiod [3].

Experimental factors

Experimental factors included freezing stress at three conditions, including no freezing (NF), freezing before spray (FBS), and freezing after spray (FAS). Plant species included wheat, winter wild oat, and turnipweed. Herbicides were applied at 0, 6.25, 12.5, 25, 50, 100, and 200% of the recommended dose for the herbicide clodinafop-propargyl (0.8 L ha^{-1}; Topik, 8% EC) and 2,4-D plus MCPA (1.5 L ha^{-1}; U 46 combi fluid, 67.5% SL).

Freezing treatment and herbicide application

Under NF, the plants grew completely in natural winter conditions without exposing to freezing stress. For FBS, the plants grew up to the 3-4 leaf stage under natural conditions. They were exposed to freezing from 7:00 P.M. to 5:00 A.M. for three nights before herbicide application [19]. Freezing treatment was performed using a thermogradient freezer (Weiss Technik). The initial temperature of the freezer was 5℃; however, the freezer was cooled down at a rate of 2℃ per hour to reach a temperature of -4℃. The temperature drop slope was adjusted based on the long-term meteorological statistics of Mashhad in the time range of herbicide application. The pots were kept at -4℃ for 1 h and then removed from the freezer (Fig. 2).

Fig. 2 Trend of temperature decrease in thermogradient freezer during the night from 7:00 P.M. to 5:00 A.M.

Each morning at 5:30 A.M., the plants were transferred out of the freezer, kept in natural conditions until 6:30 P.M., and then returned to the freezer during the night. After three nights of freezing exposure, the pots were taken out of the freezer and sprayed with herbicides after 2 h [19]. After spraying, the plants were kept outside under natural conditions for the duration of the experiment.

In the FAS treatment, freezing stress was applied for three nights by placing the samples treated with herbicides in the thermogradient freezer. For this purpose, the plants were taken out of the freezer at 5:30 A.M., transferred to natural conditions, and then returned to the freezer at 6:30 P.M. for the overnight freezing treatment (Similar to FBS treatment). Three nights after freezing treatment, the plants were transferred to natural conditions to continue the trial process (Fig. 3).

Fig. 3 Schematic diagram of the experimental study design. Abbreviation: FBS (freezing before spray); FAS (freezing after spray).

The conditions inside the thermogradient freezer were the same for both freezing treatments before and after spray. The experiment was performed in the freezer in completely dark conditions and at the same relative humidity (35±5%) for all treatments. Temperature changes in the freezer were regularly monitored when the samples were in the freezer. For all the freezing treatments, herbicides were applied at the same time. Plants were transferred to natural conditions after three nights of freezing treatment at 5:30 A.M. Also, plants related to freezing after spray were transferred to the freezer at 6:30 P.M. On the same day, plants that were not exposed to freezing were sprayed from 7:30 A.M. to 10.30 A.M. The herbicides were applied by a researcher-made moving boom sprayer equipped with a TeeJet 8002 flat fan nozzle (spray output volume of 200 L ha^{-1} at a pressure of 260 kPa). Plants were returned and maintained outdoor under natural conditions of winter after the treatment.

Measurement of traits

Four weeks after herbicide treatments, survival percentage (Eq. 1) and shoot dry weight were measured by placing the samples in an oven at 75℃ for 48 h. Plants that produced new leaves were considered alive and those that experienced severe chlorosis and stunted growth were recorded as dead [22].

$$Survival\ \% = \frac{N1}{N0} \times 100 \qquad \text{(Eq. 1)}$$

Where, N_1 and N_0 are the numbers of alive plants four weeks after herbicide treatment and the number of alive plants before herbicide treatment, respectively.

Dose–response experiments

The response of weed traits to the doses of herbicides tested was analyzed by nonlinear regression. For this purpose, the data were fitted to a three-parameter log-logistic equation (Eq. 2) [23].

$$Y = \frac{d}{1+e^{b(\log x - \log e)}} \quad \text{(Eq. 2)}$$

Where, Y is the response rate (survival % or dry weight) at dose x, d is the upper limit for Y, b is the slope of the curve around e, and e is the effective dose (g ai or ae ha^{-1}). Here, e is replaceable with LD_{50} (dose required to control 50% of individuals in the population) or GR_{50} (dose causing 50% dry weight reduction of plants).

Relative potency

Relative potency was used to determine herbicide efficacy under freezing stress and no freezing stress (Eq. 3) [24].

$$Relative\ potency = \frac{ED_{50A}}{ED_{50B}} \quad \text{(Eq. 3)}$$

Where, ED_{50A} is the dose required to reduce survival by 50% in NF, and ED_{50B} is the dose needed to reduce survival by 50% in FBS or FAS. Relative potency = 1 indicates that freezing treatment (FBS and FAS) did not affect herbicide efficacy. If relative potency > 1, it indicates that the herbicides are more efficient under freezing stress. Relative potency < 1 indicates the decreased effectiveness of the herbicides under freezing stress with respect to optimum conditions.

Selectivity index

The ratio between doses that caused 10% damage to crops and 90% damage to weeds was considered a selectivity index [24] and calculated using Eq. 4.

$$Selectivity\ index\ (10{,}90) = \frac{ED_{10\ (crop)}}{ED_{90\ (weed)}} \qquad \text{(Eq. 4)}$$

Where, ED_{90} is 90% of the herbicide effect on weed survival (herbicide dose to kill 90% of the population), and ED_{10} is 10% of the herbicide impact on crop survival (a 10% effect on the crop). The higher the SI, the more selective the herbicide in the crop [24].

Statistical analysis

The experiments were conducted using a completely randomized design in factorial arrangement with three replications. Afterward, differences between treatments were compared with the standard error of mean (SEM) values. For data analysis, analysis of variance (ANOVA) was performed using the SAS software (v. 9.4, SAS Institute Inc, Cary, NC, USA). Data for both runs were pooled as no significant differences were observed between runs ($P > 0.05$). Homogeneity of variance was confirmed using Levene's test. Analysis of biometric data was performed by the R program (v. 4.0.4) [25]. Dose-response curves were also fitted with the asymmetrical sigmoid form. LD_{50} and GR_{50} were separated using 95% confidence intervals (CI).

Results

Clodinafop-propargyl

Survival percentage and LD_{50}

The dose-response experiments were similar for NF and FAS treatments at the recommended dose (Figure 4A). Thus, in both conditions, winter wild oat was completely controlled with the recommended dose (64 g ai ha^{-1}). However, the survival of seedlings in the FBS treatment was 7%, and to achieve complete control, 2-fold the recommended dose (128 g ha^{-1}) was needed. The LD_{50} in NF, FBS, and FAS was 17.9, 18.1, and 23.2 g ha^{-1}, respectively (Table 1).

Fig. 4 Survival (A) and dry weight (B) of winter wild oat treated with various doses of clodinafop-propargyl under different freezing treatments. Abbreviation: NF (no freezing); FBS (freezing before spray): FAS (freezing after spray). The recommended dose of clodinafop-propargyl in Iran is 64 g ha^{-1}.

Table 1 The dose of clodinafop-propargyl and 2,4-D plus MCPA required for 50% mortality (LD_{50}) and the dose that caused 50% inhibition of growth noted by biomass (GR_{50}) of winter wild oat and turnipweed under different freezing treatments.

Herbicide	Freezing Treatment	b±SE	d±SE	LD_{50} ±SE	95% CI	R^2
			%	g ai or ae ha^{-1}		
Clodinafop-propargyl	NF	2.0 ± 0.19**	100.3 ± 2.8**	17.9 ± 1.2**b	15.6-20.2	0.99
	FBS	1.9 ± 0.17**	101.0 ± 2.9**	18.1 ± 1.2**b	15.7-20.5	0.99
	FAS	1.9 ± 0.19**	98.7 ± 2.9**	23.2 ± 1.7**a	19.8-26.6	0.96
2,4-D plus MCPA	NF	1.9 ± 0.20**	102.2 ± 3.2**	292.8 ± 21.9**c	249.4-336.3	0.98
	FBS	1.5 ± 0.15**	102.4 ± 3.2**	472.8 ± 42.4**a	388.8-556.8	0.98
	FAS	1.5 ± 0.14**	102.8 ± 3.5**	318.8 ± 28.9**b	261.6-376.1	0.98
		b±SE	d±SE	GR_{50} ±SE	95% CI	
			g pot^{-1}	g ai or ae ha^{-1}		
Clodinafop-propargyl	NF	2.1 ± 0.15**	0.70 ± 0.02**	10.6 ± 0.52**c	9.6-11.6	0.99
	FBS	2.0 ± 0.12**	0.80 ± 0.02**	11.6 ± 0.51**b	10.6-12.6	0.99
	FAS	1.7 ± 0.12**	0.67 ± 0.02**	12.8 ± 0.74**a	11.3-14.3	0.98
2,4-D plus MCPA	NF	2.2 ± 0.23**	0.46 ± 0.01**	176.2 ± 11.3**c	153.9-198.6	0.99
	FBS	1.7 ± 0.16**	0.48 ± 0.01**	298.6 ± 21.5**a	256.0-341.3	0.99
	FAS	1.6 ± 0.15**	0.47 ± 0.01**	207.8 ± 15.9**b	176.2-239.4	0.99

Abbreviation: NF (no freezing); FBS (freezing before spray); FAS (freezing after spray); SE: Standard Error. b is the slope of the curve around e (LD_{50} and GR_{50}); d is the upper limit for Y (survival % or dry weight); **: significant at p≤0.01. LD_{50} and GR_{50} were separated using their 95% confidence intervals (CI). The data shows the parameter's mean ± SE. The data (LD_{50} and GR_{50}) with the different letters indicate significant differences according to LSD at P ≤0.05. n = 6

Dry weight and GR_{50}

The dry weight of winter wild oat in NF and FAS with the recommended dose (64 g ha^{-1}) and FBS with twice the recommended dose (128 g ha^{-1}) reached 0 g (Fig. 4B). The GR_{50} of plants not exposed to freezing stress was 10.6 g ha^{-1}. The GR_{50} was 11.6 and 12.8 g ha^{-1} in FBS and FAS, respectively (Table 1).

2,4-D plus MCPA

Survival percentage and LD_{50}

The dose-response experiments of 2,4-D plus MCPA (similar to clodinafop-propargyl) were the same in NF and FAS treatments (Fig. 5A). In both conditions, turnipweed was completely controlled with twice the recommended dose (2025 g ae ha^{-1}). However, in the FBS treatment, the survival of turnipweed was 7%. At the recommended dose (1012.5 g ha^{-1}), the survival of turnipweed in NF, FBS, and FAS was 3%, 33%, and 20%, respectively. The LD_{50} in NF, FBS, and FAS was 293, 473, and 319 g ha^{-1}, respectively (Table 1).

Fig. 5 Survival (A) and dry weight (B) of turnipweed treated with various doses of 2,4-D plus MCPA under different freezing treatments. Abbreviation: NF (no freezing); FBS (freezing before spray): FAS (freezing after spray). The recommended dose of 2,4-D+MCPA in Iran is 1012.5 g ha^{-1}.

Dry weight and GR_{50}

The dry weight of turnipweed in NF and FAS with twice the recommended dose (2025 g ha^{-1}) of 2,4-D plus MCPA was reduced to 0 (Fig. 5 B). However, in the FBS treatment, the dry weight of turnipweed with twice the recommended dose was 0.012 g per pot. At the recommended dose, the dry weight of turnipweed in FBS was 97% and 76% more than NF and FAS, respectively. The GR_{50} of NF, FBS, and FAS were 176, 299, and 208 g ha^{-1}, respectively (Table 1).

Relative potency

The relative potency of clodinafop-propargyl and 2,4-D plus MCPA was significantly reduced in FBS and FAS compared to NF (Fig. 6). Thus, the relative potency in FBS and FAS of clodinafop-propargyl was 0.99 and 0.77, respectively, and at 2,4-D plus MCPA was 0.62 and 0.92, respectively.

Fig. 6 Relative potency of clodinafop-propargyl and 2,4-D plus MCPA under different freezing treatments. Abbreviation: FBS (freezing before spray) and FAS (freezing after spray). The error bars indicate standard error (SE). The dashed line represents the relative potency = 1.0.

Selectivity index

As shown in Table 2, selectivity index values for clodinafop-propargyl in NF, FBS, and FAS treatments were 2.4, 0.91, and 0.78, respectively, and, for 2,4-D plus MCPA, they were 2.6, 0.12, and 0.88, respectively. For clodinafop-propargyl, the LD_{10} of wheat in NF was 127.9 g ha^{-1}. However, LD_{10} of wheat in the FBS and FAS was 53.5 and 57.1 g ha^{-1}, respectively (Table 2). For 2,4-D plus MCPA, the LD_{10} of wheat in NF, FBS, and FAS was 2380, 264, and 1236 g ha^{-1}, respectively.

Table 2 Sensitivity of plant species of wheat, winter wild oat, and turnipweed to clodinafop-propargyl and 2,4-D plus MCPA under different freezing treatments.

Freezing Treatment	Plant species	Clodinafop-propargyl: LD_{10} (wheat) and LD_{90} (winter wild oat) (g ai ha^{-1}) ± SE	Clodinafop-propargyl: SI	2,4-D plus MCPA: LD_{10} (wheat) and LD_{90} (turnipweed) (g ae ha^{-1}) ± SE	2,4-D plus MCPA: SI
NF	Wheat	127.9 ± 8.8	-	2380 ± 455	-
	Weed	53.5 ± 5.4	2.4	908 ± 108	2.6
FBS	Wheat	53.5 ± 11.0	-	264 ± 61.0	-
	Weed	59.0 ± 6.4	0.91	2144 ± 327	0.12
FAS	Wheat	57.1 ± 8.7	-	1236 ± 163	-
	Weed	73.6 ± 7.2	0.78	1400 ± 199	0.88

Abbreviation: NF; No freezing, FBS: Freezing before spray, FAS: Freezing after spray SI: Selectivity index. The selectivity index (SI) was calculated as follows: *Selectivity index (10, 90):* $\frac{ED10\ (crop)}{ED90\ (weed)}$

Discussion

The required dose of clodinafop-propargyl for complete control of winter wild oat seedlings in FBS treatment was twice that of NF and FAS treatments (Fig. 4, A). The trend of the reducing survival % in seedlings related to the FBS treatment and NF was similar. The LD_{50} for clodinafop-propargyl in freezing treatments shows that exposure of winter wild oat seedlings to freezing treatment for three nights before and after spray reduces the efficacy of this herbicide compared to NF. The LD_{50} was 22% and 23% higher in the FAS compared to the FBS and NF, respectively (Table 1). Freezing treatment for three nights before and after spraying of clethodim herbicide (the mode of action similar to clodinafop-propargyl) led to an increase in LD_{50} [19]. It decreased herbicide efficacy in rigid ryegrass (*Lolium rigidum* Gaud.) populations compared to NF.

The dose of clodinafop-propargyl required to reduce winter wild oat biomass to zero in the FBS treatment was twice that of the NF and FAS treatments (Fig. 4, B). The GR_{50} is more pronounced in FBS and FAS compared to NF, indicating a decrease in the efficacy of clodinafop-propargyl. The GR_{50} was 17% and 9% higher in the FAS treatment compared to NF and FBS, respectively. Hence, according to the results of GR_{50}, three nights of freezing stress after herbicide application reduced the performance of clodinafop-propargyl more than other treatments (Table 1). It has been reported that wild oat plants (*Avena fatua*) that grew at low temperatures were less sensitive to difenzoquat than plants that grew at high temperatures [26].

Our results clearly show that the occurrence of freezing stress around spray reduces the efficacy of clodinafop-propargyl, especially in the treatment of FAS. Herbicide translocation in the phloem in freezing conditions could decline due to damage to the phloem cells, which may lead to a decrease in the herbicide efficacy. Decreased efficacy of sethoxydim at low

temperatures due to reduced herbicide translocation for Quackgrass [*Elymus repens* (L.) Gould] control was reported by Coupland [27]. In general, to maintain the herbicide efficacy under cold conditions, using clodinafop-propargyl after the freezing stress can be recommended in areas with a similar climate.

Although the three-night treatment of FBS and FAS resulted in a reduction in turnipweed control by 2,4-D plus MCPA compared to NF, this reduction was different in the two freezing treatments (Fig. 5, A). At the recommended dose (1013 g ha^{-1}) in NF, almost all turnipweed plants were controlled, while the survival percentage of turnipweed in FBS and FAS treatments were 33% and 20%, respectively. Low temperatures around the time of herbicide application reduced the effectiveness of systemic herbicides [28]. Based on LD_{50} values, the efficacy of 2,4-D plus MCPA under FBS and FAS was 1.6 and 1.1 times lower compared to NF, respectively (Table 1).

FAS is possible to damage the phloem cells, which reduces the translocation of the herbicide. The efficacy of 2,4-D plus MCPA in the treatment of FBS was reduced. The decrease in the effect of 2,4-D plus MCPA in the treatment of FBS is probably due to the reduction of absorption and translocation of this herbicide in turnipweed seedlings. In this regard, an increase in efficacy of 2,4-D and glyphosate at high temperatures compared to low temperatures in common ragweed (*Ambrosia artemisiifolia* L.) and giant ragweed (*Ambrosia trifida* L.) due to the increased absorption and/or translocation at high temperatures around herbicide application has been reported by Ganie et al. [29]. It has been also reported that low temperatures reduced glufosinate activity and translocation in wild radish (*Raphanus raphanistrum* L.) [30].

Turnipweed's dry weight at the recommended dose of 2,4-D plus MCPA (1012.5 g ha^{-1}) in FBS and FAS treatments was 35.5 and 8.5 times higher than the NF, respectively (Fig. 5, B).

Also, GR_{50} in FBS and FAS was 41% and 15.2% higher compared to NF, respectively (Table 1). Although a decrease in herbicide efficacy is evident in both FBS and FAS treatments, high GR_{50} in FBS shows an adverse effect of three nights of freezing before the application of 2,4-D plus MCPA. In other words, the efficacy of this herbicide reacts more to FBS than to FAS. In a similar study, the GR_{50} for 2,4-D increased at low temperature in common ragweed compared to high temperature [29].

The freezing stress around 2,4-D plus MCPA application reduced the efficacy of this herbicide in FBS and FAS treatments compared to NF. The decrease in the efficacy of this herbicide in the treatment of FBS is probably due to the reduction of absorption and translocation of the herbicide in the phloem during freezing. Absorption and translocation of 2,4-D plus MCPA might be reduced at low temperatures due to the effect on herbicide penetration facilitated by physicochemical factors, including the decreased rate of diffusion, increased viscosity of the cuticle, and physiological factors comprising decreases in photosynthesis, phloem translocation, and protoplasmic streaming [29]. In Iranian wheat fields, using the 2,4-D plus MCPA before the freezing stress can be recommended to maintain the herbicide efficacy under cold conditions.

When relative potency > 1, the herbicide efficacy is higher than optimal non-freezing conditions, and when relative potency < 1, the herbicide efficacy is lower than usual [24]. The relative potency values of clodinafop-propargyl and 2,4-D plus MCPA were lower than one under both FBS and FAS treatments in the present experiment (Fig. 6). In other words, freezing stress reduced the efficacy of these herbicides. Based on the results of relative potency, the reduction of clodinafop-propargyl efficacy in the FAS treatment (33%) was more significant than

in the FBS treatment (1%). Conversely, for 2,4-D plus MCPA, the reduction in efficacy was more significant in the FBS treatment (38%) compared to the FAS treatment (8%).

The selectivity index for clodinafop-propargyl and 2,4-D plus MCPA was > 1 in NF, indicating that both herbicides acted selectively in wheat (Table 2). However, applying three nights of FBS and FAS of both herbicides reduced the selectivity index to less than one. In other words, freezing stress reduced the selectivity index of both herbicides, which led to phytotoxicity in wheat. For clodinafop-propargyl, LD_{10} of wheat in NF was 58% and 55% higher than FBS and FAS, respectively. The LD_{10} values of 2,4-D plus MCPA in NF were 89% and 48% higher compared to FBS and FAS, respectively. In other words, the dose required to kill 10% of wheat in both herbicides under NF was higher than FBS and FAS. Therefore, FBS and FAS increased the sensitivity of wheat to herbicide application. Freezing stress may reduce the rate of herbicide metabolism in the crop and ultimately increase its susceptibility [17].

Conclusions

The relative potency results for both herbicides were used to confirm the results of LD_{50} and GR_{50}. Thus, LD_{50} and GR_{50} were higher for clodinafop-propargyl in the FAS treatment than in NF and FBS treatments. The LD_{50} and GR_{50} were higher for the 2,4-D plus MCPA in the FBS treatment than in the NF and FAS treatments. In other words, clodinafop-propargyl was more effective than other conditions in the treatment of three nights of FAS and 2,4-D plus MCPA in the treatment of three nights of FBS. The selectivity index for clodinafop-propargyl was lower in the FAS treatment (0.78) and for 2,4-D plus MCPA was lower in the FBS treatment (0.12). The efficacy of clodinafop-propargyl in the FAS treatment and the efficacy of 2,4-D plus MCPA in the FBS treatment were reduced. The values of the selectivity index of herbicides in the

conditions of freezing stress showed that this index could be well used to determine the selective performance of these herbicides. Overall, according to the results of this experiment, it can be stated that FAS with ACCase inhibitor herbicides, such as clodinafop-propargyl, may reduce their effectiveness in controlling winter wild oat. FBS with auxin type herbicides, such as 2,4-D plus MCPA, reduce their efficacy in controlling turnipweed. FBS for clodinafop-propargyl and FAS for 2,4-D plus MCPA had a slight adverse effect on the herbicide efficacy and selectivity index. Finally, research on the use of adjuvants, alternate herbicides, and various herbicide formulations to control grass and broadleaf weeds in Iranian wheat production is suggested to prevent the adverse effects of freezing stress on herbicides.

Conflicts of interest

The authors declare that they have no conflict of interest.

Acknowledgment

We are grateful to Ferdowsi University of Mashhad, Iran, for providing financial support for present research (Project No.3.49759). No additional external funding was received for this study.

References

1. Hatami ZM, Gherekhloo J, Rojano-Delgado AM, Osuna MD, Alcántara R, Fernández P, et al. Multiple mechanisms increase levels of resistance in *Rapistrum rugosum* to ALS herbicides. Front Plant Sci. 2016 Feb 22;7:169. https://doi.org/10.3389/fpls.2016.00169
2. Nosratti I, Sabeti P, Chaghamirzaee G, Heidari H. Weed problems, challenges, and opportunities in Iran. Crop Prot. 2020 Aug 1;134:104371. https://doi.org/10.1016/j.cropro.2017.10.007
3. Hasanfard A, Rastgoo M, Darbandi EI, Nezami A, Chauhan BS. Regeneration capacity after exposure to freezing in wild oat (*Avena ludoviciana* Durieu.) and turnipweed (*Rapistrum rugosum* (L.) All.) in comparison with winter wheat. Environ. Exp. Bot. 2021 Jan 1;181:104271. https://doi.org/10.1016/j.envexpbot.2020.104271
4. Sierra-Almeida A, Cavieres LA, Bravo LA. Warmer temperatures affect the in situ freezing resistance of the Antarctic vascular plants. Front. Plant Sci. 2018 Oct 8;9:1456. https://doi.org/10.3389/fpls.2018.01456

5. Willick IR, Tanino KK, Gusta LV. The impact of global climate change on the freezing tolerance of winter cereals in Western Canada. J. Agron. Crop Sci. 2021 Feb;207(1):88-99. https://doi.org/10.1111/jac.12447
6. Vyse K, Pagter M, Zuther E, Hincha DK. Deacclimation after cold acclimation—a crucial, but widely neglected part of plant winter survival. J. Exp. Bot. 2019 Sep 15;70(18):4595-604. https://doi.org/10.1093/jxb/erz229
7. Cici SZ, Van Acker RC. Relative freezing tolerance of facultative winter annual weeds. Can. J. Plant Sci. 2011;91(4):759-63. https://doi.org/10.4141/cjps2010-001
8. Baghestani MA, Zand E, Soufizadeh S, Beheshtian M, Haghighi A, Barjasteh A, Birgani DG, Deihimfard R. Study on the efficacy of weed control in wheat (*Triticum aestivum* L.) with tank mixtures of grass herbicides with broadleaved herbicides. Crop Prot. 2008 Jan 1;27(1):104-11. https://doi.org/10.1016/j.cropro.2007.04.013
9. Enghiad A, Ufer D, Countryman AM, Thilmany DD. An overview of global wheat market fundamentals in an era of climate concerns. Int J Agron. 2017 Jul 2;2017. https://doi.org/10.1155/2017/3931897
10. Bajwa AA, Akhter MJ, Iqbal N, Peerzada AM, Hanif Z, Manalil S, Hashim S, Ali HH, Kebaso L, Frimpong D, Namubiru H. Biology and management of Avena fatua and Avena ludoviciana: two noxious weed species of agro-ecosystems. Environ Sci Pollut Res. 2017 Aug;24(24):19465-79. https://doi.org/10.1007/s11356-017-9810-y
11. Peterson MA, McMaster SA, Riechers DE, Skelton J, Stahlman PW. 2, 4-D past, present, and future: a review. Weed Technol. 2016 Jun;30(2):303-45. https://doi.org/10.1614/WT-D-15-00131.1
12. Collings LV, Blair AM, Gay AP, Dyer CJ, Mackay N. The effect of weather factors on the performance of herbicides to control *Alopecurus myosuroides* in winter wheat. Weed Res. 2003 Apr;43(2):146-53. https://doi.org/10.1046/j.1365-3180.2003.00327.x
13. Rocha-Pereira MR, Klar AE, Martins D, Ferreira de Souza GS, Villalba J. Efecto del estrés hídrico sobre la eficacia de los herbicidas aplicados en *Urochloa decumbens*. Cienc. Investig Agrar. 2012 Apr;39(1):211-20. http://dx.doi.org/10.4067/S0718-16202012000100018
14. Johnson BC, Young BG. Influence of temperature and relative humidity on the foliar activity of mesotrione. Weed Sci. 2002 Apr;50(2):157-61. https://doi.org/10.1614/0043-1745(2002)050[0157:IOTARH]2.0.CO;2.
15. Chahal PS, Aulakh JS, Rosenbaum K, Jhala AJ. Growth stage affects dose response of selected glyphosate-resistant weeds to premix of 2,4-D choline and glyphosate (Enlist Duo (TM) herbicide*). J. Agric. Sci. 2015 Nov 1;7(11):1. https://doi.org/10.5539/jas.v7n11p1
16. Waltz AL, Martin AR, Roeth FW, Lindquist JL. Glyphosate efficacy on velvetleaf varies with application time of day. Weed Technol. 2004 Dec;18(4):931-9. https://doi.org/10.1614/WT-03-123R3
17. Godar AS, Varanasi VK, Nakka S, Prasad PV, Thompson CR, Mithila J. Physiological and molecular mechanisms of differential sensitivity of Palmer amaranth (*Amaranthus palmeri*) to mesotrione at varying growth temperatures. Plos One. 2015 May 19;10(5):e0126731. https://doi.org/10.1371/journal.pone.0126731
18. Pline WA, Wu J, Hatzios KK. Effects of temperature and chemical additives on the response of transgenic herbicide-resistant soybeans to glufosinate and glyphosate

applications. Pestic. Biochem. Phys. 1999 Oct 1;65(2):119-31. https://doi.org/10.1006/pest.1999.2437

19. Saini RK, Malone J, Preston C, Gill GS. Frost reduces clethodim efficacy in clethodim-resistant rigid ryegrass (*Lolium rigidum*) populations. Weed Sci. 2016 Jun;64(2):207-15. https://doi.org/10.1614/WS-D-15-00140.1
20. Moseley C, Hatzios KK, Hagood ES. Uptake, translocation, and metabolism of chlorimuron in soybean (*Glycine max*) and morningglory (*Ipomoea* spp.). Weed Tech. 1993 Jun;7(2):343-8. https://doi.org/10.1017/S0890037X00027706
21. Negrisoli E, Velini ED, Tofoli GR, Cavenaghi AL, Martins D, Morelli JL, Costa AG. Selectivity of pre-emergence herbicides to sugarcane treated with nematicides. Planta Daninha. 2004 Dec;22(4):567-75.
22. Powles SB, Lorraine-Colwill DF, Dellow JJ, Preston C. Evolved resistance to glyphosate in rigid ryegrass (*Lolium rigidum*) in Australia. Weed Sci. 1998 Oct;46(5):604-7.
23. Ritz C, Baty F, Streibig JC, Gerhard D. Dose-response analysis using R. Plos One. 2015 Dec 30;10(12):e0146021. https://doi.org/10.1371/journal.pone.0146021
24. Tind T, Mathiesen TJ, Jensen JE, Ritz C, Streibig JC. Using a selectivity index to evaluate logarithmic spraying in grass seed crops. Pest Manag Sci: formerly Pesticide Science. 2009 Nov;65(11):1257-62. https://doi.org/10.1002/ps.1819
25. Team RC. R Foundation for Statistical Computing, Vienna, available at: www. R-project. org. 2018.
26. Kowalczyk B, Caseley JC, McCready CC. The effect of pre-treatment temperature on some physiological responses of *Avena fatua* to difenzoquat and benzoylpropethyl. Asp Appl Biol. 1983;4:235-44.
27. Coupland D. Influence of environmental factors on the performance of sethoxydim against *Elymus repens* (L.). Weed Res. 1987 Oct;27(5):329-36. https://doi.org/10.1111/j.1365-3180.1987.tb00770.x
28. Derr J, Serensits T. Impact of application temperature on broadleaf herbicide efficacy. J Environ Hortic. 2016 Dec;34(4):123-30. https://doi.org/10.24266/0738-2898-34.4.123
29. Ganie ZA, Jugulam M, Jhala AJ. Temperature influences efficacy, absorption, and translocation of 2,4-D or glyphosate in glyphosate-resistant and glyphosate-susceptible common ragweed (*Ambrosia artemisiifolia*) and giant ragweed (*Ambrosia trifida*). Weed Sci. 2017 Sep;65(5):588-602. https://doi.org/10.1017/wsc.2017.32
30. Kumaratilake AR, Preston C. Low temperature reduces glufosinate activity and translocation in wild radish (*Raphanus raphanistrum*). Weed Sci. 2005 Feb;53(1):10-6. https://doi.org/10.1614/WS-03-140R

Figure
Click here to access/download;Figure;Figures (4).docx

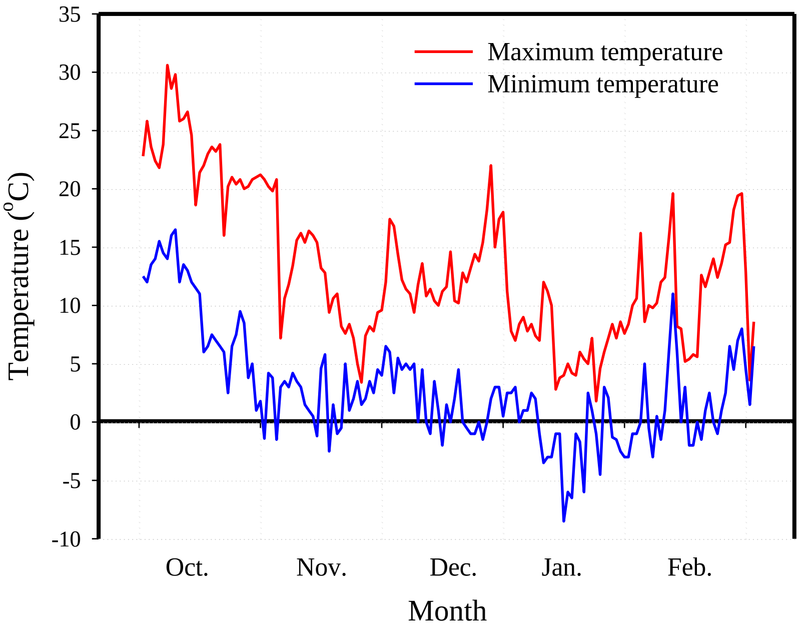

Fig 1.

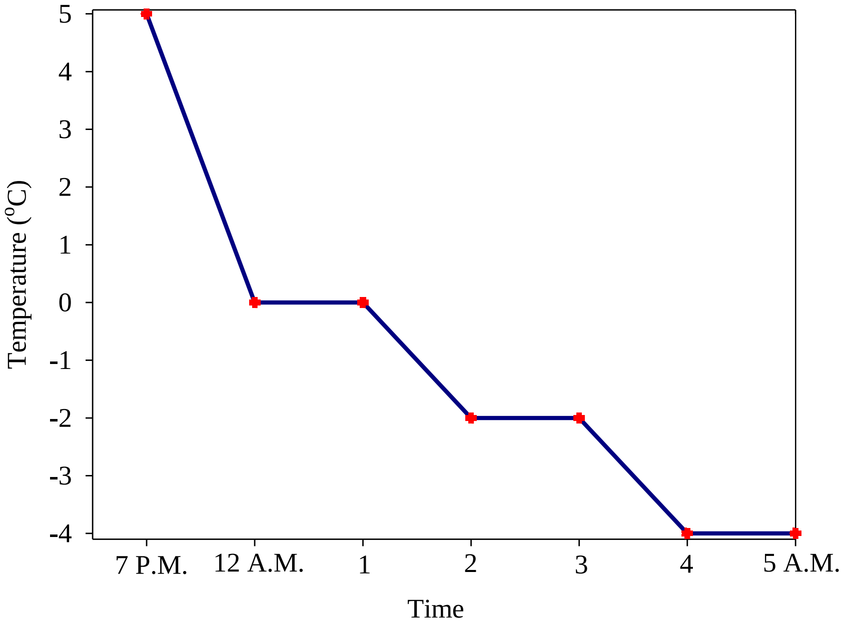

Fig. 2

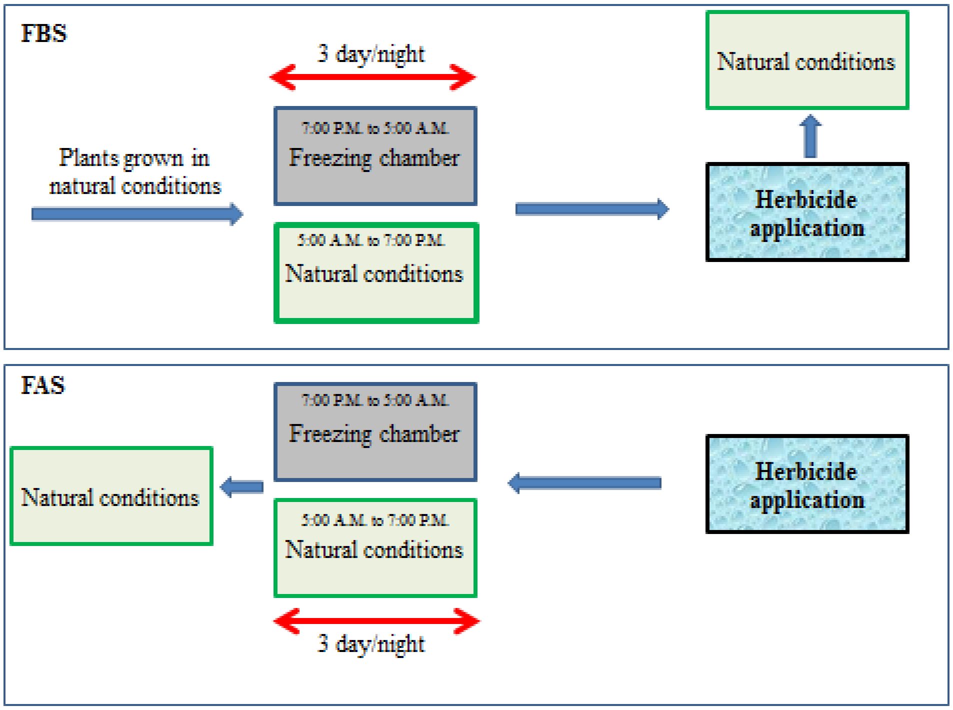
FBS
3 day/night
Natural conditions
7:00 P.M. to 5:00 A.M.
Freezing chamber
Plants grown in natural conditions
Herbicide application
5:00 A.M. to 7:00 P.M.
Natural conditions
FAS
7:00 P.M. to 5:00 A.M.
Freezing chamber
Natural conditions
Herbicide application
5:00 A.M. to 7:00 P.M.
Natural conditions
3 day/night

Fig. 3

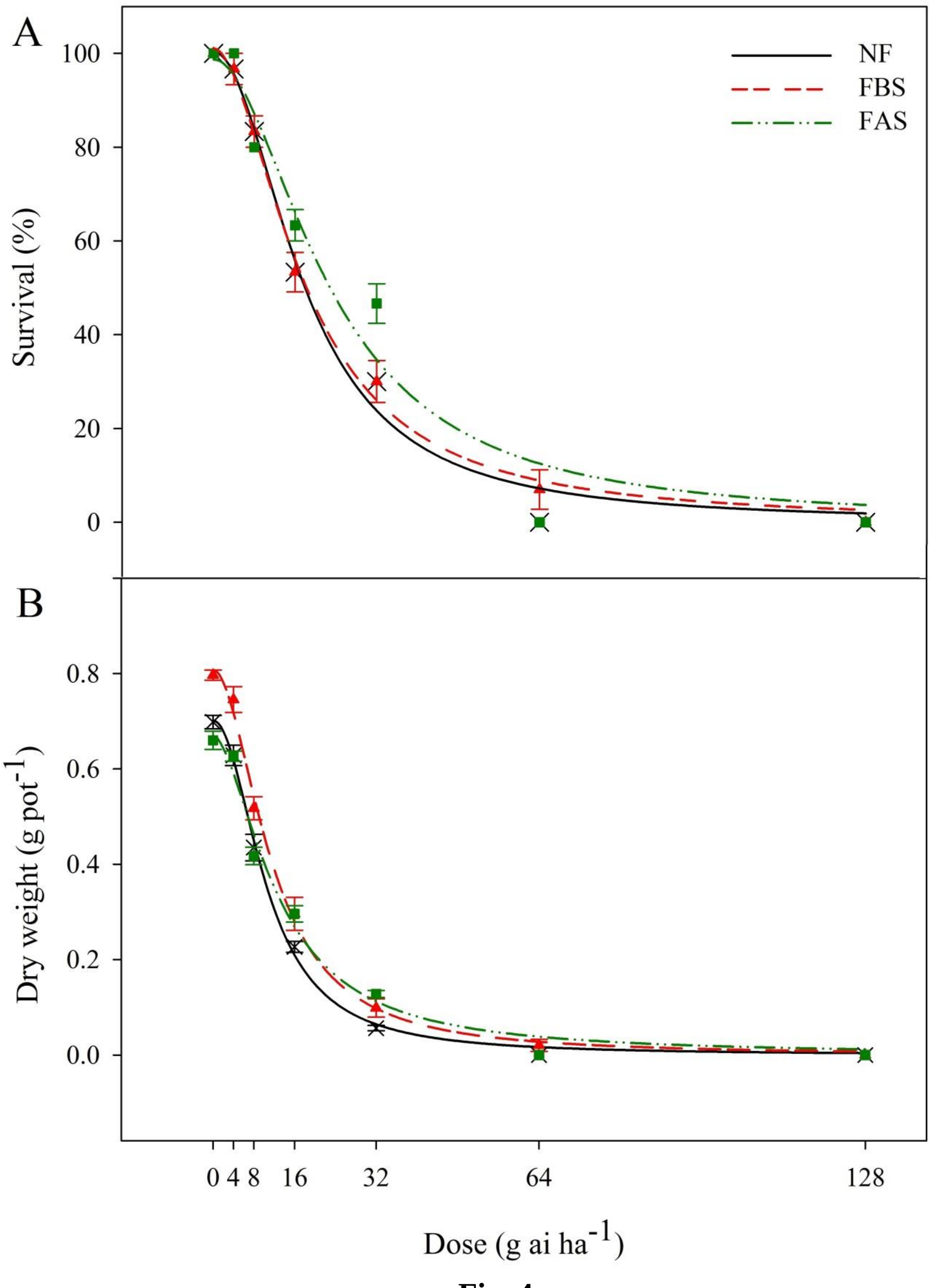
A
NF
FBS
FAS
Survival (%)
100
80
60
40
20
0
B
Dry weight (g pot-1)
0.8
0.6
0.4
0.2
0.0
0 4 8 16 32 64 128
Dose (g ai ha-1)

Fig. 4

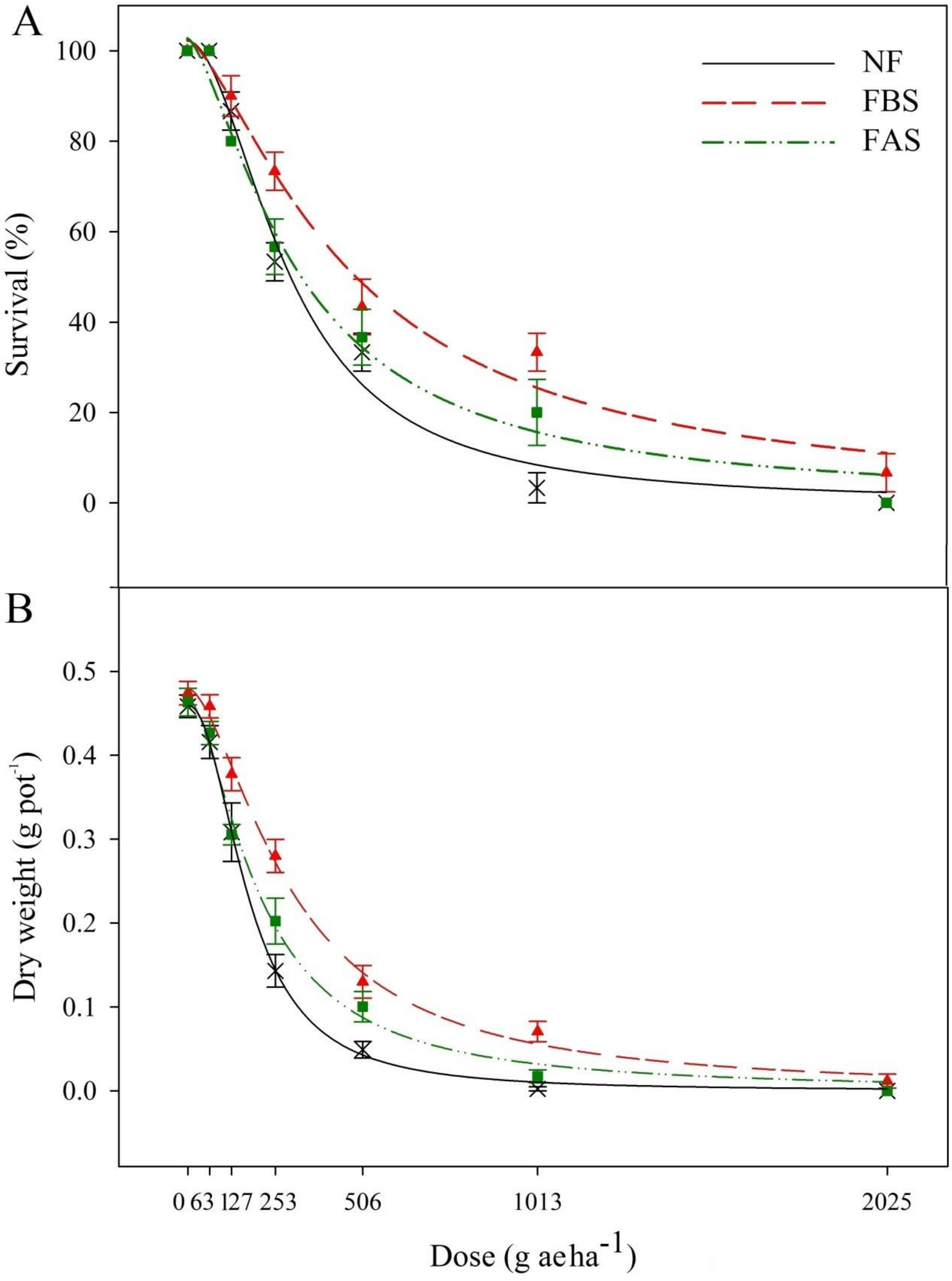
A
B
NF
FBS
FAS
Survival (%)
100
80
60
40
20
0
Dry weight (g pot-1)
0.5
0.4
0.3
0.2
0.1
0.0
0 63 127 253 506 1013 2025
Dose (g aeha-1)

Fig. 5

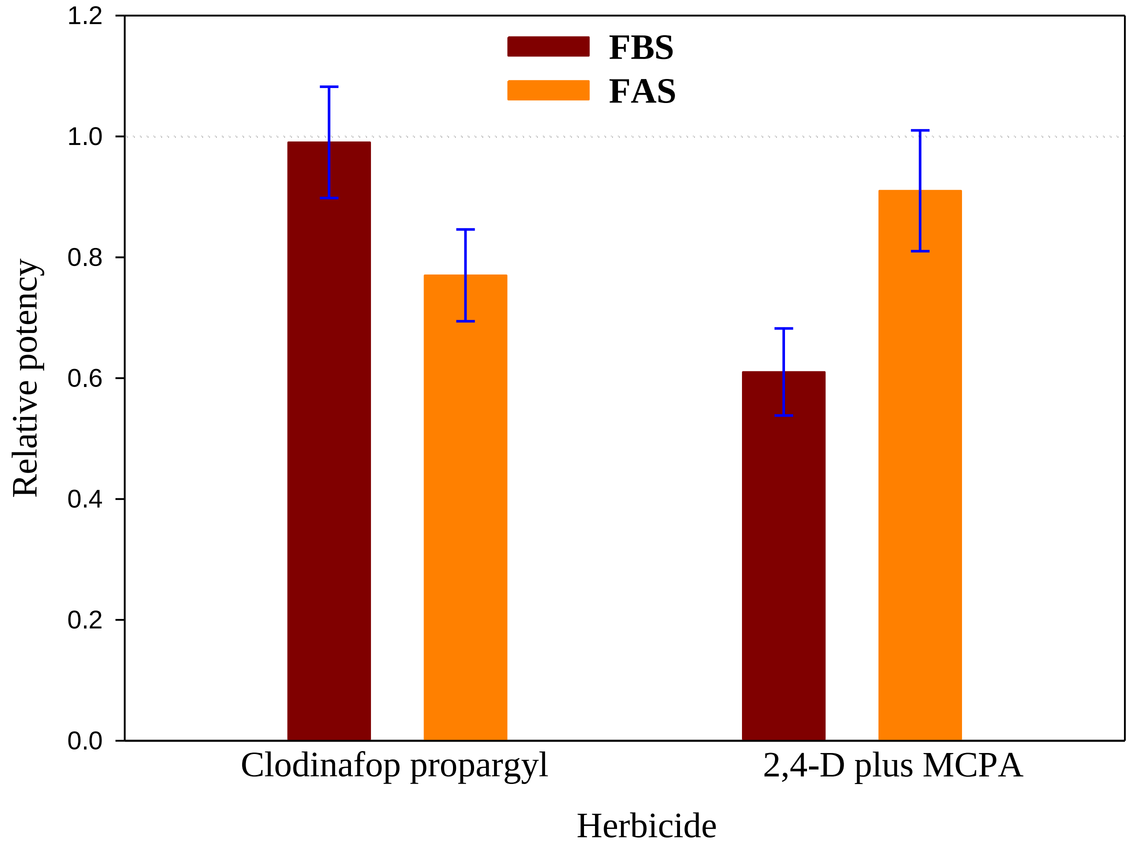

1.2
1.0
0.8
0.6
0.4
0.2
0.0
Relative potency
FBS
FAS
Clodinafop propargyl
2,4-D plus MCPA
Herbicide

Fig. 6

 Click here to access/download;Figure;Figures (3).docx

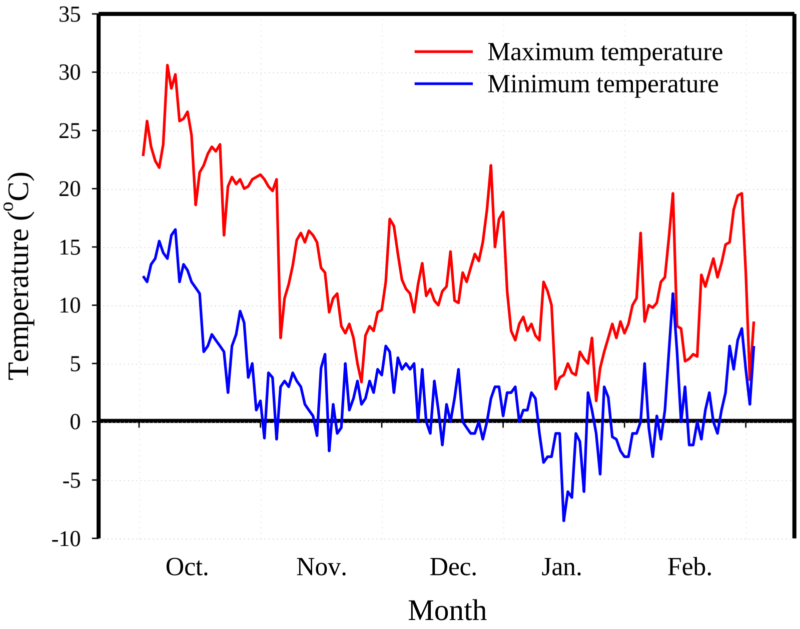

Fig 1.

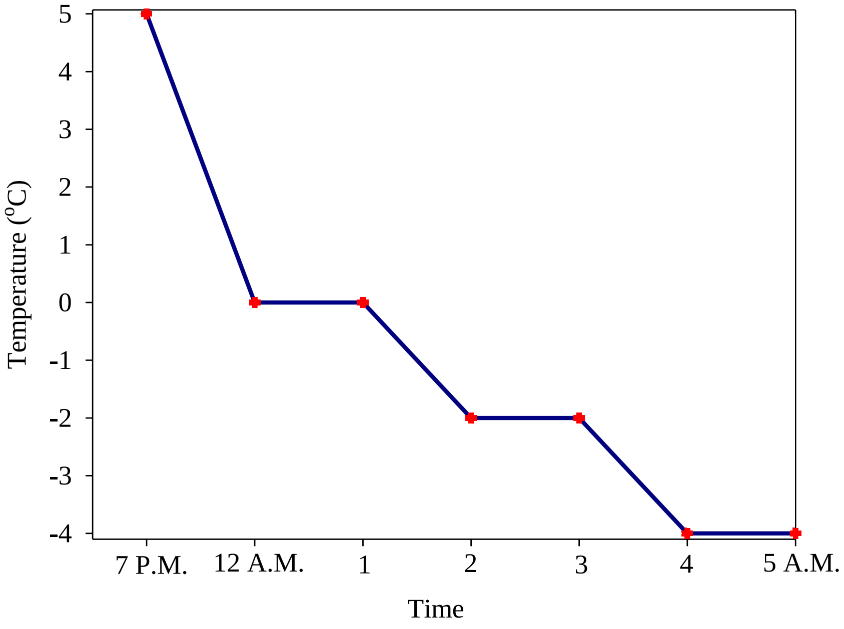

Fig. 2

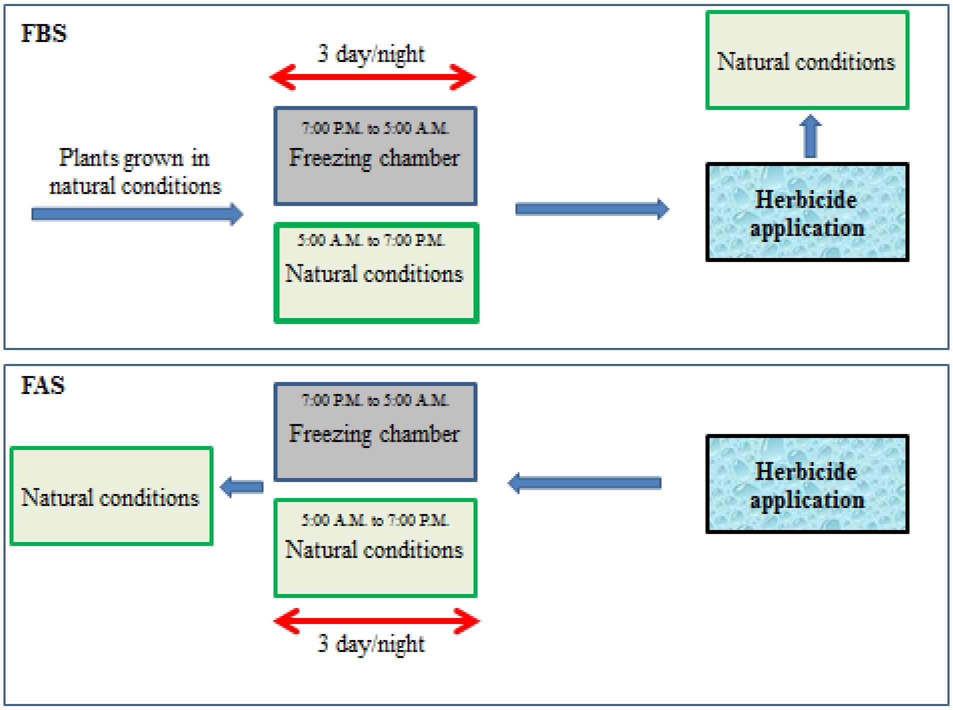
FBS
3 day/night
7:00 P.M. to 5:00 A.M.
Freezing chamber
5:00 A.M. to 7:00 P.M.
Natural conditions
Plants grown in natural conditions
Herbicide application
Natural conditions
FAS
7:00 P.M. to 5:00 A.M.
Freezing chamber
5:00 A.M. to 7:00 P.M.
Natural conditions
Natural conditions
Herbicide application
3 day/night

Fig. 3

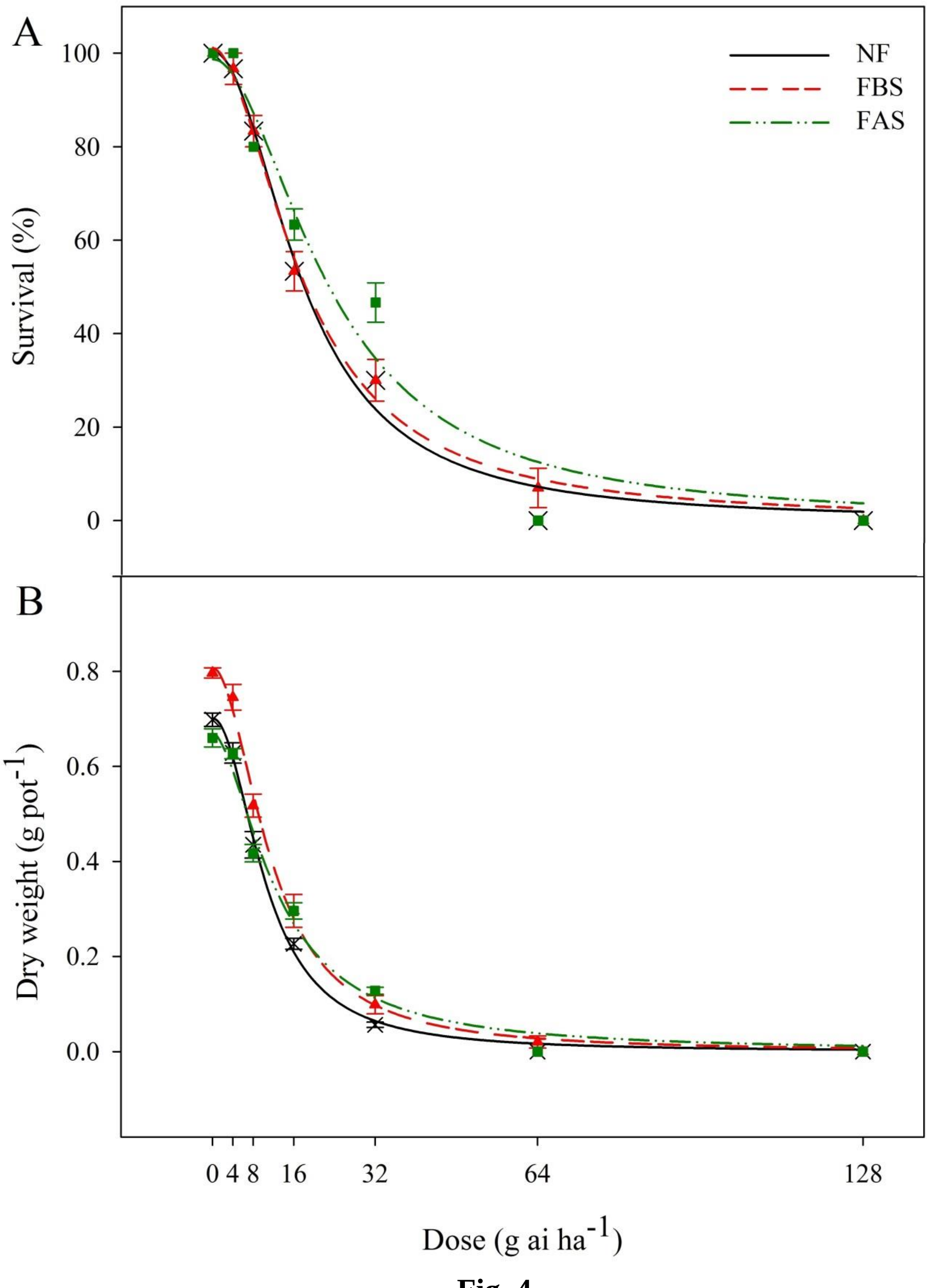
A
NF
FBS
FAS
Survival (%)
100
80
60
40
20
0
B
Dry weight (g pot-1)
0.8
0.6
0.4
0.2
0.0
0 4 8 16 32 64 128
Dose (g ai ha-1)

Fig. 4

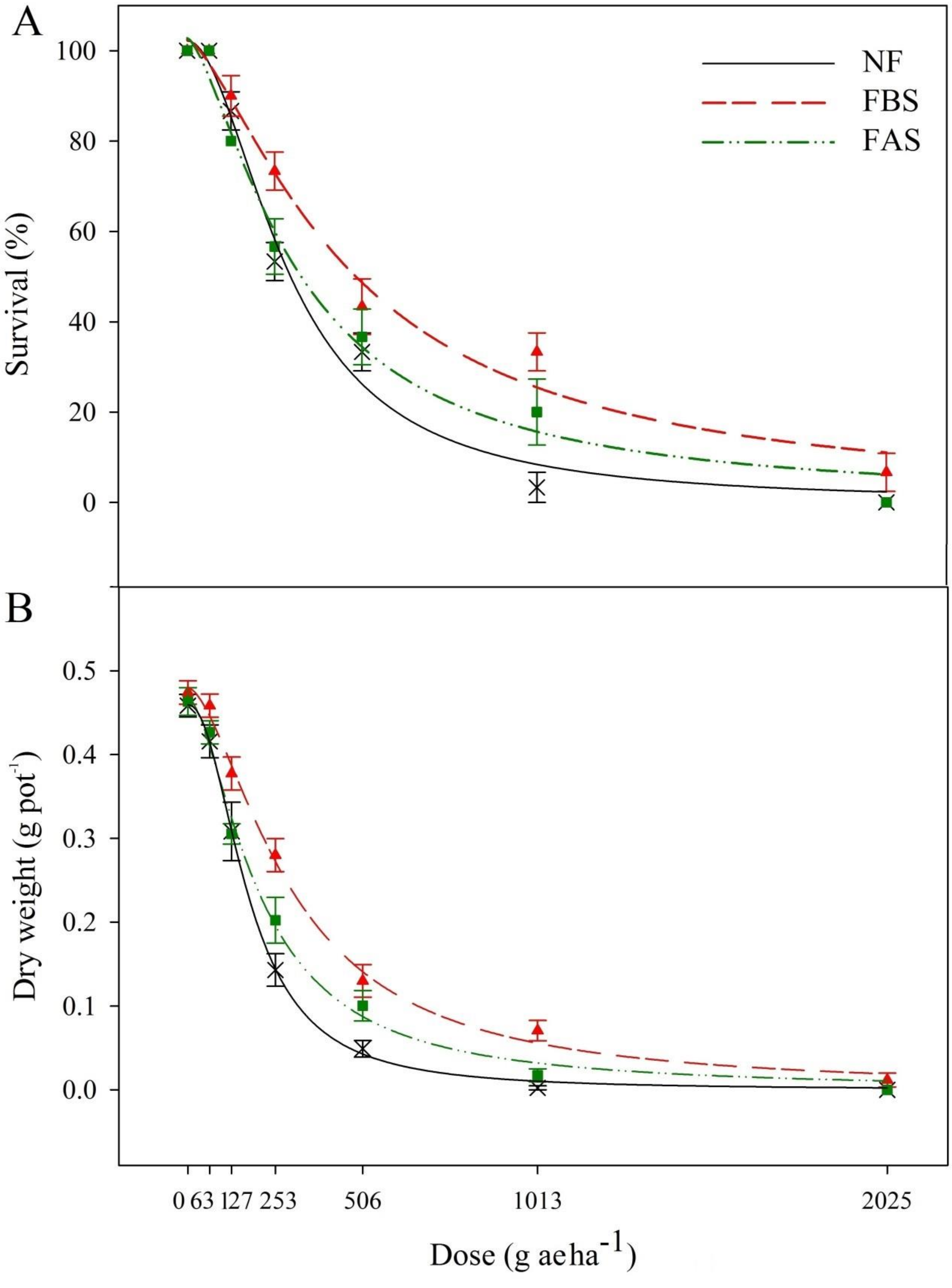

A
B
NF
FBS
FAS
Survival (%)
100
80
60
40
20
0
Dry weight (g pot-1)
0.5
0.4
0.3
0.2
0.1
0.0
0 63 127 253 506 1013 2025
Dose (g ae ha-1)

Fig. 5

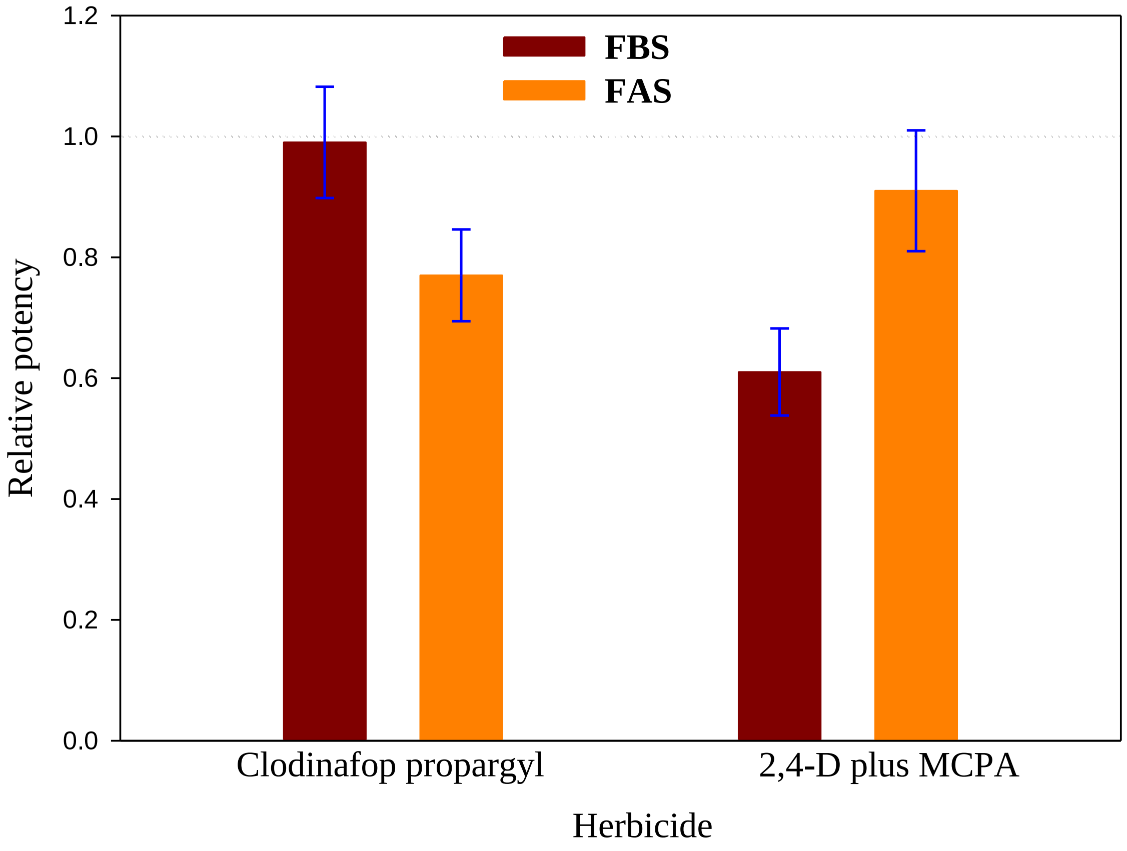

FBS
FAS
Relative potency
1.2
1.0
0.8
0.6
0.4
0.2
0.0
Clodinafop propargyl
2,4-D plus MCPA
Herbicide

Fig. 6

Click here to access/download
Supporting Information
DATA.xlsx

Freezing stress affects the efficacy of clodinafop-propargyl and 2,4-D plus MCPA on wild oat (*Avena ludoviciana* Durieu) and turnipweed [*Rapistrum rugosum* (L.) All.] in wheat (*Triticum aestivum* L.)

Short running title: Freezing and herbicide efficacy

Alireza Hasanfard[1], Mehdi Rastgoo[1], Ebrahim Izadi Darbandi[1], Ahmad Nezami[1], Bhagirath Singh Chauhan[2]

[1] Department of Agrotechnology, Faculty of Agriculture, Ferdowsi University of Mashhad, Iran

[2] Queensland Alliance for Agriculture and Food Innovation (QAAFI) *and* School of Agriculture and Food Sciences (SAFS), The University of Queensland, Gatton, Queensland 4343, Australia

***Corresponding author:** m.rastgoo@um.ac.ir

Abstract

The occurrence of freezing stress around herbicides application is one of the most important factors influencing their performance. This experiment was performed to evaluate the efficacy of clodinafop-propargyl and 2,4-D plus MCPA, the most important herbicides used in wheat fields in Iran, under the influence of a freezing treatment (-4°C). Wheat and its two common weeds, winter wild oat (*Avena ludoviciana* Durieu) and turnipweed [*Rapistrum rugosum* (L.) All.], were exposed to the freezing treatment for three nights from 7:00 P.M. to 5:00 A.M. before and after herbicide application, and their response was compared with plants that did not grow under freezing stress. Under no freezing (NF) and freezing after spray (FAS) conditions, winter wild oat was completely controlled with the recommended dose of clodinafop-propargyl (64 g ai ha^{-1}; hereafter g ha^{-1}). However, the survival percentage of winter wild oat in the freezing before spray (FBS) of clodinafop-propargyl 64 g ha^{-1} was 7%, and it was completely controlled with twice the recommended dose (128 g ha^{-1}). Under NF conditions and FAS treatment, turnipweed was completely controlled with twice the recommended dose of 2,4-D plus MCPA (2025 g ae ha^{-1}; hereafter g ha^{-1}). However, in the FBS treatment, the survival of turnipweed was 7% (2025 g ha^{-1}). The LD_{50} (dose required to control 50% of individuals in the population) and GR_{50} (dose causing 50% growth reduction of plants) rankings were NF<FBS<FAS for clodinafop-propargyl and NF<FAS<FBS for 2,4-D plus MCPA. Selectivity index for clodinafop-propargyl in NF conditions, FBS, and FAS treatments was 2.4, 0.91, and 0.78, respectively, and, for 2,4-D plus MCPA, it was 2.6, 0.12, and 0.88, respectively. According to the results of this experiment, it can be stated that the freezing stress after the spraying of clodinafop-propargyl and the freezing stress before a spray of 2,4-D plus MCPA would further reduce the efficacy of these herbicides.

Keywords: GR_{50}, Herbicide efficacy, LD_{50}, Selectivity index

Introduction

Winter wild oat (*Avena ludoviciana* Durieu) and turnipweed [*Rapistrum rugosum* (L.) All.] are the most important annual weeds of winter wheat (*Triticum aestivum* L.) in Iran, which cause significant reductions in wheat yield [1, 2]. The high reproduction potential and fitness to freezing stress, which results in a wide dispersal of weed seeds are the major challenges in the management of winter annual weeds like wild oat and turnipweed. Therefore, in cold regions of Iran, the dispersal and geographical distribution of these weeds are more significant than other weed species [3].

One of the most important consequences of climate change is the change in glaciers' occurrence patterns [4]. A sharp drop in temperature after high temperatures leads to crop damage. Increasing autumn temperatures in Iran, resulting from climate change, have led to reduced freezing tolerance of autumn crops [3]. In other words, autumn crops will not tolerate freezing stress due to the lack of hardening process in autumn. As a result, they will be damaged by decreasing temperatures during winter, and their yield will be reduced [3, 4]. It has been reported that increasing autumn temperatures resulting from climate change in western Canada led to reduced freezing tolerance of winter cereals [5]. In other words, due to the lack of optimal cold acclimation in autumn, crops will not be able to tolerate freezing stress. As a result, they will be damaged by decreasing temperatures during winter or by the occurrence of late spring frosts [6]. However, weeds generally have a higher ability to tolerate freezing stress [7]. Based on this, it can be inferred that climate change is adversely changing agricultural production and can significantly change the management of current cropping systems that have evolved under optimum climatic conditions.

Hand weeding and hoeing are not common in wheat fields, and mechanical weed management methods are not effective, so chemical methods are mainly used to control weeds in the wheat fields in Iran. Clodinafop-propargyl and 2,4-D plus MCPA are the most common herbicides used for controlling weeds in wheat fields in Iran. Due to the particular importance of this crop in food security, their wide excessive applications can be justified [8, 9]. Clodinafop-propargyl is a systemic and selective herbicide of the aryloxyphenoxypropionate (AOPP) group and used post-emergent to control annual grass weeds in wheat. This herbicide inhibits the activity of acetyl-coenzyme A carboxylase (ACCase) and thus disrupts the biosynthesis of fatty acids [10]. 2,4-D plus MCPA, synthetic auxin herbicides, is a systemic herbicide mixture registered for selective post-emergence control of annual broadleaf weeds in wheat [11].

The efficacy of herbicides is significantly affected by environmental conditions before, during, and after application [12]. It has been reported that environmental factors such as light intensity, temperature, water stress, relative humidity, and nutrient availability alter the herbicide efficacy [13, 14]. On the other hand, plant species and their growth stages also affect herbicide efficacy [15]. Numerous studies indicate that ambient temperature is one of the most influential factors affecting the performance of herbicides [14, 16, 17]. Increasing the temperature is likely to increase the absorption and translocation of some herbicides and increase their performance. In contrast, the improvement in herbicide performance at low temperatures may be due to a reduction in the rate of herbicide metabolism [17, 18]. In cold regions, freezing temperatures occur around the time of herbicide application. Therefore, the occurrence of severe freezing that causes damage to plant leaves will have adverse effects on the activity and efficacy of foliar-applied herbicides [19]. On the other hand, application of herbicides in unfavorable environmental conditions (such as adverse temperature before, during, and after herbicide

application) causes stress in the crop, and this changes the selectivity index of the herbicide, which will ultimately cause damage to the crop [20]. The selectivity index refers to the potential of a herbicide to eliminate weeds in a crop without affecting the yield or quality of the final product [21].

At the appropriate growth stage of weeds for herbicide application, the temperature usually decreases, and this issue leads to a decrease in the performance of herbicides. Due to the lack of information on the efficacy of herbicides under freezing stress, this experiment was conducted to evaluate the efficacy of clodinafop-propargyl and 2,4-D plus MCPA on wild oat and turnipweed under freezing stress before and after herbicide application.

Materials and methods

Experimental site and plant material

Seeds of wheat (cv. Mihan) were harvested from Khorasan Razavi Agricultural and Natural Resources Research and Education Center, and seeds of winter wild oat and turnipweed were collected from infested farms around Mashhad-Iran in May 2019. Experiments were conducted at the Faculty of Agriculture, the Ferdowsi University of Mashhad, Iran (Lat 36° 15′ N, Long 59° 28 E; 985 m Altitude). To break the dormancy of winter wild oat and turnipweed, their seeds were exposed to moist-chilling treatment and potassium nitrate (KNO_3) 0.2% solution for 3 days at 5℃ in the dark, respectively [3]. The seeds were placed in 9-cm diameter Petri dishes (20-25 seeds per Petri dish) on moist filter papers and kept at 20℃. After 5 days, the germination percentage of winter wild oat and turnipweed seeds was >90%. The experiment was repeated twice with three replicates. To experience the same cold acclimation, the plants were planted at approximately the same time on October 21 (first run) and 24 (second run) in 2019. Plastic pots

(12 cm in diameter and 20 cm in height) were used in the trial, and pots were filled with a mixture of sand, agricultural soil, and peat moss (2:2:1 sand, agricultural soil, and peat moss, respectively). For uniform emergence, turnipweed seeds were planted in a seedling tray and transferred to pots in the cotyledon stage. Eight germinated seeds of wild oat were planted at 4-5 cm depth below the soil surface. To induce cold acclimation, the plants were kept outdoors, grown in natural conditions (Fig. 1), and irrigated according to daily requirements until they were tested.

Fig 1. Minimum and maximum daily air temperatures at the experimental site from October 2019 to February 2020. Relative humidity of 40-50% under the natural photoperiod [3].

Experimental factors

Experimental factors included freezing stress at three conditions, including no freezing (NF), freezing before spray (FBS), and freezing after spray (FAS). Plant species included wheat, winter wild oat, and turnipweed. Herbicides were applied at 0, 6.25, 12.5, 25, 50, 100, and 200% of the recommended dose for the herbicide clodinafop-propargyl (0.8 L ha^{-1}; 8% EC) and 2,4-D plus MCPA (1.5 L ha^{-1}; 67.5% SL).

Freezing treatment and herbicide application

Under NF, the plants grew completely in natural winter conditions without exposing to freezing stress. For FBS, the plants grew up to the 3-4 leaf stage under natural conditions. They were exposed to freezing from 7:00 P.M. to 5:00 A.M. for three nights before herbicide application [19]. Freezing treatment was performed using a thermogradient freezer (Weiss Technik). The initial temperature of the freezer was 5℃; however, the freezer was cooled down at a rate of 2℃ per hour to reach a temperature of -4℃. The temperature drop slope was adjusted based on the

long-term meteorological statistics of Mashhad in the time range of herbicide application. The pots were kept at -4℃ for 1 h and then removed from the freezer (Fig. 2).

Fig. 2 Trend of temperature decrease in thermogradient freezer during the night from 7:00 P.M. to 5:00 A.M.

Each morning at 5:30 A.M., the plants were transferred out of the freezer, kept in natural conditions until 6:30 P.M., and then returned to the freezer during the night. After three nights of freezing exposure, the pots were taken out of the freezer and sprayed with herbicides after 2 h [19]. After spraying, the plants were kept outside under natural conditions for the duration of the experiment.

In the FAS treatment, freezing stress was applied for three nights by placing the samples treated with herbicides in the thermogradient freezer. For this purpose, the plants were taken out of the freezer at 5:30 A.M., transferred to natural conditions, and then returned to the freezer at 6:30 P.M. for the overnight freezing treatment (Similar to FBS treatment). Three nights after freezing treatment, the plants were transferred to natural conditions to continue the trial process (Fig. 3).

Fig. 3 Schematic diagram of the experimental study design. Abbreviation: FBS (freezing before spray); FAS (freezing after spray).

The conditions inside the thermogradient freezer were the same for both freezing treatments before and after spray. The experiment was performed in the freezer in completely dark conditions and at the same relative humidity (35±5%) for all treatments. Temperature changes in the freezer were regularly monitored when the samples were in the freezer. For all the freezing treatments, herbicides were applied at the same time. Plants were transferred to natural

conditions after three nights of freezing treatment at 5:30 A.M. Also, plants related to freezing after spray were transferred to the freezer at 6:30 P.M. On the same day, plants that were not exposed to freezing were sprayed from 7:30 A.M. to 10.30 A.M. The herbicides were applied by a researcher-made moving boom sprayer equipped with a TeeJet 8002 flat fan nozzle (spray output volume of 200 L ha^{-1} at a pressure of 260 kPa). Plants were returned and maintained outdoor under natural conditions of winter after the treatment.

Measurement of traits

Four weeks after herbicide treatments, survival percentage (Eq. 1) and shoot dry weight were measured by placing the samples in an oven at 75℃ for 48 h. Plants that produced new leaves were considered alive and those that experienced severe chlorosis and stunted growth were recorded as dead [22].

$$Survival\ \% = \frac{N1}{N0} \times 100 \qquad \text{(Eq. 1)}$$

Where, N_1 and N_0 are the numbers of alive plants four weeks after herbicide treatment and the number of alive plants before herbicide treatment, respectively.

Dose–response experiments

The response of weed traits to the doses of herbicides tested was analyzed by nonlinear regression. For this purpose, the data were fitted to a three-parameter log-logistic equation (Eq. 2) [23].

$$Y = \frac{d}{1+e^{b(\log x - loge)}} \qquad \text{(Eq. 2)}$$

Where, Y is the response rate (survival % or dry weight) at dose x, d is the upper limit for Y, b is the slope of the curve around e, and e is the effective dose (g ai or ae ha^{-1}). Here, e is replaceable with LD_{50} (dose required to control 50% of individuals in the population) or GR_{50} (dose causing 50% dry weight reduction of plants).

Relative potency

Relative potency was used to determine herbicide efficacy under freezing stress and no freezing stress (Eq. 3) [24].

$$Relative\ potency = \frac{ED_{50A}}{ED_{50B}} \qquad \text{(Eq. 3)}$$

Where, ED_{50A} is the dose required to reduce survival by 50% in NF, and ED_{50B} is the dose needed to reduce survival by 50% in FBS or FAS. Relative potency = 1 indicates that freezing treatment (FBS and FAS) did not affect herbicide efficacy. If relative potency > 1, it indicates that the herbicides are more efficient under freezing stress. Relative potency < 1 indicates the decreased effectiveness of the herbicides under freezing stress with respect to optimum conditions.

Selectivity index

The ratio between doses that caused 10% damage to crops and 90% damage to weeds was considered a selectivity index [24] and calculated using Eq. 4.

$$Selectivity\ index\ (10,90) = \frac{ED_{10\ (crop)}}{ED_{90\ (weed)}} \qquad \text{(Eq. 4)}$$

Where, ED_{90} is 90% of the herbicide effect on weed survival (herbicide dose to kill 90% of the population), and ED_{10} is 10% of the herbicide impact on crop survival (a 10% effect on the crop). The higher the SI, the more selective the herbicide in the crop [24].

Statistical analysis

The experiments were conducted using a completely randomized design in factorial arrangement with three replications. Afterward, differences between treatments were compared with the standard error of mean (SEM) values. For data analysis, analysis of variance (ANOVA) was performed using the SAS software (v. 9.4, SAS Institute Inc, Cary, NC, USA). Data for both runs were pooled as no significant differences were observed between runs ($P > 0.05$). Homogeneity of variance was confirmed using Levene's test. Analysis of biometric data was performed by the R program (v. 4.0.4) [25]. Dose-response curves were also fitted with the asymmetrical sigmoid form. LD_{50} and GR_{50} were separated using 95% confidence intervals (CI).

Results

Clodinafop-propargyl

Survival percentage and LD_{50}

The dose-response experiments were similar for NF and FAS treatments at the recommended dose (Figure 4A). Thus, in both conditions, winter wild oat was completely controlled with the recommended dose (64 g ai ha^{-1}). However, the survival of seedlings in the FBS treatment was 7%, and to achieve complete control, 2-fold the recommended dose (128 g ha^{-1}) was needed. The LD_{50} in NF, FBS, and FAS was 17.9, 18.1, and 23.2 g ha^{-1}, respectively (Table 1).

Fig. 4 Survival (A) and dry weight (B) of winter wild oat treated with various doses of clodinafop-propargyl under different freezing treatments. Abbreviation: NF (no freezing); FBS (freezing before spray): FAS (freezing after spray). The recommended dose of clodinafop-propargyl in Iran is 64 g ha^{-1}.

Table 1 The dose of clodinafop-propargyl and 2,4-D plus MCPA required for 50% mortality (LD_{50}) and the dose that caused 50% inhibition of growth noted by biomass (GR_{50}) of winter wild oat and turnipweed under different freezing treatments.

Herbicide	Freezing Treatment^{a}	b^{b}±SE^{a}	d^{c}±SE	LD_{50} ±SE	95% CI^{e}	R^2
			%	g ai or ae ha^{-1}		
Clodinafop-propargyl	NF	$2.0 \pm 0.19^{**d}$	$100.3 \pm 2.8^{**}$	$17.9 \pm 1.2^{**}$	15.6-20.2	0.99
	FBS	$1.9 \pm 0.17^{**}$	$101.0 \pm 2.9^{**}$	$18.1 \pm 1.2^{**}$	15.7-20.5	0.99
	FAS	$1.9 \pm 0.19^{**}$	$98.7 \pm 2.9^{**}$	$23.2 \pm 1.7^{**}$	19.8-26.6	0.96
2,4-D plus MCPA	NF	$1.9 \pm 0.20^{**}$	$102.2 \pm 3.2^{**}$	$292.8 \pm 21.9^{**}$	249.4-336.3	0.98
	FBS	$1.5 \pm 0.15^{**}$	$102.4 \pm 3.2^{**}$	$472.8 \pm 42.4^{**}$	388.8-556.8	0.98
	FAS	$1.5 \pm 0.14^{**}$	$102.8 \pm 3.5^{**}$	$318.8 \pm 28.9^{**}$	261.6-376.1	0.98
		b^{b}±SE^{a}	d^{c}±SE	GR_{50} ±SE	95% CI^{e}	
		g pot^{-1}		g ai or ae ha^{-1}		
Clodinafop-propargyl	NF	$2.1 \pm 0.15^{**}$	$0.70 \pm 0.02^{**}$	$10.6 \pm 0.52^{**}$	9.6-11.6	0.99
	FBS	$2.0 \pm 0.12^{**}$	$0.80 \pm 0.02^{**}$	$11.6 \pm 0.51^{**}$	10.6-12.6	0.99
	FAS	$1.7 \pm 0.12^{**}$	$0.67 \pm 0.02^{**}$	$12.8 \pm 0.74^{**}$	11.3-14.3	0.98
2,4-D plus MCPA	NF	$2.2 \pm 0.23^{**}$	$0.46 \pm 0.01^{**}$	$176.2 \pm 11.3^{**}$	153.9-198.6	0.99
	FBS	$1.7 \pm 0.16^{**}$	$0.48 \pm 0.01^{**}$	$298.6 \pm 21.5^{**}$	256.0-341.3	0.99
	FAS	$1.6 \pm 0.15^{**}$	$0.47 \pm 0.01^{**}$	$207.8 \pm 15.9^{**}$	176.2-239.4	0.99

Abbreviation: NF (no freezing); FBS (freezing before spray); FAS (freezing after spray); SE: Standard Error. b is the slope of the curve around e (LD_{50} and GR_{50}); d is the upper limit for Y (survival % or dry weight); **: significant at $p \leq 0.01$. LD_{50} and GR_{50} were separated using their 95% confidence intervals (CI). The data shows the parameter's mean ± SE. n = 6

Dry weight and GR_{50}

The dry weight of winter wild oat in NF and FAS with the recommended dose (64 g ha^{-1}) and FBS with twice the recommended dose (128 g ha^{-1}) reached 0 g (Fig. 4B). The GR_{50} of plants not exposed to freezing stress was 10.6 g ha^{-1}. The GR_{50} was 11.6 and 12.8 g ha^{-1} in FBS and FAS, respectively (Table 1).

2,4-D plus MCPA

Survival percentage and LD_{50}

The dose-response experiments of 2,4-D plus MCPA (similar to clodinafop-propargyl) were the same in NF and FAS treatments (Fig. 5A). In both conditions, turnipweed was completely controlled with twice the recommended dose (2025 g ae ha^{-1}). However, in the FBS treatment, the survival of turnipweed was 7%. At the recommended dose (1012.5 g ha^{-1}), the survival of turnipweed in NF, FBS, and FAS was 3%, 33%, and 20%, respectively. The LD_{50} in NF, FBS, and FAS was 293, 473, and 319 g ha^{-1}, respectively (Table 1).

Fig. 5 Survival (A) and dry weight (B) of turnipweed treated with various doses of 2,4-D plus MCPA under different freezing treatments. Abbreviation: NF (no freezing); FBS (freezing before spray): FAS (freezing after spray). The recommended dose of 2,4-D+MCPA in Iran is 1012.5 g ha^{-1}.

Dry weight and GR_{50}

The dry weight of turnipweed in NF and FAS with twice the recommended dose (2025 g ha^{-1}) of 2,4-D plus MCPA was reduced to 0 (Fig. 5 B). However, in the FBS treatment, the dry weight of turnipweed with twice the recommended dose was 0.012 g per pot. At the recommended dose, the dry weight of turnipweed in FBS was 97% and 76% more than NF and FAS, respectively. The GR_{50} of NF, FBS, and FAS were 176, 299, and 208 g ha^{-1}, respectively (Table 1).

Relative potency

The relative potency of clodinafop-propargyl and 2,4-D plus MCPA was significantly reduced in FBS and FAS compared to NF (Fig. 6). Thus, the relative potency in FBS and FAS of clodinafop-propargyl was 0.99 and 0.77, respectively, and at 2,4-D plus MCPA was 0.62 and 0.92, respectively.

Fig. 6 Relative potency of clodinafop-propargyl and 2,4-D plus MCPA under different freezing treatments. Abbreviation: FBS (freezing before spray) and FAS (freezing after spray). The error bars indicate standard error (SE). The dashed line represents the relative potency = 1.0.

Selectivity index

As shown in Table 2, selectivity index values for clodinafop-propargyl in NF, FBS, and FAS treatments were 2.4, 0.91, and 0.78, respectively, and, for 2,4-D plus MCPA, they were 2.6, 0.12, and 0.88, respectively. For clodinafop-propargyl, the LD_{10} of wheat in NF was 127.9 g ha^{-1}. However, LD_{10} of wheat in the FBS and FAS was 53.5 and 57.1 g ha^{-1}, respectively (Table 2). For 2,4-D plus MCPA, the LD_{10} of wheat in NF, FBS, and FAS was 2380, 264, and 1236 g ha^{-1}, respectively.

Table 2 Sensitivity of plant species of wheat, winter wild oat, and turnipweed to clodinafop-propargyl and 2,4-D plus MCPA under different freezing treatments.

Freezing Treatment	Plant species	Clodinafop-propargyl: LD_{10} (wheat) and LD_{90} (winter wild oat) (g ai ha^{-1}) ± SE	Clodinafop-propargyl: SI	2,4-D plus MCPA: LD_{10} (wheat) and LD_{90} (turnipweed) (g ae ha^{-1}) ± SE	2,4-D plus MCPA: SI
NF	Wheat	127.9 ± 8.8	-	2380 ± 455	-
	Weed	53.5 ± 5.4	2.4	908 ± 108	2.6
FBS	Wheat	53.5 ± 11.0	-	264 ± 61.0	-
	Weed	59.0 ± 6.4	0.91	2144 ± 327	0.12
FAS	Wheat	57.1 ± 8.7	-	1236 ± 163	-
	Weed	73.6 ± 7.2	0.78	1400 ± 199	0.88

Abbreviation: NF; No freezing, FBS: Freezing before spray, FAS: Freezing after spray SI: Selectivity index. The selectivity index (SI) was calculated as follows: *Selectivity index (10, 90):* $\frac{ED10\ (crop)}{ED90\ (weed)}$

Discussion

The required dose of clodinafop-propargyl for complete control of winter wild oat seedlings in FBS treatment was twice that of NF and FAS treatments. The trend of the reducing survival % in seedlings related to the FBS treatment and NF was similar. The LD_{50} for clodinafop-propargyl in freezing treatments shows that exposure of winter wild oat seedlings to freezing treatment for three nights before and after spray reduces the efficacy of this herbicide compared to NF. The LD_{50} was 22% and 23% higher in the FAS compared to the FBS and NF, respectively. Freezing treatment for three nights before and after spraying of clethodim herbicide (the mode of action similar to clodinafop-propargyl) led to an increase in LD_{50} [19]. It decreased herbicide efficacy in rigid ryegrass (*Lolium rigidum* Gaud.) populations compared to NF.

The dose of clodinafop-propargyl required to reduce winter wild oat biomass to zero in the FBS treatment was twice that of the NF and FAS treatments. The GR_{50} is more pronounced in FBS and FAS compared to NF, indicating a decrease in the efficacy of clodinafop-propargyl. The GR_{50} was 17% and 8% higher in the FAS treatment compared to NF and FBS, respectively. In other words, the occurrence of three nights of freezing stress after herbicide application reduced the performance of clodinafop-propargyl more than other treatments. It has been reported that wild oat plants (*Avena fatua*) that grew at low temperatures were less sensitive to difenzoquat than plants that grew at high temperatures [26].

Our results clearly show that the occurrence of freezing stress around spray reduces the efficacy of clodinafop-propargyl, especially in the treatment of FAS. Herbicide translocation in the phloem in freezing conditions could decline due to damage to the phloem cells, which may lead to a decrease in the herbicide efficacy. Decreased efficacy of sethoxydim at low temperatures due to reduced herbicide translocation for Quackgrass [*Elymus repens* (L.) Gould]

control was reported by Coupland [27]. In general, to maintain the herbicide efficacy under cold conditions, using clodinafop-propargyl after the freezing stress can be recommended in areas with a similar climate.

Although the three-night treatment of FBS and FAS resulted in a reduction in turnipweed control by 2,4-D plus MCPA compared to NF, this reduction was different in the two freezing treatments. At the recommended dose (1013 g ha^{-1}) in NF, almost all turnipweed plants were controlled, while the survival percentage of turnipweed in FBS and FAS treatments were 33% and 20%, respectively. Low temperatures around the time of herbicide application reduced the effectiveness of systemic herbicides [28]. Based on LD_{50} values, the efficacy of 2,4-D plus MCPA under FBS and FAS was 1.6 and 1.1 times lower compared to NF.

FAS is possible to damage the phloem cells, which reduces the translocation of the herbicide. The efficacy of 2,4-D plus MCPA in the treatment of FBS was reduced. The decrease in the effect of 2,4-D plus MCPA in the treatment of FBS is probably due to the reduction of absorption and translocation of this herbicide in turnipweed seedlings. In this regard, an increase in efficacy of 2,4-D and glyphosate at high temperatures compared to low temperatures in common ragweed (*Ambrosia artemisiifolia* L.) and giant ragweed (*Ambrosia trifida* L.) due to the increased absorption and/or translocation at high temperatures around herbicide application has been reported by Ganie et al. [29]. It has been also reported that low temperatures reduced glufosinate activity and translocation in wild radish (*Raphanus raphanistrum* L.) [30].

Turnipweed's dry weight at the recommended dose of 2,4-D plus MCPA (1012.5 g ha^{-1}) in FBS and FAS treatments was 35.5 and 8.5 times higher than the NF, respectively. Also, GR_{50} in FBS and FAS was 41% and 15.2% higher compared to NF, respectively. Although a decrease in herbicide efficacy is evident in both FBS and FAS treatments, high GR_{50} in FBS shows an

adverse effect of three nights of freezing before the application of 2,4-D plus MCPA. In other words, the efficacy of this herbicide reacts more to FBS than to FAS. In a similar study, the GR_{50} for 2,4-D increased at low temperature in common ragweed compared to high temperature [29].

The freezing stress around 2,4-D plus MCPA application reduced the efficacy of this herbicide in FBS and FAS treatments compared to NF. The decrease in the efficacy of this herbicide in the treatment of FBS is probably due to the reduction of absorption and translocation of the herbicide in the phloem during freezing. Absorption and translocation of 2,4-D plus MCPA might be reduced at low temperatures due to the effect on herbicide penetration facilitated by physicochemical factors, including the decreased rate of diffusion, increased viscosity of the cuticle, and physiological factors comprising decreases in photosynthesis, phloem translocation, and protoplasmic streaming [29]. In Iranian wheat fields, using the 2,4-D plus MCPA before the freezing stress can be recommended to maintain the herbicide efficacy under cold conditions.

When relative potency > 1, the herbicide efficacy is higher than optimal non-freezing conditions, and when relative potency < 1, the herbicide efficacy is lower than usual [24]. The relative potency values of clodinafop-propargyl and 2,4-D plus MCPA were lower than one under both FBS and FAS treatments in the present experiment. In other words, freezing stress reduced the efficacy of these herbicides. Based on the results of relative potency, the reduction of clodinafop-propargyl efficacy in the FAS treatment (33%) was more significant than in the FBS treatment (1%). Conversely, for 2,4-D plus MCPA, the reduction in efficacy was more significant in the FBS treatment (38%) compared to the FAS treatment (8%).

The selectivity index for clodinafop-propargyl and 2,4-D plus MCPA was > 1 in NF, indicating that both herbicides acted selectively in wheat. However, applying three nights of FBS and FAS of both herbicides reduced the selectivity index to less than one. In other words, freezing stress reduced the selectivity index of both herbicides, which led to phytotoxicity in wheat. For clodinafop-propargyl, LD_{10} of wheat in NF was 58% and 55% higher than FBS and FAS, respectively. The LD_{10} values of 2,4-D plus MCPA in NF were 89% and 48% higher compared to FBS and FAS, respectively. In other words, the dose required to kill 10% of wheat in both herbicides under NF was higher than FBS and FAS. Therefore, FBS and FAS increased the sensitivity of wheat to herbicide application. Freezing stress may reduce the rate of herbicide metabolism in the crop and ultimately increase its susceptibility [17].

Conclusions

The relative potency results for both herbicides were used to confirm the results of LD_{50} and GR_{50}. Thus, LD_{50} and GR_{50} were higher for clodinafop-propargyl in the FAS treatment than in NF and FBS treatments. The LD_{50} and GR_{50} were higher for the 2,4-D plus MCPA in the FBS treatment than in the NF and FAS treatments. In other words, clodinafop-propargyl was more effective than other conditions in the treatment of three nights of FAS and 2,4-D plus MCPA in the treatment of three nights of FBS. The selectivity index for clodinafop-propargyl was lower in the FAS treatment (0.78) and for 2,4-D plus MCPA was lower in the FBS treatment (0.12). The efficacy of both herbicides was affected by the FBS and FAS treatments. The efficacy of clodinafop-propargyl in the FAS treatment and the efficacy of 2,4-D plus MCPA in the FBS treatment were reduced. The values of the selectivity index of herbicides in the conditions of freezing stress showed that this index could be well used to determine the selective performance of these herbicides. Overall, according to the results of this experiment, it can be stated that FAS

with ACCase inhibitor herbicides, such as clodinafop-propargyl, may reduce their effectiveness in controlling narrow leaf weeds such as winter wild oat, and FBS with auxin type herbicides, such as 2,4-D plus MCPA, reduce their efficacy in controlling broadleaf weeds such as turnipweed. FBS for clodinafop-propargyl and FAS for 2,4-D plus MCPA had a slight adverse effect on the herbicide efficacy and selectivity index. Therefore, the use of these herbicides in the mentioned conditions (FBS for clodinafop-propargyl and FAS for 2,4-D plus MCPA) is recommended. In other words, to maintain high herbicide efficacy under cold (freezing) conditions, the use of clodinafop-propargyl after the freezing stress and 2,4-D plus MCPA before the freezing stress can be recommended in areas with a similar climate to Mashhad. Finally, the use of adjuvants, alternate herbicides, and various herbicide formulations to control grass and broadleaf weeds in Iranian wheat production is suggested to prevent the adverse effects of freezing stress on herbicides.

Conflicts of interest

The authors declare that they have no conflict of interest.

Acknowledgment

We are grateful to Ferdowsi University of Mashhad, Iran, for providing financial support for present research (Project No.3.49759). No additional external funding was received for this study.

References

1. Hatami ZM, Gherekhloo J, Rojano-Delgado AM, Osuna MD, Alcántara R, Fernández P, et al. Multiple mechanisms increase levels of resistance in *Rapistrum rugosum* to ALS herbicides. Front Plant Sci. 2016 Feb 22;7:169. https://doi.org/10.3389/fpls.2016.00169
2. Nosratti I, Sabeti P, Chaghamirzaee G, Heidari H. Weed problems, challenges, and opportunities in Iran. Crop Prot. 2020 Aug 1;134:104371. https://doi.org/10.1016/j.cropro.2017.10.007
3. Hasanfard A, Rastgoo M, Darbandi EI, Nezami A, Chauhan BS. Regeneration capacity after exposure to freezing in wild oat (*Avena ludoviciana* Durieu.) and turnipweed

(*Rapistrum rugosum* (L.) All.) in comparison with winter wheat. Environ. Exp. Bot. 2021 Jan 1;181:104271. https://doi.org/10.1016/j.envexpbot.2020.104271
4. Sierra-Almeida A, Cavieres LA, Bravo LA. Warmer temperatures affect the in situ freezing resistance of the Antarctic vascular plants. Front. Plant Sci. 2018 Oct 8;9:1456. https://doi.org/10.3389/fpls.2018.01456
5. Willick IR, Tanino KK, Gusta LV. The impact of global climate change on the freezing tolerance of winter cereals in Western Canada. J. Agron. Crop Sci. 2021 Feb;207(1):88-99. https://doi.org/10.1111/jac.12447
6. Vyse K, Pagter M, Zuther E, Hincha DK. Deacclimation after cold acclimation—a crucial, but widely neglected part of plant winter survival. J. Exp. Bot. 2019 Sep 15;70(18):4595-604. https://doi.org/10.1093/jxb/erz229
7. Cici SZ, Van Acker RC. Relative freezing tolerance of facultative winter annual weeds. Can. J. Plant Sci. 2011;91(4):759-63. https://doi.org/10.4141/cjps2010-001
8. Baghestani MA, Zand E, Soufizadeh S, Beheshtian M, Haghighi A, Barjasteh A, Birgani DG, Deihimfard R. Study on the efficacy of weed control in wheat (*Triticum aestivum* L.) with tank mixtures of grass herbicides with broadleaved herbicides. Crop Prot. 2008 Jan 1;27(1):104-11. https://doi.org/10.1016/j.cropro.2007.04.013
9. Enghiad A, Ufer D, Countryman AM, Thilmany DD. An overview of global wheat market fundamentals in an era of climate concerns. Int J Agron. 2017 Jul 2;2017. https://doi.org/10.1155/2017/3931897
10. Bajwa AA, Akhter MJ, Iqbal N, Peerzada AM, Hanif Z, Manalil S, Hashim S, Ali HH, Kebaso L, Frimpong D, Namubiru H. Biology and management of Avena fatua and Avena ludoviciana: two noxious weed species of agro-ecosystems. Environ Sci Pollut Res. 2017 Aug;24(24):19465-79. https://doi.org/10.1007/s11356-017-9810-y
11. Peterson MA, McMaster SA, Riechers DE, Skelton J, Stahlman PW. 2, 4-D past, present, and future: a review. Weed Technol. 2016 Jun;30(2):303-45. https://doi.org/10.1614/WT-D-15-00131.1
12. Collings LV, Blair AM, Gay AP, Dyer CJ, Mackay N. The effect of weather factors on the performance of herbicides to control *Alopecurus myosuroides* in winter wheat. Weed Res. 2003 Apr;43(2):146-53. https://doi.org/10.1046/j.1365-3180.2003.00327.x
13. Rocha-Pereira MR, Klar AE, Martins D, Ferreira de Souza GS, Villalba J. Efecto del estrés hídrico sobre la eficacia de los herbicidas aplicados en *Urochloa decumbens*. Cienc. Investig Agrar. 2012 Apr;39(1):211-20. http://dx.doi.org/10.4067/S0718-16202012000100018
14. Johnson BC, Young BG. Influence of temperature and relative humidity on the foliar activity of mesotrione. Weed Sci. 2002 Apr;50(2):157-61. https://doi.org/10.1614/0043-1745(2002)050[0157:IOTARH]2.0.CO;2.
15. Chahal PS, Aulakh JS, Rosenbaum K, Jhala AJ. Growth stage affects dose response of selected glyphosate-resistant weeds to premix of 2,4-D choline and glyphosate (Enlist Duo (TM) herbicide*). J. Agric. Sci. 2015 Nov 1;7(11):1. https://doi.org/10.5539/jas.v7n11p1
16. Waltz AL, Martin AR, Roeth FW, Lindquist JL. Glyphosate efficacy on velvetleaf varies with application time of day. Weed Technol. 2004 Dec;18(4):931-9. https://doi.org/10.1614/WT-03-123R3

17. Godar AS, Varanasi VK, Nakka S, Prasad PV, Thompson CR, Mithila J. Physiological and molecular mechanisms of differential sensitivity of Palmer amaranth (*Amaranthus palmeri*) to mesotrione at varying growth temperatures. Plos One. 2015 May 19;10(5):e0126731. https://doi.org/10.1371/journal.pone.0126731
18. Pline WA, Wu J, Hatzios KK. Effects of temperature and chemical additives on the response of transgenic herbicide-resistant soybeans to glufosinate and glyphosate applications. Pestic. Biochem. Phys. 1999 Oct 1;65(2):119-31. https://doi.org/10.1006/pest.1999.2437
19. Saini RK, Malone J, Preston C, Gill GS. Frost reduces clethodim efficacy in clethodim-resistant rigid ryegrass (*Lolium rigidum*) populations. Weed Sci. 2016 Jun;64(2):207-15. https://doi.org/10.1614/WS-D-15-00140.1
20. Moseley C, Hatzios KK, Hagood ES. Uptake, translocation, and metabolism of chlorimuron in soybean (*Glycine max*) and morningglory (*Ipomoea* spp.). Weed Tech. 1993 Jun;7(2):343-8. https://doi.org/10.1017/S0890037X00027706
21. Negrisoli E, Velini ED, Tofoli GR, Cavenaghi AL, Martins D, Morelli JL, Costa AG. Selectivity of pre-emergence herbicides to sugarcane treated with nematicides. Planta Daninha. 2004 Dec;22(4):567-75.
22. Powles SB, Lorraine-Colwill DF, Dellow JJ, Preston C. Evolved resistance to glyphosate in rigid ryegrass (*Lolium rigidum*) in Australia. Weed Sci. 1998 Oct;46(5):604-7.
23. Ritz C, Baty F, Streibig JC, Gerhard D. Dose-response analysis using R. Plos One. 2015 Dec 30;10(12):e0146021. https://doi.org/10.1371/journal.pone.0146021
24. Tind T, Mathiesen TJ, Jensen JE, Ritz C, Streibig JC. Using a selectivity index to evaluate logarithmic spraying in grass seed crops. Pest Manag Sci: formerly Pesticide Science. 2009 Nov;65(11):1257-62. https://doi.org/10.1002/ps.1819
25. Team RC. R Foundation for Statistical Computing, Vienna, available at: www. R-project. org. 2018.
26. Kowalczyk B, Caseley JC, McCready CC. The effect of pre-treatment temperature on some physiological responses of *Avena fatua* to difenzoquat and benzoylpropethyl. Asp Appl Biol. 1983;4:235-44.
27. Coupland D. Influence of environmental factors on the performance of sethoxydim against *Elymus repens* (L.). Weed Res. 1987 Oct;27(5):329-36. https://doi.org/10.1111/j.1365-3180.1987.tb00770.x
28. Derr J, Serensits T. Impact of application temperature on broadleaf herbicide efficacy. J Environ Hortic. 2016 Dec;34(4):123-30. https://doi.org/10.24266/0738-2898-34.4.123
29. Ganie ZA, Jugulam M, Jhala AJ. Temperature influences efficacy, absorption, and translocation of 2,4-D or glyphosate in glyphosate-resistant and glyphosate-susceptible common ragweed (*Ambrosia artemisiifolia*) and giant ragweed (*Ambrosia trifida*). Weed Sci. 2017 Sep;65(5):588-602. https://doi.org/10.1017/wsc.2017.32
30. Kumaratilake AR, Preston C. Low temperature reduces glufosinate activity and translocation in wild radish (*Raphanus raphanistrum*). Weed Sci. 2005 Feb;53(1):10-6. https://doi.org/10.1614/WS-03-140R

Freezing stress affects the efficacy of clodinafop-propargyl and 2,4-D plus MCPA on wild oat (*Avena ludoviciana* Durieu) and turnipweed [*Rapistrum rugosum* (L.) All.] in wheat (*Triticum aestivum* L.)

Short running title: Freezing and herbicide efficacy

Alireza Hasanfard[1], Mehdi Rastgoo[1], Ebrahim Izadi Darbandi[1], Ahmad Nezami[1], Bhagirath Singh Chauhan[2]

[1] Department of Agrotechnology, Faculty of Agriculture, Ferdowsi University of Mashhad, Iran

[2] Queensland Alliance for Agriculture and Food Innovation (QAAFI) *and* School of Agriculture and Food Sciences (SAFS), The University of Queensland, Gatton, Queensland 4343, Australia

***Corresponding author:** m.rastgoo@um.ac.ir

Abstract

The occurrence of freezing stress around herbicides application is one of the most important factors influencing their performance. This experiment was performed to evaluate the efficacy of clodinafop-propargyl and 2,4-D plus MCPA (2,4-Dichlorophenoxyacetic acid plus 2-methyl-4-chlorophenoxyacetic acid), the most important herbicides used in wheat fields in Iran, under the influence of a freezing treatment (-4°C). Wheat and its two common weeds, winter wild oat (*Avena ludoviciana* Durieu) and turnipweed [*Rapistrum rugosum* (L.) All.], were exposed to the freezing treatment for three nights from 7:00 P.M. to 5:00 A.M. before and after herbicide application, and their response was compared with plants that did not grow under freezing stress. Under no freezing (NF) and freezing after spray (FAS) conditions, winter wild oat was completely controlled with the recommended dose of clodinafop-propargyl (64 g ai ha^{-1}; hereafter g ha^{-1}). However, the survival percentage of winter wild oat in the freezing before spray (FBS) of clodinafop-propargyl 64 g ha^{-1} was 7%, and it was completely controlled with twice the recommended dose (128 g ha^{-1}). Under NF conditions and FAS treatment, turnipweed was completely controlled with twice the recommended dose of 2,4-D plus MCPA (2025 g ae ha^{-1}; hereafter g ha^{-1}). However, in the FBS treatment, the survival of turnipweed was 7% (2025 g ha^{-1}). The LD_{50} (dose required to control 50% of individuals in the population) and GR_{50} (dose causing 50% growth reduction of plants) rankings were NF<FBS<FAS for clodinafop-propargyl and NF<FAS<FBS for 2,4-D plus MCPA. Selectivity index for clodinafop-propargyl in NF conditions, FBS, and FAS treatments was 2.4, 0.91, and 0.78, respectively, and, for 2,4-D plus MCPA, it was 2.6, 0.12, and 0.88, respectively. According to the results of this experiment, it can be stated that the freezing stress after the spraying of clodinafop-propargyl and the freezing stress before a spray of 2,4-D plus MCPA would further reduce the efficacy of these herbicides.

Keywords: GR_{50}, Herbicide efficacy, LD_{50}, Selectivity index

Introduction

Winter wild oat (*Avena ludoviciana* Durieu) and turnipweed [*Rapistrum rugosum* (L.) All.] are the most important annual weeds of winter wheat (*Triticum aestivum* L.) in Iran, which cause significant reductions in wheat yield [1, 2]. The high reproduction potential and fitness to freezing stress, which results in a wide dispersal of weed seeds are the major challenges in the management of winter annual weeds like wild oat and turnipweed. Therefore, in cold regions of Iran, the dispersal and geographical distribution of these weeds are more significant than other weed species [3].

One of the most important consequences of climate change is the change in glaciers' occurrence patterns [4]. A sharp drop in temperature after high temperatures leads to crop damage. Increasing autumn temperatures in Iran, resulting from climate change, have led to reduced freezing tolerance of autumn crops [3]. In other words, autumn crops will not tolerate freezing stress due to the lack of hardening process in autumn. As a result, they will be damaged by decreasing temperatures during winter, and their yield will be reduced [3, 4]. It has been reported that increasing autumn temperatures resulting from climate change in western Canada led to reduced freezing tolerance of winter cereals [5]. In other words, due to the lack of optimal cold acclimation in autumn, crops will not be able to tolerate freezing stress. As a result, they will be damaged by decreasing temperatures during winter or by the occurrence of late spring frosts [6]. However, weeds generally have a higher ability to tolerate freezing stress [7]. Based on this, it can be inferred that climate change is adversely changing agricultural production and can significantly change the management of current cropping systems that have evolved under optimum climatic conditions.

Hand weeding and hoeing are not common in wheat fields, and mechanical weed management methods are not effective, so chemical methods are mainly used to control weeds in the wheat fields in Iran. Clodinafop-propargyl and 2,4-D plus MCPA (2,4-Dichlorophenoxyacetic acid plus 2-methyl-4-chlorophenoxyacetic acid) are the most common herbicides used for controlling weeds in wheat fields in Iran. Due to the particular importance of this crop in food security, their wide excessive applications can be justified [8, 9]. Clodinafop-propargyl is a systemic and selective herbicide of the aryloxyphenoxypropionate (AOPP) group and used post-emergent to control annual grass weeds in wheat. This herbicide inhibits the activity of acetyl-coenzyme A carboxylase (ACCase) and thus disrupts the biosynthesis of fatty acids [10]. 2,4-D plus MCPA, synthetic auxin herbicides, is a systemic herbicide mixture registered for selective post-emergence control of annual broadleaf weeds in wheat [11].

The efficacy of herbicides is significantly affected by environmental conditions before, during, and after application [12]. It has been reported that environmental factors such as light intensity, temperature, water stress, relative humidity, and nutrient availability alter the herbicide efficacy [13, 14]. On the other hand, plant species and their growth stages also affect herbicide efficacy [15]. Numerous studies indicate that ambient temperature is one of the most influential factors affecting the performance of herbicides [14, 16, 17]. Increasing the temperature is likely to increase the absorption and translocation of some herbicides and increase their performance. In contrast, the improvement in herbicide performance at low temperatures may be due to a reduction in the rate of herbicide metabolism [17, 18]. In cold regions, freezing temperatures occur around the time of herbicide application. Therefore, the occurrence of severe freezing that causes damage to plant leaves will have adverse effects on the activity and efficacy of foliar-applied herbicides [19]. On the other hand, application of herbicides in unfavorable

environmental conditions (such as adverse temperature before, during, and after herbicide application) causes stress in the crop, and this changes the selectivity index of the herbicide, which will ultimately cause damage to the crop [20]. The selectivity index refers to the potential of a herbicide to eliminate weeds in a crop without affecting the yield or quality of the final product [21].

At the appropriate growth stage of weeds for herbicide application, the temperature usually decreases, and this issue leads to a decrease in the performance of herbicides. Due to the lack of information on the efficacy of herbicides under freezing stress, this experiment was conducted to evaluate the efficacy of clodinafop-propargyl and 2,4-D plus MCPA on wild oat and turnipweed under freezing stress before and after herbicide application.

Materials and methods

Experimental site and plant material

Seeds of wheat (cv. Mihan) were harvested from Khorasan Razavi Agricultural and Natural Resources Research and Education Center, and seeds of winter wild oat and turnipweed were collected (collected from about 500 plants) from infested farms around Mashhad-Iran in May 2019. Experiments were conducted at the Faculty of Agriculture, the Ferdowsi University of Mashhad, Iran (Lat 36° 15′ N, Long 59° 28 E; 985 m Altitude). To break the dormancy of winter wild oat and turnipweed, their seeds were exposed to moist-chilling treatment and potassium nitrate (KNO_3) 0.2% solution for 3 days at 5°C in the dark, respectively [3]. Weed seeds were placed in 9-cm diameter Petri dishes (20-25 seeds per Petri dish) on moist filter papers and kept at 20°C. After 5 days, the germination percentage of winter wild oat and turnipweed seeds was >90%. The experiment was repeated twice with three replicates. To experience the same cold

acclimation, the plants were planted at approximately the same time on October 21 (first run) and 24 (second run) in 2019. Plastic pots (12 cm in diameter and 20 cm in height) were used in the trial, and pots were filled with a mixture of sand, agricultural soil, and peat moss (2:2:1 sand, agricultural soil, and peat moss, respectively). For uniform emergence, turnipweed germinated seeds were planted in a seedling tray. Eight turnipweed seedlings transferred to pots in the cotyledon stage. Eight germinated seeds of wild oat were planted at 4-5 cm depth below the soil surface. Wheat seeds were planted directly in each pot at the same depth. To induce cold acclimation, the plants were kept outdoors, grown in natural conditions (Fig. 1), and irrigated according to daily requirements until they were tested.

Fig 1. Minimum and maximum daily air temperatures at the experimental site from October 2019 to February 2020. Relative humidity of 40-50% under the natural photoperiod [3].

Experimental factors

Experimental factors included freezing stress at three conditions, including no freezing (NF), freezing before spray (FBS), and freezing after spray (FAS). Plant species included wheat, winter wild oat, and turnipweed. Herbicides were applied at 0, 6.25, 12.5, 25, 50, 100, and 200% of the recommended dose for the herbicide clodinafop-propargyl (0.8 L ha^{-1}; Topik, 8% EC) and 2,4-D plus MCPA (1.5 L ha^{-1}; U 46 combi fluid, 67.5% SL).

Freezing treatment and herbicide application

Under NF, the plants grew completely in natural winter conditions without exposing to freezing stress. For FBS, the plants grew up to the 3-4 leaf stage under natural conditions. They were exposed to freezing from 7:00 P.M. to 5:00 A.M. for three nights before herbicide application [19]. Freezing treatment was performed using a thermogradient freezer (Weiss Technik). The

initial temperature of the freezer was 5℃; however, the freezer was cooled down at a rate of 2℃ per hour to reach a temperature of -4℃. The temperature drop slope was adjusted based on the long-term meteorological statistics of Mashhad in the time range of herbicide application. The pots were kept at -4℃ for 1 h and then removed from the freezer (Fig. 2).

Fig. 2 Trend of temperature decrease in thermogradient freezer during the night from 7:00 P.M. to 5:00 A.M.

Each morning at 5:30 A.M., the plants were transferred out of the freezer, kept in natural conditions until 6:30 P.M., and then returned to the freezer during the night. After three nights of freezing exposure, the pots were taken out of the freezer and sprayed with herbicides after 2 h [19]. After spraying, the plants were kept outside under natural conditions for the duration of the experiment.

In the FAS treatment, freezing stress was applied for three nights by placing the samples treated with herbicides in the thermogradient freezer. For this purpose, the plants were taken out of the freezer at 5:30 A.M., transferred to natural conditions, and then returned to the freezer at 6:30 P.M. for the overnight freezing treatment (Similar to FBS treatment). Three nights after freezing treatment, the plants were transferred to natural conditions to continue the trial process (Fig. 3).

Fig. 3 Schematic diagram of the experimental study design. Abbreviation: FBS (freezing before spray); FAS (freezing after spray).

The conditions inside the thermogradient freezer were the same for both freezing treatments before and after spray. The experiment was performed in the freezer in completely dark conditions and at the same relative humidity (35±5%) for all treatments. Temperature changes in

the freezer were regularly monitored when the samples were in the freezer. For all the freezing treatments, herbicides were applied at the same time. Plants were transferred to natural conditions after three nights of freezing treatment at 5:30 A.M. Also, plants related to freezing after spray were transferred to the freezer at 6:30 P.M. On the same day, plants that were not exposed to freezing were sprayed from 7:30 A.M. to 10.30 A.M. The herbicides were applied by a researcher-made moving boom sprayer equipped with a TeeJet 8002 flat fan nozzle (spray output volume of 200 L ha^{-1} at a pressure of 260 kPa). Plants were returned and maintained outdoor under natural conditions of winter after the treatment.

Measurement of traits

Four weeks after herbicide treatments, survival percentage (Eq. 1) and shoot dry weight were measured by placing the samples in an oven at 75℃ for 48 h. Plants that produced new leaves were considered alive and those that experienced severe chlorosis and stunted growth were recorded as dead [22].

$$Survival\ \% = \frac{N1}{N0} \times 100 \qquad \text{(Eq. 1)}$$

Where, N_1 and N_0 are the numbers of alive plants four weeks after herbicide treatment and the number of alive plants before herbicide treatment, respectively.

Dose–response experiments

The response of weed traits to the doses of herbicides tested was analyzed by nonlinear regression. For this purpose, the data were fitted to a three-parameter log-logistic equation (Eq. 2) [23].

$$Y = \frac{d}{1+e^{b(\log x - \log e)}} \quad \text{(Eq. 2)}$$

Where, Y is the response rate (survival % or dry weight) at dose x, d is the upper limit for Y, b is the slope of the curve around e, and e is the effective dose (g ai or ae ha^{-1}). Here, e is replaceable with LD_{50} (dose required to control 50% of individuals in the population) or GR_{50} (dose causing 50% dry weight reduction of plants).

Relative potency

Relative potency was used to determine herbicide efficacy under freezing stress and no freezing stress (Eq. 3) [24].

$$Relative\ potency = \frac{ED_{50A}}{ED_{50B}} \quad \text{(Eq. 3)}$$

Where, ED_{50A} is the dose required to reduce survival by 50% in NF, and ED_{50B} is the dose needed to reduce survival by 50% in FBS or FAS. Relative potency = 1 indicates that freezing treatment (FBS and FAS) did not affect herbicide efficacy. If relative potency > 1, it indicates that the herbicides are more efficient under freezing stress. Relative potency < 1 indicates the decreased effectiveness of the herbicides under freezing stress with respect to optimum conditions.

Selectivity index

The ratio between doses that caused 10% damage to crops and 90% damage to weeds was considered a selectivity index [24] and calculated using Eq. 4.

$$Selectivity\ index\ (10{,}90) = \frac{ED_{10\ (crop)}}{ED_{90\ (weed)}} \quad \text{(Eq. 4)}$$

Where, ED_{90} is 90% of the herbicide effect on weed survival (herbicide dose to kill 90% of the population), and ED_{10} is 10% of the herbicide impact on crop survival (a 10% effect on the crop). The higher the SI, the more selective the herbicide in the crop [24].

Statistical analysis

The experiments were conducted using a completely randomized design in factorial arrangement with three replications. Afterward, differences between treatments were compared with the standard error of mean (SEM) values. For data analysis, analysis of variance (ANOVA) was performed using the SAS software (v. 9.4, SAS Institute Inc, Cary, NC, USA). Data for both runs were pooled as no significant differences were observed between runs ($P > 0.05$). Homogeneity of variance was confirmed using Levene's test. Analysis of biometric data was performed by the R program (v. 4.0.4) [25]. Dose-response curves were also fitted with the asymmetrical sigmoid form. LD_{50} and GR_{50} were separated using 95% confidence intervals (CI).

Results

Clodinafop-propargyl

Survival percentage and LD_{50}

The dose-response experiments were similar for NF and FAS treatments at the recommended dose (Figure 4A). Thus, in both conditions, winter wild oat was completely controlled with the recommended dose (64 g ai ha^{-1}). However, the survival of seedlings in the FBS treatment was 7%, and to achieve complete control, 2-fold the recommended dose (128 g ha^{-1}) was needed. The LD_{50} in NF, FBS, and FAS was 17.9, 18.1, and 23.2 g ha^{-1}, respectively (Table 1).

Fig. 4 Survival (A) and dry weight (B) of winter wild oat treated with various doses of clodinafop-propargyl under different freezing treatments. Abbreviation: NF (no freezing); FBS (freezing before spray): FAS (freezing after spray). The recommended dose of clodinafop-propargyl in Iran is 64 g ha^{-1}.

Table 1 The dose of clodinafop-propargyl and 2,4-D plus MCPA required for 50% mortality (LD_{50}) and the dose that caused 50% inhibition of growth noted by biomass (GR_{50}) of winter wild oat and turnipweed under different freezing treatments.

Herbicide	Freezing Treatment	b±SE	d±SE	LD_{50} ±SE	95% CI	R^2
			%	g ai or ae ha^{-1}		
Clodinafop-propargyl	NF	$2.0 \pm 0.19^{**}$	$100.3 \pm 2.8^{**}$	$17.9 \pm 1.2^{**b}$	15.6-20.2	0.99
	FBS	$1.9 \pm 0.17^{**}$	$101.0 \pm 2.9^{**}$	$18.1 \pm 1.2^{**b}$	15.7-20.5	0.99
	FAS	$1.9 \pm 0.19^{**}$	$98.7 \pm 2.9^{**}$	$23.2 \pm 1.7^{**a}$	19.8-26.6	0.96
2,4-D plus MCPA	NF	$1.9 \pm 0.20^{**}$	$102.2 \pm 3.2^{**}$	$292.8 \pm 21.9^{**c}$	249.4-336.3	0.98
	FBS	$1.5 \pm 0.15^{**}$	$102.4 \pm 3.2^{**}$	$472.8 \pm 42.4^{**a}$	388.8-556.8	0.98
	FAS	$1.5 \pm 0.14^{**}$	$102.8 \pm 3.5^{**}$	$318.8 \pm 28.9^{**b}$	261.6-376.1	0.98
		b±SE	d±SE	GR_{50} ±SE	95% CI	
			g pot^{-1}	g ai or ae ha^{-1}		
Clodinafop-propargyl	NF	$2.1 \pm 0.15^{**}$	$0.70 \pm 0.02^{**}$	$10.6 \pm 0.52^{**c}$	9.6-11.6	0.99
	FBS	$2.0 \pm 0.12^{**}$	$0.80 \pm 0.02^{**}$	$11.6 \pm 0.51^{**b}$	10.6-12.6	0.99
	FAS	$1.7 \pm 0.12^{**}$	$0.67 \pm 0.02^{**}$	$12.8 \pm 0.74^{**a}$	11.3-14.3	0.98
2,4-D plus MCPA	NF	$2.2 \pm 0.23^{**}$	$0.46 \pm 0.01^{**}$	$176.2 \pm 11.3^{**c}$	153.9-198.6	0.99
	FBS	$1.7 \pm 0.16^{**}$	$0.48 \pm 0.01^{**}$	$298.6 \pm 21.5^{**a}$	256.0-341.3	0.99
	FAS	$1.6 \pm 0.15^{**}$	$0.47 \pm 0.01^{**}$	$207.8 \pm 15.9^{**b}$	176.2-239.4	0.99

Abbreviation: NF (no freezing); FBS (freezing before spray); FAS (freezing after spray); SE: Standard Error. b is the slope of the curve around e (LD_{50} and GR_{50}); d is the upper limit for Y (survival % or dry weight); **: significant at p≤0.01. LD_{50} and GR_{50} were separated using their 95% confidence intervals (CI). The data shows the parameter's mean ± SE. The data (LD_{50} and GR_{50}) with the different letters indicate significant differences according to LSD at P ≤0.05. n = 6

Dry weight and GR_{50}

The dry weight of winter wild oat in NF and FAS with the recommended dose (64 g ha^{-1}) and FBS with twice the recommended dose (128 g ha^{-1}) reached 0 g (Fig. 4B). The GR_{50} of plants not exposed to freezing stress was 10.6 g ha^{-1}. The GR_{50} was 11.6 and 12.8 g ha^{-1} in FBS and FAS, respectively (Table 1).

2,4-D plus MCPA

Survival percentage and LD_{50}

The dose-response experiments of 2,4-D plus MCPA (similar to clodinafop-propargyl) were the same in NF and FAS treatments (Fig. 5A). In both conditions, turnipweed was completely controlled with twice the recommended dose (2025 g ae ha^{-1}). However, in the FBS treatment, the survival of turnipweed was 7%. At the recommended dose (1012.5 g ha^{-1}), the survival of turnipweed in NF, FBS, and FAS was 3%, 33%, and 20%, respectively. The LD_{50} in NF, FBS, and FAS was 293, 473, and 319 g ha^{-1}, respectively (Table 1).

Fig. 5 Survival (A) and dry weight (B) of turnipweed treated with various doses of 2,4-D plus MCPA under different freezing treatments. Abbreviation: NF (no freezing); FBS (freezing before spray): FAS (freezing after spray). The recommended dose of 2,4-D+MCPA in Iran is 1012.5 g ha^{-1}.

Dry weight and GR_{50}

The dry weight of turnipweed in NF and FAS with twice the recommended dose (2025 g ha^{-1}) of 2,4-D plus MCPA was reduced to 0 (Fig. 5 B). However, in the FBS treatment, the dry weight of turnipweed with twice the recommended dose was 0.012 g per pot. At the recommended dose, the dry weight of turnipweed in FBS was 97% and 76% more than NF and FAS, respectively. The GR_{50} of NF, FBS, and FAS were 176, 299, and 208 g ha^{-1}, respectively (Table 1).

Relative potency

The relative potency of clodinafop-propargyl and 2,4-D plus MCPA was significantly reduced in FBS and FAS compared to NF (Fig. 6). Thus, the relative potency in FBS and FAS of clodinafop-propargyl was 0.99 and 0.77, respectively, and at 2,4-D plus MCPA was 0.62 and 0.92, respectively.

Fig. 6 Relative potency of clodinafop-propargyl and 2,4-D plus MCPA under different freezing treatments. Abbreviation: FBS (freezing before spray) and FAS (freezing after spray). The error bars indicate standard error (SE). The dashed line represents the relative potency = 1.0.

Selectivity index

As shown in Table 2, selectivity index values for clodinafop-propargyl in NF, FBS, and FAS treatments were 2.4, 0.91, and 0.78, respectively, and, for 2,4-D plus MCPA, they were 2.6, 0.12, and 0.88, respectively. For clodinafop-propargyl, the LD_{10} of wheat in NF was 127.9 g ha^{-1}. However, LD_{10} of wheat in the FBS and FAS was 53.5 and 57.1 g ha^{-1}, respectively (Table 2). For 2,4-D plus MCPA, the LD_{10} of wheat in NF, FBS, and FAS was 2380, 264, and 1236 g ha^{-1}, respectively.

Table 2 Sensitivity of plant species of wheat, winter wild oat, and turnipweed to clodinafop-propargyl and 2,4-D plus MCPA under different freezing treatments.

Freezing Treatment	Plant species	Clodinafop-propargyl: LD_{10} (wheat) and LD_{90} (winter wild oat) (g ai ha^{-1}) ± SE	Clodinafop-propargyl: SI	2,4-D plus MCPA: LD_{10} (wheat) and LD_{90} (turnipweed) (g ae ha^{-1}) ± SE	2,4-D plus MCPA: SI
NF	Wheat	127.9 ± 8.8	-	2380 ± 455	-
	Weed	53.5 ± 5.4	2.4	908 ± 108	2.6
FBS	Wheat	53.5 ± 11.0	-	264 ± 61.0	-
	Weed	59.0 ± 6.4	0.91	2144 ± 327	0.12
FAS	Wheat	57.1 ± 8.7	-	1236 ± 163	-
	Weed	73.6 ± 7.2	0.78	1400 ± 199	0.88

Abbreviation: NF; No freezing, FBS: Freezing before spray, FAS: Freezing after spray SI: Selectivity index. The selectivity index (SI) was calculated as follows: *Selectivity index (10, 90):* $\frac{ED10\ (crop)}{ED90\ (weed)}$

Discussion

The required dose of clodinafop-propargyl for complete control of winter wild oat seedlings in FBS treatment was twice that of NF and FAS treatments. The trend of the reducing survival % in seedlings related to the FBS treatment and NF was similar. The LD_{50} for clodinafop-propargyl in freezing treatments shows that exposure of winter wild oat seedlings to freezing treatment for three nights before and after spray reduces the efficacy of this herbicide compared to NF. The LD_{50} was 22% and 23% higher in the FAS compared to the FBS and NF, respectively. The slight difference between NF and FBS can be due to individual variability. Freezing treatment for three nights before and after spraying of clethodim herbicide (the mode of action similar to clodinafop-propargyl) led to an increase in LD_{50} [19]. It decreased herbicide efficacy in rigid ryegrass (*Lolium rigidum* Gaud.) populations compared to NF.

The dose of clodinafop-propargyl required to reduce winter wild oat biomass to zero in the FBS treatment was twice that of the NF and FAS treatments. The GR_{50} is more pronounced in FBS and FAS compared to NF, indicating a decrease in the efficacy of clodinafop-propargyl. The GR_{50} was 17% and 8% higher in the FAS treatment compared to NF and FBS, respectively. In other words, the occurrence of three nights of freezing stress after herbicide application reduced the performance of clodinafop-propargyl more than other treatments. It has been reported that wild oat plants (*Avena fatua*) that grew at low temperatures were less sensitive to difenzoquat than plants that grew at high temperatures [26].

Our results clearly show that the occurrence of freezing stress around spray reduces the efficacy of clodinafop-propargyl, especially in the treatment of FAS. Herbicide translocation in the phloem in freezing conditions could decline due to damage to the phloem cells, which may lead to a decrease in the herbicide efficacy. Decreased efficacy of sethoxydim at low

temperatures due to reduced herbicide translocation for Quackgrass [*Elymus repens* (L.) Gould] control was reported by Coupland [27]. In general, to maintain the herbicide efficacy under cold conditions, using clodinafop-propargyl after the freezing stress can be recommended in areas with a similar climate.

Although the three-night treatment of FBS and FAS resulted in a reduction in turnipweed control by 2,4-D plus MCPA compared to NF, this reduction was different in the two freezing treatments. At the recommended dose (1013 g ha^{-1}) in NF, almost all turnipweed plants were controlled, while the survival percentage of turnipweed in FBS and FAS treatments were 33% and 20%, respectively. Low temperatures around the time of herbicide application reduced the effectiveness of systemic herbicides [28]. Based on LD_{50} values, the efficacy of 2,4-D plus MCPA under FBS and FAS was 1.6 and 1.1 times lower compared to NF.

FAS is possible to damage the phloem cells, which reduces the translocation of the herbicide. The efficacy of 2,4-D plus MCPA in the treatment of FBS was reduced. The decrease in the effect of 2,4-D plus MCPA in the treatment of FBS is probably due to the reduction of absorption and translocation of this herbicide in turnipweed seedlings. In this regard, an increase in efficacy of 2,4-D and glyphosate at high temperatures compared to low temperatures in common ragweed (*Ambrosia artemisiifolia* L.) and giant ragweed (*Ambrosia trifida* L.) due to the increased absorption and/or translocation at high temperatures around herbicide application has been reported by Ganie et al. [29]. It has been also reported that low temperatures reduced glufosinate activity and translocation in wild radish (*Raphanus raphanistrum* L.) [30].

Turnipweed's dry weight at the recommended dose of 2,4-D plus MCPA (1012.5 g ha^{-1}) in FBS and FAS treatments was 35.5 and 8.5 times higher than the NF, respectively. Also, GR_{50} in FBS and FAS was 41% and 15.2% higher compared to NF, respectively. Although a decrease

in herbicide efficacy is evident in both FBS and FAS treatments, high GR_{50} in FBS shows an adverse effect of three nights of freezing before the application of 2,4-D plus MCPA. In other words, the efficacy of this herbicide reacts more to FBS than to FAS. In a similar study, the GR_{50} for 2,4-D increased at low temperature in common ragweed compared to high temperature [29].

The freezing stress around 2,4-D plus MCPA application reduced the efficacy of this herbicide in FBS and FAS treatments compared to NF. The decrease in the efficacy of this herbicide in the treatment of FBS is probably due to the reduction of absorption and translocation of the herbicide in the phloem during freezing. Absorption and translocation of 2,4-D plus MCPA might be reduced at low temperatures due to the effect on herbicide penetration facilitated by physicochemical factors, including the decreased rate of diffusion, increased viscosity of the cuticle, and physiological factors comprising decreases in photosynthesis, phloem translocation, and protoplasmic streaming [29]. In Iranian wheat fields, using the 2,4-D plus MCPA before the freezing stress can be recommended to maintain the herbicide efficacy under cold conditions.

When relative potency > 1, the herbicide efficacy is higher than optimal non-freezing conditions, and when relative potency < 1, the herbicide efficacy is lower than usual [24]. The relative potency values of clodinafop-propargyl and 2,4-D plus MCPA were lower than one under both FBS and FAS treatments in the present experiment. In other words, freezing stress reduced the efficacy of these herbicides. Based on the results of relative potency, the reduction of clodinafop-propargyl efficacy in the FAS treatment (33%) was more significant than in the FBS treatment (1%). Conversely, for 2,4-D plus MCPA, the reduction in efficacy was more significant in the FBS treatment (38%) compared to the FAS treatment (8%).

The selectivity index for clodinafop-propargyl and 2,4-D plus MCPA was > 1 in NF, indicating that both herbicides acted selectively in wheat. However, applying three nights of FBS and FAS of both herbicides reduced the selectivity index to less than one. In other words, freezing stress reduced the selectivity index of both herbicides, which led to phytotoxicity in wheat. For clodinafop-propargyl, LD_{10} of wheat in NF was 58% and 55% higher than FBS and FAS, respectively. The LD_{10} values of 2,4-D plus MCPA in NF were 89% and 48% higher compared to FBS and FAS, respectively. In other words, the dose required to kill 10% of wheat in both herbicides under NF was higher than FBS and FAS. Therefore, FBS and FAS increased the sensitivity of wheat to herbicide application. Freezing stress may reduce the rate of herbicide metabolism in the crop and ultimately increase its susceptibility [17].

Conclusions

The relative potency results for both herbicides were used to confirm the results of LD_{50} and GR_{50}. Thus, LD_{50} and GR_{50} were higher for clodinafop-propargyl in the FAS treatment than in NF and FBS treatments. The LD_{50} and GR_{50} were higher for the 2,4-D plus MCPA in the FBS treatment than in the NF and FAS treatments. In other words, clodinafop-propargyl was more effective than other conditions in the treatment of three nights of FAS and 2,4-D plus MCPA in the treatment of three nights of FBS. The selectivity index for clodinafop-propargyl was lower in the FAS treatment (0.78) and for 2,4-D plus MCPA was lower in the FBS treatment (0.12). The efficacy of both herbicides was affected by the FBS and FAS treatments. The efficacy of clodinafop-propargyl in the FAS treatment and the efficacy of 2,4-D plus MCPA in the FBS treatment were reduced. The values of the selectivity index of herbicides in the conditions of freezing stress showed that this index could be well used to determine the selective performance of these herbicides. Overall, according to the results of this experiment, it can be stated that FAS

with ACCase inhibitor herbicides, such as clodinafop-propargyl, may reduce their effectiveness in controlling narrow leaf weeds such as winter wild oat, and FBS with auxin type herbicides, such as 2,4-D plus MCPA, reduce their efficacy in controlling broadleaf weeds such as turnipweed. FBS for clodinafop-propargyl and FAS for 2,4-D plus MCPA had a slight adverse effect on the herbicide efficacy and selectivity index. Therefore, the use of these herbicides in the mentioned conditions (FBS for clodinafop-propargyl and FAS for 2,4-D plus MCPA) is recommended. In other words, to maintain high herbicide efficacy under cold (freezing) conditions, the use of clodinafop-propargyl after the freezing stress and 2,4-D plus MCPA before the freezing stress can be recommended in areas with a similar climate to Mashhad. Finally, the use of adjuvants, alternate herbicides, and various herbicide formulations to control grass and broadleaf weeds in Iranian wheat production is suggested to prevent the adverse effects of freezing stress on herbicides.

Conflicts of interest

The authors declare that they have no conflict of interest.

Acknowledgment

We are grateful to Ferdowsi University of Mashhad, Iran, for providing financial support for present research (Project No.3.49759). No additional external funding was received for this study.

References

1. Hatami ZM, Gherekhloo J, Rojano-Delgado AM, Osuna MD, Alcántara R, Fernández P, et al. Multiple mechanisms increase levels of resistance in *Rapistrum rugosum* to ALS herbicides. Front Plant Sci. 2016 Feb 22;7:169. https://doi.org/10.3389/fpls.2016.00169
2. Nosratti I, Sabeti P, Chaghamirzaee G, Heidari H. Weed problems, challenges, and opportunities in Iran. Crop Prot. 2020 Aug 1;134:104371. https://doi.org/10.1016/j.cropro.2017.10.007
3. Hasanfard A, Rastgoo M, Darbandi EI, Nezami A, Chauhan BS. Regeneration capacity after exposure to freezing in wild oat (*Avena ludoviciana* Durieu.) and turnipweed

(*Rapistrum rugosum* (L.) All.) in comparison with winter wheat. Environ. Exp. Bot. 2021 Jan 1;181:104271. https://doi.org/10.1016/j.envexpbot.2020.104271

4. Sierra-Almeida A, Cavieres LA, Bravo LA. Warmer temperatures affect the in situ freezing resistance of the Antarctic vascular plants. Front. Plant Sci. 2018 Oct 8;9:1456. https://doi.org/10.3389/fpls.2018.01456
5. Willick IR, Tanino KK, Gusta LV. The impact of global climate change on the freezing tolerance of winter cereals in Western Canada. J. Agron. Crop Sci. 2021 Feb;207(1):88-99. https://doi.org/10.1111/jac.12447
6. Vyse K, Pagter M, Zuther E, Hincha DK. Deacclimation after cold acclimation—a crucial, but widely neglected part of plant winter survival. J. Exp. Bot. 2019 Sep 15;70(18):4595-604. https://doi.org/10.1093/jxb/erz229
7. Cici SZ, Van Acker RC. Relative freezing tolerance of facultative winter annual weeds. Can. J. Plant Sci. 2011;91(4):759-63. https://doi.org/10.4141/cjps2010-001
8. Baghestani MA, Zand E, Soufizadeh S, Beheshtian M, Haghighi A, Barjasteh A, Birgani DG, Deihimfard R. Study on the efficacy of weed control in wheat (*Triticum aestivum* L.) with tank mixtures of grass herbicides with broadleaved herbicides. Crop Prot. 2008 Jan 1;27(1):104-11. https://doi.org/10.1016/j.cropro.2007.04.013
9. Enghiad A, Ufer D, Countryman AM, Thilmany DD. An overview of global wheat market fundamentals in an era of climate concerns. Int J Agron. 2017 Jul 2;2017. https://doi.org/10.1155/2017/3931897
10. Bajwa AA, Akhter MJ, Iqbal N, Peerzada AM, Hanif Z, Manalil S, Hashim S, Ali HH, Kebaso L, Frimpong D, Namubiru H. Biology and management of Avena fatua and Avena ludoviciana: two noxious weed species of agro-ecosystems. Environ Sci Pollut Res. 2017 Aug;24(24):19465-79. https://doi.org/10.1007/s11356-017-9810-y
11. Peterson MA, McMaster SA, Riechers DE, Skelton J, Stahlman PW. 2, 4-D past, present, and future: a review. Weed Technol. 2016 Jun;30(2):303-45. https://doi.org/10.1614/WT-D-15-00131.1
12. Collings LV, Blair AM, Gay AP, Dyer CJ, Mackay N. The effect of weather factors on the performance of herbicides to control *Alopecurus myosuroides* in winter wheat. Weed Res. 2003 Apr;43(2):146-53. https://doi.org/10.1046/j.1365-3180.2003.00327.x
13. Rocha-Pereira MR, Klar AE, Martins D, Ferreira de Souza GS, Villalba J. Efecto del estrés hídrico sobre la eficacia de los herbicidas aplicados en *Urochloa decumbens*. Cienc. Investig Agrar. 2012 Apr;39(1):211-20. http://dx.doi.org/10.4067/S0718-16202012000100018
14. Johnson BC, Young BG. Influence of temperature and relative humidity on the foliar activity of mesotrione. Weed Sci. 2002 Apr;50(2):157-61. https://doi.org/10.1614/0043-1745(2002)050[0157:IOTARH]2.0.CO;2.
15. Chahal PS, Aulakh JS, Rosenbaum K, Jhala AJ. Growth stage affects dose response of selected glyphosate-resistant weeds to premix of 2,4-D choline and glyphosate (Enlist Duo (TM) herbicide*). J. Agric. Sci. 2015 Nov 1;7(11):1. https://doi.org/10.5539/jas.v7n11p1
16. Waltz AL, Martin AR, Roeth FW, Lindquist JL. Glyphosate efficacy on velvetleaf varies with application time of day. Weed Technol. 2004 Dec;18(4):931-9. https://doi.org/10.1614/WT-03-123R3

17. Godar AS, Varanasi VK, Nakka S, Prasad PV, Thompson CR, Mithila J. Physiological and molecular mechanisms of differential sensitivity of Palmer amaranth (*Amaranthus palmeri*) to mesotrione at varying growth temperatures. Plos One. 2015 May 19;10(5):e0126731. https://doi.org/10.1371/journal.pone.0126731
18. Pline WA, Wu J, Hatzios KK. Effects of temperature and chemical additives on the response of transgenic herbicide-resistant soybeans to glufosinate and glyphosate applications. Pestic. Biochem. Phys. 1999 Oct 1;65(2):119-31. https://doi.org/10.1006/pest.1999.2437
19. Saini RK, Malone J, Preston C, Gill GS. Frost reduces clethodim efficacy in clethodim-resistant rigid ryegrass (*Lolium rigidum*) populations. Weed Sci. 2016 Jun;64(2):207-15. https://doi.org/10.1614/WS-D-15-00140.1
20. Moseley C, Hatzios KK, Hagood ES. Uptake, translocation, and metabolism of chlorimuron in soybean (*Glycine max*) and morningglory (*Ipomoea* spp.). Weed Tech. 1993 Jun;7(2):343-8. https://doi.org/10.1017/S0890037X00027706
21. Negrisoli E, Velini ED, Tofoli GR, Cavenaghi AL, Martins D, Morelli JL, Costa AG. Selectivity of pre-emergence herbicides to sugarcane treated with nematicides. Planta Daninha. 2004 Dec;22(4):567-75.
22. Powles SB, Lorraine-Colwill DF, Dellow JJ, Preston C. Evolved resistance to glyphosate in rigid ryegrass (*Lolium rigidum*) in Australia. Weed Sci. 1998 Oct;46(5):604-7.
23. Ritz C, Baty F, Streibig JC, Gerhard D. Dose-response analysis using R. Plos One. 2015 Dec 30;10(12):e0146021. https://doi.org/10.1371/journal.pone.0146021
24. Tind T, Mathiesen TJ, Jensen JE, Ritz C, Streibig JC. Using a selectivity index to evaluate logarithmic spraying in grass seed crops. Pest Manag Sci: formerly Pesticide Science. 2009 Nov;65(11):1257-62. https://doi.org/10.1002/ps.1819
25. Team RC. R Foundation for Statistical Computing, Vienna, available at: www. R-project. org. 2018.
26. Kowalczyk B, Caseley JC, McCready CC. The effect of pre-treatment temperature on some physiological responses of *Avena fatua* to difenzoquat and benzoylpropethyl. Asp Appl Biol. 1983;4:235-44.
27. Coupland D. Influence of environmental factors on the performance of sethoxydim against *Elymus repens* (L.). Weed Res. 1987 Oct;27(5):329-36. https://doi.org/10.1111/j.1365-3180.1987.tb00770.x
28. Derr J, Serensits T. Impact of application temperature on broadleaf herbicide efficacy. J Environ Hortic. 2016 Dec;34(4):123-30. https://doi.org/10.24266/0738-2898-34.4.123
29. Ganie ZA, Jugulam M, Jhala AJ. Temperature influences efficacy, absorption, and translocation of 2,4-D or glyphosate in glyphosate-resistant and glyphosate-susceptible common ragweed (*Ambrosia artemisiifolia*) and giant ragweed (*Ambrosia trifida*). Weed Sci. 2017 Sep;65(5):588-602. https://doi.org/10.1017/wsc.2017.32
30. Kumaratilake AR, Preston C. Low temperature reduces glufosinate activity and translocation in wild radish (*Raphanus raphanistrum*). Weed Sci. 2005 Feb;53(1):10-6. https://doi.org/10.1614/WS-03-140R

Freezing stress affects the efficacy of clodinafop-propargyl and 2,4-D plus MCPA on wild oat (*Avena ludoviciana* Durieu) and turnipweed [*Rapistrum rugosum* (L.) All.] in wheat (*Triticum aestivum* L.)

Short running title: Freezing and herbicide efficacy

Alireza Hasanfard[1], Mehdi Rastgoo[1], Ebrahim Izadi Darbandi[1], Ahmad Nezami[1], Bhagirath Singh Chauhan[2]

[1] Department of Agrotechnology, Faculty of Agriculture, Ferdowsi University of Mashhad, Iran

[2] Queensland Alliance for Agriculture and Food Innovation (QAAFI) *and* School of Agriculture and Food Sciences (SAFS), The University of Queensland, Gatton, Queensland 4343, Australia

***Corresponding author:** m.rastgoo@um.ac.ir

Abstract

The occurrence of freezing stress around herbicides application is one of the most important factors influencing their performance. This experiment was performed to evaluate the efficacy of clodinafop-propargyl and 2,4-D plus MCPA (2,4-Dichlorophenoxyacetic acid plus 2-methyl-4-chlorophenoxyacetic acid), the most important herbicides used in wheat fields in Iran, under the influence of a freezing treatment (-4℃). Wheat and its two common weeds, winter wild oat (*Avena ludoviciana* Durieu) and turnipweed [*Rapistrum rugosum* (L.) All.], were exposed to the freezing treatment for three nights from 7:00 P.M. to 5:00 A.M. before and after herbicide application, and their response was compared with plants that did not grow under freezing stress. Under no freezing (NF) and freezing after spray (FAS) conditions, winter wild oat was completely controlled with the recommended dose of clodinafop-propargyl (64 g ai ha^{-1}; hereafter g ha^{-1}). However, the survival percentage of winter wild oat in the freezing before spray (FBS) of clodinafop-propargyl 64 g ha^{-1} was 7%, and it was completely controlled with twice the recommended dose (128 g ha^{-1}). Under NF conditions and FAS treatment, turnipweed was completely controlled with twice the recommended dose of 2,4-D plus MCPA (2025 g ae ha^{-1}; hereafter g ha^{-1}), while there was no complete control under recommended rate. However, in the FBS treatment, the survival of turnipweed was 7% under double dose. The LD_{50} (dose required to control 50% of individuals in the population) and GR_{50} (dose causing 50% growth reduction of plants) rankings were NF<FBS<FAS for clodinafop-propargyl and NF<FAS<FBS for 2,4-D plus MCPA. Selectivity index for clodinafop-propargyl in NF conditions, FBS, and FAS treatments was 2.4, 0.91, and 0.78, respectively, and, for 2,4-D plus MCPA, it was 2.6, 0.12, and 0.88, respectively. According to the results of LD_{50}, it can be stated that the freezing stress after

the spraying of clodinafop-propargyl and before the spraying of 2,4-D plus MCPA would further reduce the efficacy of these herbicides.

Keywords: GR_{50}, Herbicide efficacy, LD_{50}, Selectivity index

Introduction

Winter wild oat (*Avena ludoviciana* Durieu) and turnipweed [*Rapistrum rugosum* (L.) All.] are the most important annual weeds of winter wheat (*Triticum aestivum* L.) in Iran, which cause significant reductions in wheat yield [1, 2]. The high reproduction potential and fitness to freezing stress, which results in a wide dispersal of weed seeds are the major challenges in the management of winter annual weeds like wild oat and turnipweed. Therefore, in cold regions of Iran, the dispersal and geographical distribution of these weeds are more significant than other weed species [3].

One of the most important consequences of climate change is the change in glaciers' occurrence patterns [4]. A sharp drop in temperature after high temperatures leads to crop damage. Increasing autumn temperatures in Iran, resulting from climate change, have led to reduced freezing tolerance of autumn crops [3]. In other words, autumn crops will not tolerate freezing stress due to the lack of hardening process in autumn. As a result, they will be damaged by decreasing temperatures during winter, and their yield will be reduced [3, 4]. It has been reported that increasing autumn temperatures resulting from climate change in western Canada led to reduced freezing tolerance of winter cereals [5]. In other words, due to the lack of optimal cold acclimation in autumn, crops will not be able to tolerate freezing stress. As a result, they will be damaged by decreasing temperatures during winter or by the occurrence of late spring frosts [6]. However, weeds generally have a higher ability to tolerate freezing stress [7]. Based

on this fact, it can be inferred that climate change is adversely changing agricultural production and can significantly change the management of current cropping systems that have evolved under optimum climatic conditions.

Hand weeding and hoeing are not common in wheat fields, and mechanical weed management methods are not effective, so chemical methods are mainly used to control weeds in the wheat fields in Iran. Clodinafop-propargyl and 2,4-D plus MCPA (2,4-Dichlorophenoxyacetic acid plus 2-methyl-4-chlorophenoxyacetic acid) are the most common herbicides used for controlling weeds in wheat fields in Iran. Due to the particular importance of this crop in food security, their wide excessive applications can be justified [8, 9]. Clodinafop-propargyl is a systemic and selective herbicide of the aryloxyphenoxypropionate (APP) group and used post-emergent to control annual grass weeds in wheat. This herbicide inhibits the activity of acetyl-coenzyme A carboxylase (ACCase) and thus disrupts the biosynthesis of fatty acids [10]. 2,4-D plus MCPA, synthetic auxin herbicides, is a systemic herbicide mixture registered for selective post-emergence control of annual broadleaf weeds in wheat [11].

The efficacy of herbicides is significantly affected by environmental conditions before, during, and after application [12]. It has been reported that environmental factors such as light intensity, temperature, water stress, relative humidity, and nutrient availability alter the herbicide efficacy [13, 14]. On the other hand, plant species and their growth stages also affect herbicide efficacy [15]. Numerous studies indicate that ambient temperature is one of the most influential factors affecting the performance of herbicides [14, 16, 17]. Increasing the temperature is likely to increase the absorption and translocation of some herbicides and increase their performance. In contrast, the improvement in herbicide performance at low temperatures may be due to a reduction in the rate of herbicide metabolism [17, 18]. In cold regions, freezing temperatures

occur around the time of herbicide application. Therefore, the occurrence of severe freezing that causes damage to plant leaves will have adverse effects on the activity and efficacy of foliar-applied herbicides [19]. On the other hand, application of herbicides in unfavorable environmental conditions (such as adverse temperature before, during, and after herbicide application) causes stress in the crop, and this changes the selectivity index of the herbicide, which will ultimately cause damage to the crop [20]. The selectivity index refers to the potential of a herbicide to eliminate weeds in a crop without affecting the yield or quality of the final product [21].

At the appropriate growth stage of weeds for herbicide application, the temperature usually decreases, and this issue leads to a decrease in the performance of herbicides. Due to the lack of information on the efficacy of herbicides under freezing stress, this experiment was conducted to evaluate the efficacy of clodinafop-propargyl and 2,4-D plus MCPA on wild oat and turnipweed under freezing stress before and after herbicide application.

Materials and methods

Experimental site and plant material

Seeds of wheat (cv. Mihan) were harvested from Khorasan Razavi Agricultural and Natural Resources Research and Education Center, and seeds of winter wild oat and turnipweed were collected (collected from about 500 plants) from infested farms around Mashhad-Iran in May 2019. Experiments were conducted at the Faculty of Agriculture, the Ferdowsi University of Mashhad, Iran (Lat 36° 15′ N, Long 59° 28 E; 985 m Altitude). To break the dormancy of winter wild oat and turnipweed, their seeds were exposed to moist-chilling treatment and potassium nitrate (KNO_3) 0.2% solution for 3 days at 5℃ in the dark, respectively [3]. Weed seeds were

placed in 9-cm diameter Petri dishes (20-25 seeds per Petri dish) on moist filter papers and kept at 20℃. After 5 days, the germination percentage of winter wild oat and turnipweed seeds was >90%. The experiment was repeated twice with three replicates. To experience the same cold acclimation, the plants were planted at approximately the same time on October 21 (first run) and 24 (second run) in 2019. Plastic pots (12 cm in diameter and 20 cm in height) were used in the trial, and pots were filled with a mixture of sand, agricultural soil, and peat moss (2:2:1 sand, agricultural soil, and peat moss, respectively). For uniform emergence, turnipweed germinated seeds were planted in a seedling tray. Eight turnipweed seedlings were transferred to pots in the cotyledon stage. Eight germinated seeds of wild oat were planted at 4-5 cm depth below the soil surface. Wheat seeds were planted directly in each pot at the same depth. To induce cold acclimation, the plants were kept outdoors, grown in natural conditions (Fig. 1), and irrigated according to daily requirements until they were tested.

Fig 1. Minimum and maximum daily air temperatures at the experimental site from October 2019 to February 2020. Relative humidity of 40-50% under the natural photoperiod [3].

Experimental factors

Experimental factors included freezing stress at three conditions, including no freezing (NF), freezing before spray (FBS), and freezing after spray (FAS). Plant species included wheat, winter wild oat, and turnipweed. Herbicides were applied at 0, 6.25, 12.5, 25, 50, 100, and 200% of the recommended dose for the herbicide clodinafop-propargyl (0.8 L ha^{-1}; Topik, 8% EC) and 2,4-D plus MCPA (1.5 L ha^{-1}; U 46 combi fluid, 67.5% SL).

Freezing treatment and herbicide application

Under NF, the plants grew completely in natural winter conditions without exposing to freezing stress. For FBS, the plants grew up to the 3-4 leaf stage under natural conditions. They were exposed to freezing from 7:00 P.M. to 5:00 A.M. for three nights before herbicide application [19]. Freezing treatment was performed using a thermogradient freezer (Weiss Technik). The initial temperature of the freezer was 5℃; however, the freezer was cooled down at a rate of 2℃ per hour to reach a temperature of -4℃. The temperature drop slope was adjusted based on the long-term meteorological statistics of Mashhad in the time range of herbicide application. The pots were kept at -4℃ for 1 h and then removed from the freezer (Fig. 2).

Fig. 2 Trend of temperature decrease in thermogradient freezer during the night from 7:00 P.M. to 5:00 A.M.

Each morning at 5:30 A.M., the plants were transferred out of the freezer, kept in natural conditions until 6:30 P.M., and then returned to the freezer during the night. After three nights of freezing exposure, the pots were taken out of the freezer and sprayed with herbicides after 2 h [19]. After spraying, the plants were kept outside under natural conditions for the duration of the experiment.

In the FAS treatment, freezing stress was applied for three nights by placing the samples treated with herbicides in the thermogradient freezer. For this purpose, the plants were taken out of the freezer at 5:30 A.M., transferred to natural conditions, and then returned to the freezer at 6:30 P.M. for the overnight freezing treatment (Similar to FBS treatment). Three nights after freezing treatment, the plants were transferred to natural conditions to continue the trial process (Fig. 3).

Fig. 3 Schematic diagram of the experimental study design. Abbreviation: FBS (freezing before spray); FAS (freezing after spray).

The conditions inside the thermogradient freezer were the same for both freezing treatments before and after spray. The experiment was performed in the freezer in completely dark conditions and at the same relative humidity (35±5%) for all treatments. Temperature changes in the freezer were regularly monitored when the samples were in the freezer. For all the freezing treatments, herbicides were applied at the same time. Plants were transferred to natural conditions after three nights of freezing treatment at 5:30 A.M. Also, plants related to freezing after spray were transferred to the freezer at 6:30 P.M. On the same day, plants that were not exposed to freezing were sprayed from 7:30 A.M. to 10.30 A.M. The herbicides were applied by a researcher-made moving boom sprayer equipped with a TeeJet 8002 flat fan nozzle (spray output volume of 200 L ha^{-1} at a pressure of 260 kPa). Plants were returned and maintained outdoor under natural conditions of winter after the treatment.

Measurement of traits

Four weeks after herbicide treatments, survival percentage (Eq. 1) and shoot dry weight were measured by placing the samples in an oven at 75℃ for 48 h. Plants that produced new leaves were considered alive and those that experienced severe chlorosis and stunted growth were recorded as dead [22].

$$Survival\ \% = \frac{N1}{N0} \times 100 \qquad \text{(Eq. 1)}$$

Where, N_1 and N_0 are the numbers of alive plants four weeks after herbicide treatment and the number of alive plants before herbicide treatment, respectively.

Dose–response experiments

The response of weed traits to the doses of herbicides tested was analyzed by nonlinear regression. For this purpose, the data were fitted to a three-parameter log-logistic equation (Eq. 2) [23].

$$Y = \frac{d}{1+e^{b(\log x - \log e)}} \qquad \text{(Eq. 2)}$$

Where, Y is the response rate (survival % or dry weight) at dose x, d is the upper limit for Y, b is the slope of the curve around e, and e is the effective dose (g ai or ae ha^{-1}). Here, e is replaceable with LD_{50} (dose required to control 50% of individuals in the population) or GR_{50} (dose causing 50% dry weight reduction of plants).

Relative potency

Relative potency was used to determine herbicide efficacy under freezing stress and no freezing stress (Eq. 3) [24].

$$Relative\ potency = \frac{ED_{50A}}{ED_{50B}} \qquad \text{(Eq. 3)}$$

Where, ED_{50A} is the dose required to reduce survival by 50% in NF, and ED_{50B} is the dose needed to reduce survival by 50% in FBS or FAS. Relative potency = 1 indicates that freezing treatment (FBS and FAS) did not affect herbicide efficacy. If relative potency > 1, it indicates that the herbicides are more efficient under freezing stress. Relative potency < 1 indicates the decreased effectiveness of the herbicides under freezing stress with respect to optimum conditions.

Selectivity index

The ratio between doses that caused 10% damage to crops and 90% damage to weeds was considered a selectivity index [24] and calculated using Eq. 4.

$$Selectivity\ index\ (10{,}90) = \frac{ED_{10\ (crop)}}{ED_{90\ (weed)}} \qquad \text{(Eq. 4)}$$

Where, ED_{90} is 90% of the herbicide effect on weed survival (herbicide dose to kill 90% of the population), and ED_{10} is 10% of the herbicide impact on crop survival (a 10% effect on the crop). The higher the SI, the more selective the herbicide in the crop [24].

Statistical analysis

The experiments were conducted using a completely randomized design in factorial arrangement with three replications. Afterward, differences between treatments were compared with the standard error of mean (SEM) values. For data analysis, analysis of variance (ANOVA) was performed using the SAS software (v. 9.4, SAS Institute Inc, Cary, NC, USA). Data for both runs were pooled as no significant differences were observed between runs ($P > 0.05$). Homogeneity of variance was confirmed using Levene's test. Analysis of biometric data was performed by the R program (v. 4.0.4) [25]. Dose-response curves were also fitted with the asymmetrical sigmoid form. LD_{50} and GR_{50} were separated using 95% confidence intervals (CI).

Results

Clodinafop-propargyl

Survival percentage and LD_{50}

The dose-response experiments were similar for NF and FAS treatments at the recommended dose (Figure 4A). Thus, in both conditions, winter wild oat was completely controlled with the recommended dose (64 g ai ha^{-1}). However, the survival of seedlings in the FBS treatment was 7%, and to achieve complete control, 2-fold the recommended dose (128 g ha^{-1}) was needed. The LD_{50} in NF, FBS, and FAS was 17.9, 18.1, and 23.2 g ha^{-1}, respectively (Table 1).

Fig. 4 Survival (A) and dry weight (B) of winter wild oat treated with various doses of clodinafop-propargyl under different freezing treatments. Abbreviation: NF (no freezing); FBS (freezing before spray): FAS (freezing after spray). The recommended dose of clodinafop-propargyl in Iran is 64 g ha^{-1}.

Table 1 The dose of clodinafop-propargyl and 2,4-D plus MCPA required for 50% mortality (LD_{50}) and the dose that caused 50% inhibition of growth noted by biomass (GR_{50}) of winter wild oat and turnipweed under different freezing treatments.

Herbicide	Freezing Treatment	b±SE	d±SE	LD_{50} ±SE	95% CI	R^2
			%	g ai or ae ha^{-1}		
Clodinafop-propargyl	NF	$2.0 \pm 0.19^{**}$	$100.3 \pm 2.8^{**}$	$17.9 \pm 1.2^{**b}$	15.6-20.2	0.99
	FBS	$1.9 \pm 0.17^{**}$	$101.0 \pm 2.9^{**}$	$18.1 \pm 1.2^{**b}$	15.7-20.5	0.99
	FAS	$1.9 \pm 0.19^{**}$	$98.7 \pm 2.9^{**}$	$23.2 \pm 1.7^{**a}$	19.8-26.6	0.96
2,4-D plus MCPA	NF	$1.9 \pm 0.20^{**}$	$102.2 \pm 3.2^{**}$	$292.8 \pm 21.9^{**c}$	249.4-336.3	0.98
	FBS	$1.5 \pm 0.15^{**}$	$102.4 \pm 3.2^{**}$	$472.8 \pm 42.4^{**a}$	388.8-556.8	0.98
	FAS	$1.5 \pm 0.14^{**}$	$102.8 \pm 3.5^{**}$	$318.8 \pm 28.9^{**b}$	261.6-376.1	0.98
		b±SE	d±SE	GR_{50} ±SE	95% CI	
			g pot^{-1}	g ai or ae ha^{-1}		
Clodinafop-propargyl	NF	$2.1 \pm 0.15^{**}$	$0.70 \pm 0.02^{**}$	$10.6 \pm 0.52^{**c}$	9.6-11.6	0.99
	FBS	$2.0 \pm 0.12^{**}$	$0.80 \pm 0.02^{**}$	$11.6 \pm 0.51^{**b}$	10.6-12.6	0.99
	FAS	$1.7 \pm 0.12^{**}$	$0.67 \pm 0.02^{**}$	$12.8 \pm 0.74^{**a}$	11.3-14.3	0.98
2,4-D plus MCPA	NF	$2.2 \pm 0.23^{**}$	$0.46 \pm 0.01^{**}$	$176.2 \pm 11.3^{**c}$	153.9-198.6	0.99
	FBS	$1.7 \pm 0.16^{**}$	$0.48 \pm 0.01^{**}$	$298.6 \pm 21.5^{**a}$	256.0-341.3	0.99
	FAS	$1.6 \pm 0.15^{**}$	$0.47 \pm 0.01^{**}$	$207.8 \pm 15.9^{**b}$	176.2-239.4	0.99

Abbreviation: NF (no freezing); FBS (freezing before spray); FAS (freezing after spray); SE: Standard Error. b is the slope of the curve around e (LD_{50} and GR_{50}); d is the upper limit for Y (survival % or dry weight); **: significant at $p \leq 0.01$. LD_{50} and GR_{50} were separated using their 95% confidence intervals (CI). The data shows the parameter's mean ± SE. The data (LD_{50} and GR_{50}) with the different letters indicate significant differences according to LSD at $P \leq 0.05$. n = 6

Dry weight and GR_{50}

The dry weight of winter wild oat in NF and FAS with the recommended dose (64 g ha^{-1}) and FBS with twice the recommended dose (128 g ha^{-1}) reached 0 g (Fig. 4B). The GR_{50} of plants not exposed to freezing stress was 10.6 g ha^{-1}. The GR_{50} was 11.6 and 12.8 g ha^{-1} in FBS and FAS, respectively (Table 1).

2,4-D plus MCPA

Survival percentage and LD_{50}

The dose-response experiments of 2,4-D plus MCPA (similar to clodinafop-propargyl) were the same in NF and FAS treatments (Fig. 5A). In both conditions, turnipweed was completely controlled with twice the recommended dose (2025 g ae ha^{-1}). However, in the FBS treatment, the survival of turnipweed was 7%. At the recommended dose (1012.5 g ha^{-1}), the survival of turnipweed in NF, FBS, and FAS was 3%, 33%, and 20%, respectively. The LD_{50} in NF, FBS, and FAS was 293, 473, and 319 g ha^{-1}, respectively (Table 1).

Fig. 5 Survival (A) and dry weight (B) of turnipweed treated with various doses of 2,4-D plus MCPA under different freezing treatments. Abbreviation: NF (no freezing); FBS (freezing before spray): FAS (freezing after spray). The recommended dose of 2,4-D+MCPA in Iran is 1012.5 g ha^{-1}.

Dry weight and GR_{50}

The dry weight of turnipweed in NF and FAS with twice the recommended dose (2025 g ha^{-1}) of 2,4-D plus MCPA was reduced to 0 (Fig. 5 B). However, in the FBS treatment, the dry weight of turnipweed with twice the recommended dose was 0.012 g per pot. At the recommended dose, the dry weight of turnipweed in FBS was 97% and 76% more than NF and FAS, respectively. The GR_{50} of NF, FBS, and FAS were 176, 299, and 208 g ha^{-1}, respectively (Table 1).

Relative potency

The relative potency of clodinafop-propargyl and 2,4-D plus MCPA was significantly reduced in FBS and FAS compared to NF (Fig. 6). Thus, the relative potency in FBS and FAS of clodinafop-propargyl was 0.99 and 0.77, respectively, and at 2,4-D plus MCPA was 0.62 and 0.92, respectively.

Fig. 6 Relative potency of clodinafop-propargyl and 2,4-D plus MCPA under different freezing treatments. Abbreviation: FBS (freezing before spray) and FAS (freezing after spray). The error bars indicate standard error (SE). The dashed line represents the relative potency = 1.0.

Selectivity index

As shown in Table 2, selectivity index values for clodinafop-propargyl in NF, FBS, and FAS treatments were 2.4, 0.91, and 0.78, respectively, and, for 2,4-D plus MCPA, they were 2.6, 0.12, and 0.88, respectively. For clodinafop-propargyl, the LD_{10} of wheat in NF was 127.9 g ha^{-1}. However, LD_{10} of wheat in the FBS and FAS was 53.5 and 57.1 g ha^{-1}, respectively (Table 2). For 2,4-D plus MCPA, the LD_{10} of wheat in NF, FBS, and FAS was 2380, 264, and 1236 g ha^{-1}, respectively.

Table 2 Sensitivity of plant species of wheat, winter wild oat, and turnipweed to clodinafop-propargyl and 2,4-D plus MCPA under different freezing treatments.

Freezing Treatment	Plant species	Clodinafop-propargyl		2,4-D plus MCPA	
		LD_{10} (wheat) and LD_{90} (winter wild oat) (g ai ha^{-1}) ± SE	SI	LD_{10} (wheat) and LD_{90} (turnipweed) (g ae ha^{-1}) ± SE	SI
NF	Wheat	127.9 ± 8.8	-	2380 ± 455	-
	Weed	53.5 ± 5.4	2.4	908 ± 108	2.6
FBS	Wheat	53.5 ± 11.0	-	264 ± 61.0	-
	Weed	59.0 ± 6.4	0.91	2144 ± 327	0.12
FAS	Wheat	57.1 ± 8.7	-	1236 ± 163	-
	Weed	73.6 ± 7.2	0.78	1400 ± 199	0.88

Abbreviation: NF; No freezing, FBS: Freezing before spray, FAS: Freezing after spray SI: Selectivity index. The selectivity index (SI) was calculated as follows: *Selectivity index (10, 90):* $\frac{ED10\ (crop)}{ED90\ (weed)}$

Discussion

The required dose of clodinafop-propargyl for complete control of winter wild oat seedlings in FBS treatment was twice that of NF and FAS treatments (Fig. 4, A). The trend of the reducing survival % in seedlings related to the FBS treatment and NF was similar. The LD_{50} for clodinafop-propargyl in freezing treatments shows that exposure of winter wild oat seedlings to freezing treatment for three nights before and after spray reduces the efficacy of this herbicide compared to NF. The LD_{50} was 22% and 23% higher in the FAS compared to the FBS and NF, respectively (Table 1). Freezing treatment for three nights before and after spraying of clethodim herbicide (the mode of action similar to clodinafop-propargyl) led to an increase in LD_{50} [19]. It decreased herbicide efficacy in rigid ryegrass (*Lolium rigidum* Gaud.) populations compared to NF.

The dose of clodinafop-propargyl required to reduce winter wild oat biomass to zero in the FBS treatment was twice that of the NF and FAS treatments (Fig. 4, B). The GR_{50} is more pronounced in FBS and FAS compared to NF, indicating a decrease in the efficacy of clodinafop-propargyl. The GR_{50} was 17% and 9% higher in the FAS treatment compared to NF and FBS, respectively. Hence, according to the results of GR_{50}, three nights of freezing stress after herbicide application reduced the performance of clodinafop-propargyl more than other treatments (Table 1). It has been reported that wild oat plants (*Avena fatua*) that grew at low temperatures were less sensitive to difenzoquat than plants that grew at high temperatures [26].

Our results clearly show that the occurrence of freezing stress around spray reduces the efficacy of clodinafop-propargyl, especially in the treatment of FAS. Herbicide translocation in the phloem in freezing conditions could decline due to damage to the phloem cells, which may lead to a decrease in the herbicide efficacy. Decreased efficacy of sethoxydim at low

temperatures due to reduced herbicide translocation for Quackgrass [*Elymus repens* (L.) Gould] control was reported by Coupland [27]. In general, to maintain the herbicide efficacy under cold conditions, using clodinafop-propargyl after the freezing stress can be recommended in areas with a similar climate.

Although the three-night treatment of FBS and FAS resulted in a reduction in turnipweed control by 2,4-D plus MCPA compared to NF, this reduction was different in the two freezing treatments (Fig. 5, A). At the recommended dose (1013 g ha^{-1}) in NF, almost all turnipweed plants were controlled, while the survival percentage of turnipweed in FBS and FAS treatments were 33% and 20%, respectively. Low temperatures around the time of herbicide application reduced the effectiveness of systemic herbicides [28]. Based on LD_{50} values, the efficacy of 2,4-D plus MCPA under FBS and FAS was 1.6 and 1.1 times lower compared to NF, respectively (Table 1).

FAS is possible to damage the phloem cells, which reduces the translocation of the herbicide. The efficacy of 2,4-D plus MCPA in the treatment of FBS was reduced. The decrease in the effect of 2,4-D plus MCPA in the treatment of FBS is probably due to the reduction of absorption and translocation of this herbicide in turnipweed seedlings. In this regard, an increase in efficacy of 2,4-D and glyphosate at high temperatures compared to low temperatures in common ragweed (*Ambrosia artemisiifolia* L.) and giant ragweed (*Ambrosia trifida* L.) due to the increased absorption and/or translocation at high temperatures around herbicide application has been reported by Ganie et al. [29]. It has been also reported that low temperatures reduced glufosinate activity and translocation in wild radish (*Raphanus raphanistrum* L.) [30].

Turnipweed's dry weight at the recommended dose of 2,4-D plus MCPA (1012.5 g ha^{-1}) in FBS and FAS treatments was 35.5 and 8.5 times higher than the NF, respectively (Fig. 5, B).

Also, GR_{50} in FBS and FAS was 41% and 15.2% higher compared to NF, respectively (Table 1). Although a decrease in herbicide efficacy is evident in both FBS and FAS treatments, high GR_{50} in FBS shows an adverse effect of three nights of freezing before the application of 2,4-D plus MCPA. In other words, the efficacy of this herbicide reacts more to FBS than to FAS. In a similar study, the GR_{50} for 2,4-D increased at low temperature in common ragweed compared to high temperature [29].

The freezing stress around 2,4-D plus MCPA application reduced the efficacy of this herbicide in FBS and FAS treatments compared to NF. The decrease in the efficacy of this herbicide in the treatment of FBS is probably due to the reduction of absorption and translocation of the herbicide in the phloem during freezing. Absorption and translocation of 2,4-D plus MCPA might be reduced at low temperatures due to the effect on herbicide penetration facilitated by physicochemical factors, including the decreased rate of diffusion, increased viscosity of the cuticle, and physiological factors comprising decreases in photosynthesis, phloem translocation, and protoplasmic streaming [29]. In Iranian wheat fields, using the 2,4-D plus MCPA before the freezing stress can be recommended to maintain the herbicide efficacy under cold conditions.

When relative potency > 1, the herbicide efficacy is higher than optimal non-freezing conditions, and when relative potency < 1, the herbicide efficacy is lower than usual [24]. The relative potency values of clodinafop-propargyl and 2,4-D plus MCPA were lower than one under both FBS and FAS treatments in the present experiment (Fig. 6). In other words, freezing stress reduced the efficacy of these herbicides. Based on the results of relative potency, the reduction of clodinafop-propargyl efficacy in the FAS treatment (33%) was more significant than

in the FBS treatment (1%). Conversely, for 2,4-D plus MCPA, the reduction in efficacy was more significant in the FBS treatment (38%) compared to the FAS treatment (8%).

The selectivity index for clodinafop-propargyl and 2,4-D plus MCPA was > 1 in NF, indicating that both herbicides acted selectively in wheat (Table 2). However, applying three nights of FBS and FAS of both herbicides reduced the selectivity index to less than one. In other words, freezing stress reduced the selectivity index of both herbicides, which led to phytotoxicity in wheat. For clodinafop-propargyl, LD_{10} of wheat in NF was 58% and 55% higher than FBS and FAS, respectively. The LD_{10} values of 2,4-D plus MCPA in NF were 89% and 48% higher compared to FBS and FAS, respectively. In other words, the dose required to kill 10% of wheat in both herbicides under NF was higher than FBS and FAS. Therefore, FBS and FAS increased the sensitivity of wheat to herbicide application. Freezing stress may reduce the rate of herbicide metabolism in the crop and ultimately increase its susceptibility [17].

Conclusions

The relative potency results for both herbicides were used to confirm the results of LD_{50} and GR_{50}. Thus, LD_{50} and GR_{50} were higher for clodinafop-propargyl in the FAS treatment than in NF and FBS treatments. The LD_{50} and GR_{50} were higher for the 2,4-D plus MCPA in the FBS treatment than in the NF and FAS treatments. In other words, clodinafop-propargyl was more effective than other conditions in the treatment of three nights of FAS and 2,4-D plus MCPA in the treatment of three nights of FBS. The selectivity index for clodinafop-propargyl was lower in the FAS treatment (0.78) and for 2,4-D plus MCPA was lower in the FBS treatment (0.12). The efficacy of clodinafop-propargyl in the FAS treatment and the efficacy of 2,4-D plus MCPA in the FBS treatment were reduced. The values of the selectivity index of herbicides in the

conditions of freezing stress showed that this index could be well used to determine the selective performance of these herbicides. Overall, according to the results of this experiment, it can be stated that FAS with ACCase inhibitor herbicides, such as clodinafop-propargyl, may reduce their effectiveness in controlling winter wild oat. FBS with auxin type herbicides, such as 2,4-D plus MCPA, reduce their efficacy in controlling turnipweed. FBS for clodinafop-propargyl and FAS for 2,4-D plus MCPA had a slight adverse effect on the herbicide efficacy and selectivity index. Finally, research on the use of adjuvants, alternate herbicides, and various herbicide formulations to control grass and broadleaf weeds in Iranian wheat production is suggested to prevent the adverse effects of freezing stress on herbicides.

Conflicts of interest

The authors declare that they have no conflict of interest.

Acknowledgment

We are grateful to Ferdowsi University of Mashhad, Iran, for providing financial support for present research (Project No.3.49759). No additional external funding was received for this study.

References

1. Hatami ZM, Gherekhloo J, Rojano-Delgado AM, Osuna MD, Alcántara R, Fernández P, et al. Multiple mechanisms increase levels of resistance in *Rapistrum rugosum* to ALS herbicides. Front Plant Sci. 2016 Feb 22;7:169. https://doi.org/10.3389/fpls.2016.00169
2. Nosratti I, Sabeti P, Chaghamirzaee G, Heidari H. Weed problems, challenges, and opportunities in Iran. Crop Prot. 2020 Aug 1;134:104371. https://doi.org/10.1016/j.cropro.2017.10.007
3. Hasanfard A, Rastgoo M, Darbandi EI, Nezami A, Chauhan BS. Regeneration capacity after exposure to freezing in wild oat (*Avena ludoviciana* Durieu.) and turnipweed (*Rapistrum rugosum* (L.) All.) in comparison with winter wheat. Environ. Exp. Bot. 2021 Jan 1;181:104271. https://doi.org/10.1016/j.envexpbot.2020.104271
4. Sierra-Almeida A, Cavieres LA, Bravo LA. Warmer temperatures affect the in situ freezing resistance of the Antarctic vascular plants. Front. Plant Sci. 2018 Oct 8;9:1456. https://doi.org/10.3389/fpls.2018.01456

5. Willick IR, Tanino KK, Gusta LV. The impact of global climate change on the freezing tolerance of winter cereals in Western Canada. J. Agron. Crop Sci. 2021 Feb;207(1):88-99. https://doi.org/10.1111/jac.12447
6. Vyse K, Pagter M, Zuther E, Hincha DK. Deacclimation after cold acclimation—a crucial, but widely neglected part of plant winter survival. J. Exp. Bot. 2019 Sep 15;70(18):4595-604. https://doi.org/10.1093/jxb/erz229
7. Cici SZ, Van Acker RC. Relative freezing tolerance of facultative winter annual weeds. Can. J. Plant Sci. 2011;91(4):759-63. https://doi.org/10.4141/cjps2010-001
8. Baghestani MA, Zand E, Soufizadeh S, Beheshtian M, Haghighi A, Barjasteh A, Birgani DG, Deihimfard R. Study on the efficacy of weed control in wheat (*Triticum aestivum* L.) with tank mixtures of grass herbicides with broadleaved herbicides. Crop Prot. 2008 Jan 1;27(1):104-11. https://doi.org/10.1016/j.cropro.2007.04.013
9. Enghiad A, Ufer D, Countryman AM, Thilmany DD. An overview of global wheat market fundamentals in an era of climate concerns. Int J Agron. 2017 Jul 2;2017. https://doi.org/10.1155/2017/3931897
10. Bajwa AA, Akhter MJ, Iqbal N, Peerzada AM, Hanif Z, Manalil S, Hashim S, Ali HH, Kebaso L, Frimpong D, Namubiru H. Biology and management of Avena fatua and Avena ludoviciana: two noxious weed species of agro-ecosystems. Environ Sci Pollut Res. 2017 Aug;24(24):19465-79. https://doi.org/10.1007/s11356-017-9810-y
11. Peterson MA, McMaster SA, Riechers DE, Skelton J, Stahlman PW. 2, 4-D past, present, and future: a review. Weed Technol. 2016 Jun;30(2):303-45. https://doi.org/10.1614/WT-D-15-00131.1
12. Collings LV, Blair AM, Gay AP, Dyer CJ, Mackay N. The effect of weather factors on the performance of herbicides to control *Alopecurus myosuroides* in winter wheat. Weed Res. 2003 Apr;43(2):146-53. https://doi.org/10.1046/j.1365-3180.2003.00327.x
13. Rocha-Pereira MR, Klar AE, Martins D, Ferreira de Souza GS, Villalba J. Efecto del estrés hídrico sobre la eficacia de los herbicidas aplicados en *Urochloa decumbens*. Cienc. Investig Agrar. 2012 Apr;39(1):211-20. http://dx.doi.org/10.4067/S0718-16202012000100018
14. Johnson BC, Young BG. Influence of temperature and relative humidity on the foliar activity of mesotrione. Weed Sci. 2002 Apr;50(2):157-61. https://doi.org/10.1614/0043-1745(2002)050[0157:IOTARH]2.0.CO;2.
15. Chahal PS, Aulakh JS, Rosenbaum K, Jhala AJ. Growth stage affects dose response of selected glyphosate-resistant weeds to premix of 2,4-D choline and glyphosate (Enlist Duo (TM) herbicide*). J. Agric. Sci. 2015 Nov 1;7(11):1. https://doi.org/10.5539/jas.v7n11p1
16. Waltz AL, Martin AR, Roeth FW, Lindquist JL. Glyphosate efficacy on velvetleaf varies with application time of day. Weed Technol. 2004 Dec;18(4):931-9. https://doi.org/10.1614/WT-03-123R3
17. Godar AS, Varanasi VK, Nakka S, Prasad PV, Thompson CR, Mithila J. Physiological and molecular mechanisms of differential sensitivity of Palmer amaranth (*Amaranthus palmeri*) to mesotrione at varying growth temperatures. Plos One. 2015 May 19;10(5):e0126731. https://doi.org/10.1371/journal.pone.0126731
18. Pline WA, Wu J, Hatzios KK. Effects of temperature and chemical additives on the response of transgenic herbicide-resistant soybeans to glufosinate and glyphosate

applications. Pestic. Biochem. Phys. 1999 Oct 1;65(2):119-31. https://doi.org/10.1006/pest.1999.2437
19. Saini RK, Malone J, Preston C, Gill GS. Frost reduces clethodim efficacy in clethodim-resistant rigid ryegrass (*Lolium rigidum*) populations. Weed Sci. 2016 Jun;64(2):207-15. https://doi.org/10.1614/WS-D-15-00140.1
20. Moseley C, Hatzios KK, Hagood ES. Uptake, translocation, and metabolism of chlorimuron in soybean (*Glycine max*) and morningglory (*Ipomoea* spp.). Weed Tech. 1993 Jun;7(2):343-8. https://doi.org/10.1017/S0890037X00027706
21. Negrisoli E, Velini ED, Tofoli GR, Cavenaghi AL, Martins D, Morelli JL, Costa AG. Selectivity of pre-emergence herbicides to sugarcane treated with nematicides. Planta Daninha. 2004 Dec;22(4):567-75.
22. Powles SB, Lorraine-Colwill DF, Dellow JJ, Preston C. Evolved resistance to glyphosate in rigid ryegrass (*Lolium rigidum*) in Australia. Weed Sci. 1998 Oct;46(5):604-7.
23. Ritz C, Baty F, Streibig JC, Gerhard D. Dose-response analysis using R. Plos One. 2015 Dec 30;10(12):e0146021. https://doi.org/10.1371/journal.pone.0146021
24. Tind T, Mathiesen TJ, Jensen JE, Ritz C, Streibig JC. Using a selectivity index to evaluate logarithmic spraying in grass seed crops. Pest Manag Sci: formerly Pesticide Science. 2009 Nov;65(11):1257-62. https://doi.org/10.1002/ps.1819
25. Team RC. R Foundation for Statistical Computing, Vienna, available at: www. R-project. org. 2018.
26. Kowalczyk B, Caseley JC, McCready CC. The effect of pre-treatment temperature on some physiological responses of *Avena fatua* to difenzoquat and benzoylpropethyl. Asp Appl Biol. 1983;4:235-44.
27. Coupland D. Influence of environmental factors on the performance of sethoxydim against *Elymus repens* (L.). Weed Res. 1987 Oct;27(5):329-36. https://doi.org/10.1111/j.1365-3180.1987.tb00770.x
28. Derr J, Serensits T. Impact of application temperature on broadleaf herbicide efficacy. J Environ Hortic. 2016 Dec;34(4):123-30. https://doi.org/10.24266/0738-2898-34.4.123
29. Ganie ZA, Jugulam M, Jhala AJ. Temperature influences efficacy, absorption, and translocation of 2,4-D or glyphosate in glyphosate-resistant and glyphosate-susceptible common ragweed (*Ambrosia artemisiifolia*) and giant ragweed (*Ambrosia trifida*). Weed Sci. 2017 Sep;65(5):588-602. https://doi.org/10.1017/wsc.2017.32
30. Kumaratilake AR, Preston C. Low temperature reduces glufosinate activity and translocation in wild radish (*Raphanus raphanistrum*). Weed Sci. 2005 Feb;53(1):10-6. https://doi.org/10.1614/WS-03-140R

Manuscript Number: **PONE-D-21-33766**

Manuscript title: **Freezing stress affects the efficacy of clodinafop-propargyl and 2,4-D plus MCPA on wild oat and turnipweed in wheat**

Dear Editor

We sincerely appreciate all valuable comments and suggestions, which helped us to improve the quality of the article. We hope that you find our responses satisfactory and that the manuscript is now acceptable.

Sincerely,

Mehdi Rastgoo
Associate professor,
Department of Agrotechnology, Faculty of Agriculture, Ferdowsi University of Mashhad, Iran
Corresponding author

General comment

Your reasoning why you have chosen mix of two auxinic ingredients and discussion needs to be improved although it has been successful already. You need to use latin names of weeds in title and abstract as well. You need further information to be given about herbicides such as safener in clodinafop if there is, acid equivalent rates of auxicinic herbicides, separated amounts of 2,4-D and MCPA. I am not sure if you need both ED50 and GR50. By the way, no GR50 calculation in your materials and methods.

- The mix of these two auxinic ingredients is one of the most important compounds in the control of broadleaf weeds in wheat fields globally, whose efficiency is generally affected by abiotic stresses. We feel that we have answered this question well in the text of the paper.
- Latin names of weeds were added as Titles and Abstracts.
- The requested revision was applied to the 2,4-D (acid equvalent rates of auxicinic herbicides).
- In this experiment, we calculated two important survival and dry weight parameters. We calculated both parameters because sometimes the herbicide does not lead to complete control of the weed plant but reduces the growth of the plant and its dry weight. Therefore, the optimal effect of the herbicide may be hidden in reducing the dry weight of the herbicide.
- In the Dose-response experiments section, we have mentioned how to calculate both parameters (LD50 and GR50).

Response to the comments of the reviewer #2

We would like to thank the reviewer for the careful and thorough reading of this manuscript and the thoughtful comments and constructive suggestions, which help improve the manuscript quality. As indicated below, we have checked all the general and specific comments provided by the referees and have made necessary changes according to their indications, which are highlighted in yellow.

Reviewer #2: Reviewer comments PONE-D-21-33766R1

The revised manuscript entitled „Freezing stress affects the efficacy of clodinafop-propargyl and 2,4-D plus MCPA on wild oat and turnipweed in wheat" was significantly improved by the authors according to my previous comments.

I think that the revised manuscript is suitable for publication in PLoS One.

I have only a few formal comments on the revised manuscript which are provided below:

Abstract, line 23: The abbreviations „2,4-D" and „MCPA" have to be explained when used for the first time.

- We respect the reviewer's opinion, but this is the common name of the herbicide and should be mentioned without further explanation. It is not common to write its full name in a scientific article. However their full names are as follows.
  2,4-Dichlorophenoxyacetic acid *and* 2-methyl-4-chlorophenoxyacetic acid

Abstract, line 29 and the further text including the figures: The „ai" used in the „g ai ha-1" has to be explained. What is the difference between „g ai ha-1" and „g ha-1"??

- Both units are correct. Usually, g ai ha^{-1} is first mentioned in articles and then g ha^{-1}.

Line 134: Add a comma following the words „Each morning at 5:30 A.M., …"

- The requested revision was applied to the text.

Line 337: Use the term „optimal non-freezing conditions" instead of „normal conditions."

- The requested revision was applied to the text.

Response to the comments of the reviewer #3

In general, the paper is interesting and the topic nice. However, several serious issues ought to be addressed. First of all, I absolutely disagree with statements of the authors (in the abstract and the text) that the occurrence of freezing stress around herbicides application is one of the most important factors influencing their performance. Many more factors (rate, growth stage, adjuvant, weed species, water volume, pressure etc) play a significant role on that. Moreover, survival 7% means efficacy of 93%, farmers do not need to double the rate to achieve a complete control (usually farmers are satisfied with an efficacy of 90-95%). In wild oat authors say that under no freezing (NF) and freezing after spray (FAS) conditions, winter wild oat was completely controlled with the recommended dose of clodinafop-propargyl, while at FBS control was 93%. Why the ranking of LD50 and GR50 was different (meaning the FBS between the two others)? There is an important confusion. Furthermore, I would encourage authors a) to justify their choice to evaluate an ACCase inhibitor alone and a mixture of auxinic herbicides (and not an individual)? and b) substantially revise and improve the discussion section in order to talk more about the potential mechanism behind that reduced efficacy and the potential generalization of their results (since in many cases farmers chose to spray under low temperatures since the notice an increased susceptibility of several weeds to glyphosate and other herbicides).

- Many factors affect the effectiveness of herbicides. We also mentioned throughout the text that frost stress is "one of the most important factors (not the only one factor)" influencing the performance of herbicides.
- We completely agree with the esteemed referee regarding the ideal amount of weed control at the recommended dose (90 to 95%). But we did not know the result before the test, and naturally, we had to try twice the recommended dose. However, it is not recommended with 93% control of double dose use, and we discussed this issue in the article.
- In non-stress freezing and freezing after spraying with the recommended dose of clodinafop-propargil, the survival rate was zero (Figure 4), but the results were different for LD_{50} and GR_{50} (Table 1). Survival % and survival and dry weight 50% (LD_{50} and GR_{50}) can vary according to their definition (more details in the results and discussion section). Survival % with LD_{50} and GR_{50} can differ depending on their definition.
- More information in the results and discussion section.

We look forward to hearing from you regarding our submission. We would be glad to respond to any further questions and comments that you may have.

Manuscript Number: **PONE-D-21-33766**

Manuscript title: **Freezing stress affects the efficacy of clodinafop-propargyl and 2,4-D plus MCPA on wild oat (*Avena ludoviciana* Durieu) and turnipweed [*Rapistrum rugosum* (L.) All.] in wheat (*Triticum aestivum* L.)**

Dear Dr Uludag

Thank you very much for organizing the review of our manuscript. We have addressed all the comments/issues raised by the reviewers and have made necessary changes according to their indications, which are highlighted in purple (new revision).

The opinions of reviewer 4 are extraordinary, and we do not agree with some of them. Our manuscript has been read several times by a Native English speaker and recognized weed science expert. He emphasized that there was no problem with the language of this manuscript. The reviewer should not criticize the language of the article with a general comment. If the language has a problem, it should mention exactly which sentence? Which section?

Our MS has been read several times by one of the top experts and editors in weed science (professor Chauhan). Isn't this the reviewer's expression strange?!
Reviewer 4: Note that being a native English speaker is not synonymous with being a good writer.

His opinion about statistical analysis shocked us. We used the most powerful method and software for analysis. Regarding the data pooling, the variance of the data of the two experiments is equal (based on Bartlett's or Levene's test), and we can pool the data. You will find a simple search for high-quality sources. Data for both runs were pooled as no significant differences were observed between runs.

- *Arsham, H., & Lovric, M. (2011). Bartlett's Test. International encyclopedia of statistical science, 1, 87-88.*
- *Niyonzima, N., Bakke, S. S., Gregersen, I., Holm, S., Sandanger, Ø., Orrem, H. L., ... & Espevik, T. (2020). Cholesterol crystals use complement to increase NLRP3 signaling pathways in coronary and carotid atherosclerosis. EBioMedicine, 60, 102985.*
-*Mahajan, G., Rachaputi, R. C. N., & Chauhan, B. S. (2020). Horse purslane (Trianthema portulacastrum) control in pigeonpea with PRE and POST herbicides. Weed Technology, 34(5), 764-769.*
-*Mobli, A., Manalil, S., Khan, A. M., Jha, P., & Chauhan, B. S. (2020). Effect of emergence time on growth and fecundity of Rapistrum rugosum and Brassica tournefortii in the northern region of Australia. Scientific Reports, 10(1), 1-10.*
- *Sritan, K., & Phuenaree, B. (2021). A Comparison of Efficiency for Homogeneity of Variance Tests under Log-normal Distribution. Asian Journal of Applied Sciences, 9(4).*

We should not rely on a particular article from 19 years ago. We have explained this in a clear and complete analysis section. Note that none of the reviewers objected to our analysis except reviewer 4.

In all high-quality research, the use of standard error mean (SEM) is expected.
Saini, R. K., Malone, J., Preston, C., & Gill, G. S. (2016). Frost reduces clethodim efficacy in clethodim-resistant rigid ryegrass (Lolium rigidum) populations. Weed Science, 64(2), 207-215.
The SEM is used to determine the differences between more than one sample of data. It helps us estimate how well your sample data represents the whole population by measuring the accuracy with which the sample data represents a population using standard deviation. However, we added items like mean comparison (LSD test) to the manuscript (Table 1).
Apart from the above, we feel that the reviewer did not read the paper well. For example, we had three repetitions in each run, and each repetition had eight plants. The reviewer incorrectly stated eight plants in each repetition. In addition, despite our complete explanations and figures, the reviewer did not notice that the plants were exposed to freezing stress for three nights, which reached the minimum temperature (-4°C).

Reviewer 4 has requested the details of herbicides. Meanwhile, we have already provided all the valuable information in the paper (purple highlights in the Introduction and Experimental factors).

Sincerely,
Corresponding author

p.s. we have addressed the rest, and we expect to hear your final opinion after about a year and apply the comments of five reviewers. Naturally, you send each manuscript to each reviewer, who will have their unique comments and revisions. How long should this cycle continue? We emphasize that we applied all the comments of previous reviewers (as declared by reviewer 5). We hope that you find our responses satisfactory and that the manuscript is now acceptable.

Review Comments to the Author

Please use the space provided to explain your answers to the questions above. You may also include additional comments for the author, including concerns about dual publication, research ethics, or publication ethics. (Please upload your review as an attachment if it exceeds 20,000 characters)

Reviewer #4: The manuscript investigated the response of two problematic weed species to herbicides under freezing conditions. I have realized that some other colleagues had reviewed the manuscript. I also realized that one of the reviewers raised a concern regarding the language of the manuscript, but the authors rebutted this comment and claimed that their manuscript had been edited by “a Native English-speaker colleague”. Please note that being a native English speaker is not synonymous with being a good writer. After reading the manuscript, I also feel the language is not comprehensive in many places and does not meet the minimum standard required by international journals. Hence, I recommend editing help from a language service.

We expressed our opinion about this to the academic editor.

Specific comments

Lines 24 and 68- Please spell out these abbreviated herbicide names. The idea is that the manuscript should be comprehensive to any readers, not only to those who share the same area of expertise as the authors.

The requested information was added to the text (2,4-Dichlorophenoxyacetic acid and 2-methyl-4-chlorophenoxyacetic acid). The clodinafop-propargyl is the full name of this herbicide and not an abbreviation.

Line 100 “seeds of winter wild oat and turnipweed” How many plants did you sample? Did the samples size sufficiently represent the population?

Weed seeds are collected from at least 500 plants, and the sample size sufficiently represents the population.

Line 105 “The seeds were placed in” I assume this refers to weed seed germination. How about wheat?

The requested information was added to the text.

Line 109 “same time on October 21 (first run) and 24 (second run) in 2019”. I would not call this “a repeat in time”. We usually repeat experiments in time to cover sufficient spatial and temporal variation. Repeating an experiment 3 days after the other does not meet this criterion.

We did not claim that the experiment was repeated in time. The examination is designed based on our purposes.

Line 111 Nutrient deficiency can affect herbicide efficiency. The authors claimed they had not added any fertilizer to the potting media. This needs to be justified in the manuscript; otherwise, it is a major flaw.

We agree with the reviewer, but a large part of the soil used was farm soil, which had suitable physiochemical. Also, note that the soil conditions were the same in all experiments. Hence, the results are reported under similar conditions.

Line 112 Inconsistency. In Line 106-108, the authors claimed that they had pregerminated the seed of each weed species, and subsequently, they transplanted the seedlings. But here, they claimed they planted the seed directly. Please justify.

Both weed species were pregerminated. Then Germinated turnipweed seeds were planted in a seedling tray. Germinated wild oat seeds were planted directly in the soil. Fully and clearly explained (Please see M&M).

Line 113 Only eight plants per replicate? Would this number of plants sufficiently represent the population sample? Since the authors estimated the LD50, I suspect the low number of plants used in this experiment would sufficiently represent the variability among individuals of sampled populations? How many pregerminated seeds did you place in each pot for turnipweed?

8 plants×3Rep = (24 plants) × 2 Run= 48 plants
It makes sense for a controlled trial.
Eight turnipweed seedlings transferred to pots (added to the text).

Line 115 “irrigated according to daily requirements” How?

Irrigation was sufficient (based on the soil requirement of a pot). ~ 400 ml per pot. In this way, the plants were not subjected to drought stress.

Line 114 How did you grow wheat?

It is obvious. The wheat seed does not require to break dormancy. It does not need pre-germination either. So it was direct cultivation.

Lines 132-133 “The pots were kept at -4°C for 1 h and then removed from the freezer (Fig. 2).” I can`t follow this part. The plants were supposed to be kept in a freezer from 7pm to 5 am. But the authors remove the plants after 1 h exposure to -4 °C. Does this mean that the temperature reached to 4 °C at about 4 am?

We have provided clear explanations in the text. We have included figures 2 and 3 in the MS for clarification. The plants were exposed to the determined temperature gradient for three nights and reached the minimum temperature (-4°C).

Trend of temperature decrease in thermogradient freezer during the night from 7:00 P.M. to 5:00 A.M. (Please see Fig. 2)

Time	Temperature (℃)
7 P.M.	5
12 A.M.	Reach a temperature of 0
1 A.M.	kept at 0
2 A.M.	Reach a temperature of -2
3 A.M.	kept at -2
4 A.M.	Reach a temperature of -4
5 A.M.	kept at -4
5:30 A.M.	Removed from the freezer

Line 157 "herbicides" Please give more details than "herbicides". What was the formulation? Did you use any wetting agents? What was the trade name of each herbicide? What was the portion of each herbicide in the mixture of 2,4-D and MCPA?

For details on herbicides, refer to the Introduction and Experimental factors We would have mentioned it in the paper if we had used agents. So we did not use any agent. The trade name of each herbicide is added to the text (Experimental factors). . The manufacturing company combines this herbicide (360 g ae.L^{-1} for 2,4-D and 315 g ae.L^{-1} for MCPA). We did not mix it.

Line 174 "the curve around e, and e is the effective dose", The letter "e" is used in two different places in the equation. Which one represents the "effective dose"?

Both indicate the effective dose.

Line 195- 196 "Afterward, differences between treatments …" This is not really a solid approach to compare treatments statistically.

In all high-quality research, a standard error mean (SEM) is expected. We need SEM values for a dose-response graph, and this is necessary. However, we added items like mean comparison (LSD test) to the manuscript (Table 1).

Line 197-198 Pooling data from two experiments is a poor practice and results in missing many effects that are needed. The most appropriate statistical approach is to use a "mixed model analysis". Please see the manuscript below for more information.
Piepho, H. P., A. Bu¨chse, and K. Emrich, 2003: A hitchhiker's guide to the mixed model analysis of
randomized experiments. J. Agron. Crop Sci. 189, 310—322.

We consulted a biometrics expert. Data pooling is correct for our research. We provided additional explanations and sources for the editor.

Line 207, As one of the reviewers noted, 7% is really negligible in terms of herbicide efficacy. But As noted by the authors (Table 2), it appears that freezing treatments reduced the tolerance of wheat to the ACCase inhibiting herbicide, suggesting using 2-fold the recommended rate would definitely damage the crop.

We agree with the reviewer. At the same time, we do not mention the use of 2-fold the recommended dose. In the Discussion section, we have discussed the essence of the topic.

Line 228 In Table 1, the authors indicated the significance levels using asterisks; however, it is not clear what the asterisks represent. I assume they represent the significance of the parameters in the model rather than the differences between treatments. The authors need to use an appropriate statistical analysis to compare the estimated parameters between treatments, and the significant differences between treatments can be demonstrated using letters "e.g. a, b, etc.).

The requested information was added to the Table 1.

Line 272 "However, LD10 of wheat in" The authors must include the dose-response results for wheat treated with both herbicides under all three conditions.

Before submitting the MS, we had drawn a dose-response diagram for wheat. It is not correct at all. In this case, we will have a graph with an almost straight line (no slope) because the reduction of survival and dry weight in wheat is much less than that of weeds. Instead, we have used LD_{10} and selected the index.

Line 286 "nights before and after spray reduces the efficacy of this herbicide compared to NF." This statement is misleading. First of all, the differences in LD50 values appear to be negligible. Secondly, there is natural variation among the individuals of a population resulting in varied responses to herbicides. So such a low difference can be due to variability among individuals.

The difference between LD_{50} (NF and FBS) in clodinafop-propargyl is slight, and we mentioned this in the manuscript. Most of our emphasis in clodinafop-propargyl is on LD_{50} more in FAS. The requested information was added to the text.

Lines 289-289 "Freezing treatment for three nights before and after spraying of clethodim herbicide (the mode of action similar to clodinafop-propargyl) led to an increase in LD50 ". In the manuscript by Saini et al. 2017, the authors claimed that "Addition of frost treatments did not markedly affect survival of the S population" and eventually noted no significant differences. I feel in the current manuscript, the authors have misinterpreted their results.

See Table 2 of Saini's paper (R population). Our interpretation is correct.

Line 300- 301 “Herbicide translocation in the phloem in freezing conditions could decline due to damage to the phloem cells” The freezing treatment only lasted for 3 days; thus, I suspect impaired translocation could explain the outcome of this research. I guess in your case, freezing temperature reduced herbicide absorption.

We suspect that absorption disturbance is the main cause of efficiency reduction for the FBS. However, the reviewer's opinion is also mentioned in the discussion section.

Lines 314-322, 333-337 and 360-361 The physiological mechanisms underlying the response of plants to frost are different from those of low (cold) temperatures. So the response of plants to herbicides would be different under freezing versus low temperatures. For instance, for turnipweed, the freezing treatment before herbicide application reduced the efficacy of 2,4-d and MCPA treatment later. As there were no herbicide molecules in the plants at the time of freezing treatment, reduced translocation of the herbicide can not solely explain reduced herbicide efficacy in FBS plants.

We have made crucial assumptions, and this is justified. Differences in the response of a plant species are also possible. Hence, our justification may also be entirely correct.

Line 370 “their effectiveness in controlling narrow leaf…” you only studied one grass species. So this may not be true for other “narrow-leaved” species.

We agree with the reviewer, but please note our sentence:
Overall, according to the results of this experiment, it can be stated that FAS with ACCase inhibitor herbicides, such as clodinafop-propargyl, <u>may reduce</u> their effectiveness in controlling narrow leaf weeds such as winter wild oat .
Our personal experience has shown this essentially (anecdotal evidence). However, we need more studies in this field.

Line 374-375 “Therefore, the use of these herbicides in the mentioned conditions (FBS for clodinafop-propargyl and FAS for 2,4-D plus MCPA) is recommended.” This is misleading, as it appears the desirable crop (wheat) loses its tolerance to both herbicides under both freezing conditions. So, it is not a good idea to use these herbicides under freezing conditions to control weed species in wheat. Please revise accordingly.

Among the tried conditions, we have mentioned the best treatments. Please note that for us, the *Selectivity index* is more critical than LD_{10}. According to Table 2, this suggestion is better compared to other conditions. However, the reviewer's statement is also considered in different parts of the Discussion.

Reviewer #5: The authors incorporated the comments of both reviewers in true sense. The revised draft shaped well and can be accepted for publication.

We look forward to hearing from you regarding our submission. We would be glad to respond to any further questions and comments that you may have.

Manuscript Number: **PONE-D-21-33766**

Manuscript title: **Freezing stress affects the efficacy of clodinafop-propargyl and 2,4-D plus MCPA on wild oat (*Avena ludoviciana* Durieu) and turnipweed [*Rapistrum rugosum* (L.) All.] in wheat (*Triticum aestivum* L.)**

Dear Dr Uludag,

I want you to know how much I value your comments. Thank you very much for organizing the review of our manuscript. We have addressed all the comments/issues raised by the reviewers. The suggestions have improved the manuscript significantly. Our specific responses are given below. We hope to hear from you soon.

Sincerely,
Corresponding author

I carefully reviewed your revisions/comments. I applied most of them and presented answers for some below.

Editor's comments

Is it not controversial with line 30-31? "FBS caused 7% survival" you have mentioned

- " The requested revision was added to the text. According to the results of LD_{50}"

Is this not contraversial with abstract? As far as I understood NF and FBS caused 50% control similar for NF and FBS but FAS required more herbicide to kill 50% of the population.

According to Table 1, You should mention in the abstract clerary the difference between two herbicides/weeds because one needs hghre rate in FAS the other in FBS.

- The last sentence of the abstract was revised based on LD_{50}.

You can mention in the abstract that FR50 and LD50 gave the similar outcomes. I think the final interpretation will not change for both outputs.

- We mentioned this result in another way in the Abstract.
  "The LD_{50} (dose required to control 50% of individuals in the population) and GR_{50} (dose causing 50% growth reduction of plants) rankings were NF<FBS<FAS for clodinafop-propargyl and NF<FAS<FBS for 2,4-D plus MCPA."

Your data does not says twice. It is a value between x and 2x according to tables and figures you have given.

In addition is it FAS or FBS. I confused much.

Please see Figure 4 (A). For complete control of wild oat in FBS treatment, double the recommended dose (128 g ai ha^{-1}) is needed, and the sentence is correct.Is it not controversial with former sentence?

- Please see Figure 4

. This sentence is also controversial with the first sentence. I think the first sentence is not correct.

- Please note that the first sentence is related to Figure 4. The discussion of LD_{50} is different and clarified in Table 1.

It is confusing for me FAS requires this not FBS. Am I wrong in interpretation of figure 4 and Table 1?

- Please see Figure 4 (B). The sentence is correct.
-

This is what I have seen in your results but I mentioned above that some of your statements are controversial with this statetment. Check your abstract as well.

Check the previous sentence. According to GR_{50}, this sentence is mentioned. The sentence was improved.When you discusss effect of herbicides under freezing use all data together. You can add this paragraph above paragraphs. Because LD50, GR50, potency, all shows the same conclusion

I think using less word to explain your data makse it easier for the readers.

- Yes, I accept your opinion. We did this in the Conclusions. It confirms the relative potency of the previous sections.

This is eactly what your data shows. Some sentemces I have shown above are contoversial. Could you please check them carefully.

- I appreciate your attention. I carefully checked different parts of the MS. Ambiguities are resolved when we refer to figures and tables. I have made some changes to the paper.

I do not think so this is result of your experiment for clodinafop. FBS and NF is not statistically different for clodinaforp. please follow the what statstics tell.

- The sentence was deleted.

I recommend you that please avoid generilazation. You can say an ACCase inhibitor clodinafop... It helps your aim without generilazation. As you know not all chemistries have similar effects.

- Yes, your opinion is correct. Our sentence is also such that it does not refer to all the herbicides of this family.

As I mentioned above use winter wild oat, a narrow leaf weed. It helps you avoiding unnecessary genetralization.

- The requested revision was applied to the text.

I do not think you need this psrt.

- The sentence was deleted.

I think this is not discussed in your manuscript. Instead, you can say that these things should be researched .

- The requested revision was applied to the text.
