# Supplementary material for: Freezing stress affects the efficacy of clodinafop-propargyl and 2,4-D plus MCPA on wild oat (Avena ludoviciana Durieu) and turnipweed [Rapistrum rugosum (L.) All.] in wheat (Triticum aestivum L.)
Source: PLoS One. 2022 Oct 6;17(10):e0274945. doi: 10.1371/journal.pone.0274945 (PMC9536574; doi:10.1371/journal.pone.0274945)
Supplement: Attachment — Submitted filename: Response to Reviewers.docx [file pone.0274945.s002.docx]

Manuscript Number: **PONE-D-21-33766**

Manuscript title: **Freezing stress affects the efficacy of clodinafop-propargyl and 2*,*4*-*D plus MCPA on wild oat and turnipweed in wheat**

Dear Editor

Thank you very much for arranging the review of our manuscript. We greatly appreciate the reviewers for their complimentary comments and suggestions. We hope that you find our responses satisfactory and that the manuscript is now acceptable.

Sincerely,

Mehdi Rastgoo

Associate professor,

Department of Agrotechnology, Faculty of Agriculture, Ferdowsi University of Mashhad, Iran

Corresponding author

**Response to the comments of the reviewer #1**

We would like to thank the reviewer for the careful and thorough reading of this manuscript and the thoughtful comments and constructive suggestions, which help improve the manuscript quality. As indicated below, we have checked all the general and specific comments provided by the referees and have made necessary changes according to their indications, which are highlighted in yellow.

**Reviewer #1**: Unpredicted variability in temperature is associated with frequent extreme low-temperature events. And it is threatening global food security. Due to the lack of information on the efficacy of herbicides under freezing stress, this research was conducted to evaluate the efficacy of clodinafop-propargyl and 2,4-D plus MCPA on wild oat and turnipweed under freezing stress before and after herbicide application. The research results have guiding significance for production, but the article still needs to be revised and improved.

（1）The introduction part should increase the impact of global climate change on the occurrence of low temperature adversity events. And its impact on wheat production.

- The requested information has been added to the text.

（2）Experimental site and plant material part：Explain the amount of fertilizer used and the ratio of N, P, K.

- We did not use fertilizer in this experiment.

（3）The source of the meteorological data in Figure 1 should be clearly explained.

- The requested information has been added to the text.

（4）Results part: The description of the results needs to be more in-depth. For example, how much does FBS significantly increase or decrease compared to NF?

- We respect the reviewer's opinion, but according to the results of the clodinafop-propargyl, we have written the requested information in another expression (gray highlight), and its re-expression is repetitive. But in the case of 2,4-D plus MCPA, the results were explained in more detail, and the requested information was added to the text (yellow highlight).

（5）The discussion part is not deep enough, some physiological discussions should be added.

- The requested information has been added to the text.

（6）The description in the conclusion part needs to be refined to increase readability.

- The requested information was added to the text. Clear text added.

（7）There are grammatical and editorial issues somewhere in this paper. Many of them could cause difficulties to the readers.

- We respect the reviewer's opinion, but a Native English-speaker colleague has edited the manuscript's text. We rechecked the text.

（8）The article does not have data on wheat yield. It would be very good if we could increase the data on the effects of herbicides before and after low temperature on wheat yield.

- It is an exciting suggestion. We are going to study the wheat yield in future studies. In this study, we focused on the effect of freezing stress on herbicide efficacy in the early stages of plant species. In general, it is predicted that wheat yield will decrease due to herbicide efficiency and its adverse effects on the crop (selectivity index).

**Response to the comments of the reviewer #2**

We greatly appreciate the reviewer's positive comments. As indicated below, we have checked all the general and specific comments provided by the referees and have made necessary changes according to their indications, which are highlighted in yellow.

**Reviewer #2**: Dear Authors,

Reviewer comments PONE-D-21-33766

The manuscript entitled „Freezing stress affects the efficacy of clodinafop-propargyl and 2,4-D plus MCPA on wild oat and turnipweed in wheat“ represents a useful study focused on the impact of freezing stress on the efficiency of two herbicides treatments, clodinafop-propargyl and 2,4-D plus MCPA treatments, resulting in the statement that freezing stress attenuates the efficiency of herbicides in weed elimination as indicated by a decreased selectivity index. This result also indicates that freezing increases potential harmfulness of these herbicides for wheat as a crop.

I have only a few minor comments on the present manuscript:

In Materials and methods, Statistical analysis, as well as in Results, Table 1, a kind of a post-hoc test following ANOVA analysis used for determination of significant differences, has to be specified both in Materials and methods, Statistical analysis section and in results, Table 1 legend. Moreover, in Table 1 legend, it has to be clearly stated that the presented data represent mean±SE, and the number of replicates has to be specified.

- The requested information has been added to the text and Table 1.

Further formal comments on the manuscript text:

Abstract, lines 37-38: Add a comma both preceding and following the words „for 2,4-D plus MCPA,…“

- The requested revision was applied to the text.

Introduction, line 53: Use the term „winter cereals“ instead of „autumn cereals“ for autumn-sown cereals which have vernalization requirement thus they require winter temperatures prior to flowering.

- The requested revision was applied to the text.

Introduction, line 59: Use the term „optimum climatic conditions“ instead of „normal climatic conditions“.

- The requested revision was applied to the text.

Materials and methods, line 109: Replace the verb „obtain“ with „induce“ in the statement „To induce plant cold acclimation, the plants were kept outdoors,…“

- The requested revision was applied to the text.

Materials and methods, line 125: The authors wrote that „Freezing treatment was performed using a thermogradient freezer.“ However, basic information on the freezer has to be given including the manufacturer (company). Was the freezing treatment always just to -4 °C?

- The requested information was added to the text.
- Trend of temperature decrease in thermogradient freezer during the night from 7:00 P.M. to 5:00 A.M. (Please see Fig. 2 and gray highlight)

| **Time** | **Temperature (℃)** |
| --- | --- |
| 7 P.M. | 5 |
| 12 A.M. | Reach a temperature of 0 |
| 1 A.M. | kept at 0 |
| 2 A.M. | Reach a temperature of -2 |
| 3 A.M. | kept at -2 |
| 4 A.M. | Reach a temperature of -4 |
| 5 A.M. | kept at -4 |
| 5:30 A.M. | Removed from the freezer |

Line 175: Remove „the“ preceding the term „relative potency“.

- The requested revision was applied to the text.

Line 182: Modify the statement as follows: „Relative potency < 1 indicates the decreased effectiveness of the herbicides under freezing stress with respect to optimum conditions.“

- The requested revision was applied to the text.

Results, Figure 4 legend, line 209; Figure 5 legend, line 246: Write rather „64 g ha-1“, not „64 g ai ha-1“.

- The requested revision was applied to the text.

Results, line 252: Use a plural form of the verb „were“ instead of „was“ in the statement „The GR50 of NF, FBS, and FAS were 176, 299, and 208 g ha-1, respectively…“

-	The requested revision was applied to the text.

Line 263: Remove „the“ preceding the term „selectivity index“ and modify the statement as follows: „As shown in Table 2, selectivity index values for clodinafop-propargyl in NF, FBS, and FAS treatments were 2.4, 0.91, and 0.78, respectively, and, for 2,4-D plus MCPA, they were 2.6, 0.12, and 0.88, respectively.“

-	The requested revision was applied to the text.

Discussion, line 299: Remove „the“ Preceding the chemical name „clodinafop propargyl“.

-	The requested revision was applied to the text.

Discussion, line 332: Replace the word „less“ with „lower“ in the statement „The relative potency values of clodinafop-propargyl and 2,4-D plus MCPA were lower than one under both FBS and FAS treatments in the present experiment.“

- The requested revision was applied to the text.

Discussion, line 343: Modify the statement into a plural form as follows: „The LD10 values of 2,4-D plus MCPA in NF were 89% and 48% higher compared to FBS and FAS, respectively.“

- The requested revision was applied to the text.

We look forward to hearing from you regarding our submission. We would be glad to respond to any further questions and comments that you may have.
