# Supplementary material for: Freezing stress affects the efficacy of clodinafop-propargyl and 2,4-D plus MCPA on wild oat (Avena ludoviciana Durieu) and turnipweed [Rapistrum rugosum (L.) All.] in wheat (Triticum aestivum L.)
Source: PLoS One. 2022 Oct 6;17(10):e0274945. doi: 10.1371/journal.pone.0274945 (PMC9536574; doi:10.1371/journal.pone.0274945)
Supplement: Attachment — Submitted filename: Response to Reviewers.docx [file pone.0274945.s005.docx]

Manuscript Number: **PONE-D-21-33766**

Manuscript title: **Freezing stress affects the efficacy of clodinafop-propargyl and 2*,*4*-*D plus MCPA on wild oat and turnipweed in wheat**

Dear Editor

We sincerely appreciate all valuable comments and suggestions, which helped us to improve the quality of the article. We hope that you find our responses satisfactory and that the manuscript is now acceptable.

Sincerely,

Mehdi Rastgoo

Associate professor,

Department of Agrotechnology, Faculty of Agriculture, Ferdowsi University of Mashhad, Iran

Corresponding author

*General comment*

Your reasoning why you have chosen mix of two auxinic ingredients and discussion needs to be improved although it has been successful already. You need to use latin names of weeds in title and abstract as well. You need further information to be given about herbicides such as safener in clodinafop if there is, acid equvalent rates of auxicinic herbicides, separated amounts of 2,4-D and MCPA. I am not sure if you need both ED50 and GR50. By the way, no GR50 calculation in your materials and methods.

- The mix of these two auxinic ingredients is one of the most important compounds in the control of broadleaf weeds in wheat fields globally, whose efficiency is generally affected by abiotic stresses. We feel that we have answered this question well in the text of the paper.
- Latin names of weeds were added as Titles and Abstracts.
- The requested revision was applied to the 2,4-D (acid equvalent rates of auxicinic herbicides).
- In this experiment, we calculated two important survival and dry weight parameters. We calculated both parameters because sometimes the herbicide does not lead to complete control of the weed plant but reduces the growth of the plant and its dry weight. Therefore, the optimal effect of the herbicide may be hidden in reducing the dry weight of the herbicide.
- In the Dose-response experiments section, we have mentioned how to calculate both parameters (LD50 and GR50).

**Response to the comments of the reviewer #2**

We would like to thank the reviewer for the careful and thorough reading of this manuscript and the thoughtful comments and constructive suggestions, which help improve the manuscript quality. As indicated below, we have checked all the general and specific comments provided by the referees and have made necessary changes according to their indications, which are highlighted in yellow.

**Reviewer #2**: Reviewer comments PONE-D-21-33766R1

The revised manuscript entitled „Freezing stress affects the efficacy of clodinafop-propargyl and 2,4-D plus MCPA on wild oat and turnipweed in wheat“ was significantly improved by the authors according to my previous comments.

I think that the revised manuscript is suitable for publication in PLoS One.

I have only a few formal comments on the revised manuscript which are provided below:

Abstract, line 23: The abbreviations „2,4-D“ and „MCPA“ have to be explained when used for the first time.

- We respect the reviewer's opinion, but this is the common name of the herbicide and should be mentioned without further explanation. It is not common to write its full name in a scientific article. However their full names are as follows.

2,4-Dichlorophenoxyacetic acid *and* 2-methyl-4-chlorophenoxyacetic acid

Abstract, line 29 and the further text including the figures: The „ai“ used in the „g ai ha-1“ has to be explained. What is the difference between „g ai ha-1“ and „g ha-1“??

- Both units are correct. Usually, g ai ha^-1^ is first mentioned in articles and then g ha^-1^.

Line 134: Add a comma following the words „Each morning at 5:30 A.M., …“

-	The requested revision was applied to the text.

Line 337: Use the term „optimal non-freezing conditions“ instead of „normal conditions.“

- The requested revision was applied to the text.

**Response to the comments of the reviewer #3**

In general, the paper is interesting and the topic nice. However, several serious issues ought to be addressed. First of all, I absolutely disagree with statements of the authors (in the abstract and the text) that the occurrence of freezing stress around herbicides application is one of the most important factors influencing their performance. Many more factors (rate, growth stage, adjuvant, weed species, water volume, pressure etc) play a significant role on that. Moreover, survival 7% means efficacy of 93%, farmers do not need to double the rate to achieve a complete control (usually farmers are satisfied with an efficacy of 90-95%). In wild oat authors say that under no freezing (NF) and freezing after spray (FAS) conditions, winter wild oat was completely controlled with the recommended dose of clodinafop-propargyl, while at FBS control was 93%. Why the ranking of LD50 and GR50 was different (meaning the FBS between the two others)? There is an important confusion. Furthermore, I would encourage authors a) to justify their choice to evaluate an ACCase inhibitor alone and a mixture of auxinic herbicides (and not an individual)? and b) substantially revise and improve the discussion section in order to talk more about the potential mechanism behind that reduced efficacy and the potential generalization of their results (since in many cases farmers chose to spray under low temperatures since the notice an increased susceptibility of several weeds to glyphosate and other herbicides).

- Many factors affect the effectiveness of herbicides. We also mentioned throughout the text that frost stress is "one of the most important factors (not the only one factor)" influencing the performance of herbicides.
- We completely agree with the esteemed referee regarding the ideal amount of weed control at the recommended dose (90 to 95%). But we did not know the result before the test, and naturally, we had to try twice the recommended dose. However, it is not recommended with 93% control of double dose use, and we discussed this issue in the article.
- In non-stress freezing and freezing after spraying with the recommended dose of clodinafop-propargil, the survival rate was zero (Figure 4), but the results were different for LD_50_ and GR_50_ (Table 1). Survival % and survival and dry weight 50% (LD_50_ and GR_50_) can vary according to their definition (more details in the results and discussion section). Survival % with LD_50_ and GR_50_ can differ depending on their definition.
- More information in the results and discussion section.

We look forward to hearing from you regarding our submission. We would be glad to respond to any further questions and comments that you may have.
