# Supplementary material for: Freezing stress affects the efficacy of clodinafop-propargyl and 2,4-D plus MCPA on wild oat (Avena ludoviciana Durieu) and turnipweed [Rapistrum rugosum (L.) All.] in wheat (Triticum aestivum L.)
Source: PLoS One. 2022 Oct 6;17(10):e0274945. doi: 10.1371/journal.pone.0274945 (PMC9536574; doi:10.1371/journal.pone.0274945)
Supplement: Attachment — Submitted filename: Response to Reviewers (3).docx [file pone.0274945.s006.docx]

Manuscript Number: **PONE-D-21-33766**

Manuscript title: **Freezing stress affects the efficacy of clodinafop-propargyl and 2,4-D plus MCPA on wild oat (*Avena ludoviciana* Durieu) and turnipweed [*Rapistrum rugosum* (L.) All.] in wheat (*Triticum aestivum* L.)**

Dear Dr Uludag

Thank you very much for organizing the review of our manuscript. We have addressed all the comments/issues raised by the reviewers and have made necessary changes according to their indications, which are highlighted in purple (new revision).

The opinions of reviewer 4 are extraordinary, and we do not agree with some of them. Our manuscript has been read several times by a Native English speaker and recognized weed science expert. He emphasized that there was no problem with the language of this manuscript. The reviewer should not criticize the language of the article with a general comment. If the language has a problem, it should mention exactly which sentence? Which section?

Our MS has been read several times by one of the top experts and editors in weed science (professor Chauhan). Isn't this the reviewer's expression strange?!

*Reviewer 4: Note that being a native English speaker is not synonymous with being a good writer.*

His opinion about statistical analysis shocked us. We used the most powerful method and software for analysis. Regarding the data pooling, the variance of the data of the two experiments is equal (based on Bartlett's or Levene’s test), and we can pool the data. You will find a simple search for high-quality sources. Data for both runs were pooled as no significant differences were observed between runs.

*- Arsham, H., & Lovric, M. (2011). Bartlett's Test. International encyclopedia of statistical science, 1, 87-88.*

*- Niyonzima, N., Bakke, S. S., Gregersen, I., Holm, S., Sandanger, Ø., Orrem, H. L., ... & Espevik, T. (2020). Cholesterol crystals use complement to increase NLRP3 signaling pathways in coronary and carotid atherosclerosis. EBioMedicine, 60, 102985.*

*-Mahajan, G., Rachaputi, R. C. N., & Chauhan, B. S. (2020). Horse purslane (Trianthema portulacastrum) control in pigeonpea with PRE and POST herbicides. Weed Technology, 34(5), 764-769.*

*-Mobli, A., Manalil, S., Khan, A. M., Jha, P., & Chauhan, B. S. (2020). Effect of emergence time on growth and fecundity of Rapistrum rugosum and Brassica tournefortii in the northern region of Australia. Scientific Reports, 10(1), 1-10.*

*- Sritan, K., & Phuenaree, B. (2021). A Comparison of Efficiency for Homogeneity of Variance Tests under Log-normal Distribution. Asian Journal of Applied Sciences, 9(4).*

We should not rely on a particular article from 19 years ago. We have explained this in a clear and complete analysis section. Note that none of the reviewers objected to our analysis except reviewer 4.

In all high-quality research, the use of standard error mean (SEM) is expected.

*Saini, R. K., Malone, J., Preston, C., & Gill, G. S. (2016). Frost reduces clethodim efficacy in clethodim-resistant rigid ryegrass (Lolium rigidum) populations. Weed Science, 64(2), 207-215.*

The SEM is used to determine the differences between more than one sample of data. It helps us estimate how well your sample data represents the whole population by measuring the accuracy with which the sample data represents a population using standard deviation. However, we added items like mean comparison (LSD test) to the manuscript (Table 1).

Apart from the above, we feel that the reviewer did not read the paper well. For example, we had three repetitions in each run, and each repetition had eight plants. The reviewer incorrectly stated eight plants in each repetition. In addition, despite our complete explanations and figures, the reviewer did not notice that the plants were exposed to freezing stress for three nights, which reached the minimum temperature (-4°C).

Reviewer 4 has requested the details of herbicides. Meanwhile, we have already provided all the valuable information in the paper (purple highlights in the Introduction and Experimental factors).

Sincerely,

Corresponding author

**p.s.** we have addressed the rest, and we expect to hear your final opinion after about a year and apply the comments of five reviewers. Naturally, you send each manuscript to each reviewer, who will have their unique comments and revisions. How long should this cycle continue? We emphasize that we applied all the comments of previous reviewers (as declared by reviewer 5). We hope that you find our responses satisfactory and that the manuscript is now acceptable.

*Review Comments to the Author*

Please use the space provided to explain your answers to the questions above. You may also include additional comments for the author, including concerns about dual publication, research ethics, or publication ethics. (Please upload your review as an attachment if it exceeds 20,000 characters)

***Reviewer #4:*** The manuscript investigated the response of two problematic weed species to herbicides under freezing conditions. I have realized that some other colleagues had reviewed the manuscript. I also realized that one of the reviewers raised a concern regarding the language of the manuscript, but the authors rebutted this comment and claimed that their manuscript had been edited by “a Native English-speaker colleague”. Please note that being a native English speaker is not synonymous with being a good writer. After reading the manuscript, I also feel the language is not comprehensive in many places and does not meet the minimum standard required by international journals. Hence, I recommend editing help from a language service.

We expressed our opinion about this to the academic editor.

*Specific comments*

Lines 24 and 68- Please spell out these abbreviated herbicide names. The idea is that the manuscript should be comprehensive to any readers, not only to those who share the same area of expertise as the authors.

The requested information was added to the text (2,4-Dichlorophenoxyacetic acid and 2-methyl-4-chlorophenoxyacetic acid). The clodinafop-propargyl is the full name of this herbicide and not an abbreviation.

Line 100 “seeds of winter wild oat and turnipweed” How many plants did you sample? Did the samples size sufficiently represent the population?

Weed seeds are collected from at least 500 plants, and the sample size sufficiently represents the population.

Line 105 “The seeds were placed in” I assume this refers to weed seed germination. How about wheat?

The requested information was added to the text.

Line 109 “same time on October 21 (first run) and 24 (second run) in 2019”. I would not call this “a repeat in time”. We usually repeat experiments in time to cover sufficient spatial and temporal variation. Repeating an experiment 3 days after the other does not meet this criterion.

We did not claim that the experiment was repeated in time. The examination is designed based on our purposes.

Line 111 Nutrient deficiency can affect herbicide efficiency. The authors claimed they had not added any fertilizer to the potting media. This needs to be justified in the manuscript; otherwise, it is a major flaw.

We agree with the reviewer, but a large part of the soil used was farm soil, which had suitable physiochemical. Also, note that the soil conditions were the same in all experiments. Hence, the results are reported under similar conditions.

Line 112 Inconsistency. In Line 106-108, the authors claimed that they had pregerminated the seed of each weed species, and subsequently, they transplanted the seedlings. But here, they claimed they planted the seed directly. Please justify.

Both weed species were pregerminated. Then Germinated turnipweed seeds were planted in a seedling tray. Germinated wild oat seeds were planted directly in the soil. Fully and clearly explained (Please see M&M).

Line 113 Only eight plants per replicate? Would this number of plants sufficiently represent the population sample? Since the authors estimated the LD50, I suspect the low number of plants used in this experiment would sufficiently represent the variability among individuals of sampled populations? How many pregerminated seeds did you place in each pot for turnipweed?

8 plants×3Rep = (24 plants) × 2 Run= 48 plants

It makes sense for a controlled trial.

Eight turnipweed seedlings transferred to pots (added to the text).

Line 115 “irrigated according to daily requirements” How?

Irrigation was sufficient (based on the soil requirement of a pot). ~ 400 ml per pot. In this way, the plants were not subjected to drought stress.

Line 114 How did you grow wheat?

It is obvious. The wheat seed does not require to break dormancy. It does not need pre-germination either. So it was direct cultivation.

Lines 132-133 “The pots were kept at -4℃ for 1 h and then removed from the freezer (Fig. 2).” I can`t follow this part. The plants were supposed to be kept in a freezer from 7pm to 5 am. But the authors remove the plants after 1 h exposure to -4 °C. Does this mean that the temperature reached to 4 °C at about 4 am?

We have provided clear explanations in the text. We have included figures 2 and 3 in the MS for clarification. The plants were exposed to the determined temperature gradient for three nights and reached the minimum temperature (-4°C).

Trend of temperature decrease in thermogradient freezer during the night from 7:00 P.M. to 5:00 A.M. (Please see Fig. 2)

| **Time** | **Temperature (℃)** |
| --- | --- |
| 7 P.M. | 5 |
| 12 A.M. | Reach a temperature of 0 |
| 1 A.M. | kept at 0 |
| 2 A.M. | Reach a temperature of -2 |
| 3 A.M. | kept at -2 |
| 4 A.M. | Reach a temperature of -4 |
| 5 A.M. | kept at -4 |
| 5:30 A.M. | Removed from the freezer |

Line 157 “herbicides” Please give more details than “herbicides”. What was the formulation? Did you use any wetting agents? What was the trade name of each herbicide? What was the portion of each herbicide in the mixture of 2,4-D and MCPA?

For details on herbicides, refer to the Introduction and Experimental factors We would have mentioned it in the paper if we had used agents. So we did not use any agent. The trade name of each herbicide is added to the text (Experimental factors). . The manufacturing company combines this herbicide (360 g ae.L^-1^ for 2,4-D and 315 g ae.L^-1^ for MCPA). We did not mix it.

Line 174 “the curve around e, and e is the effective dose”, The letter “e” is used in two different places in the equation. Which one represents the “effective dose”?

Both indicate the effective dose.

Line 195- 196 “Afterward, differences between treatments …” This is not really a solid approach to compare treatments statistically.

In all high-quality research, a standard error mean (SEM) is expected. We need SEM values for a dose-response graph, and this is necessary. However, we added items like mean comparison (LSD test) to the manuscript (Table 1).

Line 197-198 Pooling data from two experiments is a poor practice and results in missing many effects that are needed. The most appropriate statistical approach is to use a “mixed model analysis”. Please see the manuscript below for more information.
Piepho, H. P., A. Bu¨chse, and K. Emrich, 2003: A hitchhiker’s guide to the mixed model analysis of
randomized experiments. J. Agron. Crop Sci. 189, 310—322.

We consulted a biometrics expert. Data pooling is correct for our research. We provided additional explanations and sources for the editor.

Line 207, As one of the reviewers noted, 7% is really negligible in terms of herbicide efficacy. But As noted by the authors (Table 2), it appears that freezing treatments reduced the tolerance of wheat to the ACCase inhibiting herbicide, suggesting using 2-fold the recommended rate would definitely damage the crop.

We agree with the reviewer. At the same time, we do not mention the use of 2-fold the recommended dose. In the Discussion section, we have discussed the essence of the topic.

Line 228 In Table 1, the authors indicated the significance levels using asterisks; however, it is not clear what the asterisks represent. I assume they represent the significance of the parameters in the model rather than the differences between treatments. The authors need to use an appropriate statistical analysis to compare the estimated parameters between treatments, and the significant differences between treatments can be demonstrated using letters “e.g. a, b, etc.).

The requested information was added to the Table 1.

Line 272 “However, LD10 of wheat in” The authors must include the dose-response results for wheat treated with both herbicides under all three conditions.

Before submitting the MS, we had drawn a dose-response diagram for wheat. It is not correct at all. In this case, we will have a graph with an almost straight line (no slope) because the reduction of survival and dry weight in wheat is much less than that of weeds. Instead, we have used LD_10_ and selected the index.

Line 286 “nights before and after spray reduces the efficacy of this herbicide compared to NF.” This statement is misleading. First of all, the differences in LD50 values appear to be negligible. Secondly, there is natural variation among the individuals of a population resulting in varied responses to herbicides. So such a low difference can be due to variability among individuals.

The difference between LD_50_ (NF and FBS) in clodinafop-propargyl is slight, and we mentioned this in the manuscript. Most of our emphasis in clodinafop-propargyl is on LD_50_ more in FAS. The requested information was added to the text.

Lines 289-289 “Freezing treatment for three nights before and after spraying of clethodim herbicide (the mode of action similar to clodinafop-propargyl) led to an increase in LD50 “. In the manuscript by Saini et al. 2017, the authors claimed that “Addition of frost treatments did not markedly affect survival of the S population” and eventually noted no significant differences. I feel in the current manuscript, the authors have misinterpreted their results.

See Table 2 of Saini's paper (R population). Our interpretation is correct.

Line 300- 301 “Herbicide translocation in the phloem in freezing conditions could decline due to damage to the phloem cells” The freezing treatment only lasted for 3 days; thus, I suspect impaired translocation could explain the outcome of this research. I guess in your case, freezing temperature reduced herbicide absorption.

We suspect that absorption disturbance is the main cause of efficiency reduction for the FBS. However, the reviewer's opinion is also mentioned in the discussion section.

Lines 314-322, 333-337 and 360-361 The physiological mechanisms underlying the response of plants to frost are different from those of low (cold) temperatures. So the response of plants to herbicides would be different under freezing versus low temperatures. For instance, for turnipweed, the freezing treatment before herbicide application reduced the efficacy of 2,4-d and MCPA treatment later. As there were no herbicide molecules in the plants at the time of freezing treatment, reduced translocation of the herbicide can not solely explain reduced herbicide efficacy in FBS plants.

We have made crucial assumptions, and this is justified. Differences in the response of a plant species are also possible. Hence, our justification may also be entirely correct.

Line 370 “their effectiveness in controlling narrow leaf…” you only studied one grass species. So this may not be true for other “narrow-leaved” species.

We agree with the reviewer, but please note our sentence:

Overall, according to the results of this experiment, it can be stated that FAS with ACCase inhibitor herbicides, such as clodinafop-propargyl, may reduce their effectiveness in controlling narrow leaf weeds such as winter wild oat.

Our personal experience has shown this essentially (anecdotal evidence). However, we need more studies in this field.

Line 374-375 “Therefore, the use of these herbicides in the mentioned conditions (FBS for clodinafop-propargyl and FAS for 2,4-D plus MCPA) is recommended.” This is misleading, as it appears the desirable crop (wheat) loses its tolerance to both herbicides under both freezing conditions. So, it is not a good idea to use these herbicides under freezing conditions to control weed species in wheat. Please revise accordingly.

Among the tried conditions, we have mentioned the best treatments. Please note that for us, the Selectivity index is more critical than LD_10_. According to Table 2, this suggestion is better compared to other conditions. However, the reviewer's statement is also considered in different parts of the Discussion.

Reviewer #5: The authors incorporated the comments of both reviewers in true sense. The revised draft shaped well and can be accepted for publication.

We look forward to hearing from you regarding our submission. We would be glad to respond to any further questions and comments that you may have.
