# Supplementary material for: Freezing stress affects the efficacy of clodinafop-propargyl and 2,4-D plus MCPA on wild oat (Avena ludoviciana Durieu) and turnipweed [Rapistrum rugosum (L.) All.] in wheat (Triticum aestivum L.)
Source: PLoS One. 2022 Oct 6;17(10):e0274945. doi: 10.1371/journal.pone.0274945 (PMC9536574; doi:10.1371/journal.pone.0274945)
Supplement: Attachment — Submitted filename: Reviewer comments PONE-D-21-33766.docx [file pone.0274945.s001.docx]

Reviewer comments PONE-D-21-33766

The manuscript entitled „Freezing stress affects the efficacy of clodinafop-propargyl and 2,4-D plus MCPA on wild oat and turnipweed in wheat“ represents a useful study focused on the impact of freezing stress on the efficiency of two herbicides treatments, clodinafop-propargyl and 2,4-D plus MCPA treatments, resulting in the statement that freezing stress attenuates the efficiency of herbicides in weed elimination as indicated by a decreased selectivity index. This result also indicates that freezing increases potential harmfulness of these herbicides for wheat as a crop.

I have only a few minor comments on the present manuscript:

In Materials and methods, Statistical analysis, as well as in Results, Table 1, a kind of a post-hoc test following ANOVA analysis used for determination of significant differences, has to be specified both in Materials and methods, Statistical analysis section and in results, Table 1 legend. Moreover, in Table 1 legend, it has to be clearly stated that the presented data represent mean±SE, and the number of replicates has to be specified.

Further formal comments on the manuscript text:

Abstract, lines 37-38: Add a comma both preceding and following the words „for 2,4-D plus MCPA,…“

Introduction, line 53: Use the term „winter cereals“ instead of „autumn cereals“ for autumn-sown cereals which have vernalization requirement thus they require winter temperatures prior to flowering.

Introduction, line 59: Use the term „optimum climatic conditions“ instead of „normal climatic conditions“.

Materials and methods, line 109: Replace the verb „obtain“ with „induce“ in the statement „To induce plant cold acclimation, the plants were kept outdoors,…“

Materials and methods, line 125: The authors wrote that „Freezing treatment was performed using a thermogradient freezer.“ However, basic information on the freezer has to be given including the manufacturer (company). Was the freezing treatment always just to -4 °C?

Line 175: Remove „the“ preceding the term „relative potency“.

Line 182: Mdify the statement as follows: „Relative potency < 1 indicates the decreased effectiveness of the herbicides under freezing stress with respect to optimum conditions.“

Results, Figure 4 legend, line 209; Figure 5 legend, line 246: Write rather „64 g ha^-1^“, not „64 g ai ha^-1^“.

Results, line 252: Use a plural form of the verb „were“ instead of „was“ in the statement „The GR50 of NF, FBS, and FAS were 176, 299, and 208 g ha^-1^, respectively…“

Line 263: Remove „the“ preceding the term „selectivity index“ and modify the statement as follows: „As shown in Table 2, selectivity index values for clodinafop-propargyl in NF, FBS, and FAS treatments were 2.4, 0.91, and 0.78, respectively, and, for 2,4-D plus MCPA, they were 2.6, 0.12, and 0.88, respectively.“

Discussion, line 299: Remove „the“ Preceding the chemical name „clodinafop propargyl“.

Discussion, line 332: Replace the word „less“ with „lower“ in the statement „The relative potency values of clodinafop-propargyl and 2,4-D plus MCPA were lower than one under both FBS and FAS treatments in the present experiment.“

Discussion, line 343: Modify the statement into a plural form as follows: „The LD10 values of 2,4-D plus MCPA in NF were 89% and 48% higher compared to FBS and FAS, respectively.“

Final recommendation: Accept after a minor revision.
