# Supplementary material for: Freezing stress affects the efficacy of clodinafop-propargyl and 2,4-D plus MCPA on wild oat (Avena ludoviciana Durieu) and turnipweed [Rapistrum rugosum (L.) All.] in wheat (Triticum aestivum L.)
Source: PLoS One. 2022 Oct 6;17(10):e0274945. doi: 10.1371/journal.pone.0274945 (PMC9536574; doi:10.1371/journal.pone.0274945)
Supplement: Attachment — Submitted filename: Reviewer comments PONE-D-21-33766R1.docx [file pone.0274945.s003.docx]

Reviewer comments PONE-D-21-33766R1

The revised manuscript entitled „Freezing stress affects the efficacy of clodinafop-propargyl and 2,4-D plus MCPA on wild oat and turnipweed in wheat“ was significantly improved by the authors according to my previous comments.

I think that the revised manuscript is suitable for publication in PLoS One.

I have only a few formal comments on the revised manuscript which are provided below:

Abstract, line 23: The abbreviations „2,4-D“ and „MCPA“ have to be explained when used for the first time.

Abstract, line 29 and the further text including the figures: The „ai“ used in the „g ai ha^-1^“ has to be explained. What is the difference between „g ai ha^-1^“ and „g ha^-1^“??

Line 134: Add a comma following the words „Each morning at 5:30 A.M., …“

Line 337: Use the term „optimal non-freezing conditions“ instead of „normal conditions.“

Final recommendation: Accept after a formal revision.
