# Supplementary material for: Freezing stress affects the efficacy of clodinafop-propargyl and 2,4-D plus MCPA on wild oat (Avena ludoviciana Durieu) and turnipweed [Rapistrum rugosum (L.) All.] in wheat (Triticum aestivum L.)
Source: PLoS One. 2022 Oct 6;17(10):e0274945. doi: 10.1371/journal.pone.0274945 (PMC9536574; doi:10.1371/journal.pone.0274945)
Supplement: Attachment — Submitted filename: Reviewer comments PONE-D-21-33766R3.docx [file pone.0274945.s007.docx]

Reviewer comments PONE-D-21-33766R3

The revised manuscript entitled „Freezing stress affects the efficacy of clodinafop-propargyl and 2,4-D plus MCPA on wild oat (Avena ludoviciana Durieu) and turnipweed (Rapistrum rugosum (L.) All.) in wheat“ was improved by the authors in accordnace with my and other reviewers´ comments.

I have no further major comment on the revised manuscript.

I have only a few formal comments on the revised manuscript which are given below:

Introduction, line 64: Add the word „fact“ in the statement „Based on this fact,…“

Materials and methods, line 116: Add the verb „were“ in the statement „…were transferred to pots…“

Discussion, line 310: Add the word „respectively“ at the end of the statemnt „the efficacy of 2,4-D plus MCPA under FBS and FAS was 1.6 and 1.1 times lower compared to NF, respectively.“

Final recommendation: Accept.
